# Novel 1′-homo-*N*-2′-deoxy-α-nucleosides: synthesis, characterization and biological activity†

**DOI:** 10.1039/d0ra03254a

**Published:** 2020-04-21

**Authors:** Alejandro Carnero, Virginia Martín-Nieves, Yogesh S. Sanghvi, Olivia O. Russel, Leda Bassit, Raymond F. Schinazi, Susana Fernández, Miguel Ferrero

**Affiliations:** Departamento de Química Orgánica e Inorgánica, Universidad de Oviedo 33006-Oviedo Asturias Spain mferrero@uniovi.es fernandezgsusana@uniovi.es; Rasayan Inc. 2802 Crystal Ridge Road Encinitas CA 92024-6615 USA; Center for AIDS Research, Laboratory of Biochemical Pharmacology, Department of Pediatrics, Emory University School of Medicine Atlanta GA 30322 USA

## Abstract

For the first time, a series of novel 1′-homo-*N*-2′-deoxy-α-nucleosides containing natural nucleobases as well as 5-fluoro and 5-iodopyrimidine analogs have been synthesized in an efficient manner. Additionally, a high yield protocol for the assembly of a dimeric scaffold containing two sugar moieties linked to the *N*-1 and *N*-3 positions of a single pyrimidine base has been accomplished. The structures of the novel homonucleosides were established by a single crystal X-ray structure of 1′-homo-*N*-2′-deoxy-α-adenosine and NMR studies. The biological activity of these 1′-homo-*N*-2′-deoxy-α-nucleosides as antiviral (HIV-1 and HBV) and cytotoxic studies was measured in multiple cell systems. The unique structure and easy accessibility of these compounds may allow their use in the design of new nucleoside analogs with potential biological activity and as a scaffold for combinatorial chemistry.

## Abstract 2

Novel 1′-homo-*N*-2′-deoxy-α-nucleosides and dimers.

## Graphical abstract

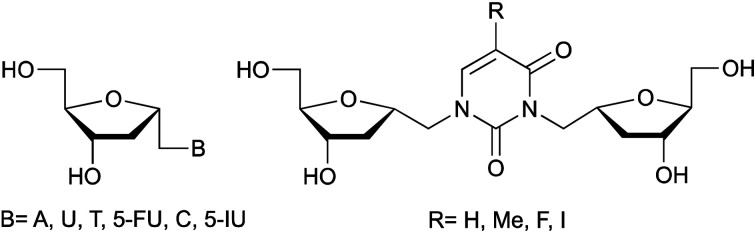

## 1. Introduction

Nucleosides are building-blocks of nucleic acids, where these molecules play a key role in the genetic process of all living organisms. Natural nucleosides offer a unique framework for the design of novel therapeutic drugs due to their central function in biological processes. As a result, over thirty chemically modified nucleosides and their analogs are on the market for the treatment of cancer and viral infections.^1^ Undoubtedly nucleoside scaffolds have been an excellent starting point for introducing chemical modifications leading to the discovery of biologically functional molecules. In nature, nucleosides appear mainly as β-anomers and rarely as the α-anomers. More recently the syntheses of α-anomeric nucleosides have attracted wide attention due to their unique properties and potential applications.^2^ Additionally, the position of the glycosidic bond could be altered by inserting another carbon atom to create 1′-homo-*N*-nucleoside structures. This structural motif imparts several unique features such as the absence of an anomeric effect, enzymatic and chemical stability of the glycosidic bond, conformational flexibility, extra lipophilicity and increased distance between the 5′-hydroxyl group and the base.^3^ In our on-going quest for the design and synthesis of novel nucleoside analogs, we embarked on the synthesis of less common modification 1′-homo-*N*-2′-deoxy-α-nucleosides where two features are incorporated in a single molecule. To the best of our knowledge, this is the first report on the synthesis of 1′-homo-*N*-2′-deoxy-α-nucleosides containing all four natural bases in an efficient manner. Additionally, herein we describe the synthesis of a bifunctional scaffold where two sugar units were linked to a single pyrimidine base.

Considering the importance of nucleoside analogs as bioactive motif, we reasoned that new methods for the synthesis of 1′-homo-*N*-2′-deoxy-α-nucleosides are warranted. Hitherto, two major methods were used to prepare 1′-homo-*N*-nucleosides: (i) introduction of the nucleobase by nucleophilic displacement of a leaving group at *C*-1′ exocyclic methyl group of the pentofuranose ring; and (ii) *de novo* construction of the nucleobase by condensation reactions from methylamino derivatives at *C*-1′.^4^

**Fig. 1:**
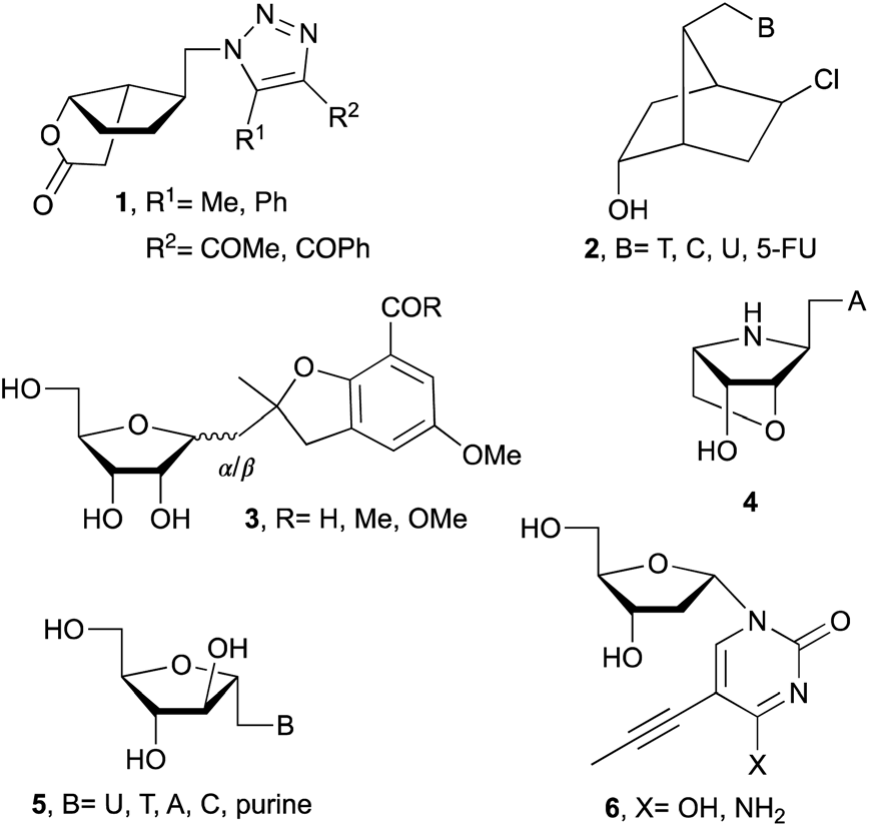
Structures of 1′-homo-*N*-nucleosides and/or α-nucleosides.

Following these routes, 1′-homo-*N*-bicyclic carbonucleosides 1 (Fig. 1),^5^ 1′-homo-*N*-bicyclico-[2.2.1]heptane nucleosides 2,^6^ emissive 1′-homo-*N*-nucleosides 3,^7^ and 1′-homo-*N*-locked nucleoside 4^8^ were synthesized. Also, α-nucleosides analogs such as arabinonucleosides 5 or 2′-deoxy-α-nucleosides 6 are easily formed due to reduced steric hindrance compared with β-ribonucleosides.^2,9^ Additionally, α-nucleosides are used to prepare α-oligonucleotides which leads to more stable duplexes.^10^

Related with the research described in this manuscript, β-ribo^11^ and α-ribo^12^ 1′-homo-*N*-nucleosides (7 and 8, respectively) have been prepared with variable success due to the use of multistep process and undesired side reactions. Whereas, fewer examples are available related to the synthesis of 1′-homo-*N*-2′-deoxynucleosides. For example, β-deoxy derivatives (9) were described by Saladino and Crucianelli,^13*a*^ Doboszewski,^13*b*,*c*^ and Herdewijn.^13*d*^ In relation to the closest work described here, that is, the synthesis of α-deoxy 1′-homo-*N*-2′-nucleosides only Beaucage and co-workers^14^ have reported the synthesis with one nucleobase, thymine (10c).

Our pursuit for the design of novel nucleosides and the development of their synthesis protocols,^8,15^ here we wish to report the synthesis of 1′-homo-*N*-2′-deoxy-α-nucleosides 10 with both purine and pyrimidine nucleobases. Installation of bioactive 5-fluoro and 5-iodo uracil derivatives is also demonstrated to access new structures and test their biological activities. Eight modified nucleosides reported herein were tested for their biological activity as antiviral against HIV-1 and HBV. Their cytotoxicity assay is also included in this study.

## 2. Results and discussion

### Synthesis of 1′-homo-*N*-2′-deoxy-α-nucleosides

**Fig. 2:**
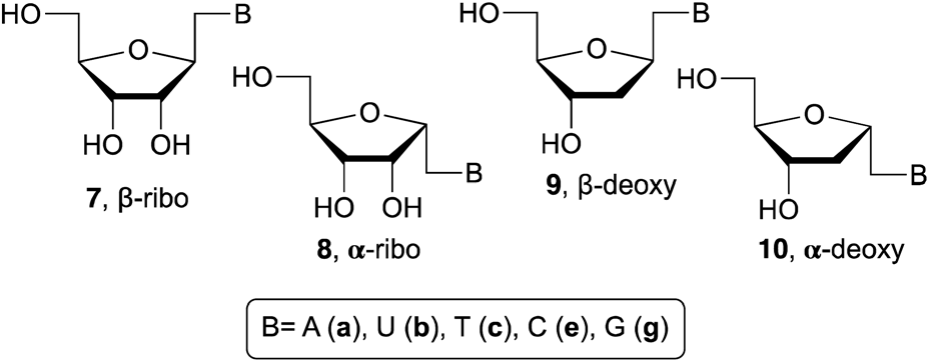
Structures of α/β-ribo and α/β-2′-deoxy 1′-homo-*N*-nucleosides.

**Fig. 3:**
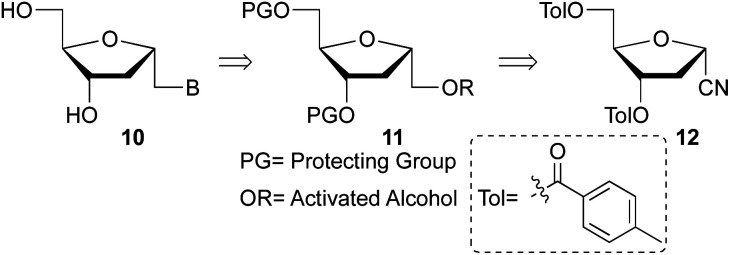
Retrosynthetic analysis to 1′-homo-*N*-2′-deoxy-α-nucleosides.

Beaucage and coworkers reported on the synthesis of 1′-homo-α-thymidine where pyrimidine ring was constructed *via* an acyclic analog.^14^ This approach is not practical for the synthesis of multiple analogs containing both natural and unnatural nucleobases. Therefore, we wish to develop a new synthetic route which permits the synthesis of any 1′-homo-α-nucleoside analog from a single precursor such as sugar derivative 11. To prepare the 1′-homo-*N*-2′-deoxy-α-nucleoside derivatives 10, we envisioned a synthetic approach wherein the nucleobase was introduced by nucleophilic displacement of a leaving group in sugar precursor 11, readily obtained from the α-cyano sugar derivative 12 (Fig. 2 and 3).^16^

**Scheme 1:**
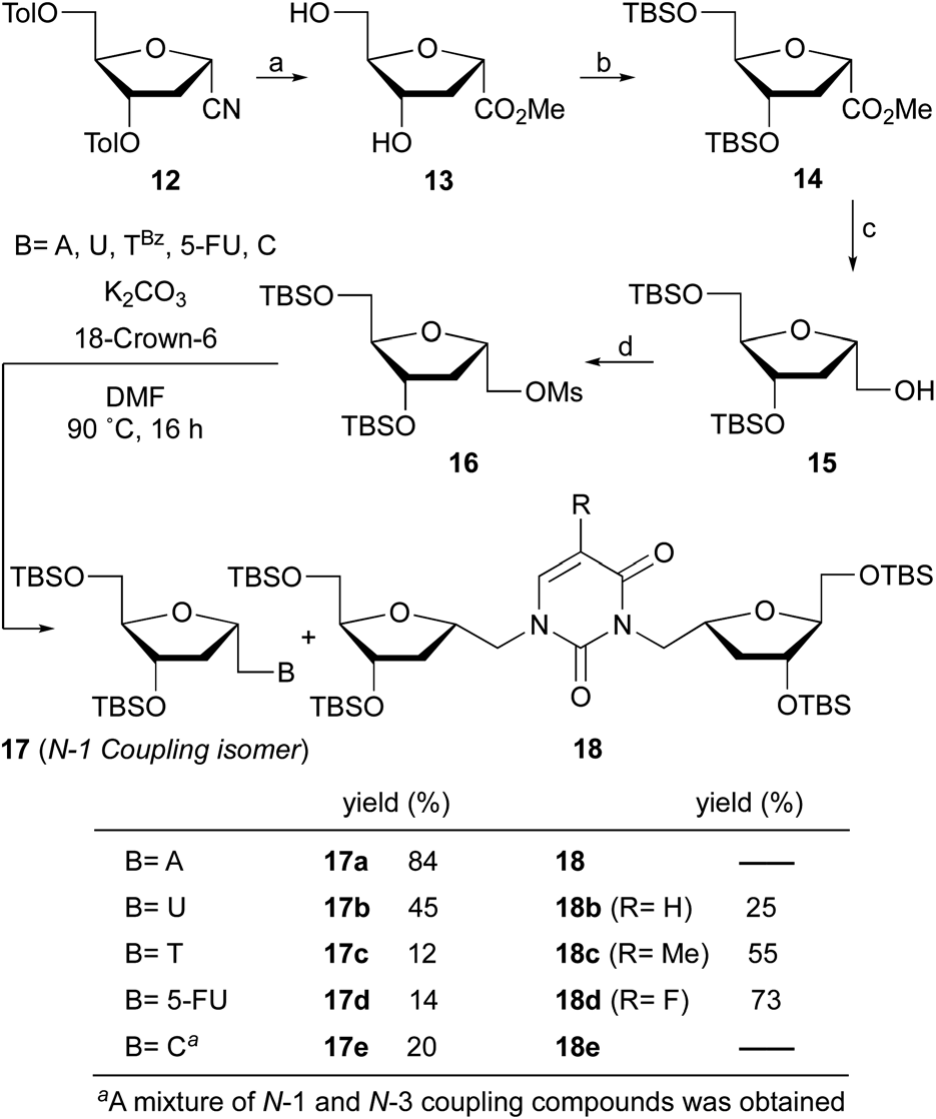
Reaction conditions: (a) KOH, MeOH/H_2_O, rt, 3 h (75%); (b) TBSCl, imidazole, CH_2_Cl_2_, 50 °C, 5 h (85%); (c) LiAlH_4_, THF, −45 °C, 30 min (90%); (d) MsCl, DMAP, Py, 0 °C to rt, 24 h (95%).

Therefore, nitrile 12 was converted into the corresponding methyl ester by treatment with potassium hydroxide in MeOH/H_2_O. Under these conditions, removal of the toluoyl protecting groups is also observed, affording compound 13 in 75% isolated yield after chromatography (Scheme 1). One-pot deprotection of toluoyl groups, hydrolysis of nitrile and *in situ* esterification demonstrates a highly efficient protocol compared to the literature report for the corresponding β-anomer.^16*b*^

Protection of the hydroxyl groups with *tert*-butyldimethylsilyl chloride (TBSCl) in the presence of imidazole gave silyl ether derivative 14. The reduction of ester 14 with lithium aluminum hydride in THF at −45 °C afforded after 30 min alcohol 15 in 90% yield. It is noteworthy that higher reaction temperatures during reduction leads to deprotection of the silyl groups and low temperature is critical to the successful outcome. Transformation of the alcohol into the mesylate 16 was achieved in excellent yield with methanesulfonyl chloride and catalytic DMAP in pyridine.

With mesylate 16 in hand, the stage was set for rapid synthesis of α-homonucleosides 10. Next, we opted for the base mediated displacement reaction using K_2_CO_3_ and 18-crown-6 in DMF. To our delight, the coupling of unprotected adenine base with mesylate 16 under these conditions at 90 °C furnished 1′-homo-*N*-2′-deoxy-α-adenosine (17a) in 84% yield after column chromatography purification. With the guanine base, the reaction of mesylate 16 under similar conditions furnished a complex mixture of products.

Under identical reaction conditions, uracil, a pyrimidine base when coupled with mesylate 16 furnished a mixture of two products. The expected 1′-homo-*N*-2′-deoxy-α-uridine (17b) was isolated in 45% yield. The second product was characterized as a dimer 18b, in which the uracil is linked with two deoxyribose moieties at the *N*-1 and *N*-3 positions of the base. The structure of dimer 18b was supported by spectral data.

Interestingly, the coupling of thymine base with mesylate 16 furnished two products where desired homonucleoside 17c was isolated in 12% yield and dimer 18c in 55% yield (Scheme 1). Similarly, the coupling of 5-fluorouracil base with mesylate 16 resulted in the formation of dimer 18d in 73% yield as a major product along with 17d in 14% yield. This observation is unprecedented where a dimer is obtained as a major product and offers a new synthetic pathway to the assembly of unique nucleoside scaffolds.

**Fig. 4:**
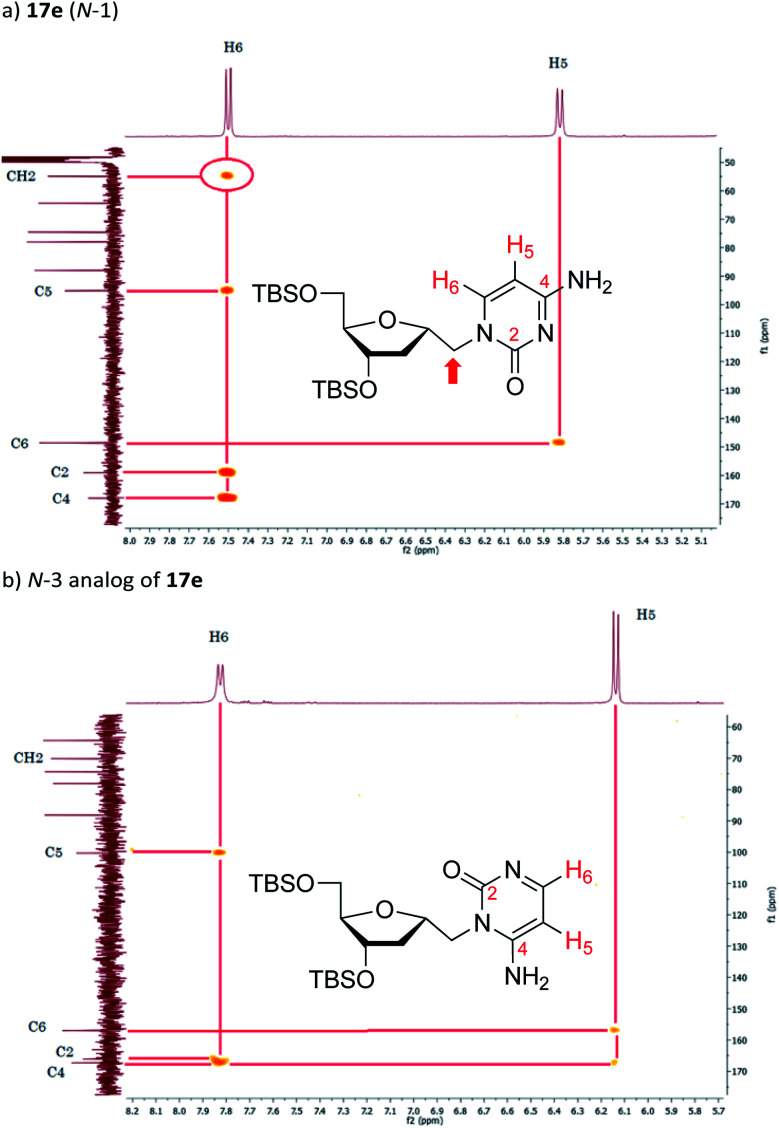
HMBC spectra enlargements of: (a) 17e and (b) its *N*-3 analog.

The synthesis of 1′-homo-*N*-2′-deoxy-α-cytidine was also complicated due to the formation of two products. Reaction of 16 with cytosine gave a mixture of *N*-1 (compound 17e) and *N*-3 (see Fig. 4b) coupled nucleosides. The structure of these products was established by homonuclear (COSY) and heteronuclear (HSQC, HMBC) correlations. In the ^13^C–^1^H HMBC experiment for 17e, a correlation cross-peak was observed between the H6 proton of the base and the methylene carbon at 6′-position. However, in the *N*-3 derivative this cross-peak does not exist.

**Scheme 2:**
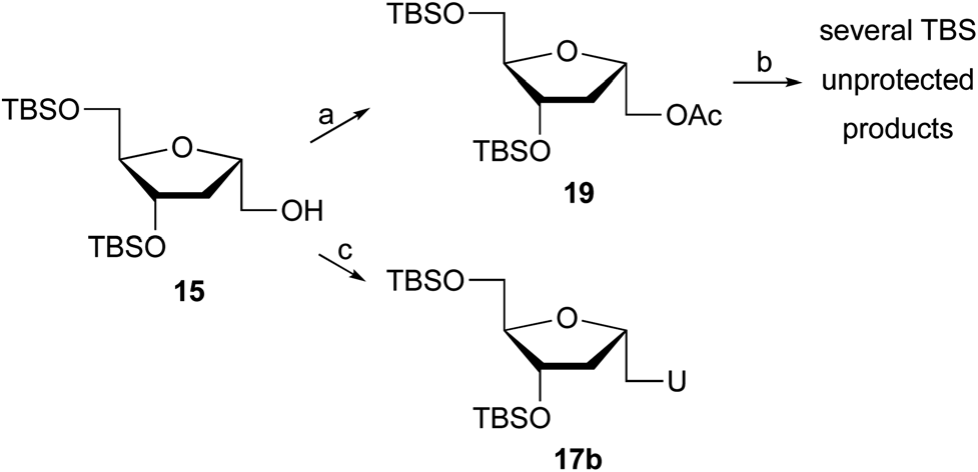
Reaction conditions: (a) Ac_2_O, Et_3_N, DMAP, CH_2_Cl_2_, rt, 2 h (90%); (b) (i) Base (U, 5-FU, 5-IU), BSA, MeCN, 80 °C, 1 h, (ii) TMSOTf, 0 °C to 80 °C; (c) Ph_3_P, DIAD, uracil, THF, −10 °C to 25 °C, 24 h (15%).

In an attempt to improve the yield of the desired pyrimidine coupled products, other strategies were tested. Glycosylation under Vorbrüggen's conditions^17^ required acetate 19 (Scheme 2), which was easily prepared from 15 by treatment with acetic anhydride, Et_3_N, and DMAP in CH_2_Cl_2_. Reaction of 19 with silylated bases such as uracil or 5-fluorouracil or 5-iodouracil in the presence of trimethylsilyl triflate failed to provide the coupled product, while deprotection of the TBS groups was observed.

The Mitsunobu reaction is an important technique in nucleoside synthesis for the direct coupling of alcohols with bases under mild conditions. Thus, treatment of 15 with uracil, Ph_3_P and DIAD generated 1′-homo-*N*-2′-deoxy-α-uridine (17b), but with only 15% yield. The low yield is due, in part, to the difficulty of isolating the product during chromatography, since several byproducts are obtained post Mitsunobu reaction.

Since alternative coupling protocol failed to improve the outcome of the reaction and the formation of desired product, we revisited the first protocol offering a viable option. This time, we replaced the mesylate with tosylate hoping that we might observe better coupling efficiency and overall yield.

**Scheme 3:**
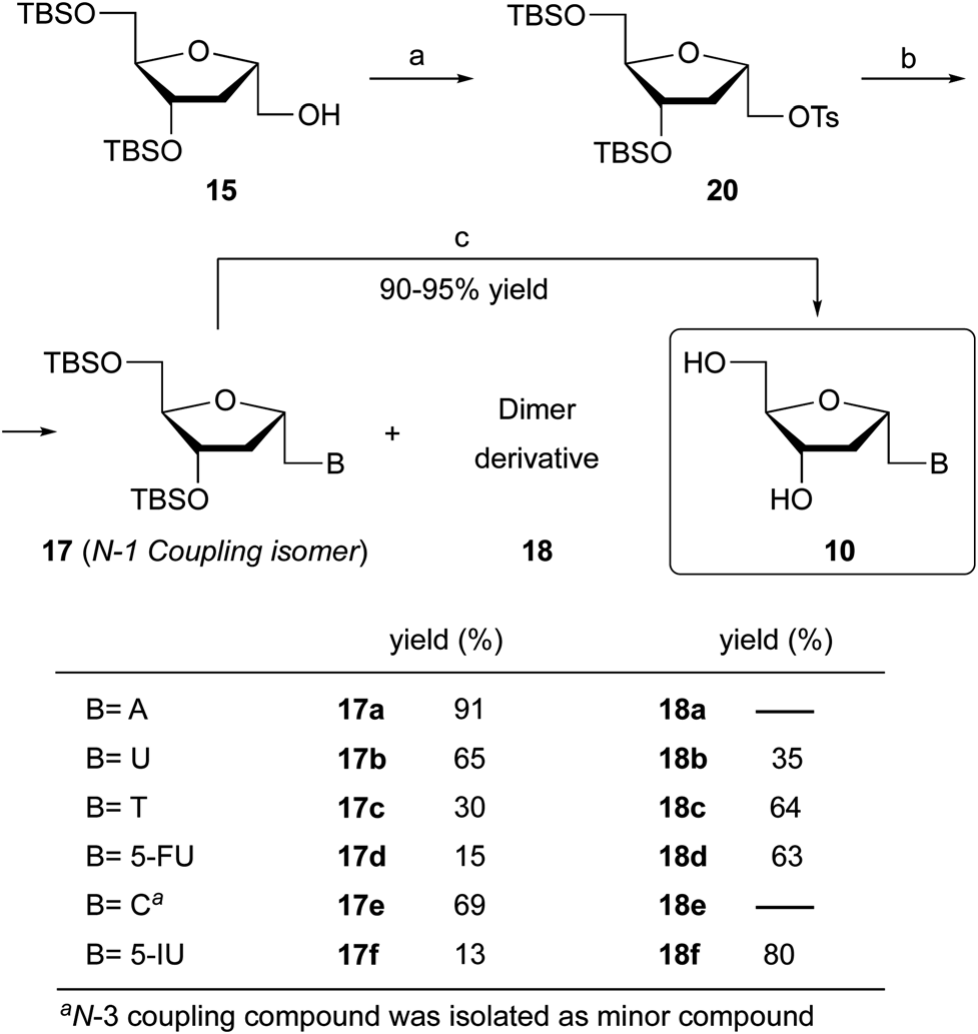
Reaction conditions: (a) TsCl, DMAP, Py, 0 °C to rt, 9 h (90%); (b) B = A, U, T^Bz^, 5-FU, C, 5-IU, K_2_CO_3_, 18-crown-6, DMF, 90 °C, 7–8 h; (c) TBAF, THF, 25 °C, 2 h (90–95%).

The synthesis of tosylate 20 (Scheme 3) was straightforward by the reaction of alcohol 15 with *p*-toluensulfonyl chloride (TsCl) and catalytic DMAP in pyridine. Next, we performed the coupling reaction of 20 with the corresponding nucleobase in the presence of K_2_CO_3_ and 18-crown-6. The reaction of 20 with adenine proceeds with excellent yield, giving rise to α-homonucleoside 17a with a 91% isolated yield, slightly higher than when the reaction was carried out with mesylated 16. Significant improvement was observed when 20 reacted with uracil or cytosine, isolating 1′-homo-*N*-2′-deoxy-α-uridine (17b) and 1′-homo-*N*-2′-deoxy-α-cytidine (17e) with 65% and 69% yield, respectively, *versus* 45% and 20% yield obtained from the mesylate derivative. However, the coupling of the tosylated derivative 20 with thymine led exclusively to the dimer 18c. To the best of our knowledge, this is first report of dimer 18c synthesis in high yield. Next, in order to control the regioselectivity of the coupling with thymine, the *N*-3-position of the base was protected. The reaction was carried out according to the procedure described by Ludek and Meier.^18^ For that, thymine was allowed to react with benzoyl chloride in acetonitrile-pyridine to give *N*^3^-benzoylthymine. Reaction of 20 and *N*^3^-benzoylthymine under the reaction conditions indicated above afforded 1′-homo-*N*-2′-deoxy-α-thymidine (17c) in 30% yield, although the major product of the reaction was still the dimer 18c, probably due to the *in situ* deprotection of the base during the coupling. Nevertheless, the formation of the desired product 17c has been considerably improved when *N*^3^ protected thymine was utilized.

The coupling of 20 and 5-fluorouracil or 5-iodouracil was performed next. The major product was the dimer derivative but the *N*-1 coupled analog was also formed in 15% and 13% yield, respectively. In both cases, the *N*-3 protection of the base did not improve the yield of the *N*-1 substituted product. Multiple products were observed when 20 was coupled with the guanine base.

**Fig. 5:**
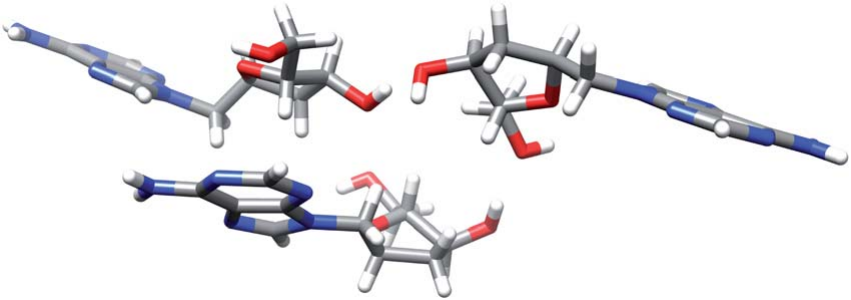
X-ray structure of 1′-homo-*N*-2′-deoxy-α-adenosine (10a). The structure shows three molecules per asymmetric unit.

Finally, the treatment of compounds 17a–f with tetrabutylammonium fluoride (TBAF) in THF afforded the 1′-homo-*N*-2′-deoxy-α-nucleosides 10a–f in 90–95% yield. The structure of all derivatives was established by NMR homonuclear (COSY) and heteronuclear (HSQC, HMBC) correlations. Unambiguous confirmation of the structure of the synthesized compounds was obtained by a single crystal X-ray analysis of 10a (Fig. 5).

Single crystal X-ray data for compound 10a is given in Table 1. Homo-*N*-2′-deoxynucleoside analog 10a crystallized as colourless small crystals. Fig. 5 is a Chimera drawing,^19^ which corresponds to asymmetric unit X-ray diffraction studies of compound 10a.^20^ Note that the sugar conformation is North type with the 2′-*exo* and 3′-*endo* configurations. The structure shows three molecules per asymmetric unit linked by H-bonds, that is, six molecules per crystal.

**Table 1:** Crystal data for homo-*N*-nucleoside analog 10a

Formula	C_11_H_15_N_5_O_3_
Molecular weight	265.28
Crystal system	Monoclinic
Space group	*P*2_1_
*a*, Å	15.8711 (6)
*b*, Å	8.04111 (18)
*c*, Å	16.4342 (5)
*Z*	6
Final *R*	0.035
Reflections	4865

**Scheme 4:**
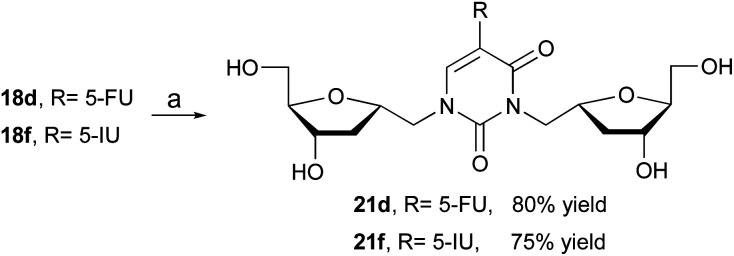
Reaction conditions: (a) TBAF, THF, 25 °C, 2 h.

The formation of various dimers 18 is synthetically important because it offers an access to the assembly of bifunctional 2′-deoxynucleoside analogs. Literature search for reports on similar structures has been limited to two examples. First report by Prystas and Sorm^21^ describing the formation of *N*^1^,*N*^3^-diribofuranosyl derivative as a byproduct during ribosylation of 6-methyl-4-methoxy-2(1*H*)-pyrimidinone with a ribofuranosyl chloride derivative. In the second example, Zhang and co-workers^22^ reported the isolation of a similar derivative in the synthesis of isonucleosides. In order to further investigate the importance of this bifunctional nucleoside, we have evaluated the biological activity of dimeric compounds 21 (Scheme 4). For that, desilylation of 18d and 18f with TBAF in THF furnished the desired derivatives 21d and 21f in 80% and 75% yield, respectively.

### Biological evaluation

#### Antiviral assays against HIV-1^23^

A set of eight compounds 10a–f and 21d,f were tested against HIV-1 (strain LAI) and compared to 3′-azido-3′-deoxythymidine (AZT, zidovudine) as a control in an assay with human peripheral blood mononuclear (PBM) cells. Positive control AZT exhibited expected anti-HIV-1 activity, with EC_50_ of 0.006 μM. There was no anti-HIV activity observed from the six homonucleosides 10a–f and the two dimeric compounds 21d,f tested, all with an EC_50_ > 10 μM.

#### Antiviral assays against HBV^23*c*,24^

As expected, the control Lamivudine demonstrates desired antiviral response, with an EC_50_ value < 10 μM (97% HBV DNA inhibition at 10 μM). Among eight derivatives, none displayed anti-HBV activity, with EC_50_ values > 10 μM.

#### Cytotoxicity profile^23*c*,25^

These assays were performed in human PBM, T lymphoblast CEM-CCRF (CEM), African green monkey kidney (Vero) or human liver (HepG2) cells. Results represent means from triplicate wells. Positive control cycloheximide exhibited expected toxicity in the PBM, CEM, Vero, and HepG2 cells, with an IC_50_ (μM) of 0.6, 0.08, 0.5, and 0.8, respectively (data not shown). None of the eight compounds exhibited apparent cytotoxicity (IC_50_ values > 100 μM) in four cell types tested.

## 3. Experimental

### General

TLC plates were visualized with 2.5% *p*-anisaldehyde, 3.5% sulfuric acid, and 1% acetic acid in ethanol. Pure α-cyano-sugar 12 is commercially available^16*a*^ and T^Bz^ was prepared as previously reported.^18^ Unless otherwise specified column chromatography was performed over silica 60 Å (230–400 mesh).

### General procedure for desilylation of 17. Synthesis of 10

TBAF (6 eq., 1.0 M in THF) was added dropwise to a stirred solution of 17 (1 eq.) in anhydrous THF (0.1 M) at 0 °C. After 5 min, the ice bath was removed, and the reaction mixture was stirred at room temperature for 2 h. Next, solvents were evaporated, and the residue was purified by column chromatography (gradient eluent 15–20% MeOH/CH_2_Cl_2_ for 10a–c, 10e; gradient eluent 5–20% MeOH/CH_2_Cl_2_ for 10d and 10f) to afford 10a–f in 90–95% yield.

#### 1′-Homo-*N*-2′-deoxy-α-adenosine (10a)

95% yield. *R*_f_: 0.18 (20% MeOH/CH_2_Cl_2_); ^1^H NMR (300.13 MHz, MeOH-*d*_4_): *δ* 1.69 (dt, 1H, H_2′_, *J* = 13.1, 5.8 Hz), 2.38 (dt, 1H, H_2′_, *J* = 13.3, 6.7 Hz), 3.50 (m, 1H, H_5′_), 3.59 (dd, 1H, H_5′_, *J* = 11.8, 3.9 Hz), 3.85 (q, 1H, H_4′_, *J* = 4.5 Hz), 4.25 (dt, 1H, H_3′_, *J* = 6.6, 5.2 Hz), 4.38 (m, 2H, H_6′_), 4.45 (m, 1H, H_1′_), 8.16 (s, 1H, H_2_) 8.20 (s, 1H, H_8_) ppm; ^13^C NMR (75.5 MHz, MeOH-*d*_4_): *δ* 38.5 (C_2′_), 48.8 (C_6′_), 63.3 (C_5′_), 73.2 (C_3′_), 77.8 (C_1′_), 87.6 (C_4′_), 119.7 (C_4_), 143.6 (C_2_), 150.8 (C_5_), 153.6 (C_8_), 157.2 (C_6_) ppm; HRMS (ESI^+^, *m*/*z*): calcd for C_11_H_16_N_5_O_3_ [M + H]^+^: 266.1248, found: 266.1251.

#### 1′-Homo-*N*-2′-deoxy-α-uridine (10b)

95% yield. *R*_f_: 0.13 (10% MeOH/CH_2_Cl_2_); ^1^H NMR (300.13 MHz, MeOH-*d*_4_): *δ* 1.70 (ddd, 1H, H_2′_, *J* = 13.1, 5.9, 4.9 Hz), 2.33 (m, 1H, H_2′_), 3.53 (m, 2H, H_5′_), 3.87 (m, 2H, H_4′_ + H_6′_), 3.96 (dd, 1H, H_6′_, *J* = 14.1, 3.4 Hz), 4.25 (dt, 1H, H_3′_, *J* = 6.6, 4.6 Hz), 4.34 (m, 1H, H_1′_), 5.63 (d, 1H, H_5_, *J* = 7.9 Hz), 7.60 (d, 1H, H_6_, *J* = 7.9 Hz) ppm; ^13^C NMR (75.5 MHz, MeOH-*d*_4_): *δ* 38.4 (C_2′_), 53.2 (C_6′_), 63.3 (C_5′_), 73.3 (C_3′_), 77.7 (C_1′_), 87.6 (C_4′_), 101.6 (C_5_), 148.5 (C_6_), 153.0 (C_2_), 166.9 (C_4_) ppm; HRMS (ESI^+^, *m*/*z*): calcd for C_10_H_14_N_2_NaO_5_ [M + Na]^+^: 265.0795, found: 265.0797.

#### 1′-Homo-*N*-α-thymidine (10c)^14^

95% yield. *R*_f_: 0.40 (15% MeOH/CH_2_Cl_2_); ^1^H NMR (300.13 MHz, MeOH-*d*_4_): *δ* 1.69 (m, 1H, H_2′_), 1.87 (d, 3H, *Me*–H_5_, *J* = 1.1 Hz), 2.33 (dt, 1H, H_2′_, *J* = 13.9, 7.1 Hz), 3.55 (qd, 2H, H_5′_, *J* = 11.8, 4.6 Hz), 3.87 (m, 3H, H_4′_ + 2H_6′_), 4.24 (dt, 1H, H_3′_, *J* = 6.6, 4.7 Hz), 4.34 (m, 1H, H_1′_), 7.44 (s, 1H, H_6_, *J* = 1.2 Hz) ppm; ^13^C NMR (75.5 MHz, MeOH-*d*_4_): *δ* 12.2 (*C*H_3_), 38.5 (C_2′_), 53.1 (C_6′_), 63.4 (C_5′_), 73.3 (C_3′_), 77.7 (C_1′_), 87.6 (C_4′_), 110.4 (C_5_), 144.3 (C_6_), 153.1 (C_2_), 167.0 (C_4_) ppm; HRMS (ESI^+^, *m*/*z*): calcd for C_11_H_17_N_2_O_5_ [M + H]^+^: 257.1132, found: 257.1134.

#### 1′-Homo-*N*-2′-deoxy-α-5-fluorouridine (10d)

90% yield. *R*_f_: 0.29 (15% MeOH/CH_2_Cl_2_); ^1^H NMR (300.13 MHz, MeOH-*d*_4_): *δ* 1.70 (ddd, 1H, H_2′_, *J* = 13.2, 5.9, 4.8 Hz), 2.34 (dt, 1H, H_2′_, *J* = 12.0, 6.0 Hz), 3.55 (m, 2H, H_5′_), 3.88 (m, 3H, H_4′_ + 2H_6′_), 4.24 (m, 1H, H_3′_), 4.35 (m, 1H, H_1′_), 7.81 (d, 1H, H_6_, *J* = 6.4 Hz) ppm; ^13^C NMR (75.5 MHz, MeOH-*d*_4_): *δ* 38.4 (C_2′_), 53.2 (C_6′_), 63.3 (C_5′_), 73.3 (C_3′_), 77.7 (C_1′_), 87.6 (C_4′_), 132.4 (d, C_6_, *J* = 33.5 Hz), 141.3 (d, C_5_, *J* = 231.1 Hz), 151.7 (C_2_), 159.9 (d, C_4_, *J* = 25.7 Hz) ppm; HRMS (ESI^+^, *m*/*z*): calcd for C_10_H_14_FN_2_O_5_ [M + H]^+^: 261.0881, found: 261.0874 and calcd for C_10_H_13_FN_2_NaO_5_ [M + Na]^+^: 283.0701, found: 283.0695.

#### 1′-Homo-*N*-2′-deoxy-α-cytidine (10e)

95% yield. *R*_f_: 0.07 (15% MeOH/CH_2_Cl_2_); ^1^H NMR (300.13 MHz, MeOH-*d*_4_): *δ* 1.68 (ddd, 1H, H_2′_, *J* = 13.1, 6.0, 5.2 Hz), 2.33 (dt, 1H, H_2′_, *J* = 13.8, 7.0 Hz), 3.50 (m, 1H, H_5′_), 3.58 (dd, 1H, H_5′_, *J* = 11.8, 4.0 Hz), 3.80 (m, 1H, H_6′_), 3.84 (m, 1H, H_4′_), 4.04 (dd, 1H, H_6′_, *J* = 13.8, 3.1 Hz), 4.24 (dt, 1H, H_3′_, *J* = 6.6, 4.9 Hz), 4.36 (m, 1H, H_1′_), 5.82 (d, 1H, H_5_, *J* = 7.2 Hz), 7.58 (d, 1H, H_6_, *J* = 7.3 Hz) ppm; ^13^C NMR (75.5 MHz, MeOH-*d*_4_): *δ* 38.6 (C_2′_), 54.7 (C_6′_), 63.4 (C_5′_), 73.3 (C_3′_), 77.6 (C_1′_), 87.4 (C_4′_), 95.2 (C_5_), 148.6 (C_6_), 159.1 (C_2_), 168.0 (C_4_) ppm; HRMS (ESI^+^, *m*/*z*): calcd for C_10_H_16_N_3_O_4_ [M + H]^+^: 242.1135, found: 242.1138.

#### 1′-Homo-*N*-2′-deoxy-α-5-iodouridine (10f)

92% yield. *R*_f_: 0.47 (15% MeOH/CH_2_Cl_2_); ^1^H NMR (300.13 MHz, MeOH-*d*_4_): *δ* 1.69 (dt, 1H, H_2′_, *J* = 13.1, 5.6 Hz), 2.33 (dt, 1H, H_2′_, *J* = 13.9, 7.3 Hz), 3.54 (m, 2H, H_5′_), 3.89 (m, 3H, H_4′_ + 2H_6′_), 4.25 (m, 1H, H_3′_), 4.33 (m, 1H, H_1′_), 8.05 (s, 1H, H_6_) ppm; HRMS (ESI^+^, *m*/*z*): calcd for C_10_H_14_IN_2_O_5_ [M + H]^+^: 368.9942, found: 368.9943 and calcd for C_10_H_13_IN_2_NaO_5_ [M + Na]^+^: 390.9761, found: 390.9764.

### 1,2-Dideoxy-1α-(methoxycarbonyl)-d-ribofuranose (13)

To a solution of 12 (500 mg, 1.3 mmol) in MeOH (15 mL) at room temperature were added 3.1 mL of a 2.5 M KOH solution (25% MeOH : H_2_O). The progress of the reaction was monitored by TLC (10% MeOH/CH_2_Cl_2_) until no further reaction was apparent (3 h). Next, the mixture was neutralized with DOWEX 50WX8 hydrogen form (50–100 mesh). After removal of the resin by filtration, the solution was evaporated, and the residue was purified by column chromatography (5% MeOH/CH_2_Cl_2_) to afford 13 as a yellowish oil in 75% yield. *R*_f_: 0.22 (5% MeOH/CH_2_Cl_2_); ^1^H NMR (300.13 MHz, MeOH-*d*_4_): *δ* 2.11 (dt, 1H, H_2_, *J* = 13.2, 3.9 Hz), 2.47 (ddd, 1H, H_2_, *J* = 13.2, 8.8, 6.2 Hz), 3.59 (m, 2H, H_5_), 3.73 (s, 3H, *Me*), 3.99 (q, 1H, H_4_, *J* = 4.2 Hz), 4.23 (dt, 1H, H_3_, *J* = 6.3, 3.6 Hz), 4.60 (dd, 1H, H_1_, *J* = 8.8, 4.2 Hz) ppm; ^13^C NMR (75.5 MHz, MeOH-*d*_4_): *δ* 39.7 (C_2_), 52.5 (Me), 63.1 (C_5_), 72.7 (C_3_), 77.3 (C_1_), 88.6 (C_4_), 175.6 (*C*=O) ppm; HRMS (ESI^+^, *m*/*z*): calcd for C_7_H_12_NaO_5_ [M + Na]^+^: 199.0577, found: 199.0578.

### 3,5-Bis-*O*-(*tert*-butyldimethylsilyl)-1,2-dideoxy-1α-(methoxycarbonyl)-d-ribofuranose (14)

TBSCl (904.3 mg, 6 mmol) was added to a solution of 13 (210 mg, 1.2 mmol) and imidazole (408.5 mg, 6 mmol) in anhydrous CH_2_Cl_2_ (4 mL) at room temperature. The reaction mixture was stirred at 50 °C for 5 h, then diluted with water/ice, and extracted with CH_2_Cl_2_. The organic layers were dried (Na_2_SO_4_) and evaporated in a vacuum. The residue was purified by column chromatography (10% EtOAc/hexane) to yield 14 as a viscous liquid in 85% yield. *R*_f_: 0.65 (20% EtOAc/hexane); ^1^H NMR (300.13 MHz, MeOH-*d*_4_): *δ* 0.088 (s, 3H, Si–*Me*), 0.095 (s, 3H, Si–*Me*), 0.098 (s, 3H, Si–*Me*), 0.102 (s, 3H, Si–*Me*), 0.87 (s, 9H, Si–^*t*^*Bu*), 0.92 (s, 9H, Si–^*t*^*Bu*), 2.16 (dt, 1H, H_2_, *J* = 13.0, 2.4 Hz), 2.39 (ddd, 1H, H_2_, *J* = 13.0, 8.9, 5.2 Hz), 3.53 (dd, 1H, H_5_, *J* = 11.0, 5.8 Hz), 3.67 (dd, 1H, H_5_, *J* = 11.0, 3.8 Hz), 3.71 (s, 3H, O–*Me*), 4.01 (ddd, 1H, H_4_, *J* = 5.7, 3.7, 2.0 Hz), 4.36 (dt, 1H, H_3_, *J* = 5.2, 2.1 Hz), 4.59 (dd, 1H, H_1_, *J* = 8.9, 2.7 Hz) ppm; ^13^C NMR (75.5 MHz, MeOH-*d*_4_): *δ* −5.3 (Si–*C*H_3_), −5.2 (Si–*C*H_3_), −4.7 (Si–*C*H_3_), −4.6 (Si–*C*H_3_), 18.7 (Si*C*Me_3_), 19.2 (Si*C*Me_3_), 26.2 (3*C*H_3_–^*t*^Bu), 26.4 (3*C*H_3_–^*t*^Bu), 39.9 (C_2_), 52.5 (Me), 64.3 (C_5_), 74.2 (C_3_), 77.7 (C_1_), 89.5 (C_4_), 175.4 (*C*=O) ppm; HRMS (ESI^+^, *m*/*z*): calcd for C_19_H_40_NaO_5_Si_2_ [M + Na]^+^: 427.2306, found: 427.2302.

### 3,5-Bis-*O*-(*tert*-butyldimethylsilyl)-1,2-dideoxy-1α-(hydroxymethyl)-d-ribofuranose (15)

LiAlH_4_ (44 mg, 1.16 mmol) was added to a solution of 14 (360 mg, 0.89 mmol) in anhydrous THF (6 mL) at −45 °C, and the reaction was stirred for 30 min at the same temperature. Next, the mixture was diluted with THF and MeOH, the residue was filtered on Celite and concentrated to afford 15 as a viscous liquid (90% yield), which was sufficiently pure for the next step. *R*_f_: 0.34 (20% EtOAc/hexane); ^1^H NMR (300.13 MHz, MeOH-*d*_4_): *δ* 0.082 (s, 3H, Si–*Me*), 0.084 (s, 3H, Si–*Me*), 0.10 (s, 6H, Si–*Me*), 0.91 (s, 9H, Si–^*t*^*Bu*), 0.92 (s, 9H, Si–^*t*^*Bu*), 1.73 (ddd, 1H, H_2_, *J* = 12.8, 5.9, 4.8 Hz), 2.24 (ddd, 1H, H_2_, *J* = 12.9, 7.5, 6.5 Hz), 3.54 (dd, 1H, H_6_, *J* = 11.4, 4.6 Hz), 3.62 (m, 3H, 2H_5_ + H_6_), 3.81 (q, 1H, H_4_, *J* = 4.1 Hz), 4.13 (m, 1H, H_1_), 4.40 (ddd, 1H, H_3_, *J* = 6.4, 4.7, 3.9 Hz) ppm; ^13^C NMR (75.5 MHz, MeOH-*d*_4_): *δ* −5.3 (Si–*C*H_3_), −5.2 (Si–*C*H_3_), −4.6 (Si–*C*H_3_), −4.5 (Si–*C*H_3_), 18.8 (Si*C*Me_3_), 19.2 (Si*C*Me_3_), 26.3 (3*C*H_3_–^*t*^Bu), 26.4 (3*C*H_3_–^*t*^Bu), 37.9 (C_2_), 64.4 (C_5_), 66.0 (C_6_), 74.4 (C_3_), 80.9 (C_1_), 87.9 (C_4_) ppm; HRMS (ESI^+^, *m*/*z*): calcd for C_18_H_40_NaO_4_Si_2_ [M + Na]^+^: 399.2357, found: 399.2358.

### 3,5-Bis-*O*-(*tert*-butyldimethylsilyl)-1,2-dideoxy-1α-(((methylsulfonyl)oxy)methyl)-d-ribofuranose (16)

To a solution of 15 (135 mg, 0.36 mmol) in anhydrous pyridine (0.65 mL) at 0 °C were added MsCl (62 μL, 0.8 mmol) and catalytic DMAP (5 mg). The solution was stirred at room temperature for 24 h. Then, the solvent was removed in a vacuum, and the residue was dissolved in water and extracted with Et_2_O. The organic layers were dried (Na_2_SO_4_) and evaporated in a vacuum. The residue was purified by column chromatography (20% EtOAc/hexane) to yield 16 as a viscous liquid in 95% yield. *R*_f_: 0.42 (20% EtOAc/hexane); ^1^H NMR (300.13 MHz, MeOH-*d*_4_): *δ* 0.08 (s, 6H, Si–*Me*), 0.11 (s, 6H, Si–*Me*), 0.92 (s, 18H, Si–^*t*^*Bu*), 1.75 (dt, 1H, H_2_, *J* = 13.0, 3.9 Hz), 2.32 (m, 1H, H_2_), 3.09 (s, 3H, Ms–*Me*), 3.61 (dq, 2H, H_5_, *J* = 11.0, 4.6 Hz), 3.89 (q, 1H, H_4_, *J* = 4.3 Hz), 4.20 (m, 1H, H_6_), 4.39 (m, 3H, H_1_ + H_3_ + H_6_) ppm; ^13^C NMR (75.5 MHz, MeOH-*d*_4_): *δ* −5.3 (Si–*C*H_3_), −5.2 (Si–*C*H_3_), −4.62 (Si–*C*H_3_), −4.57 (Si–*C*H_3_), 18.8 (Si*C*Me_3_), 19.2 (Si*C*Me_3_), 26.3 (3*C*H_3_–^*t*^Bu), 26.4 (3*C*H_3_–^*t*^Bu), 37.5 (Ms–*Me*), 37.6 (C_2_), 64.4 (C_5_), 73.4 (C_6_), 74.4 (C_3_), 78.0 (C_1_), 88.6 (C_4_) ppm; HRMS (ESI^+^, *m*/*z*): calcd for C_19_H_42_NaO_6_SSi_2_ [M + Na]^+^: 477.2133, found: 477.2134.

### General procedure for coupling of 20 with nucleobases. Synthesis of 17 and 18

To a solution of 20 in anhydrous DMF (0.1 M) was added K_2_CO_3_ (1.5 eq. for A, C, 5-FU, and 5-IU; 1.2 eq. for U; 2.1 eq. for T^Bz^), 18-crown-6 (1.5 eq.), and the nucleobase (1.2 eq. for A, U, C, and T^Bz^; 1.5 eq. for 5-FU and 5-IU). The mixture was stirred at 90 °C and the reaction progress followed by TLC (5% MeOH/CH_2_Cl_2_) until no further reaction was apparent (7–8 h). Solvents were evaporated and the residue was purified by column chromatography (5% MeOH/CH_2_Cl_2_) to yield 17 (or 18). Yields are indicated in Scheme 3.

#### 3,5-Bis-*O*-(*tert*-butyldimethylsilyl)-1′-homo-*N*-2′-deoxy-α-adenosine (17a)

White solid. mp 137–138 °C; *R*_f_: 0.32 (5% MeOH/CH_2_Cl_2_); ^1^H NMR (300.13 MHz, MeOH-*d*_4_): *δ* −0.02 (s, 3H, Si–*Me*), 0.01 (s, 3H, Si–*Me*), 0.06 (s, 3H, Si–*Me*), 0.08 (s, 3H, Si–*Me*), 0.86 (s, 9H, Si–^*t*^*Bu*), 0.87 (s, 9H, Si–^*t*^*Bu*), 1.72 (dt, 1H, H_2′_, *J* = 13.1, 5.0 Hz), 2.38 (dt, 1H, H_2′_, *J* = 13.4, 6.6 Hz), 3.60 (m, 2H, H_5′_), 3.87 (q, 1H, H_4′_, *J* = 4.0 Hz), 4.40 (m, 4H, H_1′_ + H_3′_ + 2H_6′_), 8.09 (s, 1H, H_2_), 8.20 (s, 1H, H_8_) ppm; ^13^C NMR (75.5 MHz, MeOH-*d*_4_): *δ* −5.3 (2Si–*C*H_3_), −4.6 (2Si–*C*H_3_), 18.9 (Si*C*Me_3_), 19.1 (Si*C*Me_3_), 26.29 (3*C*H_3_–^*t*^Bu), 26.34 (3*C*H_3_–^*t*^Bu), 39.1 (C_2′_), 48.9 (C_6′_), 64.4 (C_5′_), 74.6 (C_3′_), 78.3 (C_1′_), 88.3 (C_4′_), 119.7 (C_4_), 143.5 (C_2_), 150.8 (C_5_), 153.6 (C_8_), 157.2 (C_6_) ppm; HRMS (ESI^+^, *m*/*z*): calcd for C_23_H_44_N_5_O_3_Si_2_ [M + H]^+^: 494.2977, found: 494.2984.

#### 3,5-Bis-*O*-(*tert*-butyldimethylsilyl)-1′-homo-*N*-2′-deoxy-α-uridine (17b)

White solid. mp 102–103 °C; *R*_f_: 0.49 (5% MeOH/CH_2_Cl_2_); ^1^H NMR (300.13 MHz, MeOH-*d*_4_): *δ* 0.05 (s, 3H, Si–*Me*), 0.06 (s, 3H, Si–*Me*), 0.11 (s, 6H, Si–*Me*), 0.90 (s, 9H, Si–^*t*^*Bu*), 0.92 (s, 9H, Si–^*t*^*Bu*), 1.70 (dt, 1H, H_2′_, *J* = 13.0, 4.9 Hz), 2.32 (dt, 1H, H_2′_, *J* = 13.5, 6.9 Hz), 3.62 (m, 2H, H_5′_), 3.87 (m, 2H, H_4′_ + H_6′_), 3.96 (dd, 1H, H_6′_, *J* = 14.1, 3.0 Hz), 4.32 (m, 1H, H_1′_), 4.43 (m, 1H, H_3′_), 5.62 (d, 1H, H_5_, *J* = 7.8 Hz), 7.53 (d, 1H, H_6_, *J* = 7.9 Hz) ppm; ^13^C NMR (75.5 MHz, MeOH-*d*_4_): *δ* −5.3 (Si–*C*H_3_), −5.2 (Si–*C*H_3_), −4.6 (Si–*C*H_3_), −4.5 (Si–*C*H_3_), 18.9 (Si*C*Me_3_), 19.2 (Si*C*Me_3_), 26.3 (3*C*H_3_–^*t*^Bu), 26.4 (3*C*H_3_–^*t*^Bu), 38.9 (C_2′_), 53.4 (C_6′_), 64.4 (C_5′_), 74.6 (C_3′_), 78.1 (C_1′_), 88.2 (C_4′_), 101.6 (C_5_), 148.4 (C_6_), 152.9 (C_2_), 166.8 (C_4_) ppm; HRMS (ESI^+^, *m*/*z*): calcd for C_22_H_42_N_2_NaO_5_Si_2_ [M + Na]^+^: 493.2524, found: 493.2529.

#### 3,5-Bis-*O*-(*tert*-butyldimethylsilyl)-1′-homo-*N*-α-thymidine (17c)

*R*_f_: 0.16 (50% Et_2_O/hexane); ^1^H NMR (300.13 MHz, MeOH-*d*_4_): *δ* 0.05 (s, 3H, Si–*Me*), 0.06 (s, 3H, Si–*Me*), 0.11 (s, 6H, Si–*Me*), 0.90 (s, 9H, Si–^*t*^*Bu*), 0.92 (s, 9H, Si–^*t*^*Bu*), 1.70 (ddd, 1H, H_2′_, *J* = 12.9, 5.9, 4.5 Hz), 1.86 (d, 3H, *Me–*C_5_, *J* = 1.1 Hz), 2.32 (dt, 1H, H_2′_, *J* = 13.6, 6.6 Hz), 3.62 (dd, 2H, H_5′_, *J* = 4.4, 1.9 Hz), 3.88 (m, 3H, H_4′_ + 2H_6′_), 4.31 (m, 1H, H_1′_), 4.41 (m, 1H, H_3′_), 7.37 (s, 1H, H_6_) ppm; ^13^C NMR (75.5 MHz, MeOH-*d*_4_): *δ* −5.30 (Si–*C*H_3_), −5.26 (Si–*C*H_3_), −4.6 (Si–*C*H_3_), −4.5 (Si–*C*H_3_), 12.3 (*C*H_3_–C_5_) 18.9 (Si*C*Me_3_), 19.2 (Si*C*Me_3_), 26.3 (3*C*H_3_–^*t*^Bu), 26.4 (3*C*H_3_–^*t*^Bu), 39.0 (C_2′_), 53.1 (C_6′_), 64.4 (C_5′_), 74.6 (C_3′_), 78.2 (C_1′_), 88.2 (C_4′_), 110.4 (C_5_), 144.2 (C_6_), 152.9 (C_2_), 167.0 (C_4_) ppm; HRMS (ESI^+^, *m*/*z*): calcd for C_23_H_45_N_2_O_5_Si_2_ [M + H]^+^: 485.2862, found: 485.2851.

#### 3,5-Bis-*O*-(*tert*-butyldimethylsilyl)-1′-homo-*N*-2′-deoxy-α-5-fluorouridine (17d)

White solid. mp 134–136 °C; *R*_f_: 0.57 (5% MeOH/CH_2_Cl_2_); ^1^H NMR (300.13 MHz, MeOH-*d*_4_): *δ* 0.05 (s, 3H, Si–*Me*), 0.07 (s, 3H, Si–*Me*), 0.10 (s, 3H, Si–*Me*), 0.11 (s, 3H, Si–*Me*), 0.90 (s, 9H, Si–^*t*^*Bu*), 0.91 (s, 9H, Si–^*t*^*Bu*), 1.70 (ddd, 1H, H_2′_, *J* = 13.0, 5.7, 4.4 Hz), 2.34 (dt, 1H, H_2′_, *J* = 13.7, 7.0 Hz), 3.62 (dd, 2H, H_5′_, *J* = 4.3, 1.7 Hz), 3.88 (m, 3H, H_4′_ + 2H_6′_), 4.33 (m, 1H, H_1′_), 4.42 (m, 1H, H_3′_), 7.74 (d, 1H, H_6_, *J* = 6.3 Hz) ppm; ^13^C NMR (75.5 MHz, MeOH-*d*_4_): *δ* −5.31 (Si–*C*H_3_), −5.26 (Si–*C*H_3_), −4.6 (Si–*C*H_3_), −4.55 (Si–*C*H_3_), 18.9 (Si*C*Me_3_), 19.2 (Si*C*Me_3_), 26.3 (3*C*H_3_–^*t*^Bu), 26.4 (3*C*H_3_–^*t*^Bu), 38.9 (C_2′_), 53.2 (C_6′_), 64.4 (C_5′_), 74.6 (C_3′_), 78.2 (C_1′_), 88.2 (C_4′_), 132.3 (d, C_6_, *J* = 33.5 Hz), 141.2 (d, C_5_, *J* = 131.5 Hz), 151.6 (C_2_), 159.9 (d, C_4_, *J* = 25.3 Hz) ppm; HRMS (ESI^+^, *m*/*z*): calcd for C_22_H_42_FN_2_O_5_Si_2_ [M + H]^+^: 489.2611, found: 489.2635 and calcd for C_22_H_41_FN_2_NaO_5_Si_2_ [M + Na]^+^: 511.2430, found: 511.2456.

#### 3,5-Bis-*O*-(*tert*-butyldimethylsilyl)-1′-homo-*N*-2′-deoxy-α-cytidine (17e)

*R*_f_: 0.22 (5% MeOH/CH_2_Cl_2_); ^1^H NMR (300.13 MHz, MeOH-*d*_4_): *δ* 0.04 (s, 3H, Si–*Me*), 0.05 (s, 3H, Si–*Me*), 0.11 (s, 6H, Si–*Me*), 0.89 (s, 9H, Si–^*t*^*Bu*), 0.92 (s, 9H, Si–^*t*^*Bu*), 1.68 (dt, 1H, H_2′_, *J* = 12.8, 5.2 Hz), 2.32 (dt, 1H, H_2′_, *J* = 13.3, 6.8 Hz), 3.61 (m, 2H, H_5′_), 3.77 (dd, 1H, H_6′_, *J* = 13.7, 8.5 Hz), 3.85 (q, 1H, H_4′_, *J* = 4.1 Hz), 4.05 (dd, 1H, H_6′_, *J* = 13.8, 2.7 Hz), 4.32 (m, 1H, H_1′_), 4.41 (dt, 1H, H_3′_, *J* = 5.9, 4.2 Hz), 5.82 (d, 1H, H_5_, *J* = 7.2 Hz), 7.50 (d, 1H, H_6_, *J* = 7.2 Hz) ppm; ^13^C NMR (75.5 MHz, MeOH-*d*_4_): *δ* −5.3 (Si–*C*H_3_), −5.2 (Si–*C*H_3_), −4.6 (Si–*C*H_3_), −4.5 (Si–*C*H_3_), 18.9 (Si*C*Me_3_), 19.2 (Si*C*Me_3_), 26.3 (3*C*H_3_–^*t*^Bu), 26.4 (3*C*H_3_–^*t*^Bu), 39.0 (C_2′_), 54.9 (C_6′_), 64.4 (C_5′_), 74.5 (C_3′_), 78.0 (C_1′_), 88.0 (C_4′_), 95.2 (C_5_), 148.5 (C_6_), 159.0 (C_2_), 168.0 (C_4_) ppm; HRMS (ESI^+^, *m*/*z*): calcd for C_22_H_44_N_3_O_4_Si_2_ [M + H]^+^: 470.2865, found: 470.2868.

#### 3,5-Bis-*O*-(*tert*-butyldimethylsilyl)-1′-homo-*N*-2′-deoxy-α-5-iodouridine (17f)

*R*_f_: 0.63 (5% MeOH/CH_2_Cl_2_); ^1^H NMR (300.13 MHz, MeOH-*d*_4_): *δ* 0.05 (s, 3H, Si–*Me*), 0.07 (s, 3H, Si–*Me*), 0.11 (s, 6H, Si–*Me*), 0.90 (s, 9H, Si–^*t*^*Bu*), 0.92 (s, 9H, Si–^*t*^*Bu*), 1.70 (ddd, 1H, H_2′_, *J* = 13.0, 5.9, 4.4 Hz), 2.33 (ddd, 1H, H_2′_, *J* = 13.5, 7.6, 6.4 Hz), 3.62 (dd, 2H, H_5′_, *J* = 4.4, 1.6 Hz), 3.90 (m, 3H, H_4′_ + 2H_6′_), 4.30 (m, 1H, H_1′_), 4.42 (m, 1H, H_3′_), 7.98 (s, 1H, H_6_) ppm; ^13^C NMR (75.5 MHz, MeOH-*d*_4_): *δ* −5.2 (2Si–*C*H_3_), −4.56 (Si–*C*H_3_), −4.52 (Si–*C*H_3_), 18.9 (Si*C*Me_3_), 19.2 (Si*C*Me_3_), 26.37 (3*C*H_3_–^*t*^Bu), 26.42 (3*C*H_3_–^*t*^Bu), 38.9 (C_2′_), 53.2 (C_6′_), 64.4 (C_5′_), 67.2 (C_5_), 74.7 (C_3′_), 78.0 (C_1′_), 88.3 (C_4′_), 152.6 (C_6_), 152.7 (C_2_), 163.4 (C_4_) ppm; HRMS (ESI^+^, *m*/*z*): calcd for C_22_H_42_IN_2_O_5_Si_2_ [M + H]^+^: 597.1671, found: 597.1684 and calcd for C_22_H_41_IN_2_NaO_5_Si_2_ [M + Na]^+^: 619.1491, found: 619.1504.

#### *N* ^1^,*N*^3^-Bis-[3,5-bis-*O*-(*tert*-butyldimethylsilyl)-1,2-dideoxy-α-ribofuranosylmethyl]uracil (18b)

Colourless oil. *R*_f_: 0.40 (20% EtOAc/hexane); ^1^H NMR (300.13 MHz, MeOH-*d*_4_): *δ* 0.04 (s, 6H, Si–*Me*), 0.05 (s, 3H, Si–*Me*), 0.06 (s, 3H, Si–*Me*), 0.11 (s, 12H, Si–*Me*), 0.89 (s, 9H, Si–^*t*^*Bu*), 0.90 (s, 9H, Si–^*t*^*Bu*), 0.92 (s, 9H, Si–^*t*^*Bu*), 0.93 (s, 9H, Si–^*t*^*Bu*), 1.74 (m, 2H, H_2′_), 2.27 (m, 2H, H_2′_), 3.60 (m, 4H, H_5′_), 3.78 (dd, 1H, H_7′_, *J* = 12.9, 3.9 Hz), 3.92 (m, 4H, 2H_4′_ + 2H_6′_), 4.38 (m, 4H, 2H_1′_ + 2H_3′_), 4.56 (dd, 1H, H_7′_, *J* = 12.9, 8.8 Hz), 5.69 (d, 1H, H_5_, *J* = 7.9 Hz), 7.51 (d, 1H, H_6_, *J* = 7.9 Hz) ppm; ^13^C NMR (75.5 MHz, MeOH-*d*_4_): *δ* −5.3 (2Si–*C*H_3_), −5.2 (2Si–*C*H_3_), −4.6 (2Si–*C*H_3_), −4.5 (2Si–*C*H_3_), 18.8 (Si*C*Me_3_), 18.9 (Si*C*Me_3_), 19.2 (2Si*C*Me_3_), 26.36 (6*C*H_3_–^*t*^Bu), 26.42 (3*C*H_3_–^*t*^Bu), 26.44 (3*C*H_3_–^*t*^Bu), 39.0 (C_2′_), 39.8 (C_2′_), 46.2 (C_7′_), 54.5 (C_6′_), 64.4 (2C_5′_), 74.6 (C_3′_), 74.7 (C_3′_), 77.4 (C_1′_), 78.1 (C_1′_), 88.0 (C_4′_), 88.2 (C_4′_), 101.0 (C_5_), 146.4 (C_6_), 153.2 (C_2_), 165.7 (C_4_) ppm; HRMS (ESI^+^, *m*/*z*): calcd for C_40_H_81_N_2_O_8_Si_4_ [M + H]^+^: 829.5065, found: 829.5065.

#### *N* ^1^,*N*^3^-Bis-[3,5-bis-*O*-(*tert*-butyldimethylsilyl)-1,2-dideoxy-α-ribofuranosylmethyl]thymine (18c)

Colourless oil. *R*_f_: 0.50 (20% EtOAc/hexane); ^1^H NMR (300.13 MHz, MeOH-*d*_4_): *δ* 0.04 (s, 6H, Si–*Me*), 0.049 (s, 3H, Si–*Me*), 0.054 (s, 3H, Si–*Me*), 0.108 (s, 6H, Si–*Me*), 0.114 (s, 6H, Si–*Me*), 0.891 (s, 9H, Si–^*t*^*Bu*), 0.893 (s, 9H, Si–^*t*^*Bu*), 0.92 (s, 9H, Si–^*t*^*Bu*), 0.93 (s, 9H, Si–^*t*^*Bu*), 1.74 (m, 2H, H_2′_), 1.89 (d, 3H, H_7_, *J* = 0.8 Hz), 2.27 (m, 2H, H_2′_), 3.60 (m, 4H, H_5′_), 3.79 (dd, 1H, H_7′_, *J* = 13.0, 3.9 Hz), 3.90 (m, 4H, 2H_4′_ + 2H_6′_), 4.36 (m, 4H, 2H_1′_ + 2H_3′_), 4.58 (dd, 1H, H_7′_, *J* = 13.0, 8.8 Hz), 7.37 (d, 1H, H_6_, *J* = 1.0 Hz) ppm; ^13^C NMR (75.5 MHz, MeOH-*d*_4_): *δ* −5.3 (2Si–*C*H_3_), −5.2 (2Si–*C*H_3_), −4.6 (2Si–*C*H_3_), −4.5 (2Si–*C*H_3_), 13.1 (C_7_), 18.8 (Si*C*Me_3_), 18.9 (Si*C*Me_3_), 19.2 (2Si*C*Me_3_), 26.36 (6*C*H_3_–^*t*^Bu), 26.42 (6*C*H_3_–^*t*^Bu), 39.0 (C_2′_), 39.9 (C_2′_), 46.5 (C_7′_), 54.2 (C_6′_), 64.4 (C_5′_), 64.5 (C_5′_), 74.6 (C_3′_), 74.7 (C_3′_), 77.5 (C_1′_), 78.2 (C_1′_), 88.0 (C_4′_), 88.1 (C_4′_), 109.6 (C_5_), 142.4 (C_6_), 153.2 (C_2_), 166.0 (C_4_) ppm; HRMS (ESI^+^, *m*/*z*): calcd for C_41_H_83_N_2_O_8_Si_4_ [M + H]^+^: 843.5221, found: 843.5218.

#### *N* ^1^,*N*^3^-Bis-[3,5-bis-*O*-(*tert*-butyldimethylsilyl)-1,2-dideoxy-α-ribofuranosylmethyl]-5-fluorouracil (18d)

Colourless oil. *R*_f_: 0.50 (20% EtOAc/hexane); ^1^H NMR (300.13 MHz, MeOH-*d*_4_): *δ* 0.039 (s, 3H, Si–*Me*), 0.041 (s, 3H, Si–*Me*), 0.05 (s, 3H, Si–*Me*), 0.06 (s, 3H, Si–*Me*), 0.10 (s, 6H, Si–*Me*), 0.113 (s, 3H, Si–*Me*), 0.115 (s, 3H, Si–*Me*), 0.890 (s, 9H, Si–^*t*^*Bu*), 0.892 (s, 9H, Si–^*t*^*Bu*), 0.91 (s, 9H, Si–^*t*^*Bu*), 0.93 (s, 9H, Si–^*t*^*Bu*), 1.75 (m, 2H, H_2′_), 2.28 (m, 2H, H_2′_), 3.58 (m, 4H, H_5′_), 3.77 (dd, 1H, H_7′_, *J* = 13.1, 3.7 Hz), 3.92 (m, 4H, 2H_4′_ + 2H_6′_), 4.38 (m, 4H, 2H_1′_ + 2H_3′_), 4.62 (dd, 1H, H_7′_, *J* = 12.9, 9.1 Hz), 7.75 (d, 1H, H_6_, *J* = 5.9 Hz) ppm; ^13^C NMR (75.5 MHz, MeOH-*d*_4_): *δ* −5.24 (2Si–*C*H_3_), −5.17 (Si–*C*H_3_), −5.1 (Si–*C*H_3_), −4.54 (2Si–*C*H_3_), −4.48 (2Si–*C*H_3_), 18.8 (Si*C*Me_3_), 18.9 (Si*C*Me_3_), 19.2 (2Si*C*Me_3_), 26.38 (6*C*H_3_–^*t*^Bu), 26.43 (3*C*H_3_–^*t*^Bu), 26.5 (3*C*H_3_–^*t*^Bu), 39.0 (C_2′_), 39.7 (C_2′_), 46.9 (C_7′_), 54.3 (C_6′_), 64.4 (C_5′_), 64.5 (C_5′_), 74.6 (C_3′_), 74.8 (C_3′_), 77.3 (C_1′_), 78.1 (C_1′_), 88.1 (2C_4′_), 130.6 (d, C_6_, *J* = 33.3 Hz), 140.8 (d, C_5_, *J* = 229.4 Hz), 151.7 (C_2_), 159.5 (d, C_4_, *J* = 25.2 Hz) ppm; HRMS (ESI^+^, *m*/*z*): calcd for C_40_H_79_FN_2_NaO_8_Si_4_ [M + Na]^+^: 869.4790, found: 869.4762.

#### *N* ^1^,*N*^3^-Bis-[3,5-bis-*O*-(*tert*-butyldimethylsilyl)-1,2-dideoxy-α-ribofuranosylmethyl]-5-iodouracil (18f)

Colourless oil. *R*_f_: 0.56 (20% EtOAc/hexane); ^1^H NMR (300.13 MHz, MeOH-*d*_4_): *δ* 0.04 (s, 3H, Si–*Me*), 0.050 (s, 3H, Si–*Me*), 0.052 (s, 3H, Si–*Me*), 0.07 (s, 3H, Si–*Me*), 0.108 (s, 6H, Si–*Me*), 0.114 (s, 6H, Si–*Me*), 0.89 (s, 9H, Si–^*t*^*Bu*), 0.90 (s, 9H, Si–^*t*^*Bu*), 0.92 (s, 9H, Si–^*t*^*Bu*), 0.93 (s, 9H, Si–^*t*^*Bu*), 1.73 (m, 2H, H_2′_), 2.28 (m, 2H, H_2′_), 3.62 (m, 4H, H_5′_), 3.82 (dd, 1H, H_7′_, *J* = 13.1, 3.7 Hz), 3.89 (m, 2H, 2H_4′_), 3.96 (m, 2H, 2H_6′_), 4.38 (m, 4H, 2H_1′_ + 2H_3′_), 4.63 (dd, 1H, H_7′_, *J* = 13.0, 9.0 Hz), 7.99 (s, 1H, H_6_) ppm; ^13^C NMR (75.5 MHz, MeOH-*d*_4_): *δ* −5.24 (Si–*C*H_3_), −5.19 (Si–*C*H_3_), −5.1 (2Si–*C*H_3_), −4.5 (4Si–*C*H_3_), 18.8 (Si*C*Me_3_), 18.9 (Si*C*Me_3_), 19.2 (2Si*C*Me_3_), 26.4 (6*C*H_3_–^*t*^Bu), 26.5 (6*C*H_3_–^*t*^Bu), 38.9 (C_2′_), 39.8 (C_2′_), 47.8 (C_7′_), 54.4 (C_6′_), 64.4 (2C_5′_), 66.5 (C_5_), 74.6 (C_3′_), 74.7 (C_3′_), 77.4 (C_1′_), 78.0 (C_1′_), 88.0 (C_4′_), 88.3 (C_4′_), 150.9 (C_6_), 152.9 (C_2_), 162.4 (C_4_) ppm; HRMS (ESI^+^, *m*/*z*): calcd for C_40_H_80_IN_2_O_8_Si_4_ [M + H]^+^: 955.4031, found: 955.4033 and calcd for C_40_H_79_IN_2_NaO_8_Si_4_ [M + Na]^+^: 977.3850, found: 977.3851.

### 3,5-Bis-*O*-(*tert*-butyldimethylsilyl)-1,2-dideoxy-1α-((acetoxy)methyl)-d-ribofuranose (19)

To a solution of 15 (44 mg, 0.12 mmol) in anhydrous CH_2_Cl_2_ (1.2 mL) at room temperature was added Et_3_N (50 μL, 0.36 mmol), Ac_2_O (170 μL, 1.8 mmol) and catalytic DMAP (2 mg). The solution was stirred for 2 h. Solvents were evaporated, and the residue purified by column chromatography (10% EtOAc/hexane) to afford 19 in 90% yield as a viscous liquid. *R*_f_: 0.65 (20% EtOAc/hexane); ^1^H NMR (300.13 MHz, MeOH-*d*_4_): *δ* 0.081 (s, 3H, Si–*Me*), 0.085 (s, 3H, Si–*Me*), 0.10 (s, 6H, Si–*Me*), 0.91 (s, 9H, Si–^*t*^*Bu*), 0.92 (s, 9H, Si–^*t*^*Bu*), 1.71 (dt, 1H, H_2_, *J* = 13.1, 4.5 Hz), 2.05 (s, 3H, Ac–*Me*), 2.28 (dt, 1H, H_2_, *J* = 13.6, 6.9 Hz), 3.63 (qd, 2H, H_5_, *J* = 11.0, 4.5 Hz), 3.85 (q, 1H, H_4_, *J* = 3.9 Hz), 4.05 (dd, 1H, H_6_, *J* = 10.9, 3.7 Hz), 4.24 (m, 1H, H_6_), 4.27 (m, 1H, H_1_), 4.40 (dt, 1H, H_3_, *J* = 6.2, 3.9 Hz) ppm; ^13^C NMR (75.5 MHz, MeOH-*d*_4_): *δ* −5.3 (Si–*C*H_3_), −5.2 (Si–*C*H_3_), −4.6 (Si–*C*H_3_), −4.5 (Si–*C*H_3_), 18.8 (Si*C*Me_3_), 19.2 (Si*C*Me_3_), 20.8 (Ac–*C*H_3_), 26.3 (3*C*H_3_–^*t*^Bu), 26.4 (3*C*H_3_–^*t*^Bu), 38.1 (C_2_), 64.4 (C_5_), 68.1 (C_6_), 74.4 (C_3_), 78.1 (C_1_), 88.4 (C_4_), 172.6 (C=O) ppm.

### 3,5-Bis-*O*-(*tert*-butyldimethylsilyl)-1,2-dideoxy-1α-((tosyloxy)methyl)-d-ribofuranose (20)

To a solution of 15 (100 mg, 0.26 mmol) in anhydrous pyridine (0.5 mL) at 0 °C were added TsCl (111.3 mg, 0.58 mmol) and catalytic DMAP (5 mg). The solution was stirred at room temperature for 9 h. Solvents were evaporated, and the residue was purified by column chromatography (15% EtOAc/hexane) to yield 20 as a colourless viscous liquid in 90% yield. *R*_f_: 0.60 (20% EtOAc/hexane); ^1^H NMR (300.13 MHz, MeOH-*d*_4_): *δ* 0.040 (s, 3H, Si–*Me*), 0.042 (s, 3H, Si–*Me*), 0.05 (s, 3H, Si–*Me*), 0.06 (s, 3H, Si–*Me*), 0.84 (s, 9H, Si–^*t*^*Bu*), 0.90 (s, 9H, Si–^*t*^*Bu*), 1.64 (dt, 1H, H_2_, *J* = 13.3, 3.4 Hz), 2.21 (ddd, 1H, H_2_, *J* = 13.7, 8.1, 5.8 Hz), 2.45 (s, 3H, Ts–*Me*), 3.46 (dd, 1H, H_5_, *J* = 10.9, 5.6 Hz), 3.57 (dd, 1H, H_5_, *J* = 10.9, 3.8 Hz), 3.69 (m, 1H, H_4_), 3.98 (dd, 1H, H_6_, *J* = 10.0, 4.0 Hz), 4.12 (dd, 1H, H_6_, *J* = 10.0, 7.6 Hz), 4.27 (m, 1H, H_1_), 4.33 (m, 1H, H_3_), 7.43 (d, 2H, H_arom_, *J* = 8.5 Hz), 7.79 (d, 2H, H_arom_, *J* = 8.4 Hz) ppm; ^13^C NMR (75.5 MHz, MeOH-*d*_4_): *δ* −5.3 (Si–*C*H_3_), −5.2 (Si–*C*H_3_), −4.6 (2Si–*C*H_3_), 18.7 (Si*C*Me_3_), 19.2 (Si*C*Me_3_), 21.6 (*C*H_3_–Ts), 26.3 (3*C*H_3_–^*t*^Bu), 26.4 (3^*t*^Bu–*C*H_3_), 37.6 (C_2_), 64.4 (C_5_), 73.6 (C_6_), 74.5 (C_3_), 77.9 (C_1_), 88.7 (C_4_), 129.1 (2C_arom_), 131.0 (2C_arom_), 134.4 (C_ipso_), 146.3 (C_ipso_) ppm; HRMS (ESI^+^, *m*/*z*): calcd for C_25_H_47_O_6_SSi_2_ [M + H]^+^: 531.2626, found: 531.2632.

### General procedure for desilylation of 18d and 18f. Synthesis of 21d and 21f

A procedure analogous to that described for the synthesis of 10 was used.

#### *N* ^1^,*N*^3^-Bis-(1,2-dideoxy-α-ribofuranosylmethyl)-5-fluorouracil (21d)

Colourless oil. *R*_f_: 0.15 (15% MeOH/CH_2_Cl_2_); ^1^H NMR (300.13 MHz, MeOH-*d*_4_): *δ* 1.74 (m, 2H, H_2′_), 2.33 (m, 2H, H_2′_), 3.54 (m, 4H, H_5′_), 3.90 (m, 5H, 2H_4′_ + 2H_6′_ + H_7′_), 4.22 (m, 2H, H_3′_), 4.39 (m, 2H, H_1′_), 4.50 (dd, 1H, H_7′_, *J* = 12.6, 8.8 Hz), 7.84 (d, 1H, H_6_, *J* = 6.0 Hz) ppm; ^13^C NMR (75.5 MHz, MeOH-*d*_4_): *δ* 38.4 (C_2′_), 39.3 (C_2′_), 46.6 (C_7′_), 54.2 (C_6′_), 63.2 (C_5′_), 63.4 (C_5′_), 73.3 (C_3′_), 73.4 (C_3′_), 76.7 (C_1′_), 77.7 (C_1′_), 87.1 (C_4′_), 87.7 (C_4′_), 130.9 (d, C_6_, *J* = 33.5 Hz), 140.8 (d, C_5_, *J* = 229.0 Hz), 151.9 (C_2_), 159.6 (d, C_4_, *J* = 25.0 Hz) ppm; HRMS (ESI^+^, *m*/*z*): calcd for C_16_H_24_FN_2_O_8_ [M + H]^+^: 391.1511, found: 391.1526 and calcd for C_16_H_23_FN_2_NaO_8_ [M + Na]^+^: 413.1331, found: 413.1345.

#### *N* ^1^,*N*^3^-Bis-(1,2-dideoxy-α-ribofuranosylmethyl)-5-iodouracil (21f)

Colourless oil. *R*_f_: 0.24 (15% MeOH/CH_2_Cl_2_); ^1^H NMR (300.13 MHz, MeOH-*d*_4_): *δ* 1.73 (m, 2H, H_2′_), 2.32 (m, 2H, H_2′_), 3.53 (m, 4H, H_5′_), 3.89 (m, 3H, 2H_4′_ + H_7′_), 3.96 (m, 2H, H_6′_), 4.23 (m, 2H, H_3′_), 4.39 (m, 2H, H_1′_), 4.52 (dd, 1H, H_7′_, *J* = 12.9, 8.7 Hz), 8.08 (s, 1H, H_6_) ppm; ^13^C NMR (75.5 MHz, MeOH-*d*_4_): *δ* 38.4 (C_2′_), 39.3 (C_2′_), 47.6 (C_7′_), 54.4 (C_6′_), 63.2 (C_5′_), 63.3 (C_5′_), 66.4 (C_5_), 73.4 (2C_3′_), 76.7 (C_1′_), 77.6 (C_1′_), 87.0 (C_4′_), 87.8 (C_4′_), 151.2 (C_6_), 153.1 (C_2_), 162.5 (C_4_) ppm; HRMS (ESI^+^, *m*/*z*): calcd for C_16_H_24_IN_2_O_8_ [M + H]^+^: 499.0572, found: 499.0577 and calcd for C_16_H_23_IN_2_NaO_8_ [M + Na]^+^: 521.0391, found: 521.0397.

### Antiviral assays

#### HIV assay

The assay was performed as described previously,^23*a*,*b*^ with some modifications described elsewhere.^23*c*^ Briefly, human PBM cells were stimulated with PHA/IL-2 prior to infection with HIV-1 LAI (MOI 0.1) for 6 days in the presence of various concentrations of test compounds (10a–f, 21d, and 21f) or AZT (control). Supernatants were harvested, and HIV-1 RT was quantified. Median effective concentrations (EC_50_) were determined using described method.^23*d*^

#### HBV assay

HepAD38 cells^24^ were seeded at 50 000 cells per well in collagen-coated 96-well plates. Test compounds or 3TC (control) were added to HepAD38 cells to a final concentration of 10 μM. Real-time PCR for HBV DNA lasted 7 days. On day 7, total DNA was purified from supernatant using commercially available kit (DNeasy 96 Blood & Tissue kit, Qiagen). The HBV DNA was amplified in a real-time PCR assay using LightCycler 480 (Roche) as described elsewhere.^23*c*^ All samples were tested in triplicate. Analysis: the concentration of compound that inhibited HBV DNA replication by 50% (EC_50_) was determined by linear regression.

#### Cytotoxicity assays

These were performed in primary blood mononuclear (PBM), T lymphoblast CEM-CCRF (CEM), African green monkey kidney (Vero) or human liver (HepG2) cells *via* MTT assay using the CellTiter 96^®^. Non-Radioactive Cell Proliferation (Promega) kit as previously described.^23*c*^ Cytotoxicity was expressed as the concentration of test compounds that inhibited cell proliferation by 50% (IC_50_) and calculated using the Chou and Talalay method.^25^

## 4. Conclusions

We have synthesized a series of novel 1′-homo-*N*-2′-deoxy-α-nucleoside analogs of A, U, T, 5-FU, C, and 5-IU *via* coupling of a nucleobase and a tosylated intermediate sugar precursor readily obtained from a cyano derivative, which is easily accessible on large-scale. Displacement reactions took place in the presence of K_2_CO_3_ and 18-crown-6 in DMF at elevated temperature. The structure of all derivatives was established by NMR spectroscopy and confirmation was obtained by a single crystal X-ray analysis of 1′-homo-*N*-2′-deoxy-α-adenosine. It is worthy of mention that only the thymine derivative has been previously described synthesized *via* a more complex route. In addition to the desired compounds we have obtained the corresponding dimer derivatives resulting from the coupling of the sugar moiety at the *N*-1 and *N*-3 positions of the base. The occurrence of these dimers is very interesting since few derivatives of this type have been reported in the literature, primarily as byproducts. Eight homonucleosides, including dimers, were tested as antiviral agents. These compounds did not exhibit selective antiviral activity against HIV-1 and HBV. Also, none of them exhibited apparent cytotoxicity in all four cells types tested. Although the results obtained from the preliminary biological screening led to no hit, we believe these novel α-nucleosides may serve as important building-blocks for therapeutic oligonucleotides.^10^ This study established a facile path for the synthesis of multifunctional nucleoside analogs that may elicit biological activity and make an attractive scaffold for drug discovery using the combinatorial approach.

## Conflicts of interest

There are no conflicts to declare.

## Supplementary Material

RA-010-D0RA03254A-s001

RA-010-D0RA03254A-s002
