# Supplementary material for: Novel 1′-homo-N-2′-deoxy-α-nucleosides: synthesis, characterization and biological activity
Source: RSC Adv. 2020 Apr 21;10(27):15815–24. doi: 10.1039/d0ra03254a (PMC8486263; doi:10.1039/d0ra03254a)

Electronic Supplementary Material (ESI) for RSC Advances.
This journal is © The Royal Society of Chemistry 2020

Novel 1’-Homo-*N*-2’-deoxy-α-nucleosides: Synthesis, Characterization and Biological Activity

Alejandro Carnero,[a] Virginia Martín-Nieves,[a] Yogesh S. Sanghvi,[b] Olivia O. Russel,[c] Leda Bassit,[c] Raymond F. Schinazi,[c] Susana Fernández[a,*] and Miguel Ferrero,[a,*]

[a] *Departamento de Química Orgánica e Inorgánica, Universidad de Oviedo, Avenida Julián de Clavería 8, 33006-Oviedo (Asturias), Spain*

[b] *Rasayan Inc., 2802 Crystal Ridge Road, Encinitas, CA 92024-6615, USA*

[c] *Center for AIDS Research, Laboratory of Biochemical Pharmacology, Department of Pediatrics, Emory University School of Medicine, Atlanta, GA 30322, USA*

M.F.: mferrero@uniovi.es; S.F.: fernandezgsusana@uniovi.es.

SUPPORTING INFORMATION (page 1 of 145 pages)

Table of Contents

COPIES OF 1D and 2D NMR SPECTRA OF COMPOUNDS:

^{1}H NMR of **10a** S3
^{13}C NMR of **10a** S4
DEPT-135 NMR of **10a** S5
COSY of **10a** S6
HSQC of **10a** S7
HMBC of **10a** S8
^{1}H NMR of **10b** S9
^{13}C NMR of **10b** S10
DEPT-135 NMR of **10b** S11
COSY of **10b** S12
HSQC of **10b** S13
HMBC of **10b** S110
^{1}H NMR of **10c** S15
^{13}C NMR of **10c** S16
DEPT-135 NMR of **10c** S17
COSY of **10c** S18
HSQC of **10c** S19
HMBC of **10c** S20
^{1}H NMR of **10d** S21
^{13}C NMR of **10d** S22
DEPT-135 NMR of **10d** S23
COSY of **10d** S210
HSQC of **10d** S25
HMBC of **10d** S26
^{1}H NMR of **10e** S27
^{13}C NMR of **10e** S28
DEPT-135 NMR of **10e** S29
COSY of **10e** S30
HSQC of **10e** S31
HMBC of **10e** S32
^{1}H NMR of **10f** S33
COSY of **10f** S34
^{1}H NMR of **13** S35
^{13}C NMR of **13** S36
DEPT-135 NMR of **13** S37
COSY of **13** S38
HSQC of **13** S39
HMBC of **13** S40
^{1}H NMR of **14** S41
^{13}C NMR of **14** S42
DEPT-135 NMR of **14** S43
COSY of **14** S44
HSQC of **14** S45
HMBC of **14** S46
^{1}H NMR of **15** S47
^{13}C NMR of **15** S48
DEPT-135 NMR of **15** S49
COSY of **15** S50
HSQC of **15** S51
HMBC of **15** S52
^{1}H NMR of **16** S53
^{13}C NMR of **16** S54
DEPT-135 NMR of **16** S55
COSY of **16** S56
HSQC of **16** S57
HMBC of **16** S58

^{1}H NMR of **17a**	S59
^{13}C NMR of **17a**	S60
DEPT-135 NMR of **17a**	S61
COSY of **17a**	S62
HSQC of **17a**	S63
HMBC of **17a**	S64
^{1}H NMR of **17b**	S65
^{13}C NMR of **17b**	S66
DEPT-135 NMR of **17b**	S67
COSY of **17b**	S68
HSQC of **17b**	S69
HMBC of **17b**	S70
^{1}H NMR of **17c**	S71
^{13}C NMR of **17c**	S72
DEPT-135 NMR of **17c**	S73
COSY of **17c**	S74
HSQC of **17c**	S75
HMBC of **17c**	S76
^{1}H NMR of **17d**	S77
^{13}C NMR of **17d**	S78
DEPT-135 NMR of **17d**	S79
COSY of **17d**	S80
HSQC of **17d**	S81
HMBC of **17d**	S82
^{1}H NMR of **17e**	S83
^{13}C NMR of **17e**	S84
DEPT-135 NMR of **17e**	S85
COSY of **17e**	S86
HSQC of **17e**	S87
HMBC of **17e**	S88
^{1}H NMR of N-3 analogue of **17e**	S89
^{13}C NMR of N-3 analogue of **17e**	S90
DEPT-135 NMR of N-3 anal. of **17e**	S91
COSY of N-3 analogue of **17e**	S92
HSQC of N-3 analogue of **17e**	S93
HMBC of N-3 analogue of **17e**	S94
^{1}H NMR of **17f**	S95
^{13}C NMR of **17f**	S96
DEPT-135 NMR of **17f**	S97
COSY of **17f**	S98
HSQC of **17f**	S99
HMBC of **17f**	S100
^{1}H NMR of **18b**	S101
^{13}C NMR of **18b**	S102
DEPT-135 NMR of **18b**	S103
COSY of **18b**	S104
HSQC of **18b**	S105
HMBC of **18b**	S106
^{1}H NMR of **18c**	S107
^{13}C NMR of **18c**	S108
DEPT-135 NMR of **18c**	S109
COSY of **18c**	S110
HSQC of **18c**	S111
HMBC of **18c**	S118
^{1}H NMR of **18d**	S113
^{13}C NMR of **18d**	S114
DEPT-135 NMR of **18d**	S115
COSY of **18d**	S116
HSQC of **18d**	S117
HMBC of **18d**	S118
^{1}H NMR of **18f**	S119
^{13}C NMR of **18f**	S180
DEPT-135 NMR of **18f**	S181
COSY of **18f**	S182
HSQC of **18f**	S183
HMBC of **18f**	S124
^{1}H NMR of **19**	S125
^{13}C NMR of **19**	S126
DEPT-135 NMR of **19**	S127
^{1}H NMR of **20**	S128
^{13}C NMR of **20**	S129
DEPT-135 NMR of **20**	S130
COSY of **20**	S131
HSQC of **20**	S132
HMBC of **20**	S133
^{1}H NMR of **21d**	S134
^{13}C NMR of **21d**	S135
DEPT-135 NMR of **21d**	S136
COSY of **21d**	S137
HSQC of **21d**	S138
HMBC of **21d**	S139
^{1}H NMR of **21f**	S140
^{13}C NMR of **21f**	S141
DEPT-135 NMR of **21f**	S142
COSY of **21f**	S143
HSQC of **21f**	S144
HMBC of **21f**	S145

1’-Homo-*N*-2’-deoxy-α-adenosine (10a)

^{1}H NMR (300.13 MHz, MeOH-d_4)

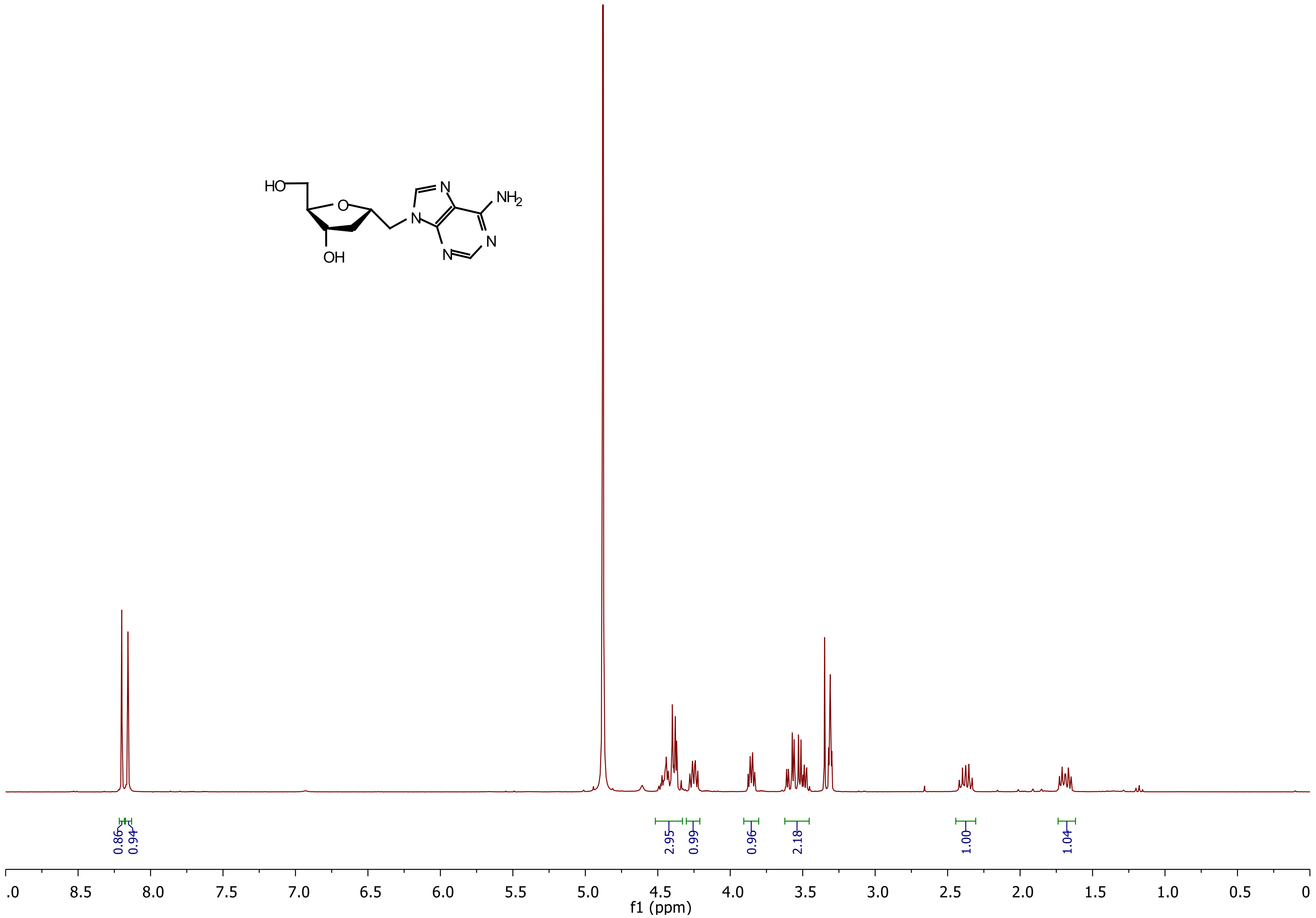

1’-Homo-*N*-2’-deoxy-α-adenosine (10a)

^{13}C NMR (75.5 MHz, MeOH-d_4)

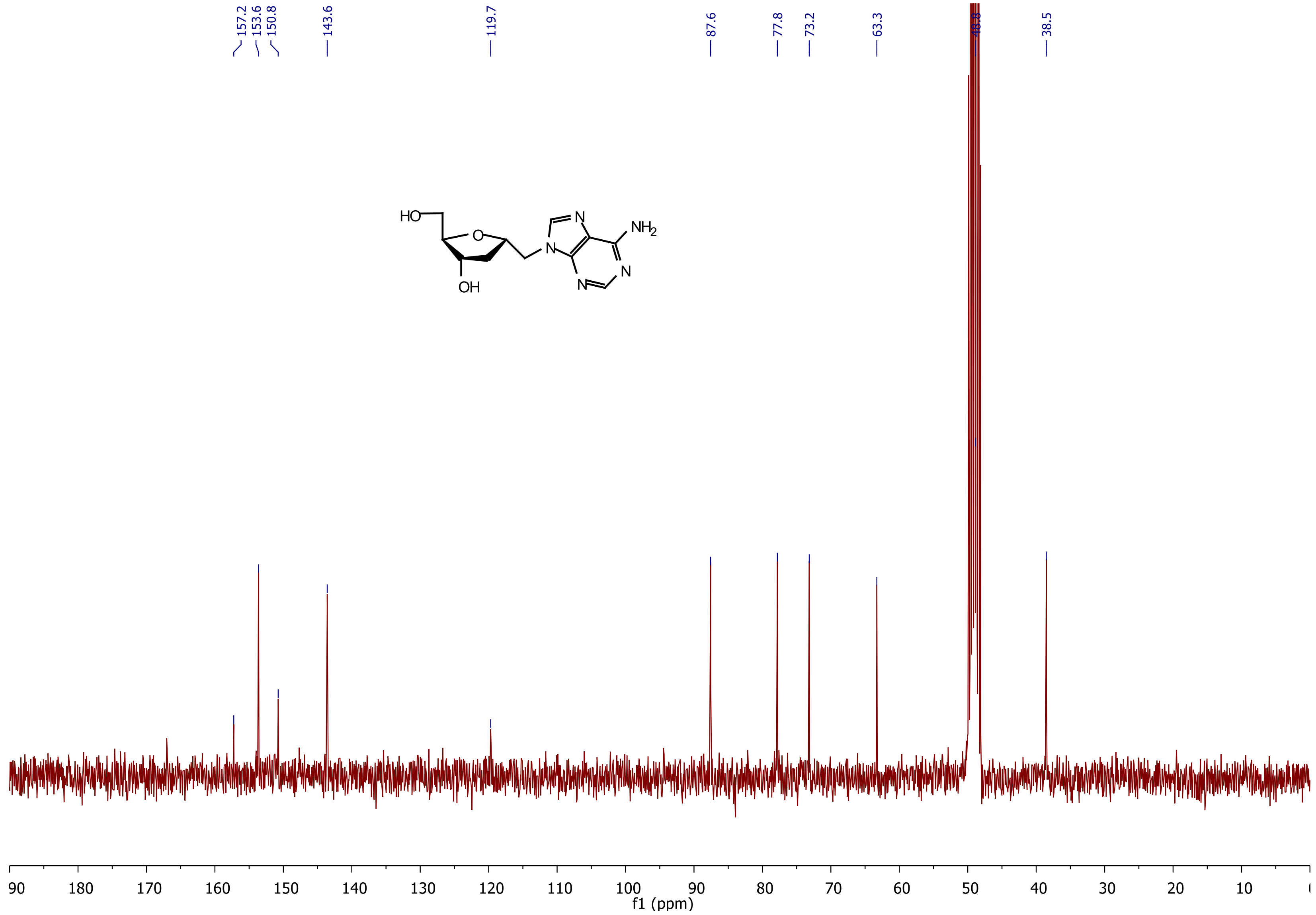

1’-Homo-*N*-2’-deoxy-α-adenosine (10a)

DEPT NMR (75.5 MHz, MeOH-d_4)

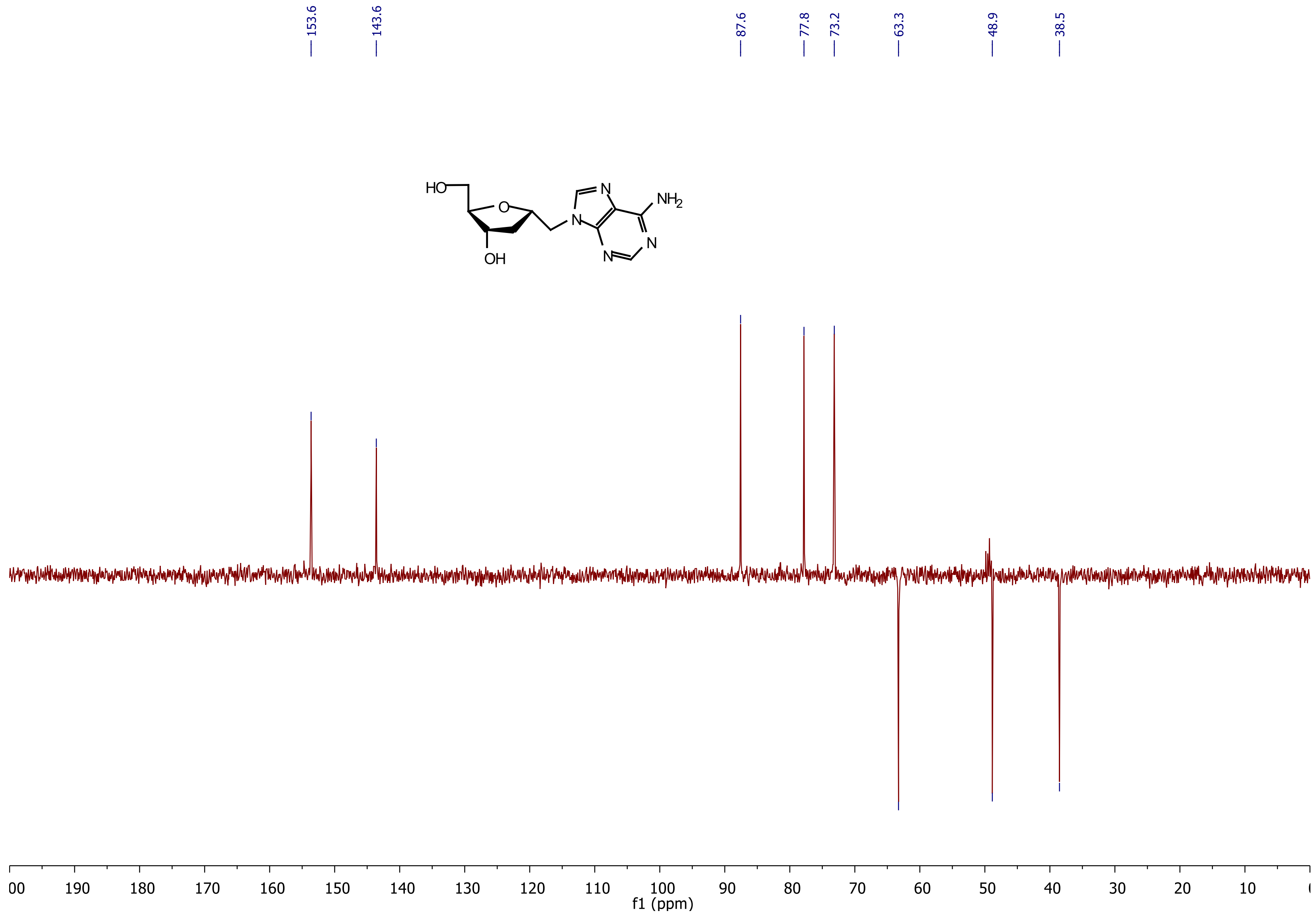

1’-Homo-*N*-2’-deoxy-α-adenosine (10a)

COSY NMR (MeOH-d_4)

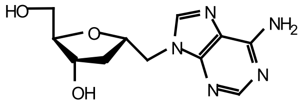

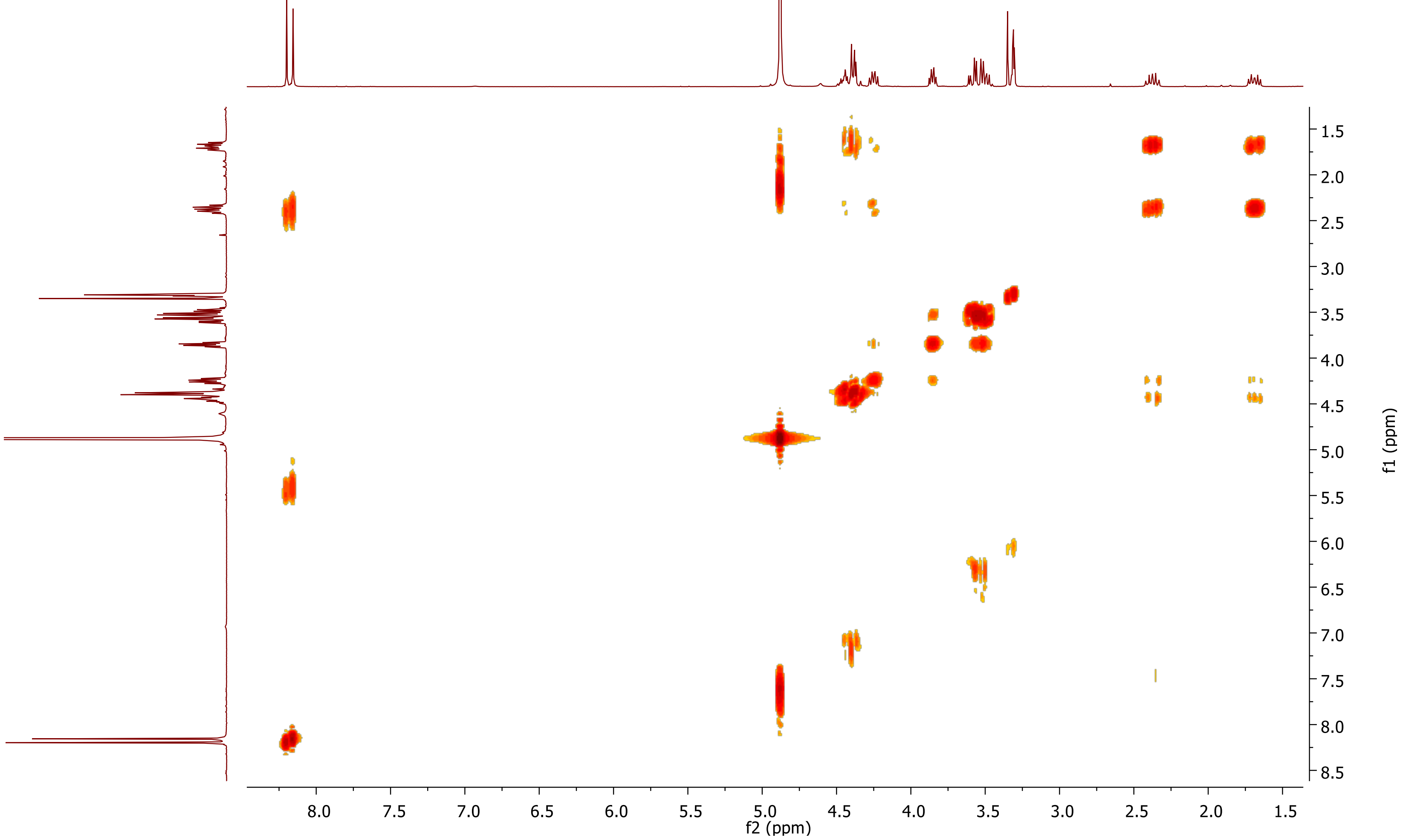

1’-Homo-*N*-2’-deoxy-α-adenosine (10a)

HSQC NMR (MeOH-d_4)

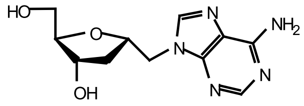

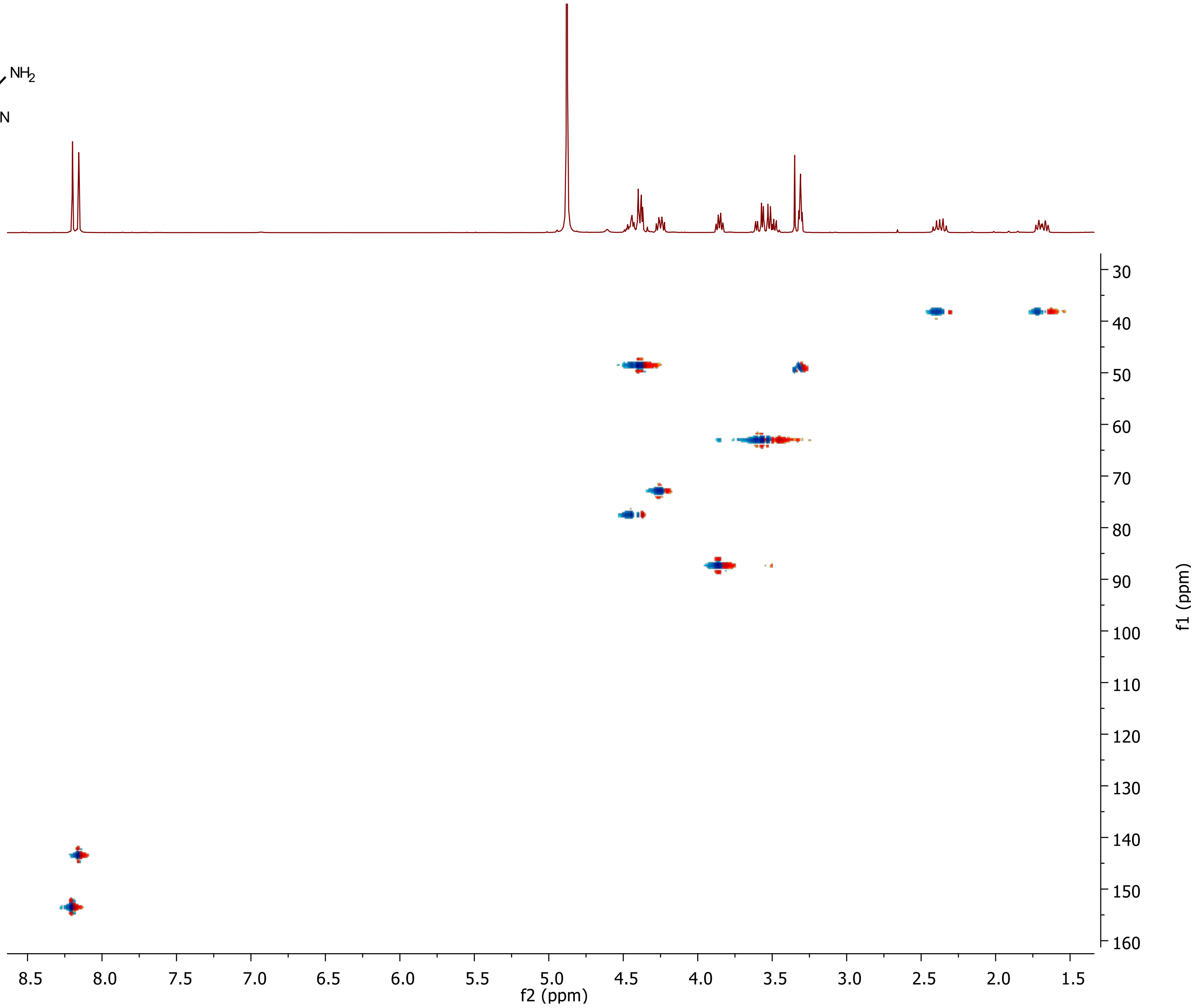

1’-Homo-*N*-2’-deoxy-α-adenosine (10a)

HMBC NMR (MeOH-d_4)

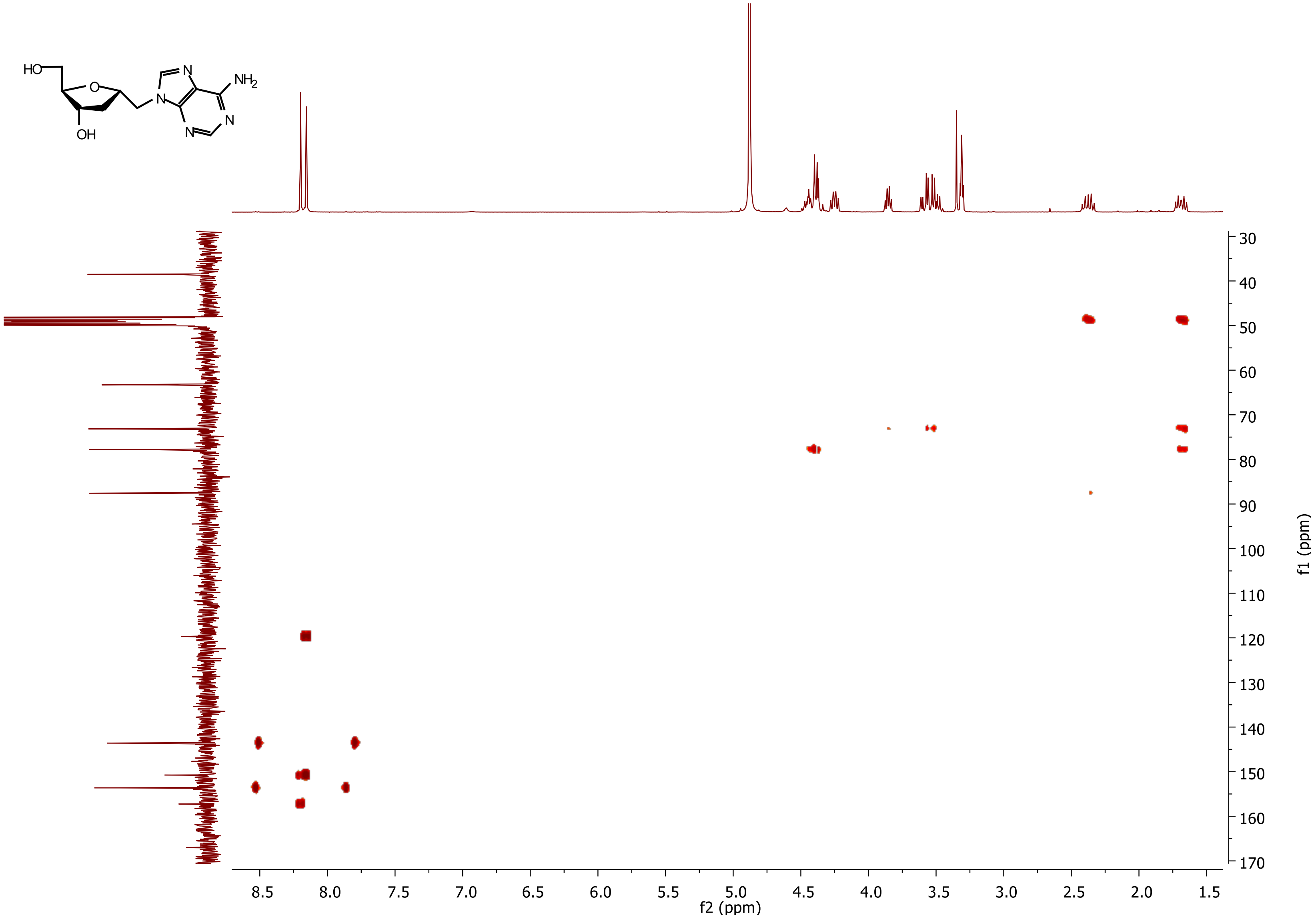

1'-Homo-*N*-2'-deoxy-α-uridine (10b)

^{1}H NMR (300.13 MHz, MeOH-d_4)

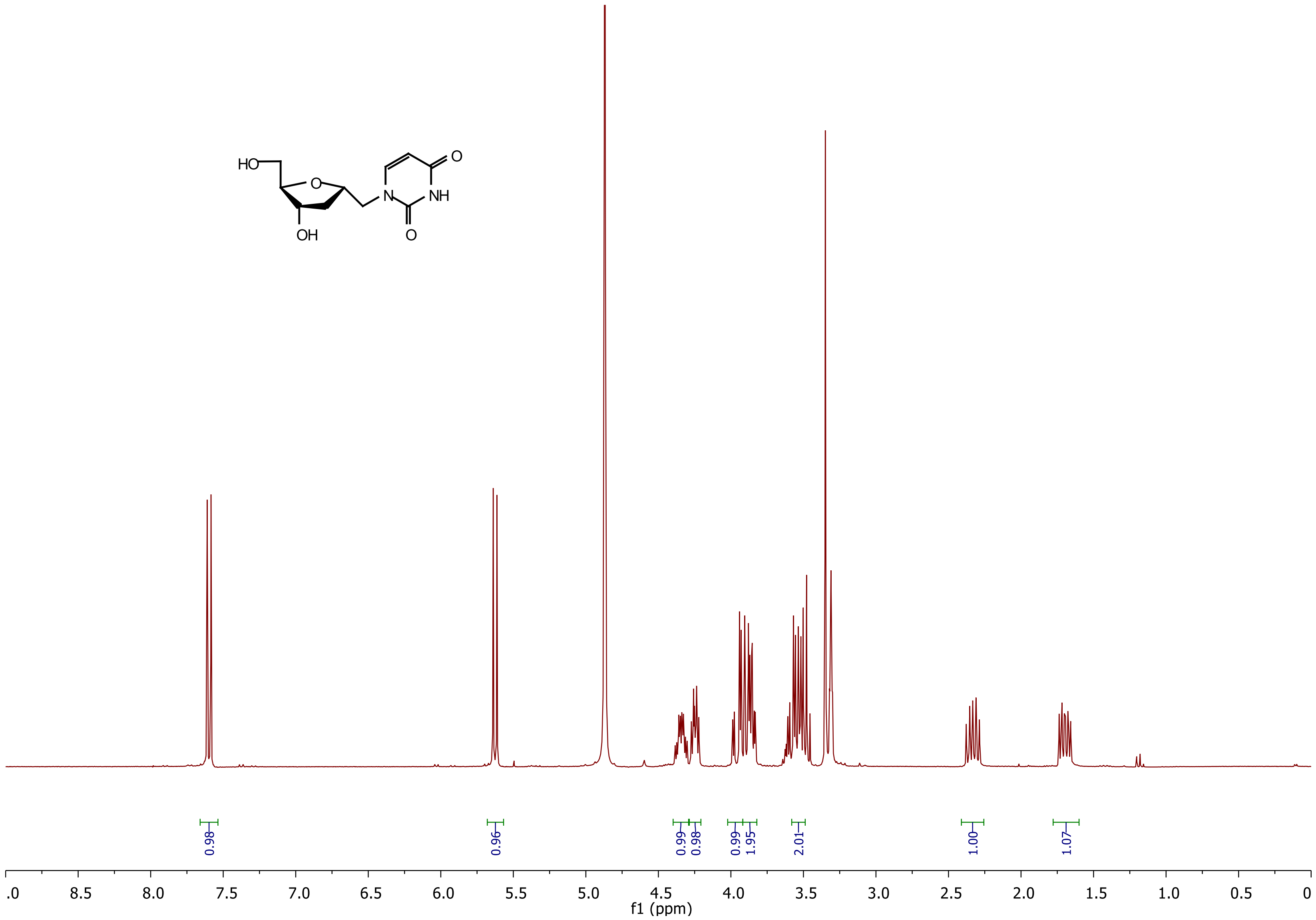

1’-Homo-*N*-2’-deoxy-α-uridine (10b)

^{13}C NMR (75.5 MHz, MeOH-d_4)

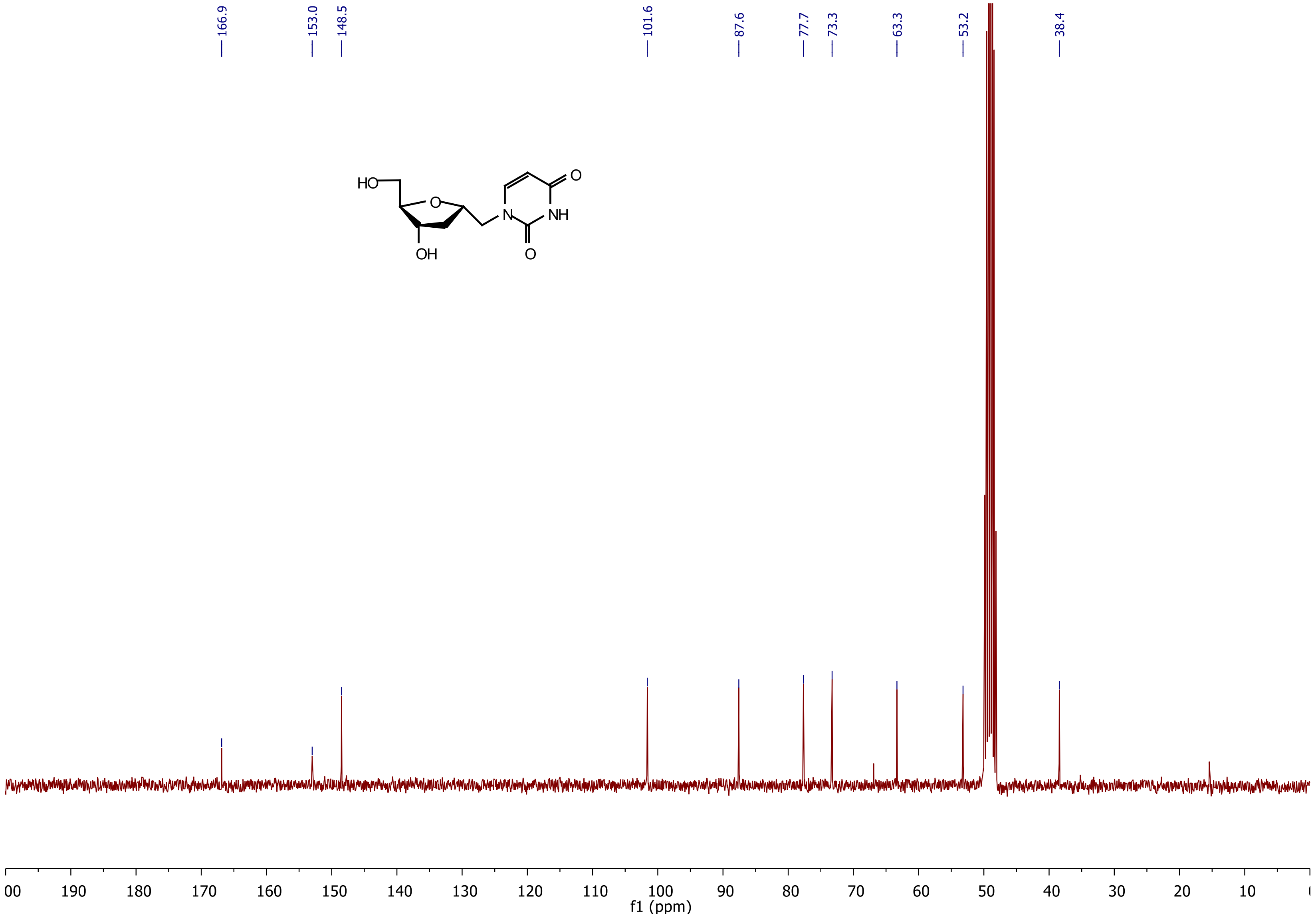

1’-Homo-*N*-2’-deoxy-α-uridine (10b)

DEPT NMR (75.5 MHz, MeOH-d_4)

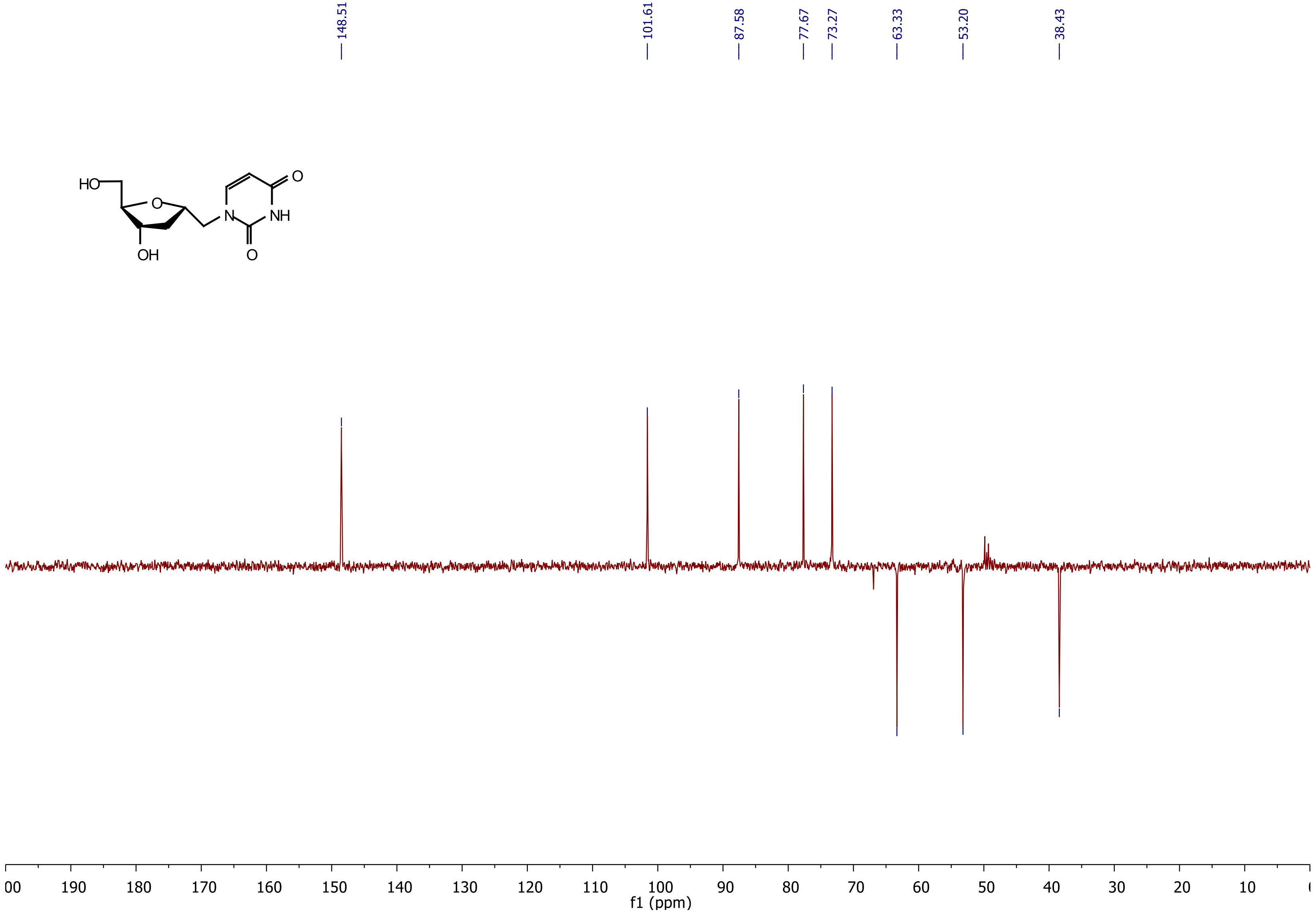

1’-Homo-*N*-2’-deoxy-α-uridine (10b)

COSY NMR (MeOH-d_4)

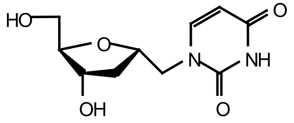

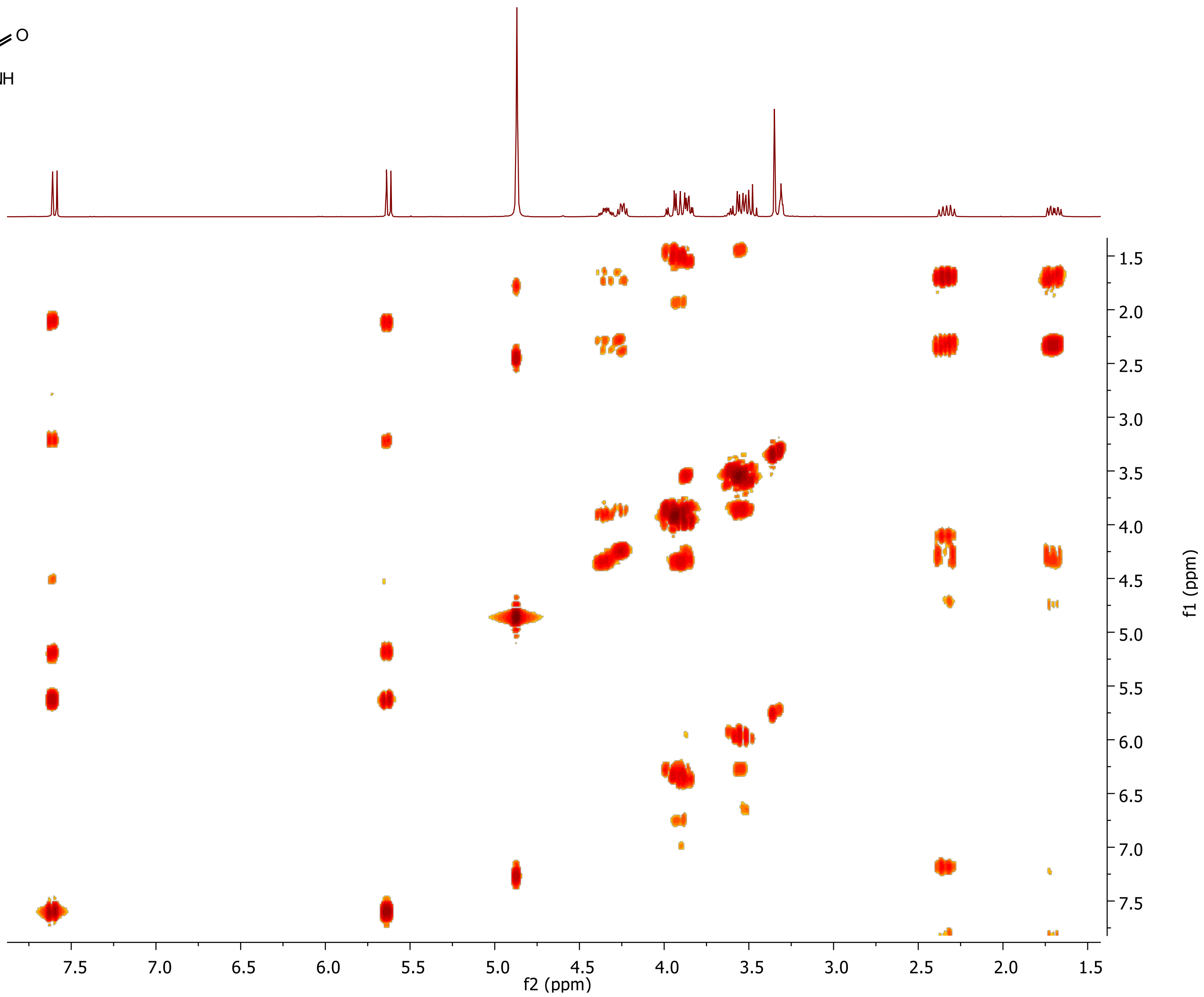

1’-Homo-*N*-2’-deoxy-α-uridine (10b)

HSQC NMR (MeOH-d_4)

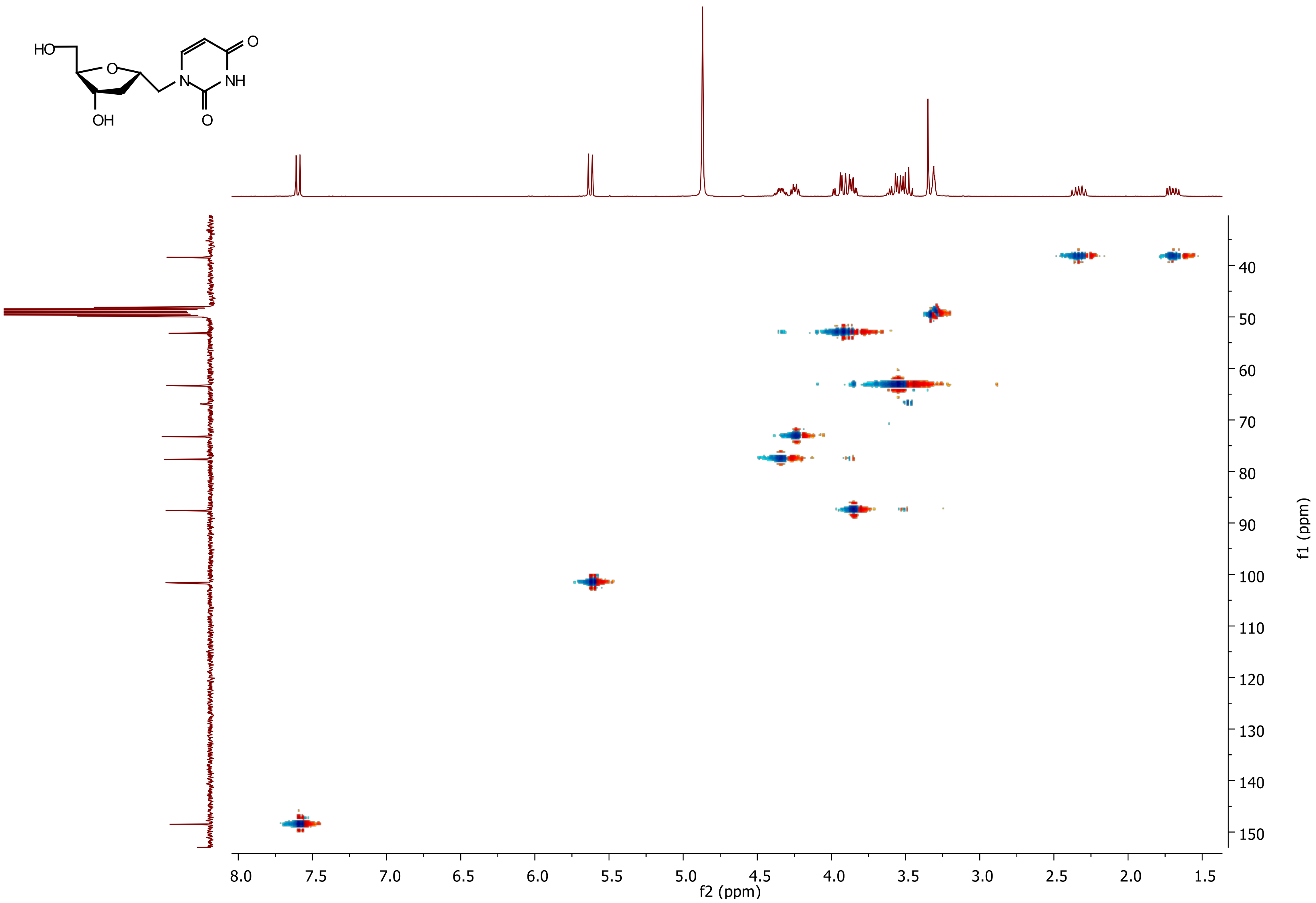

1’-Homo-*N*-2’-deoxy-α-uridine (10b)

HMBC NMR (MeOH-d_4)

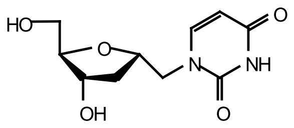

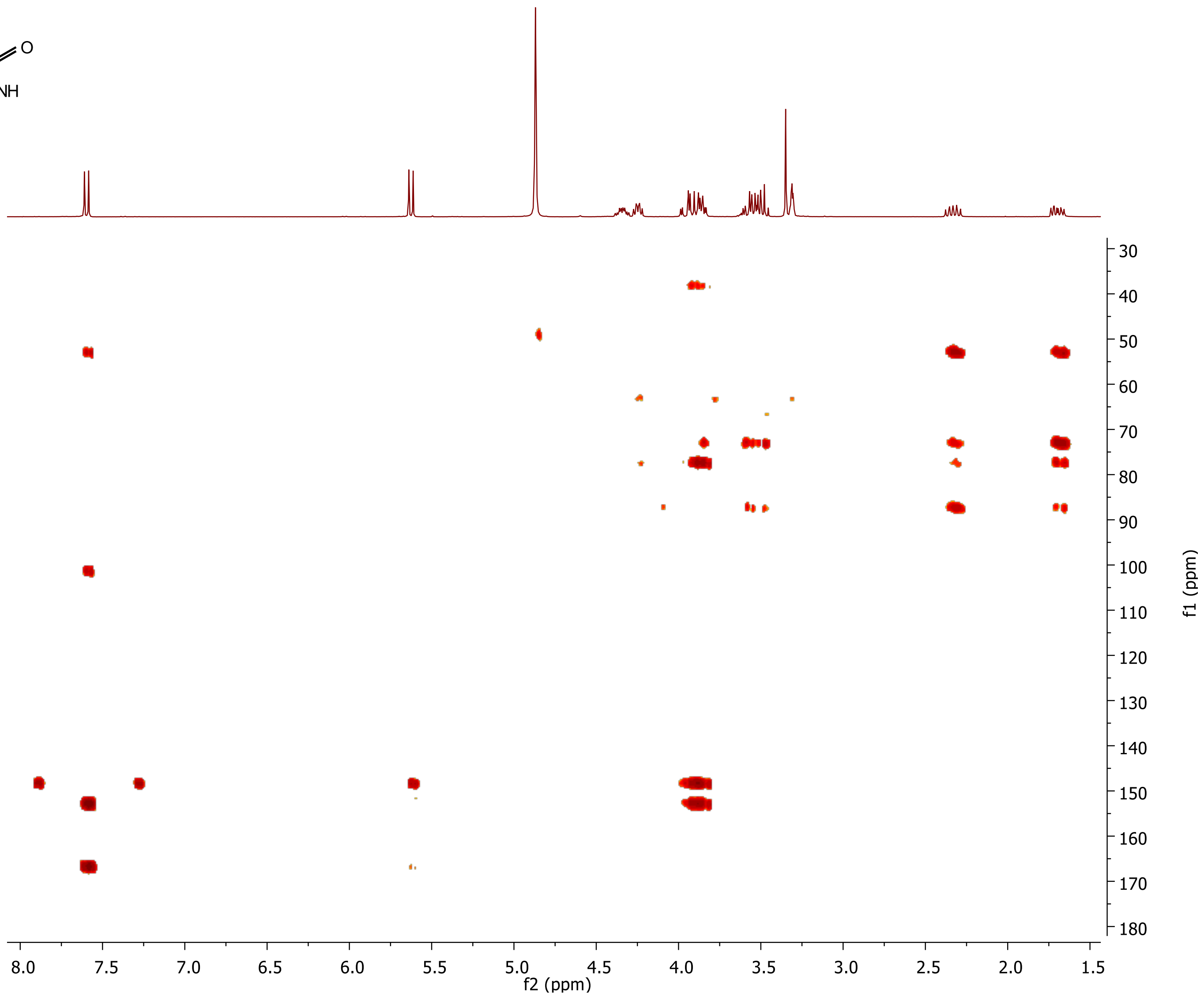

1’-Homo-*N*-α-thymidine (10c)

^{1}H NMR (300.13 MHz, MeOH-d_4)

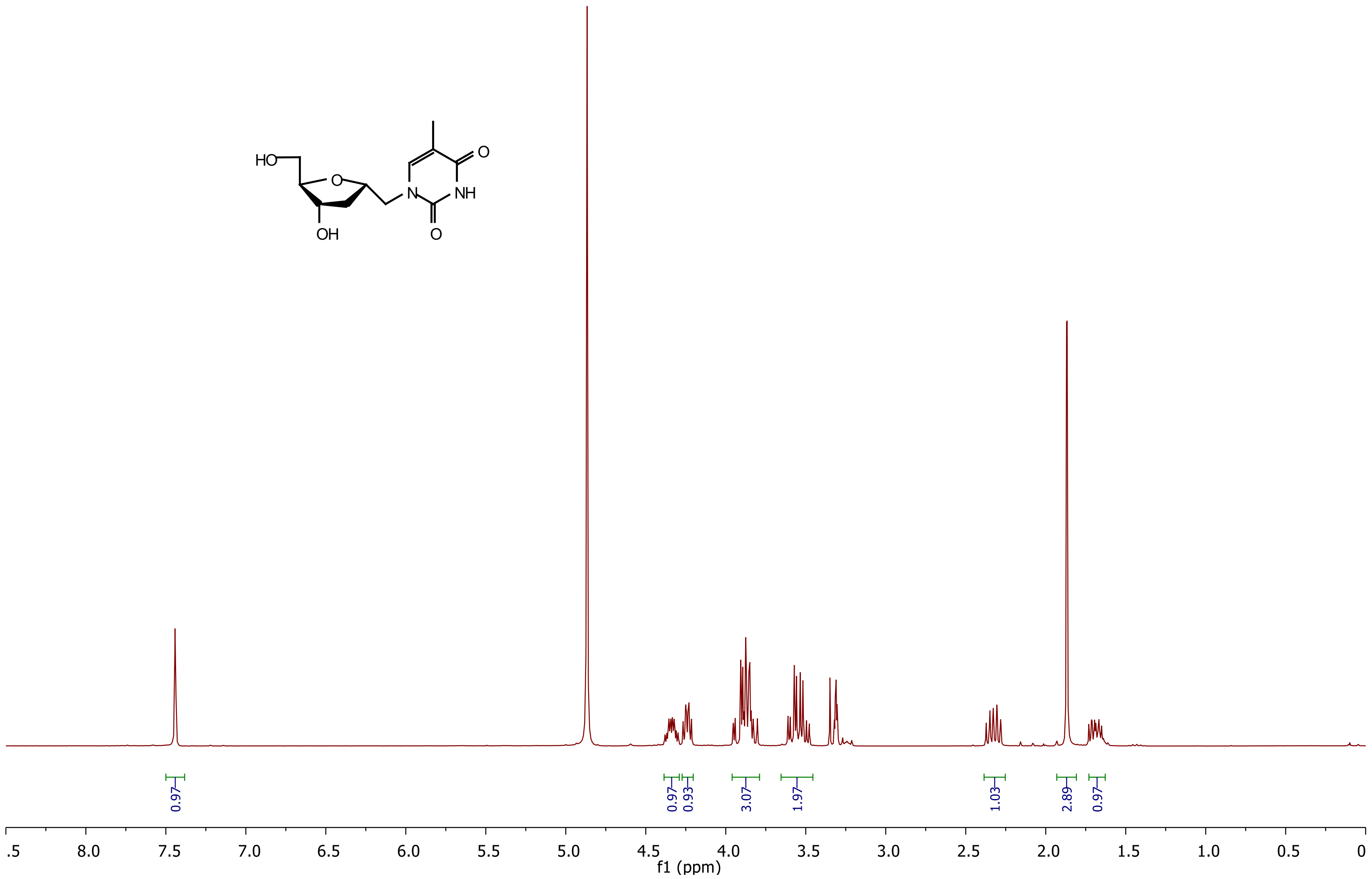

1’-Homo-*N*-α-thymidine (10c)

^{13}C NMR (75.5 MHz, MeOH-d_4)

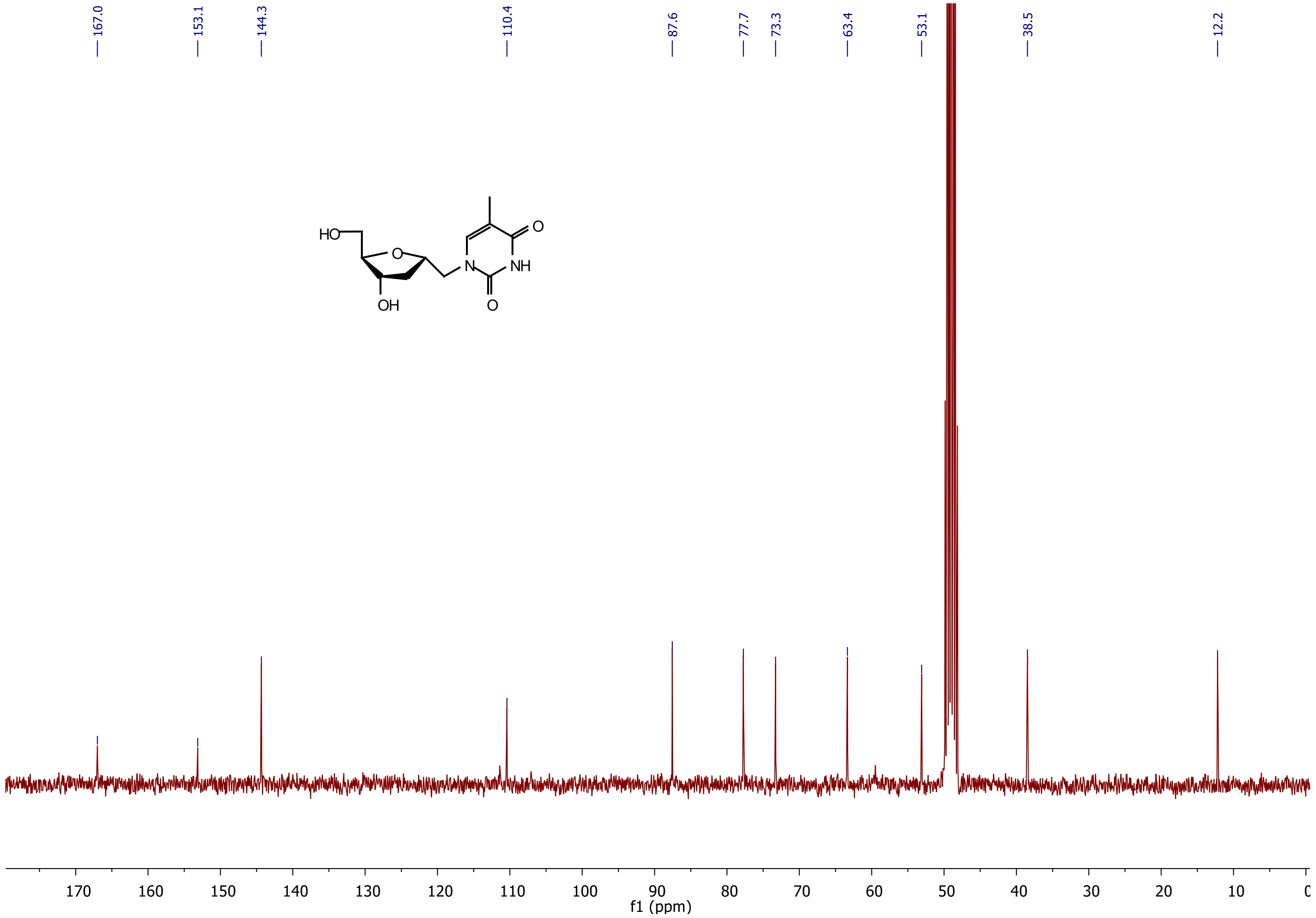

1’-Homo-*N*-α-thymidine (10c)

DEPT NMR (75.5 MHz, MeOH-d_4)

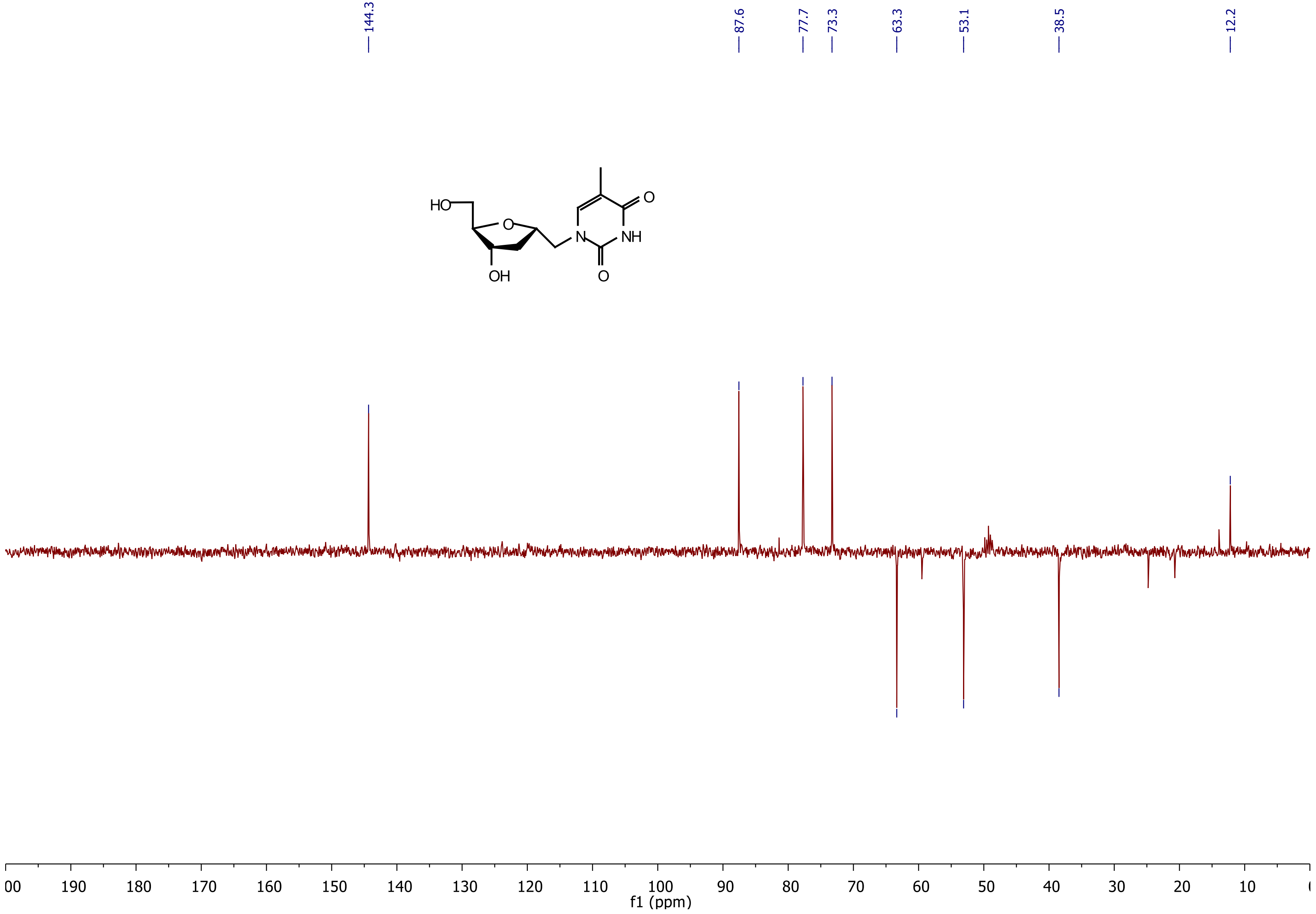

1'-Homo-*N*-α-thymidine (10c)

COSY NMR (MeOH-d_4)

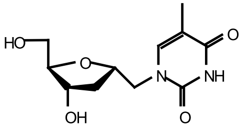

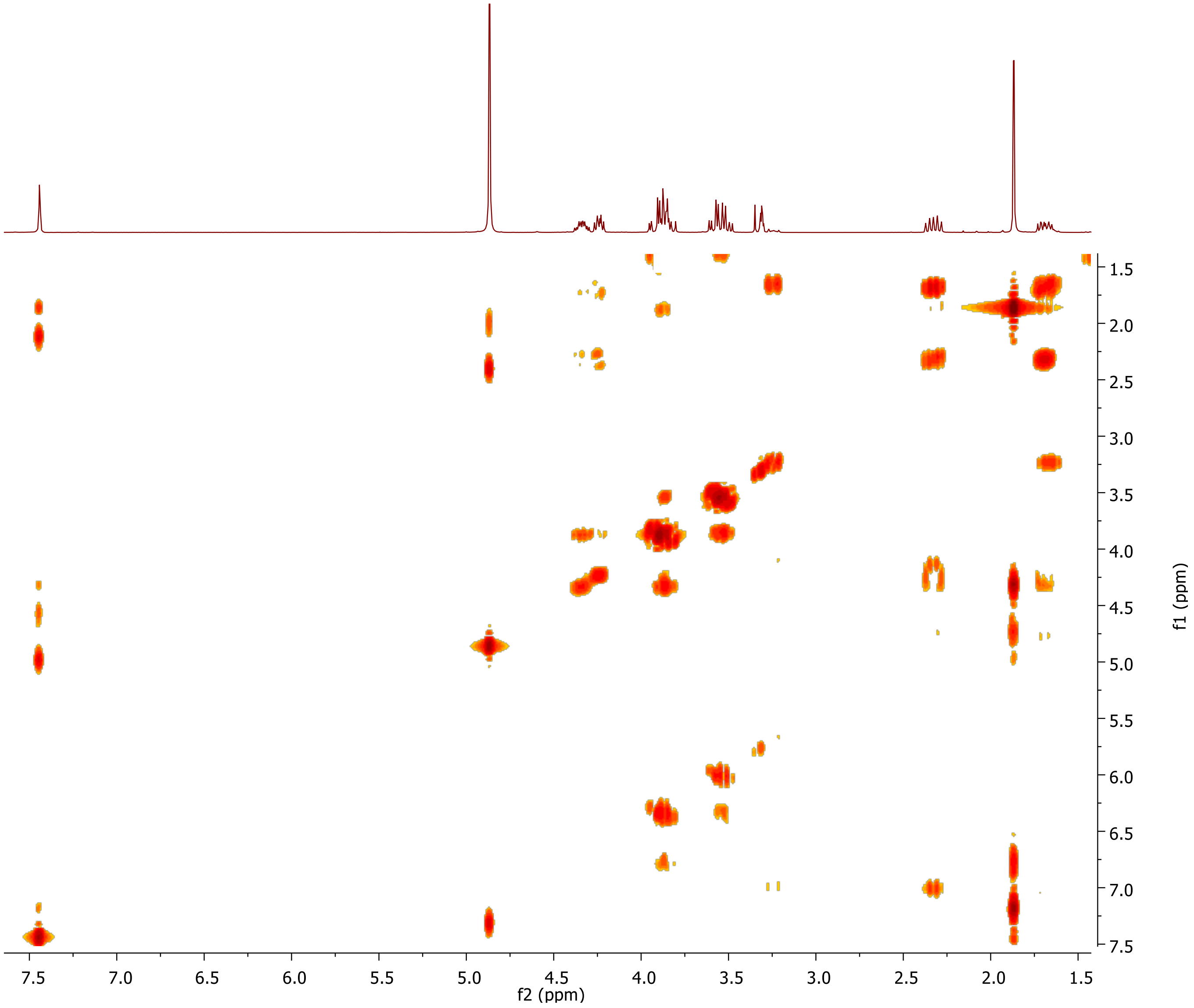

1’-Homo-*N*-α-thymidine (10c)

HSQC NMR (MeOH-d_4)

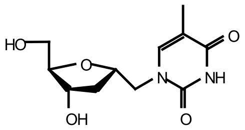

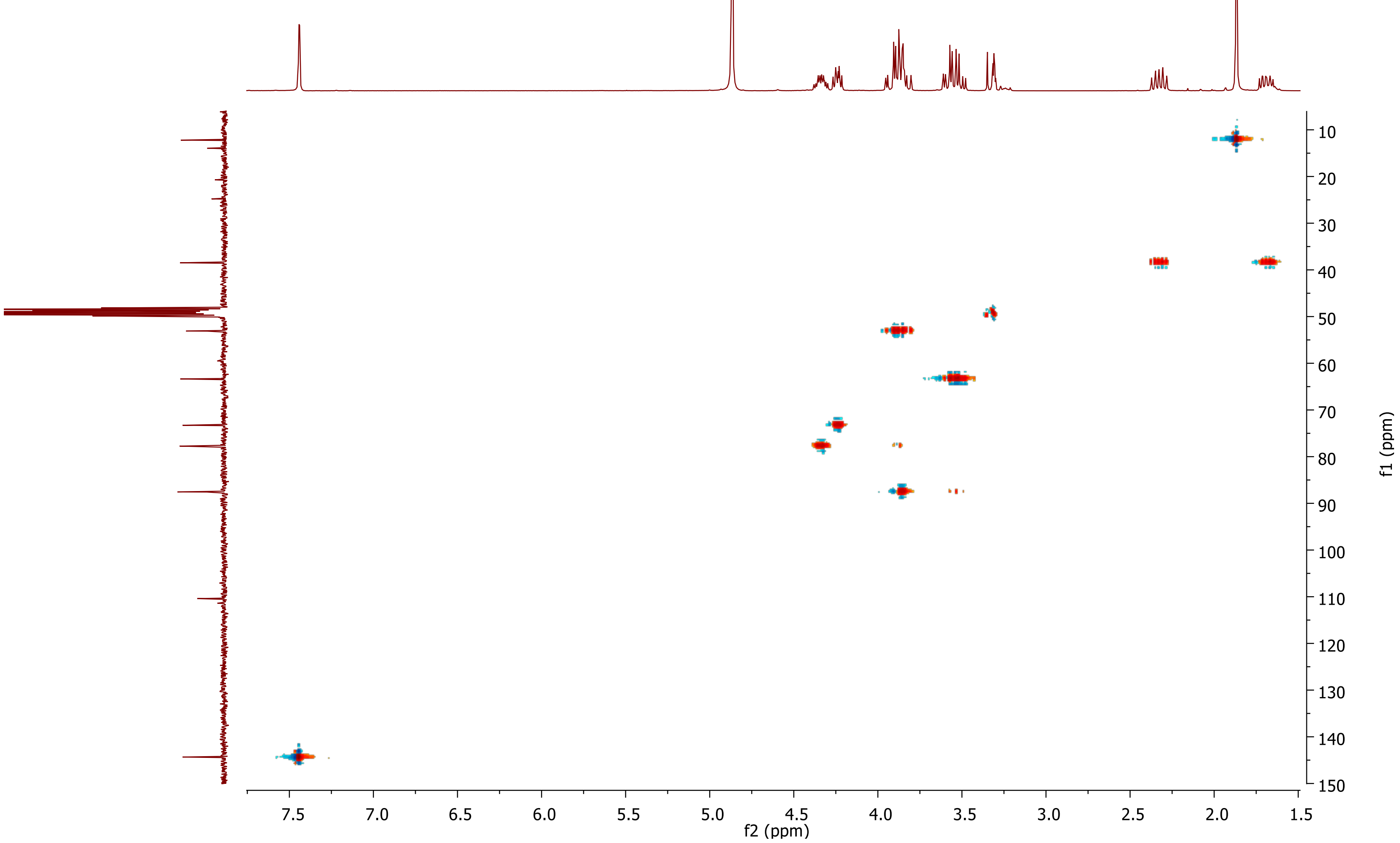

1’-Homo-*N*-α-thymidine (10c)

HMBC NMR (MeOH-d_4)

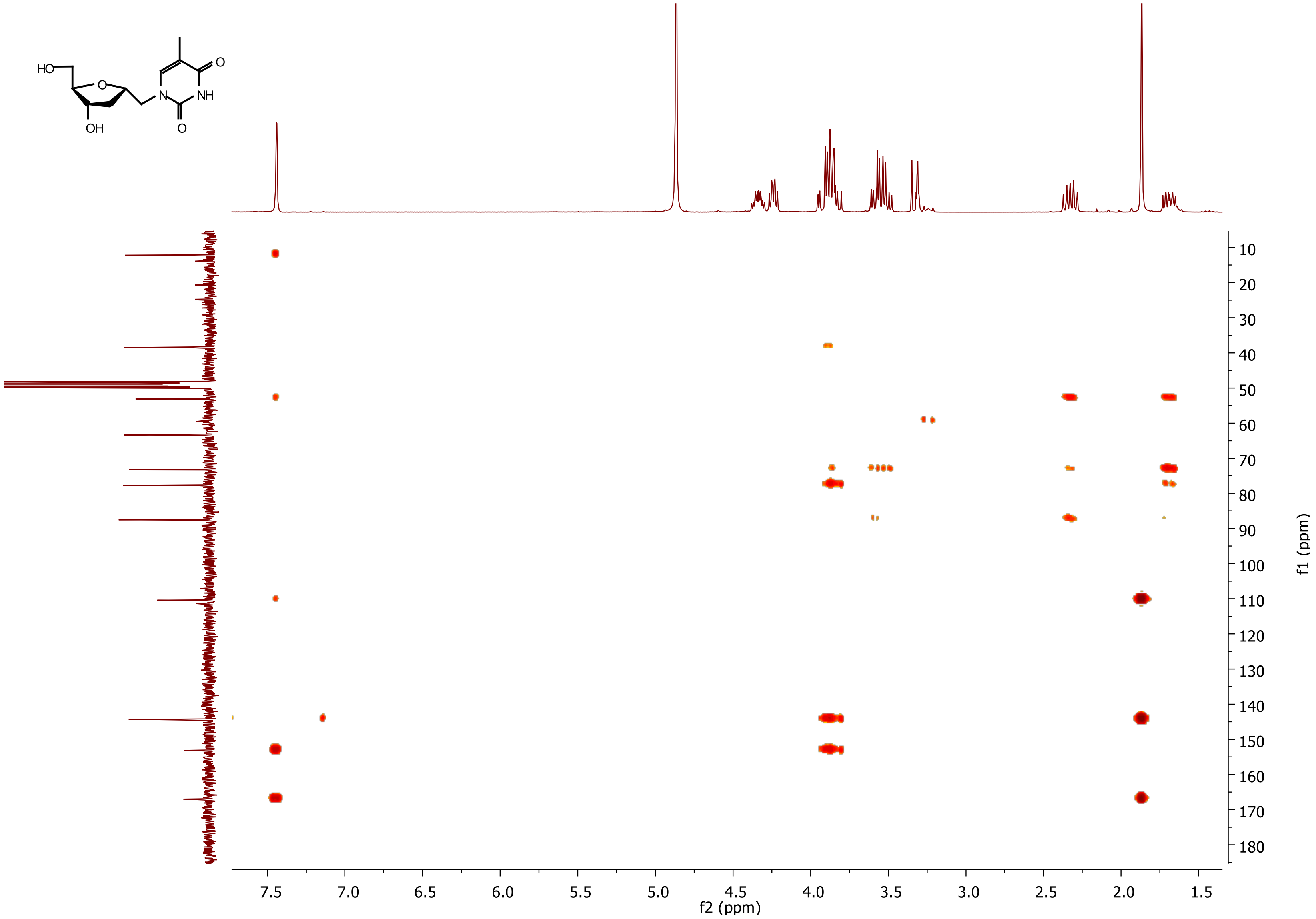

1'-Homo-*N*-2'-deoxy-α-5-fluorouridine (10d)

^{1}H NMR (300.13 MHz, MeOH-d_4)

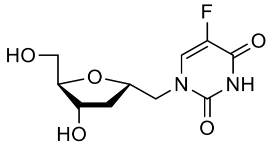

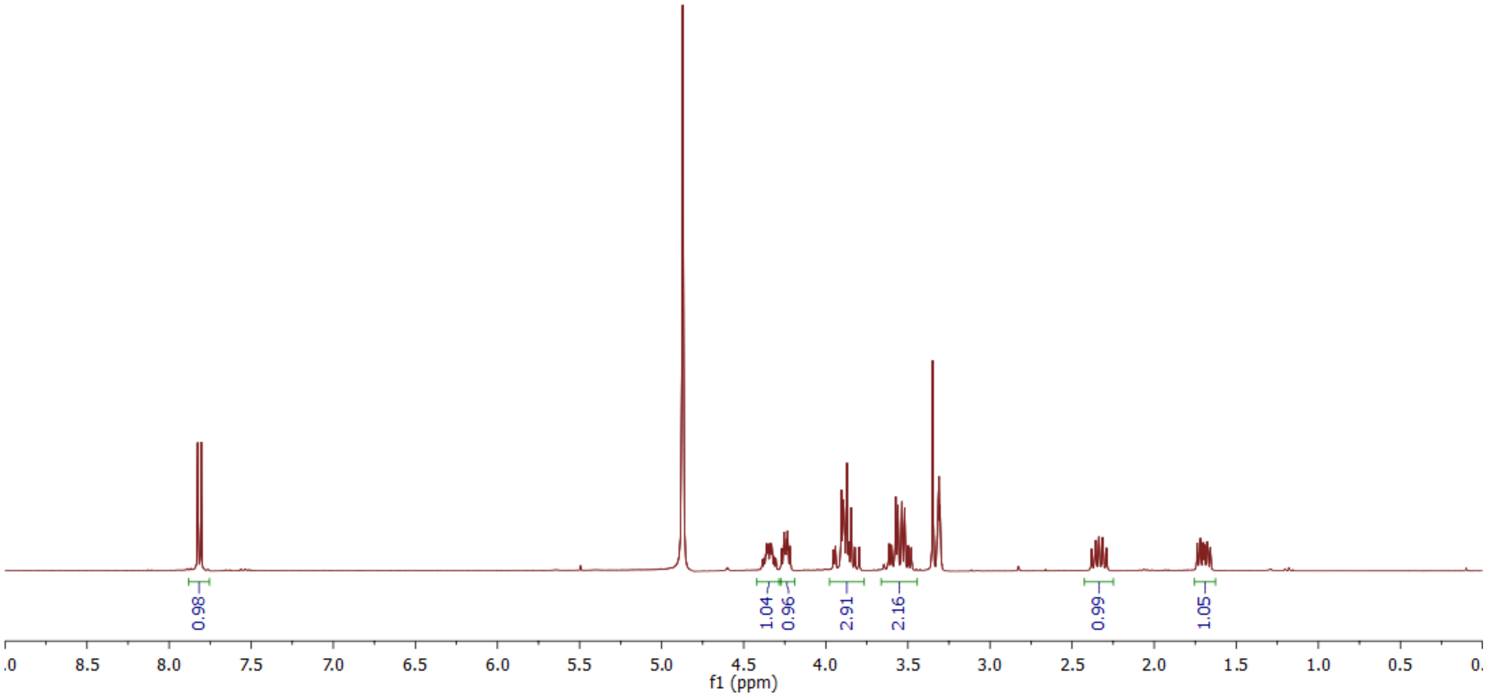

1'-Homo-*N*-2'-deoxy-α-5-fluorouridine (10d)

^{13}C NMR (75.5 MHz, MeOH-d_4)

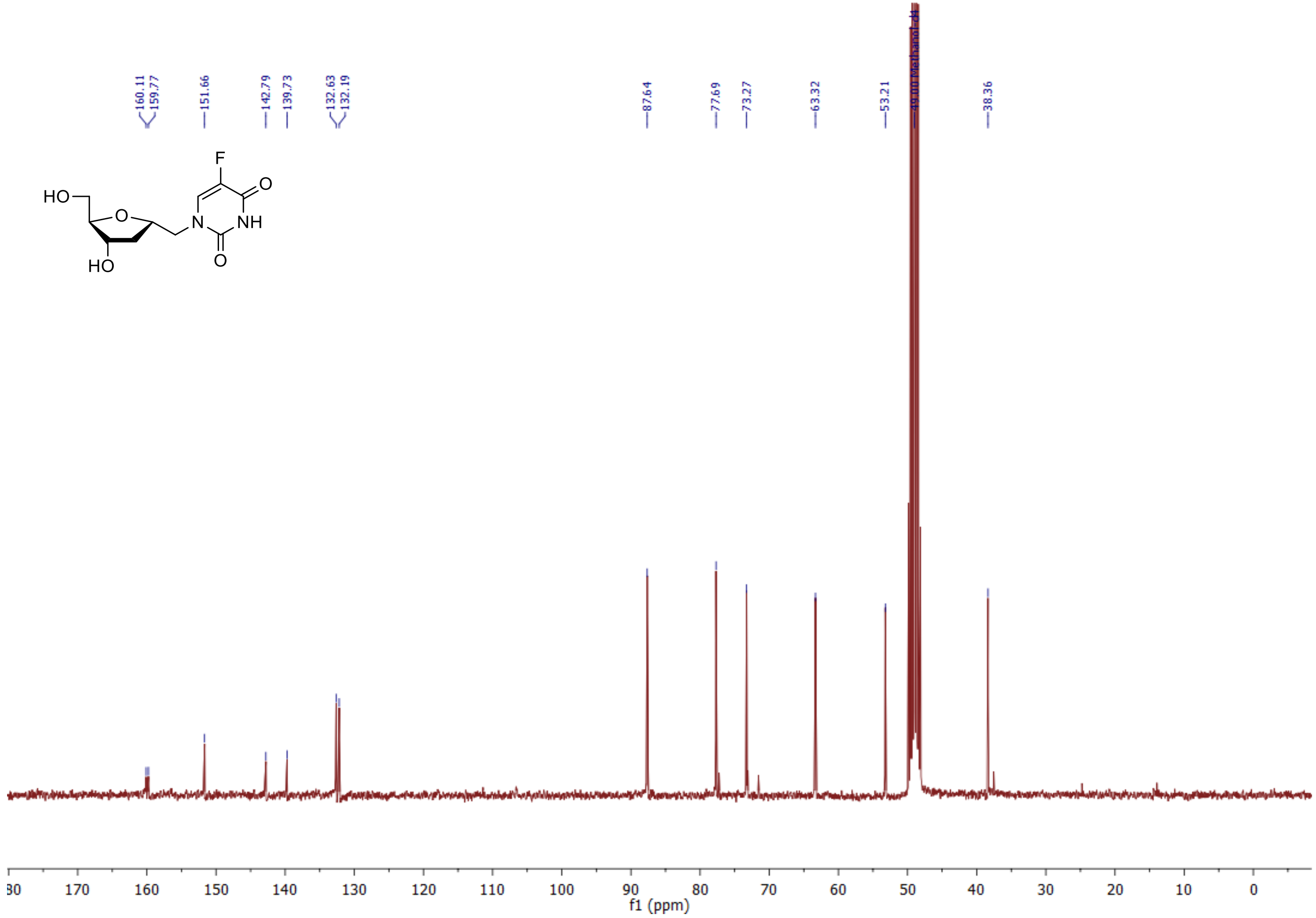

1'-Homo-*N*-2'-deoxy-α-5-fluorouridine (10d)

DEPT 135 NMR (75.5 MHz, MeOH-d_4)

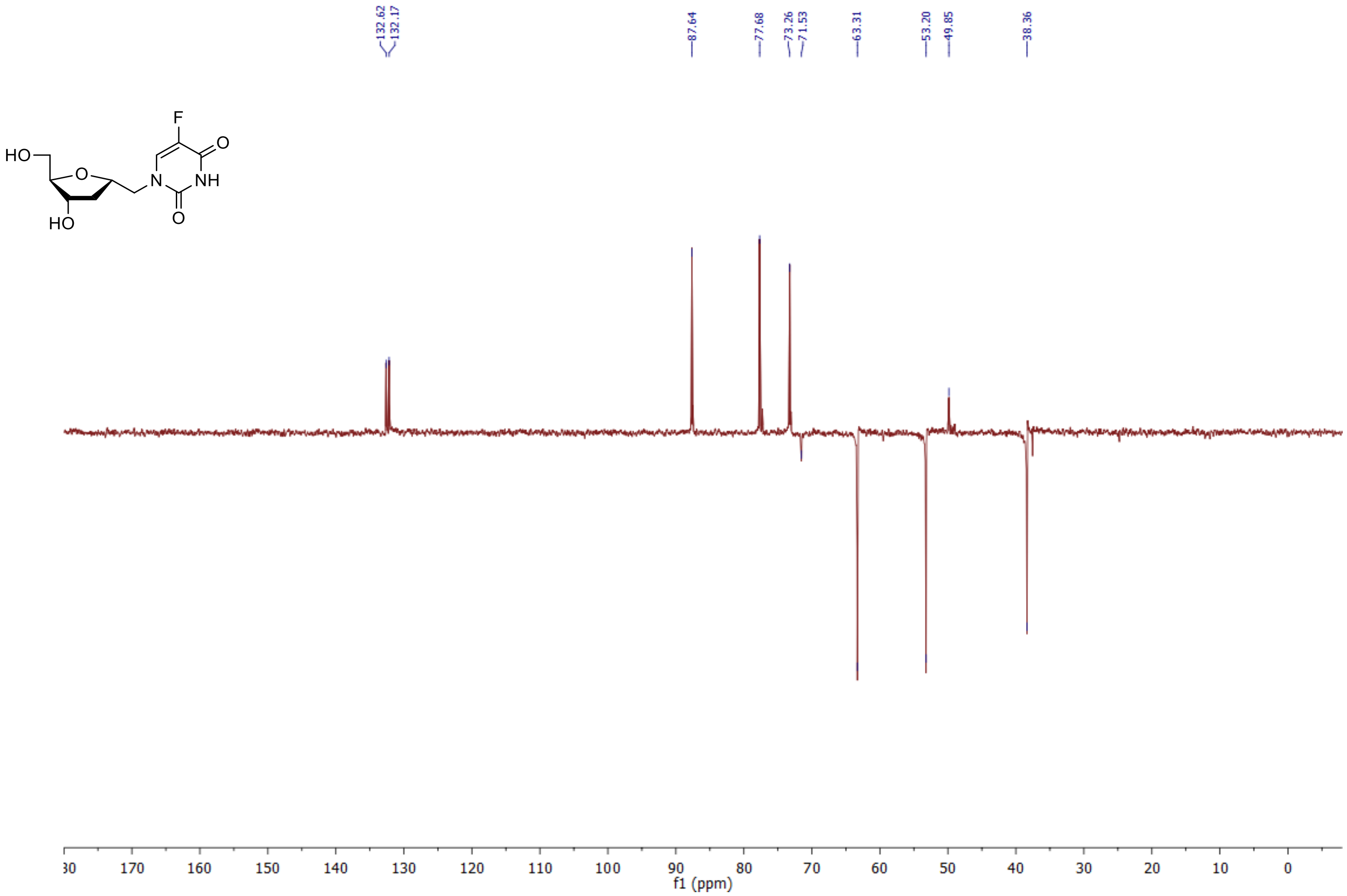

1’-Homo-*N*-2’-deoxy-α-5-fluorouridine (10d)

COSY NMR (MeOH-d_4)

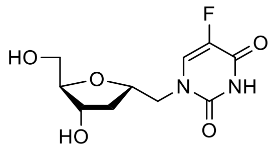

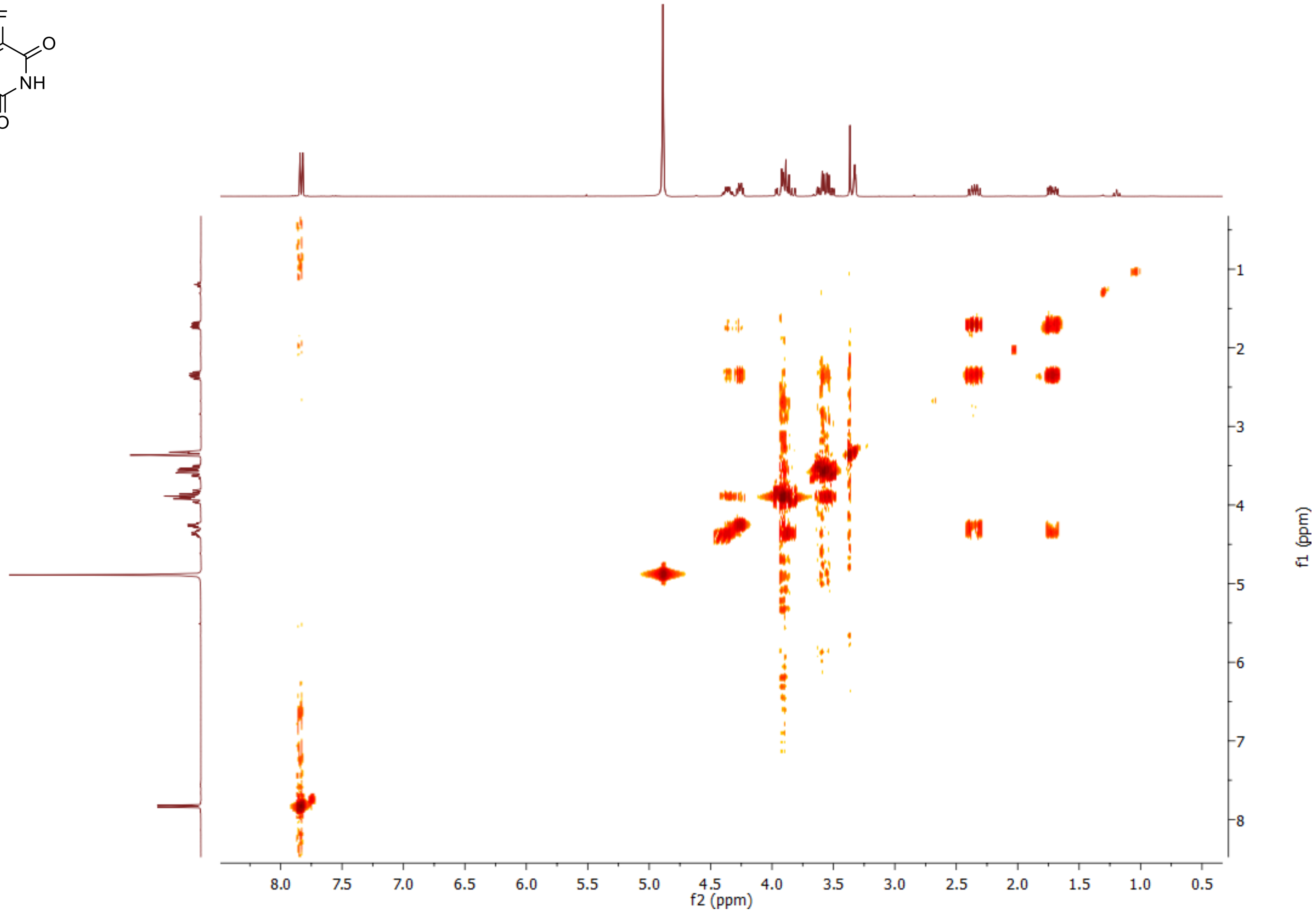

1’-Homo-*N*-2’-deoxy-α-5-fluorouridine (10d)

HSQC NMR (MeOH-d_4)

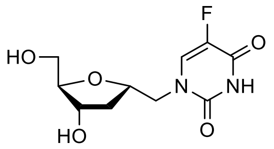

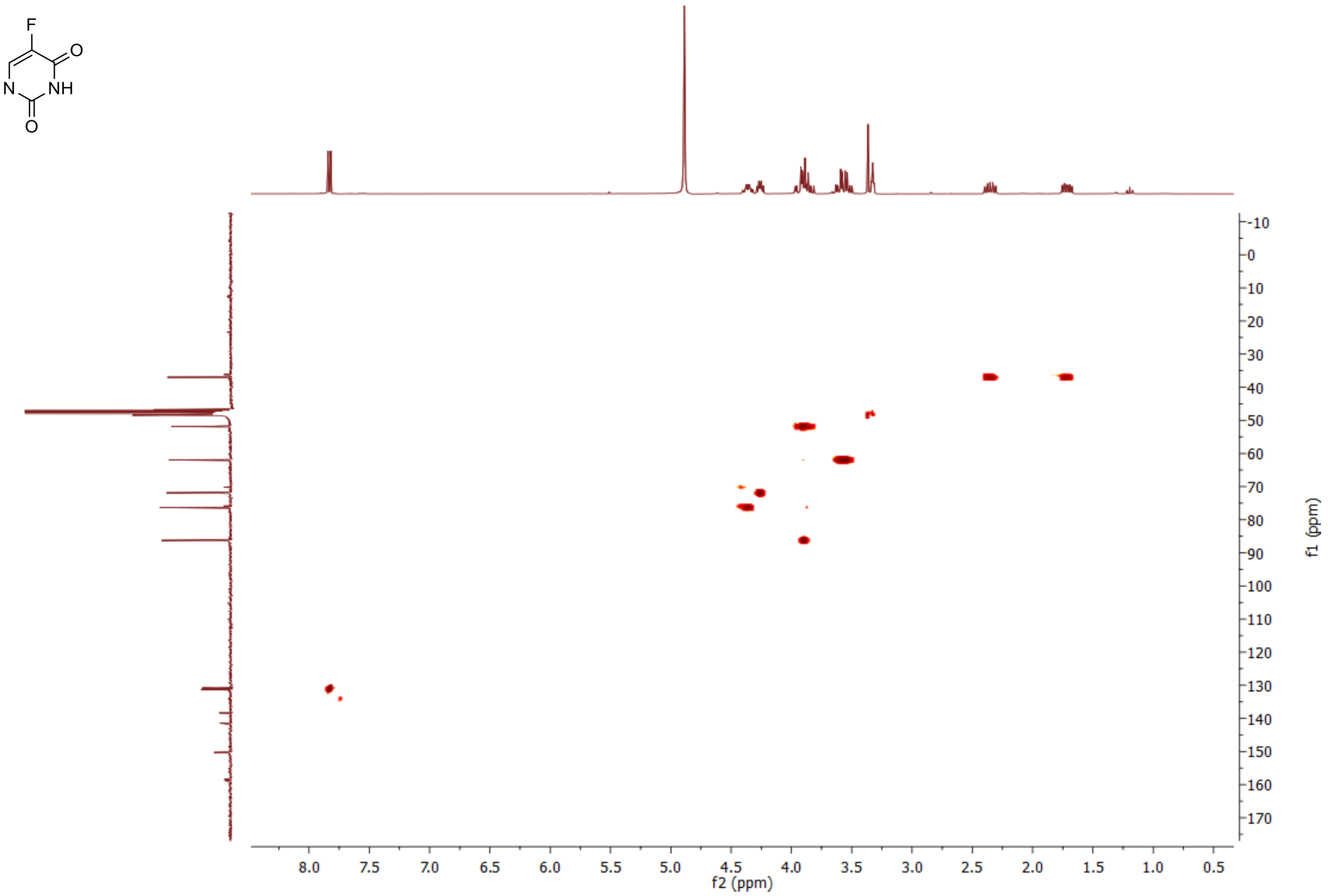

HMBC NMR (MeOH-d_4)

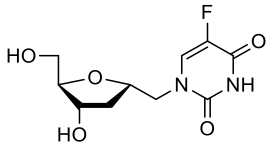

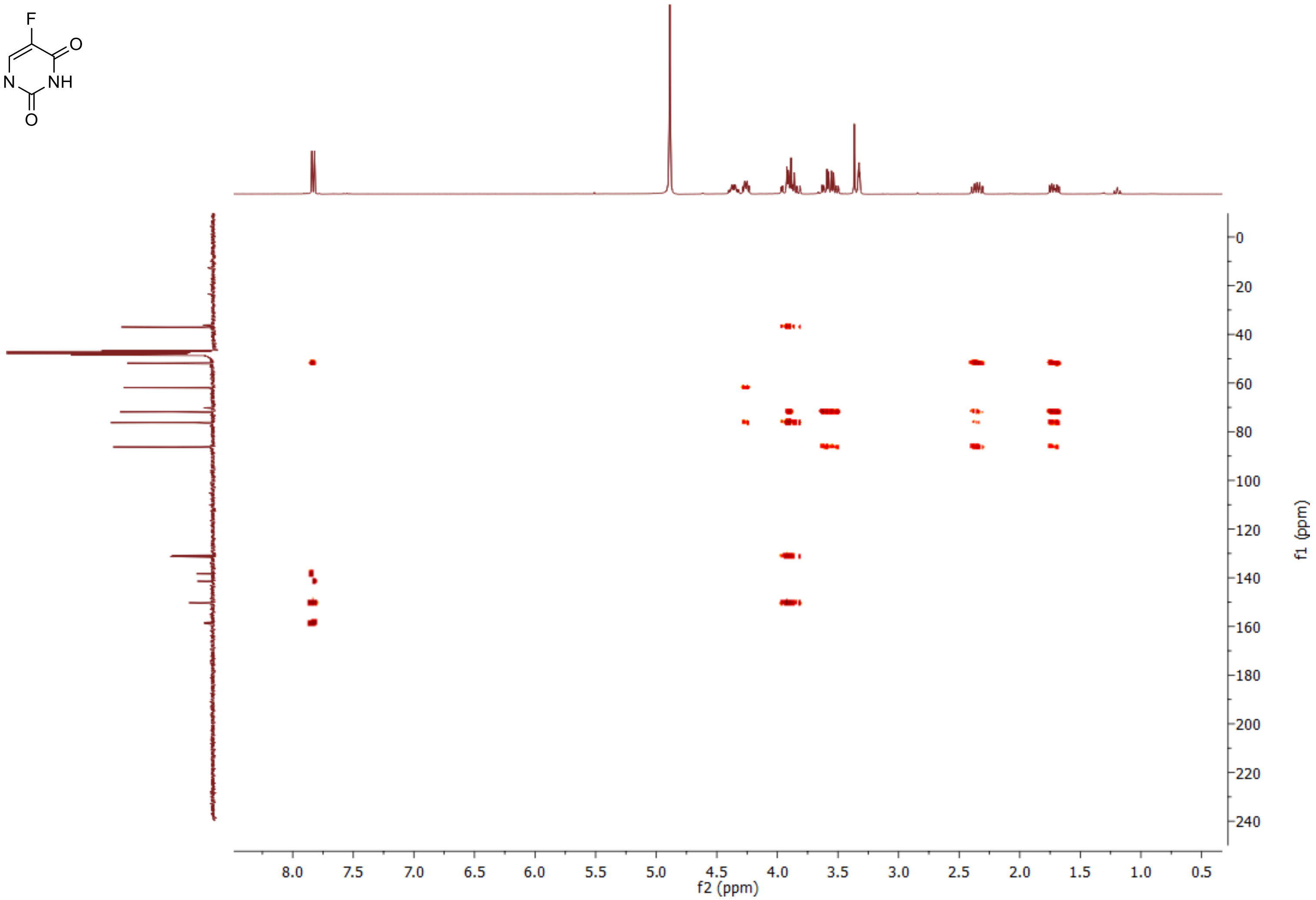

1’-Homo-*N*-2’-deoxy-α-cytidine (10e)

^{1}H NMR (300.13 MHz, MeOH-d_4)

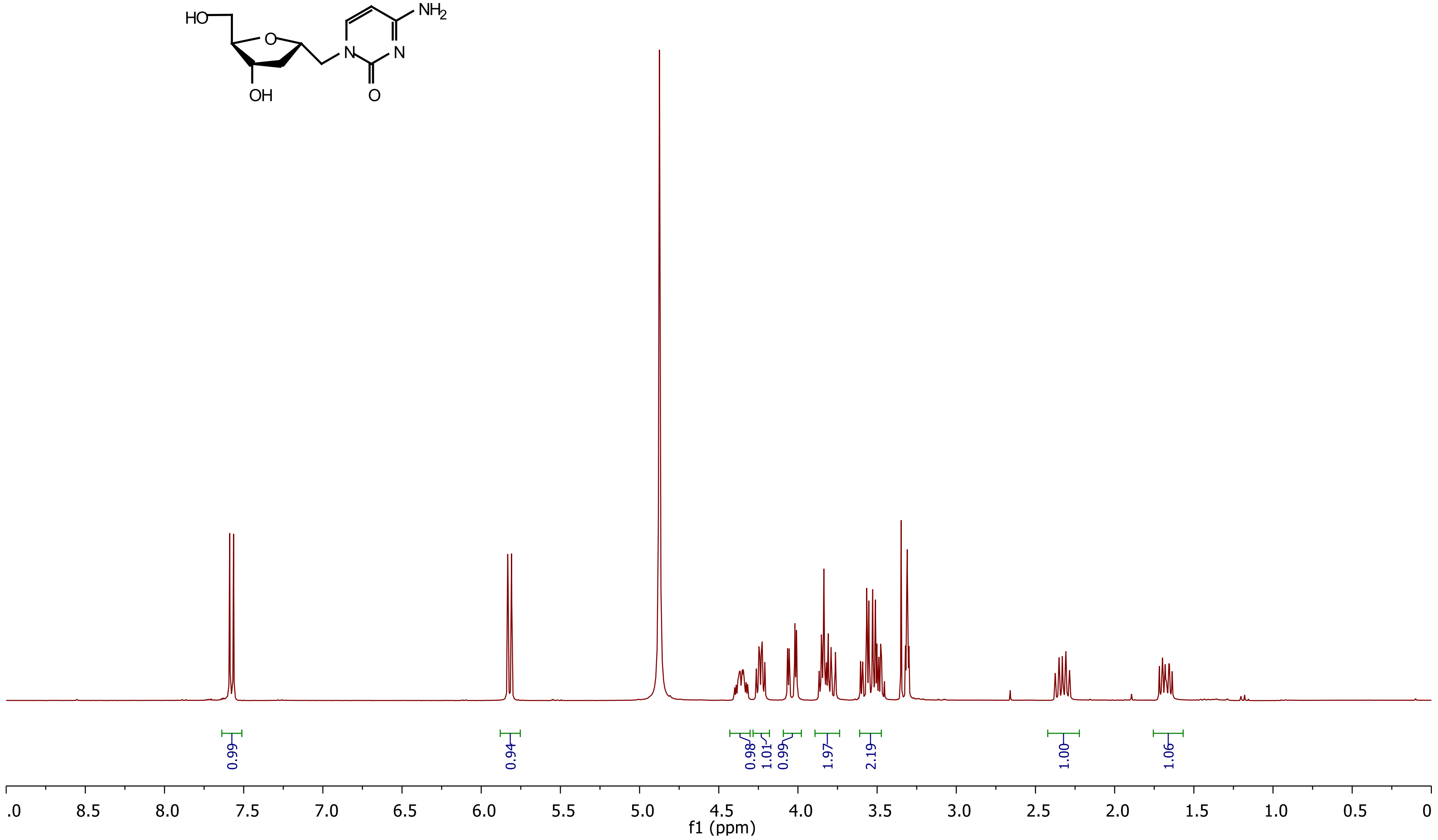

1’-Homo-*N*-2’-deoxy-α-cytidine (10e)

^{13}C NMR (75.5 MHz, MeOH-d_4)

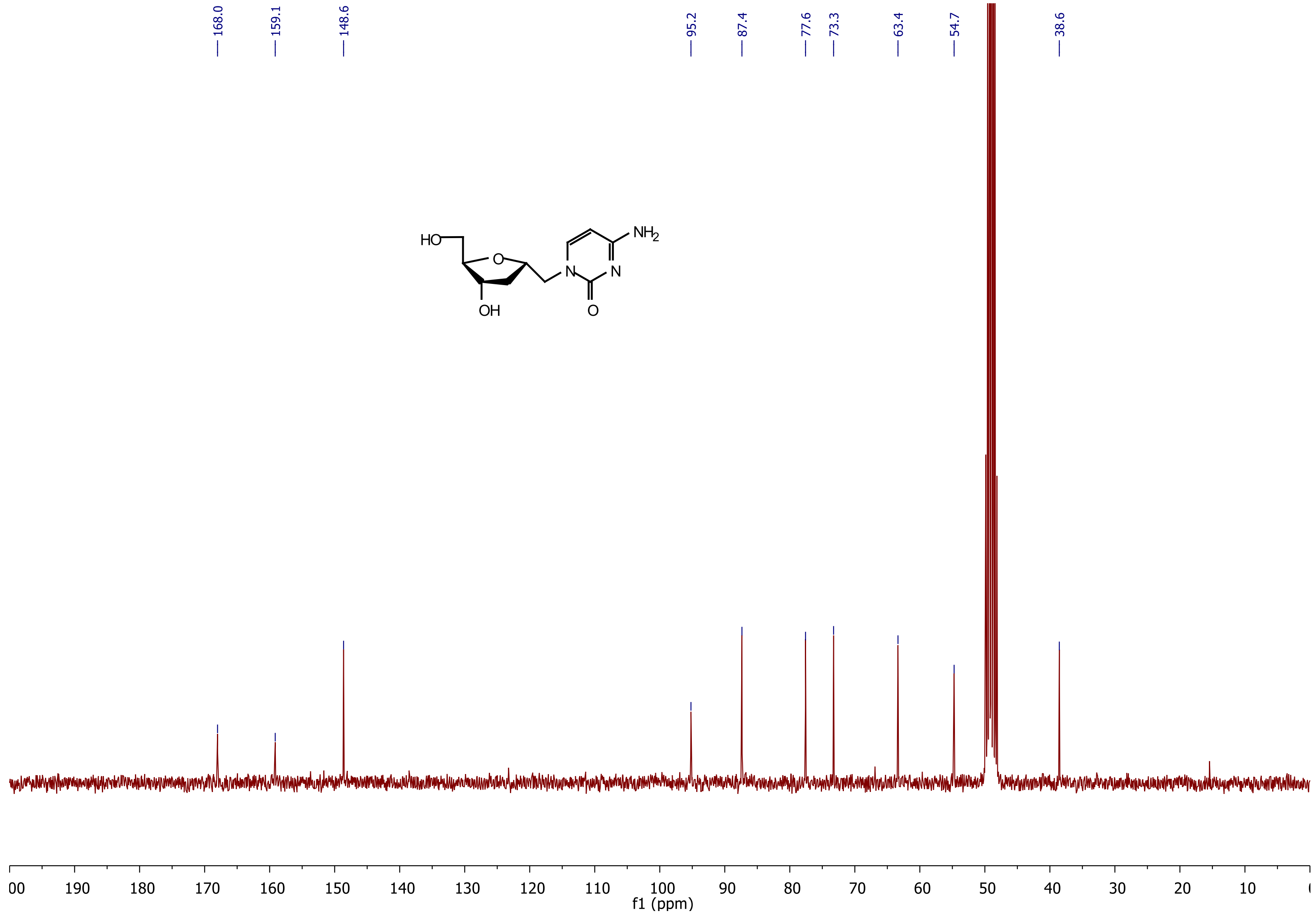

1’-Homo-*N*-2’-deoxy-α-cytidine (10e)

DEPT NMR (75.5 MHz, MeOH-d_4)

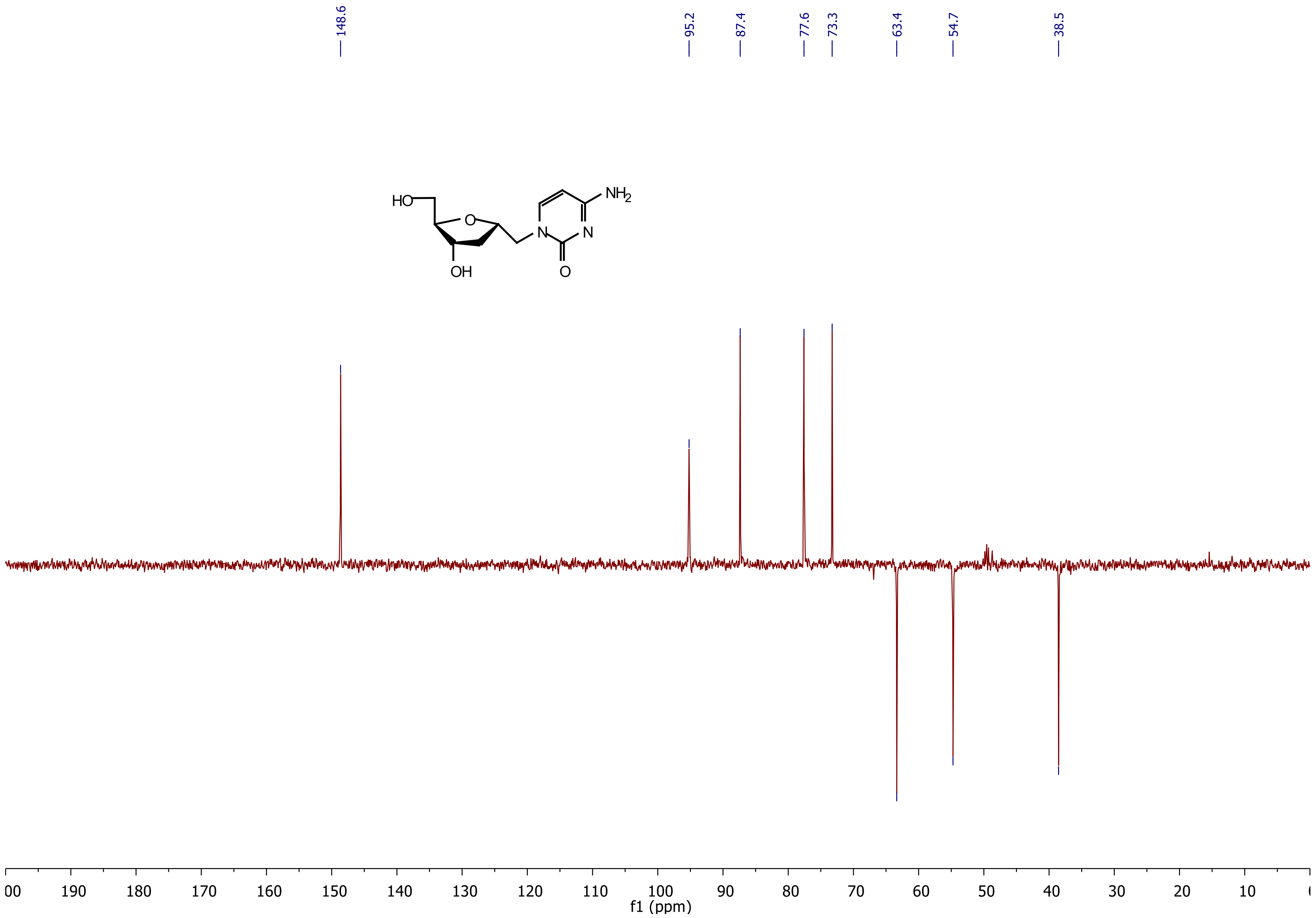

COSY NMR (MeOH-d_4)

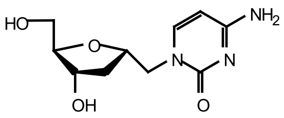

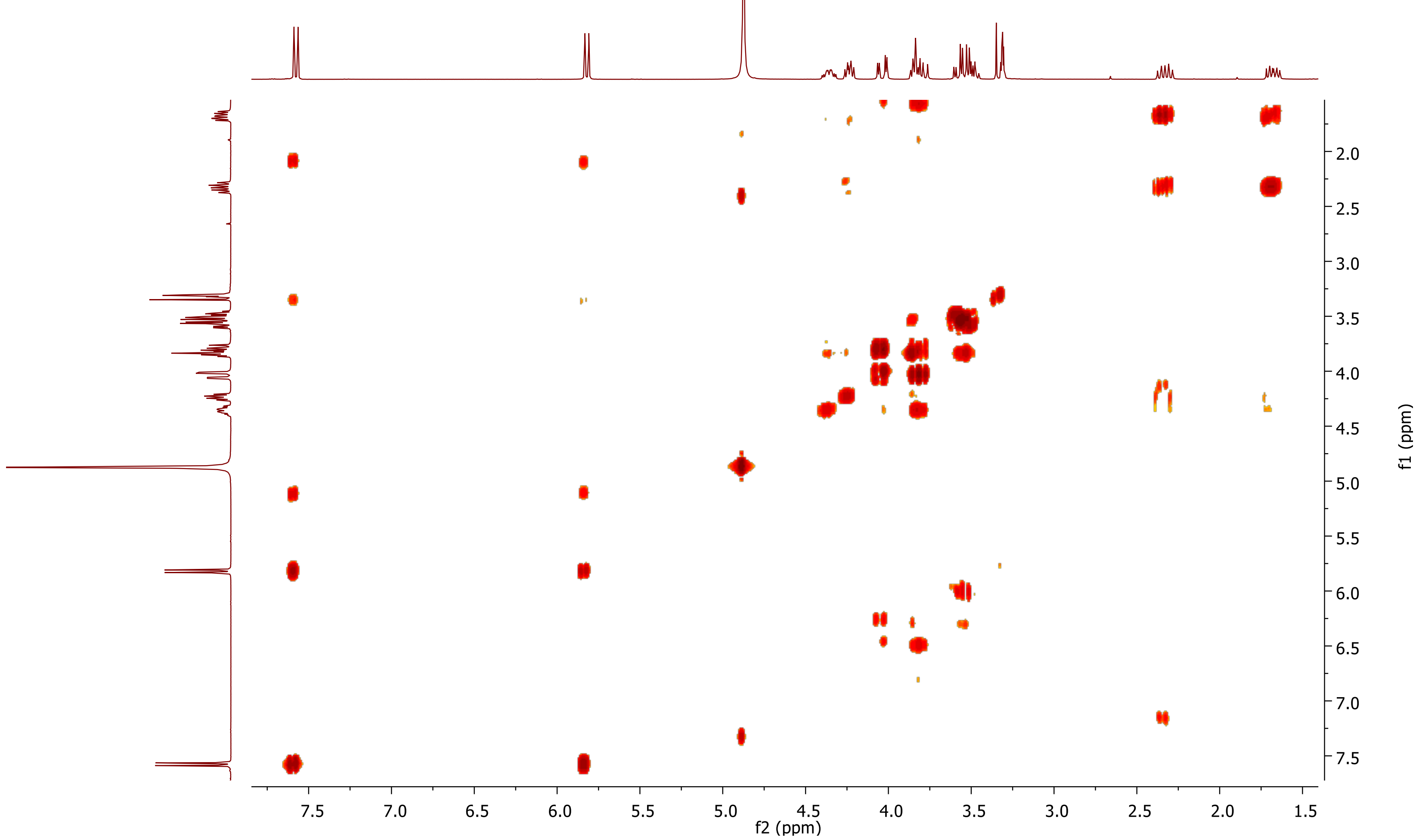

1’-Homo-*N*-2’-deoxy-α-cytidine (10e)

HSQC NMR (MeOH-d_4)

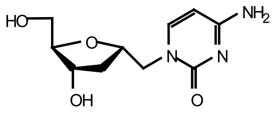

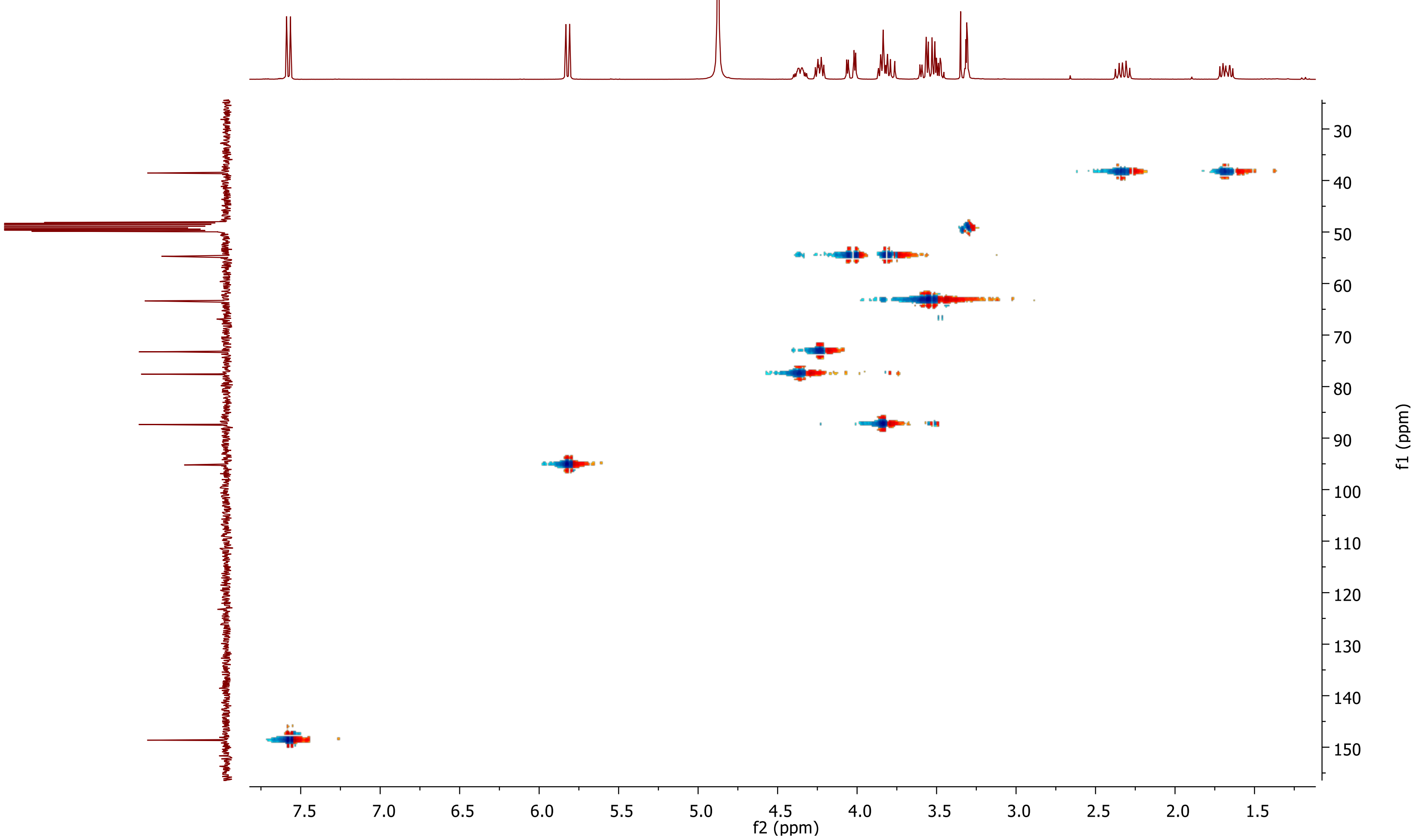

1’-Homo-*N*-2’-deoxy-α-cytidine (10e)

HMBC NMR (MeOH-d_4)

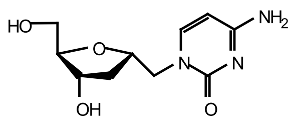

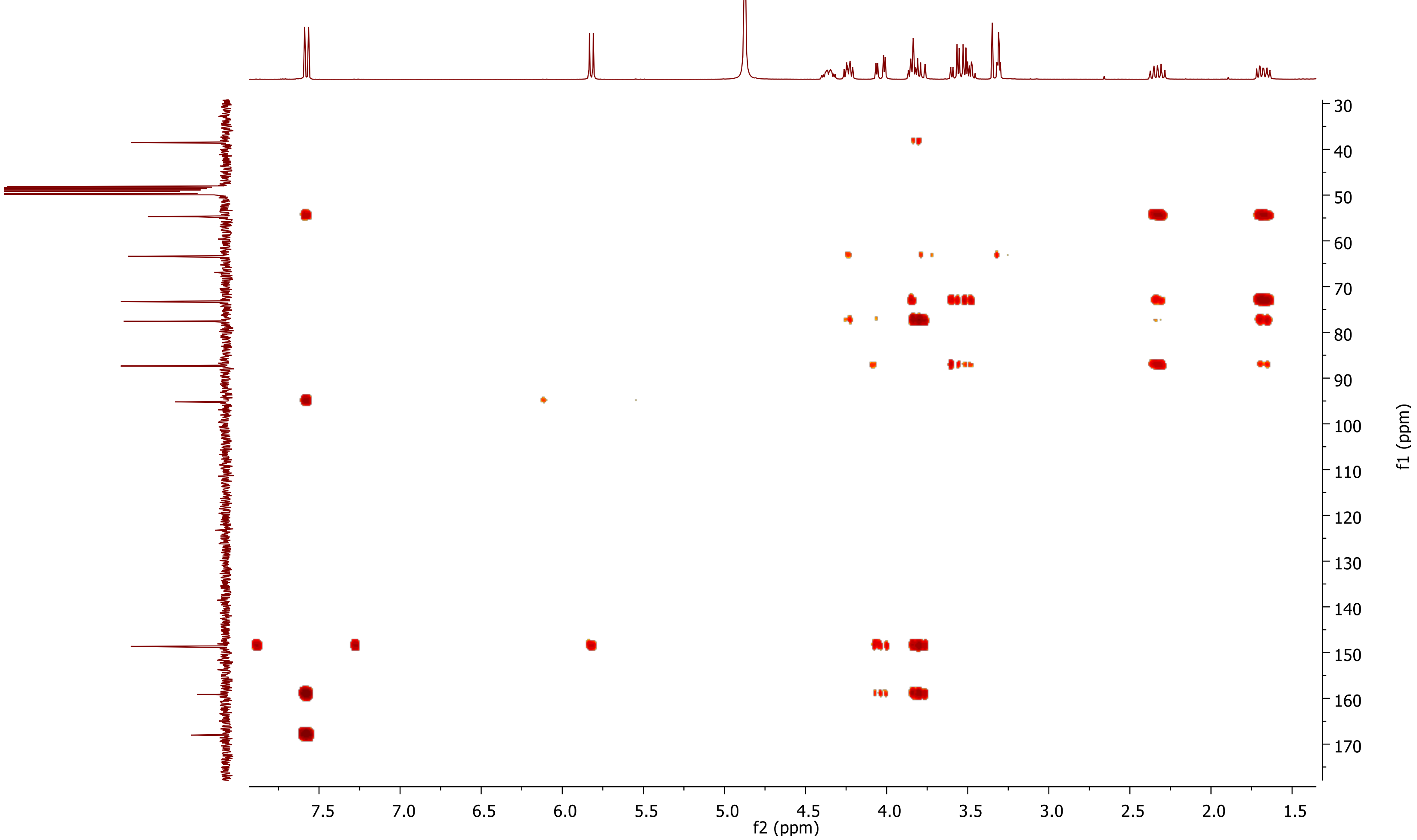

1’-Homo-*N*-2’-deoxy-α-5-iodouridine (10f)

^{1}H NMR (300.13 MHz, MeOH-d_4)

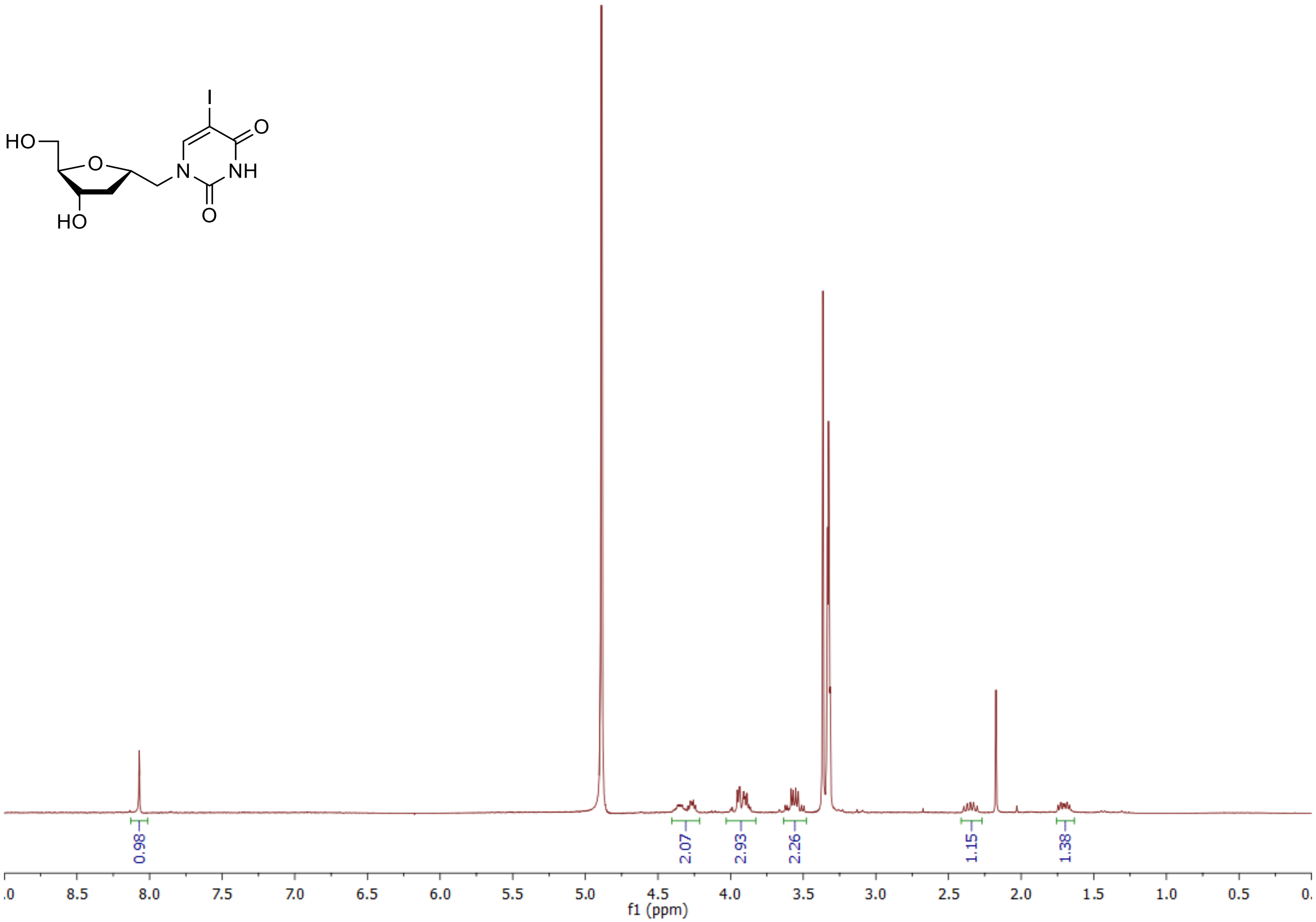

1'-Homo-*N*-2'-deoxy-α-5-iodouridine (10f)

COSY NMR (MeOH-d_4)

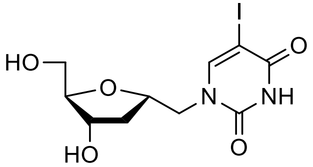

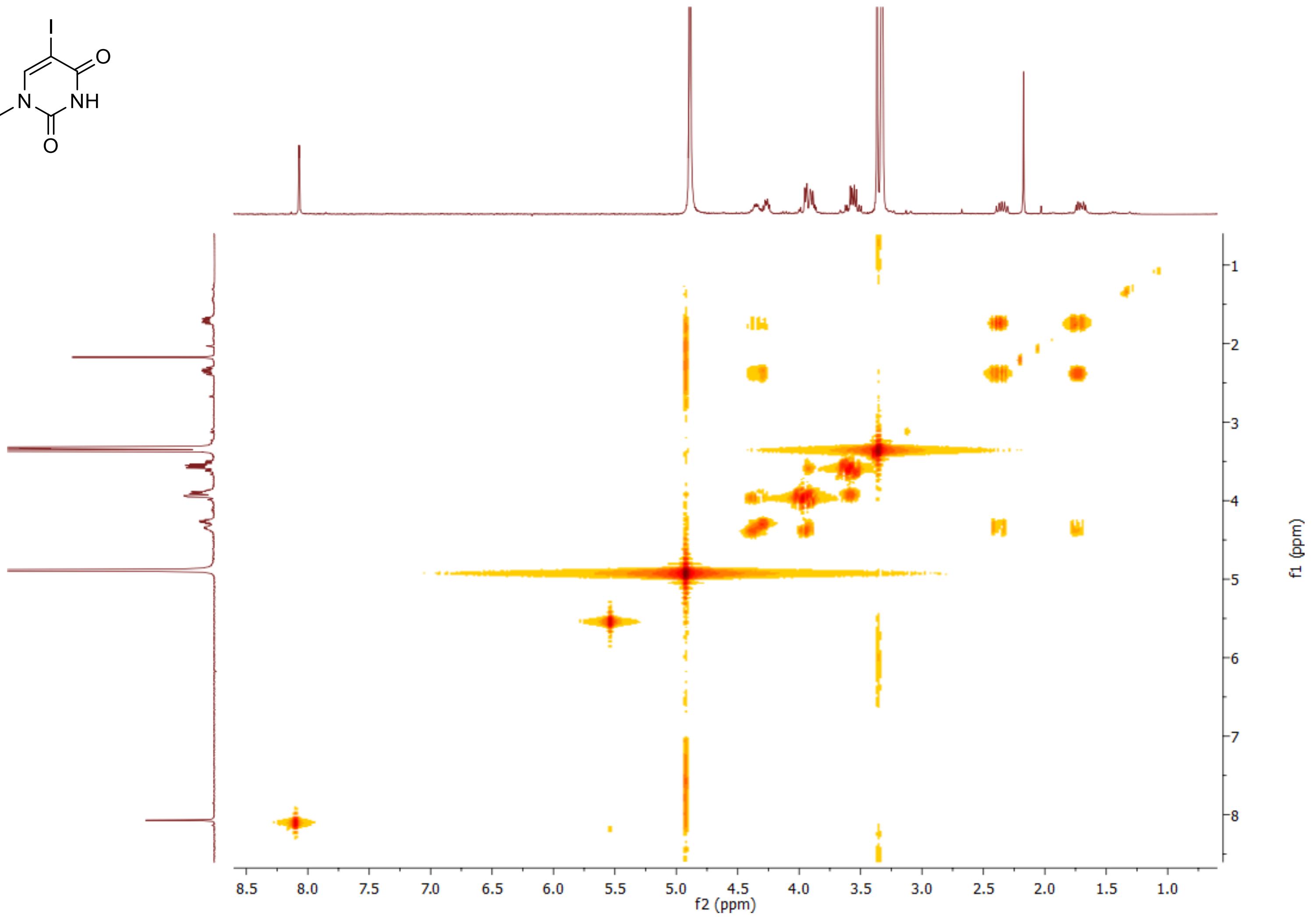

1,2-Dideoxy-1α-(methoxycarbonyl)-D-ribofuranose (13)

^{1}H NMR (300.13 MHz, MeOH-d_4)

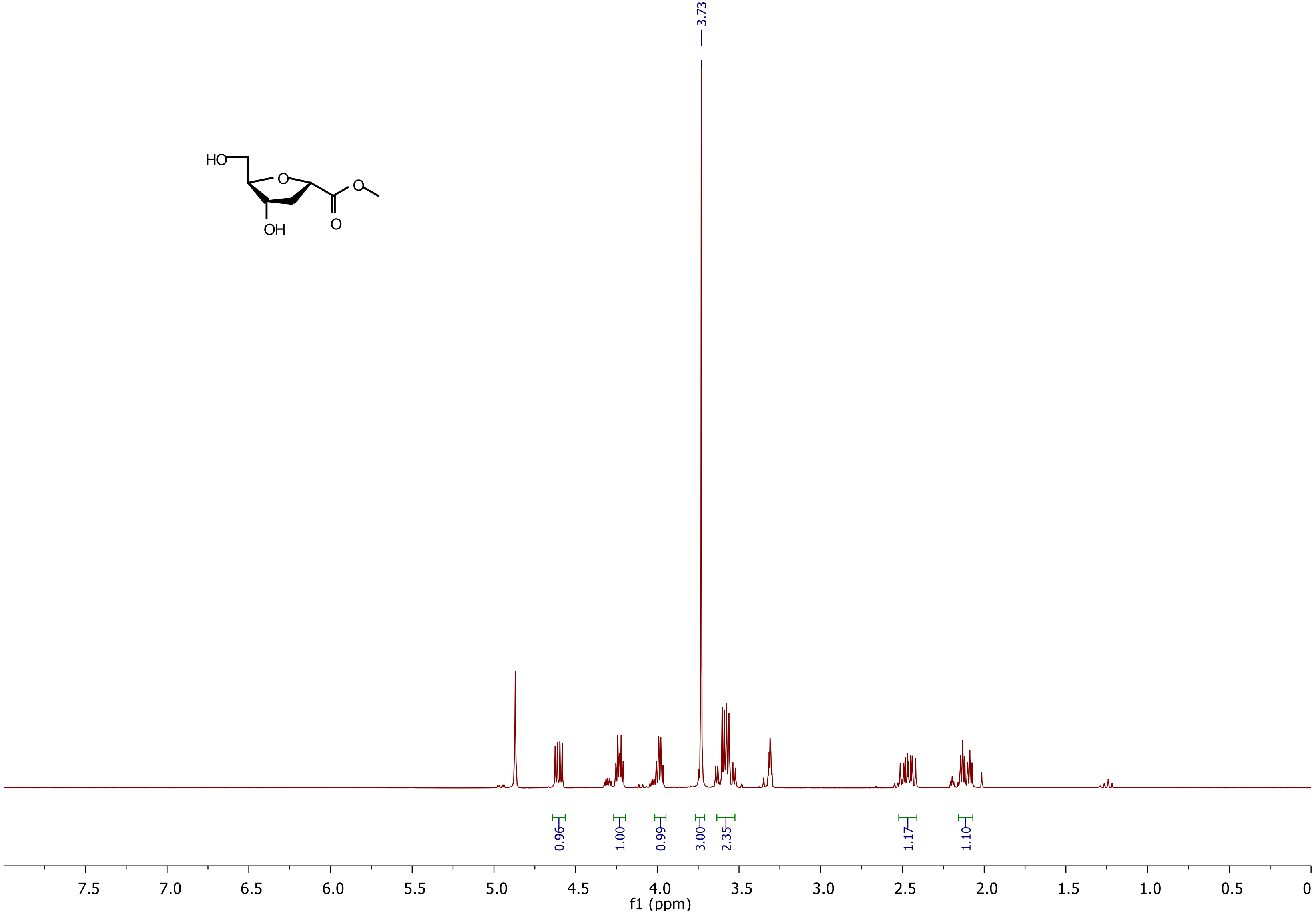

1,2-Dideoxy-1α-(methoxycarbonyl)-ᴅ-ribofuranose (13)

^{13}C NMR (75.5 MHz, MeOH-d_4)

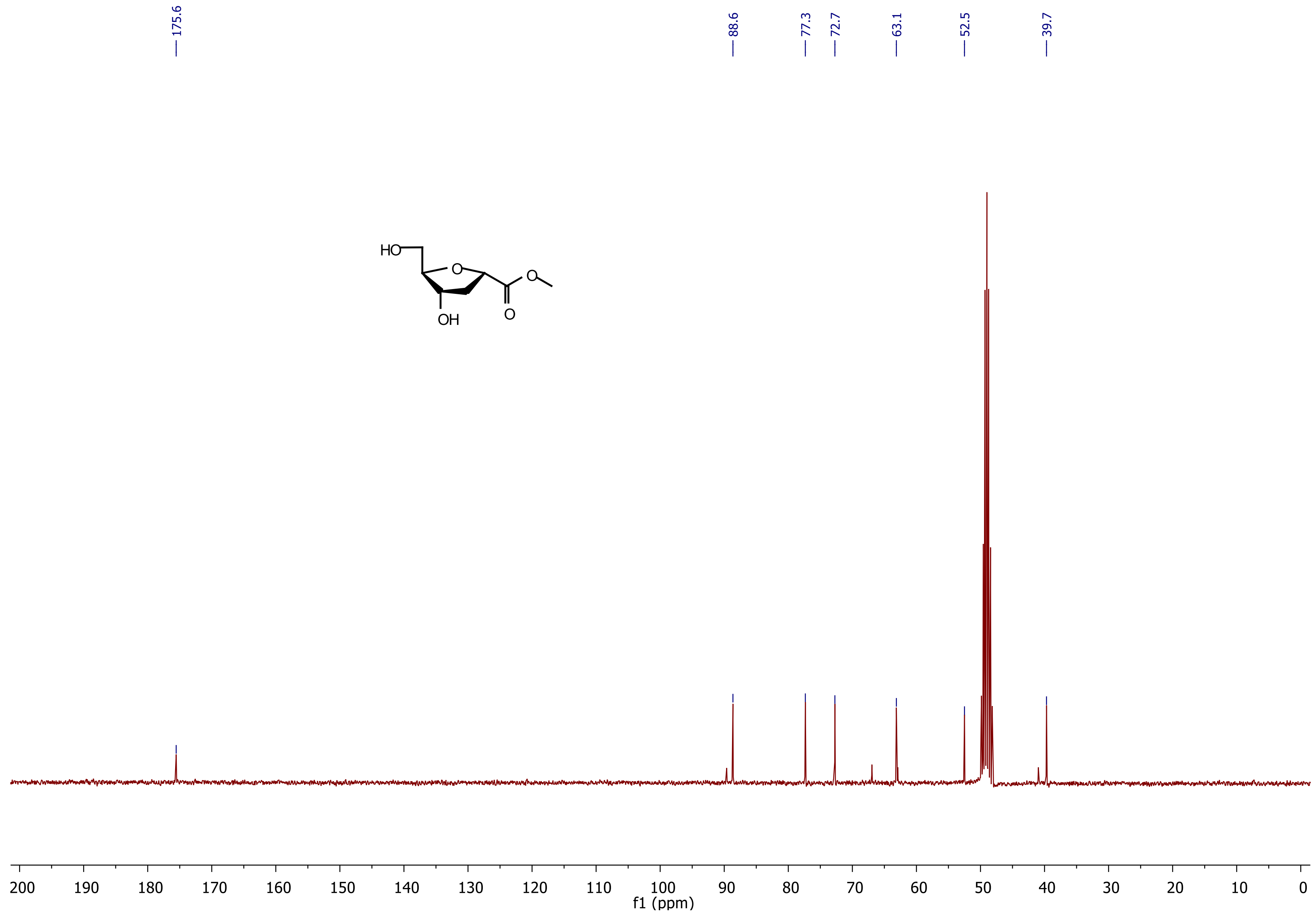

1,2-Dideoxy-1α-(methoxycarbonyl)-D-ribofuranose (13)

DEPT NMR (75.5 MHz, MeOH-d_4)

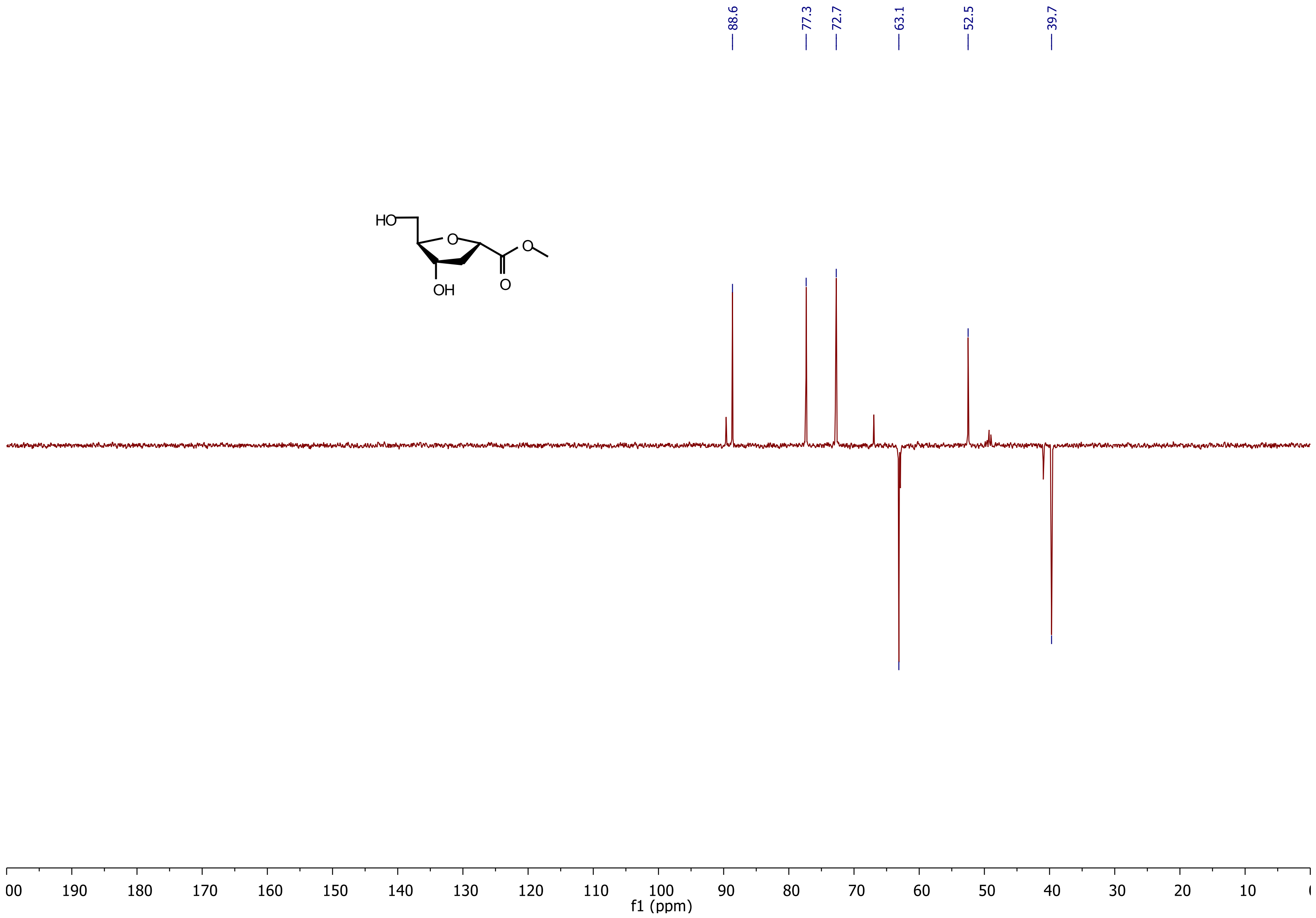

1,2-Dideoxy-1α-(methoxycarbonyl)-D-ribofuranose (13)

COSY NMR (MeOH-d_4)

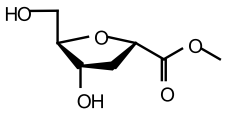

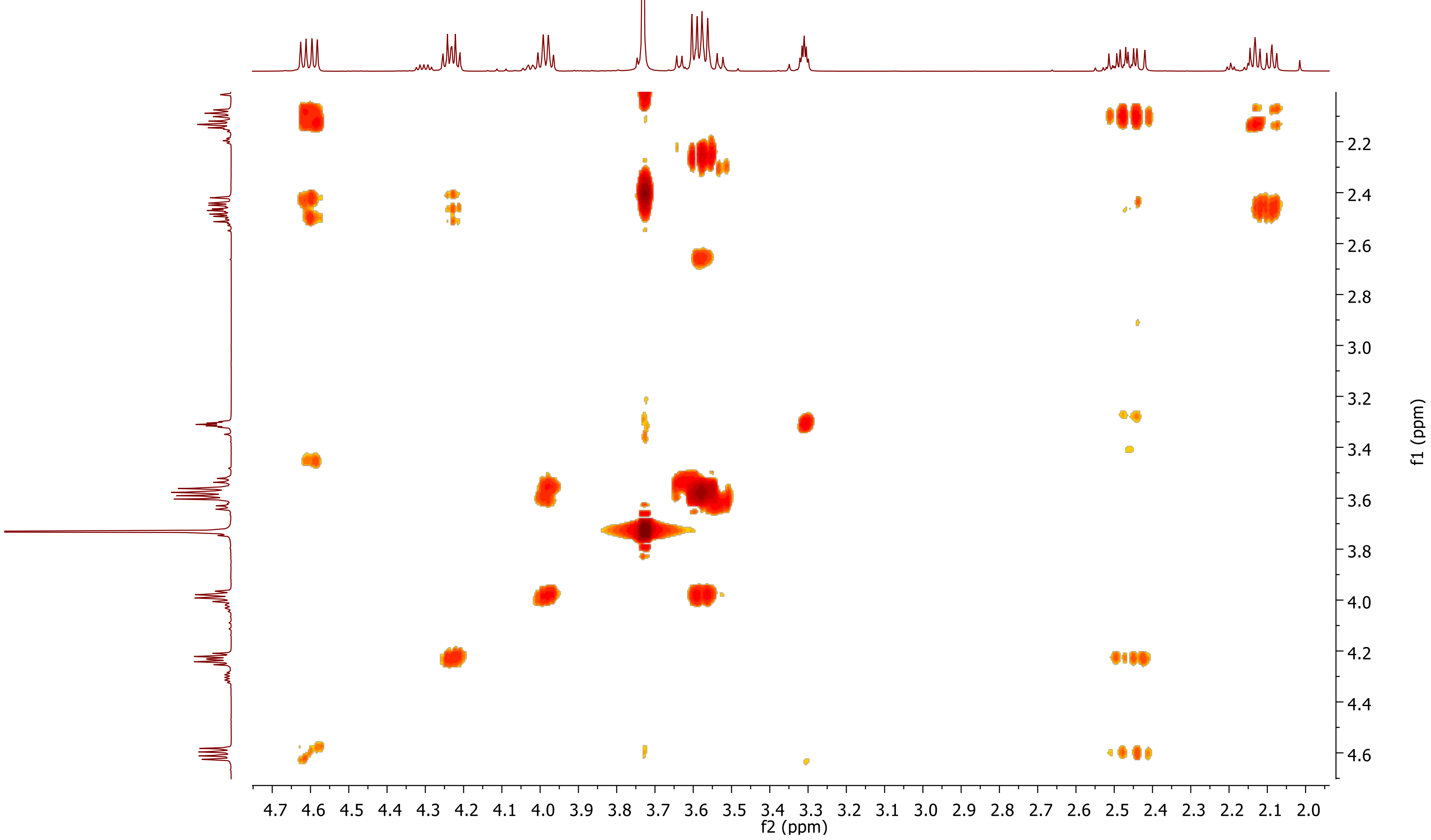

1,2-Dideoxy-1α-(methoxycarbonyl)-D-ribofuranose (7)

HSQC NMR (MeOH-d_4)

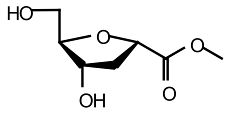

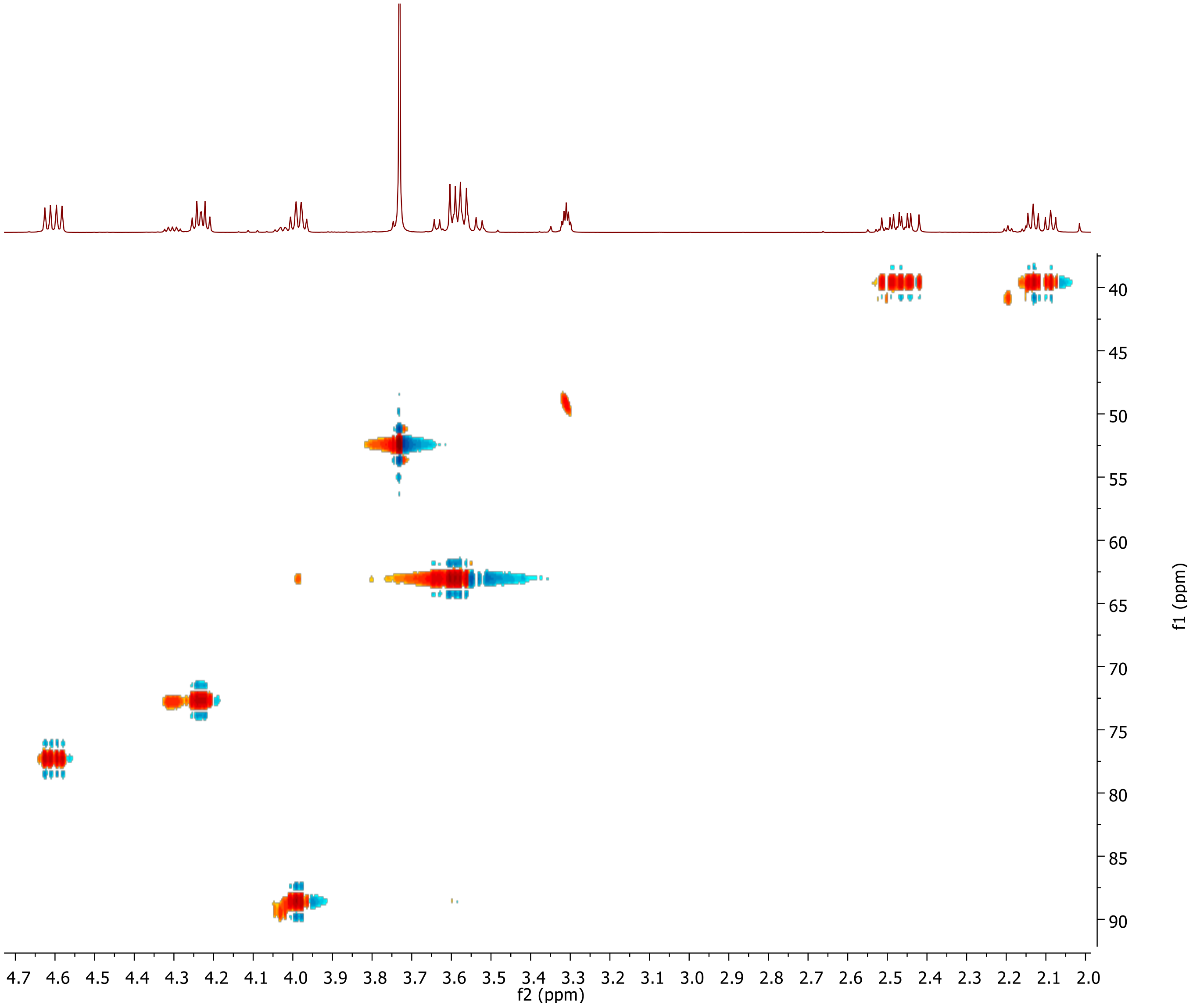

1,2-Dideoxy-1α-(methoxycarbonyl)-D-ribofuranose (13)

HMBC NMR (MeOH-d_4)

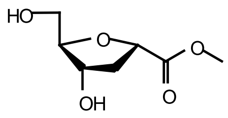

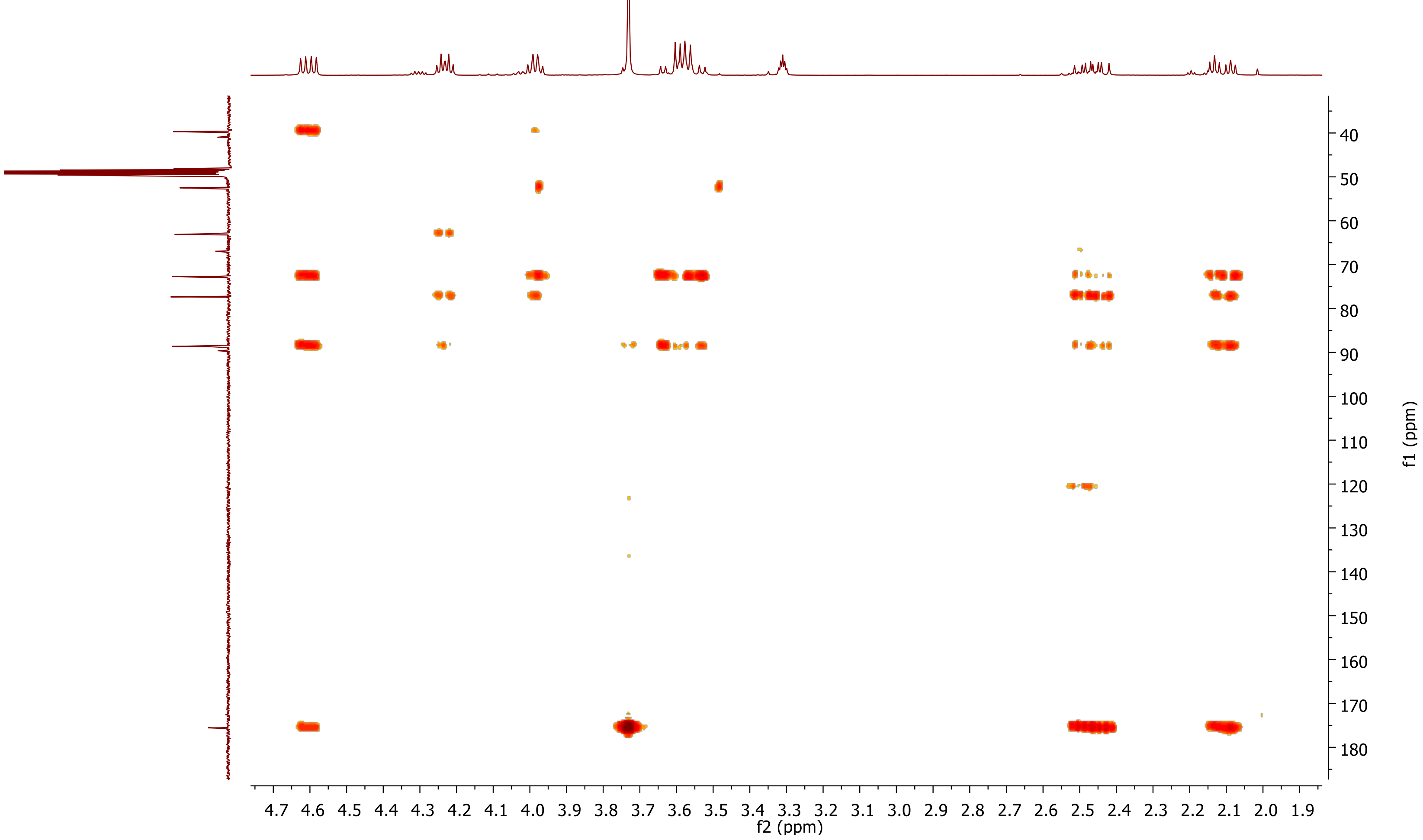

3,5-bis-*O*-(*tert*-Butyldimethylsilyl)-1,2-dideoxy-1α-(methoxycarbonyl)-D-ribofuranose (20)

^{1}H NMR (300.13 MHz, MeOH-d_4)

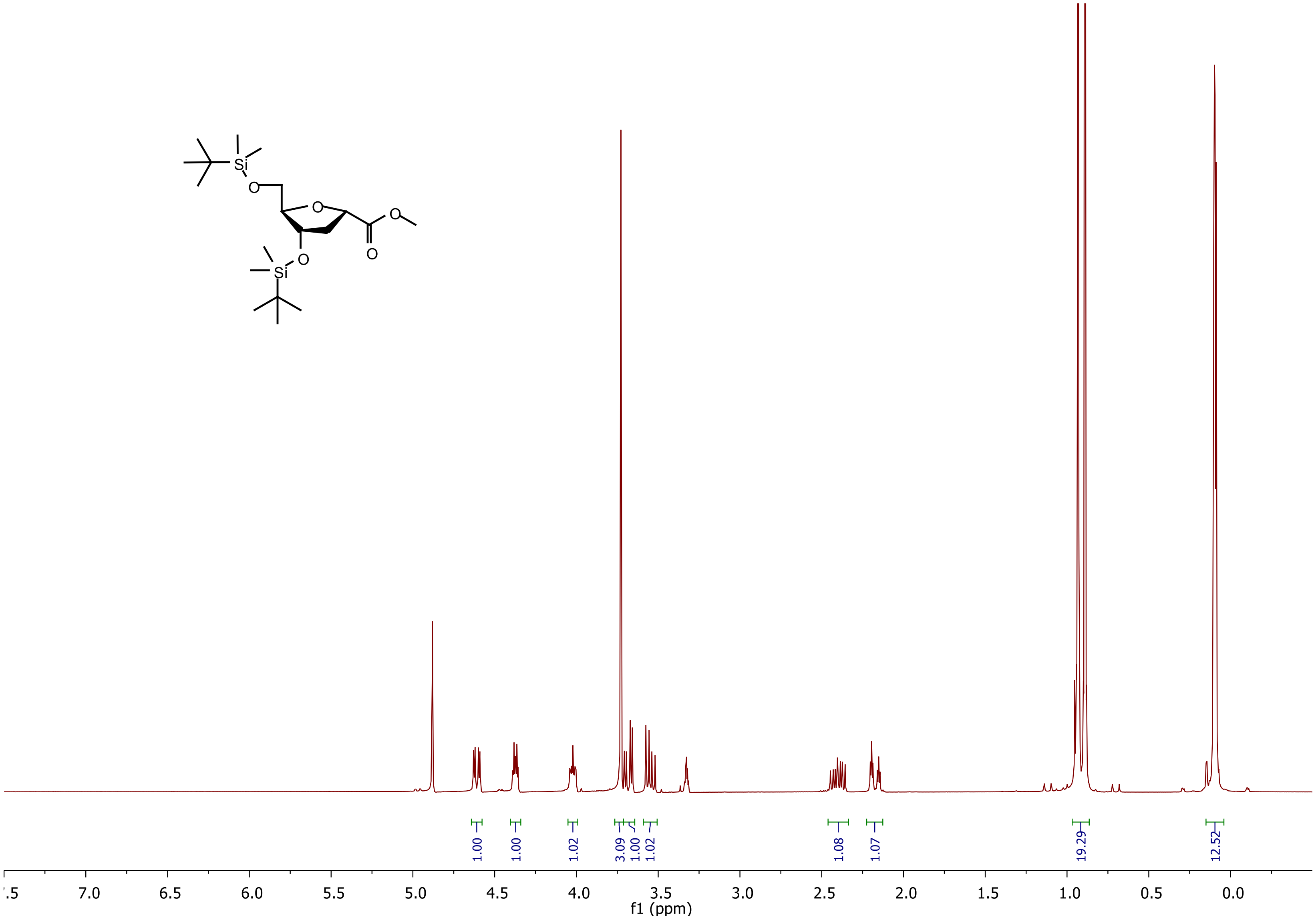

3,5-bis-*O*-(*tert*-Butyldimethylsilyl)-1,2-dideoxy-1α-(methoxycarbonyl)-ᴅ-ribofuranose (20)

^{13}C NMR (75.5 MHz, MeOH-d_4)

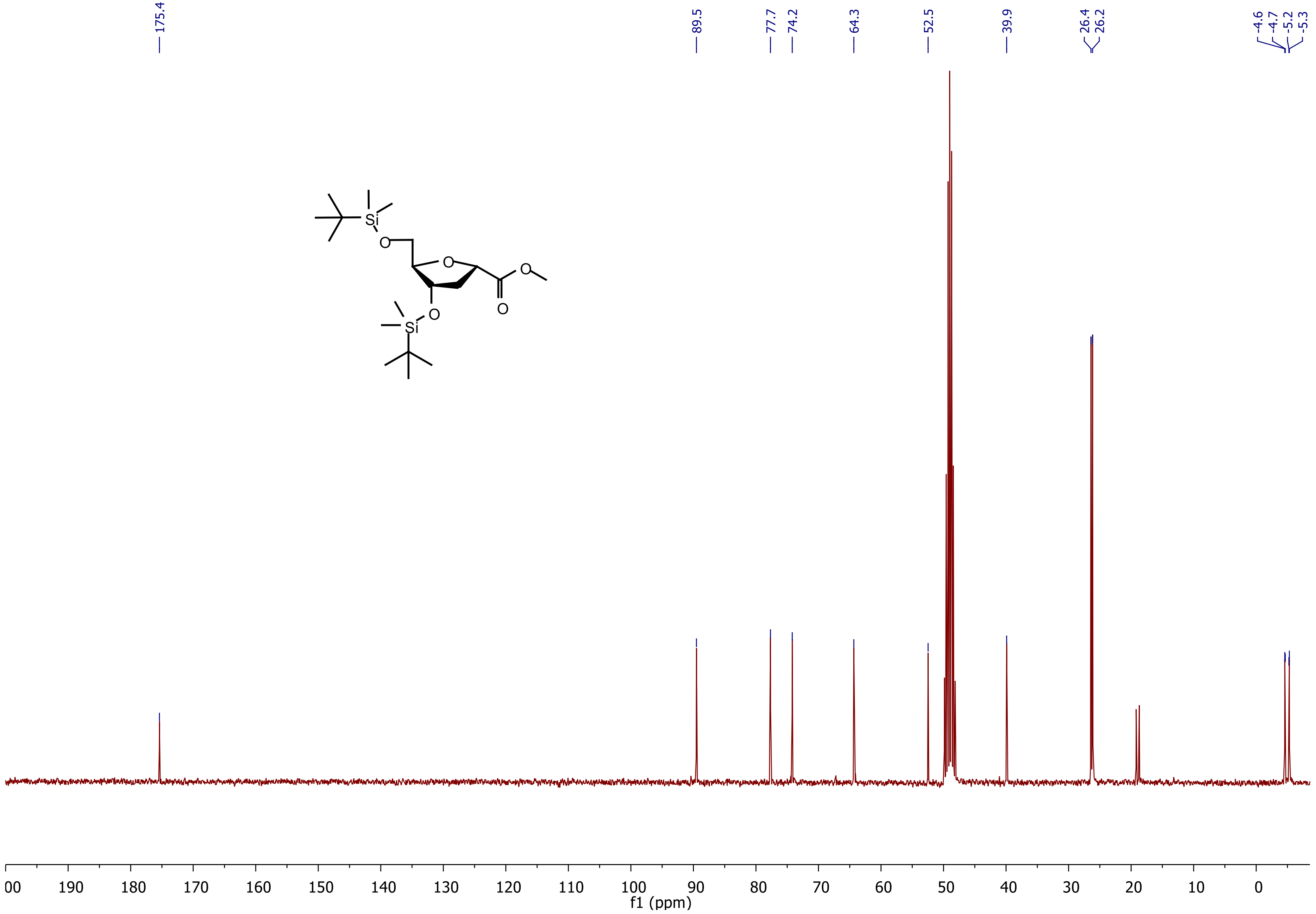

3,5-bis-*O*-(*tert*-Butyldimethylsilyl)-1,2-dideoxy-1α-(methoxycarbonyl)-D-ribofuranose (20)

DEPT NMR (75.5 MHz, MeOH-d_4)

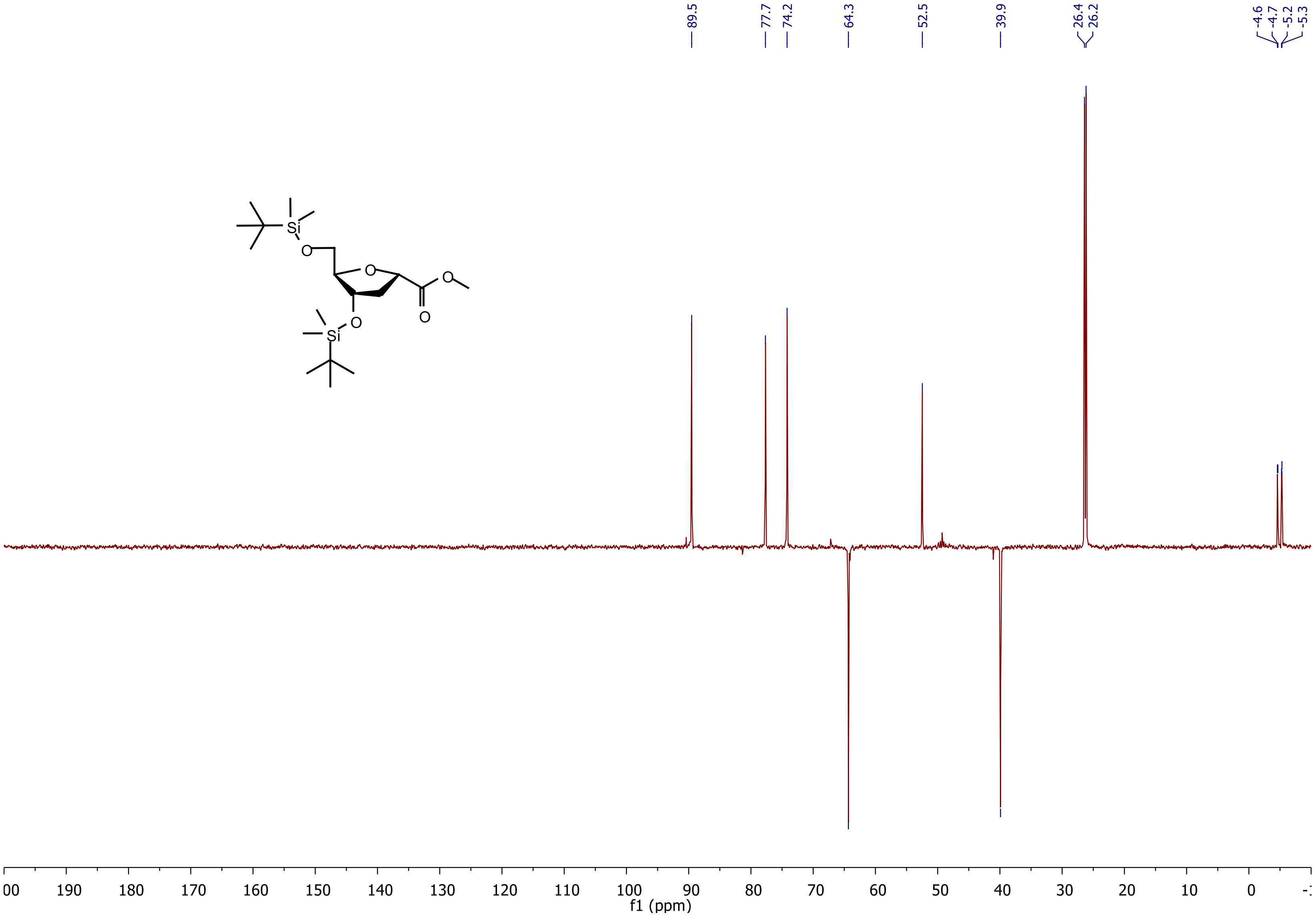

3,5-bis-*O*-(*tert*-Butyldimethylsilyl)-1,2-dideoxy-1α-(methoxycarbonyl)-D-ribofuranose (20)

COSY NMR (MeOH-d_4)

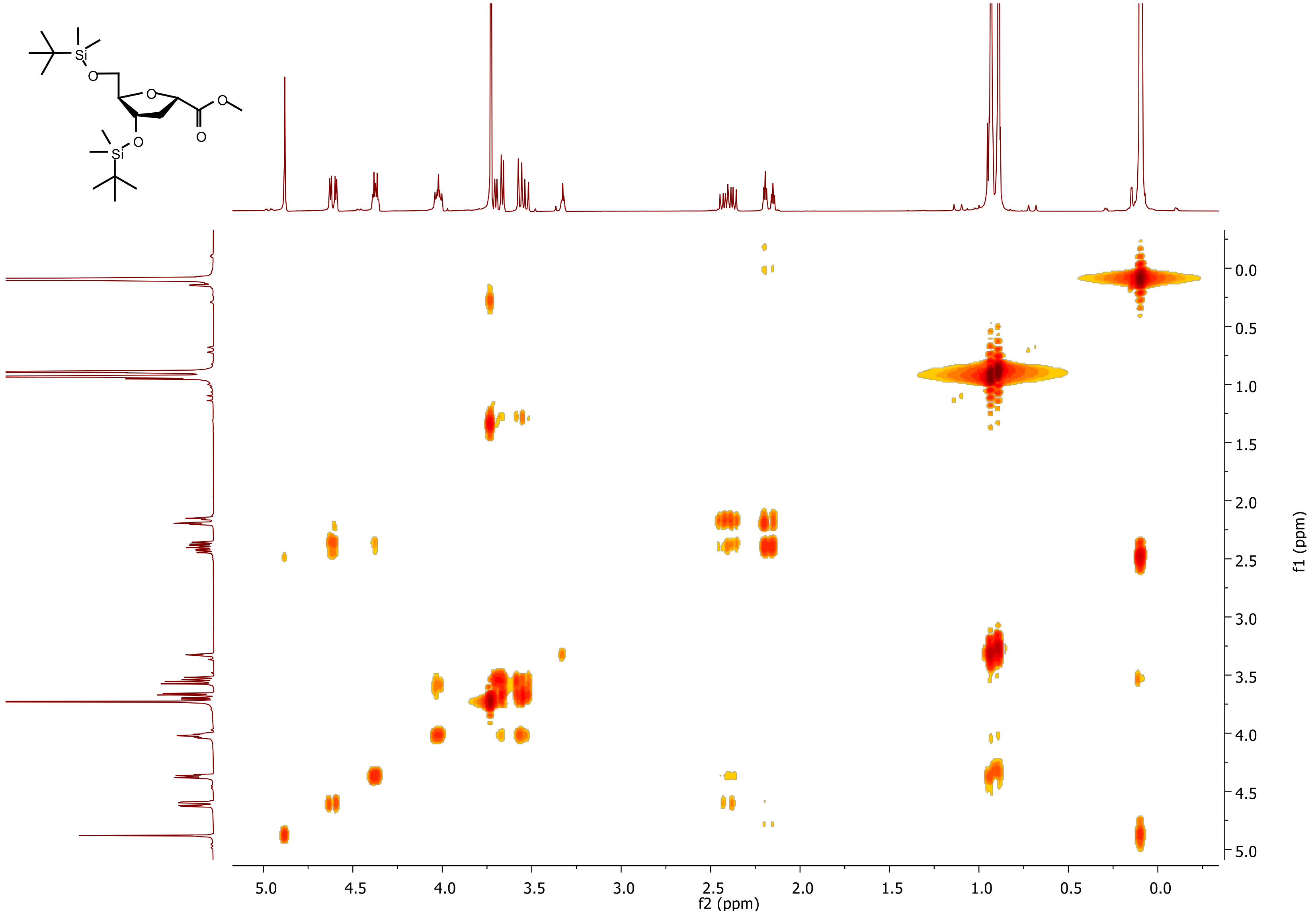

3,5-bis-*O*-(*tert*-Butyldimethylsilyl)-1,2-dideoxy-1α-(methoxycarbonyl)-D-ribofuranose (20)

HSQC NMR (MeOH-d_4)

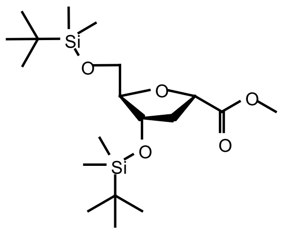

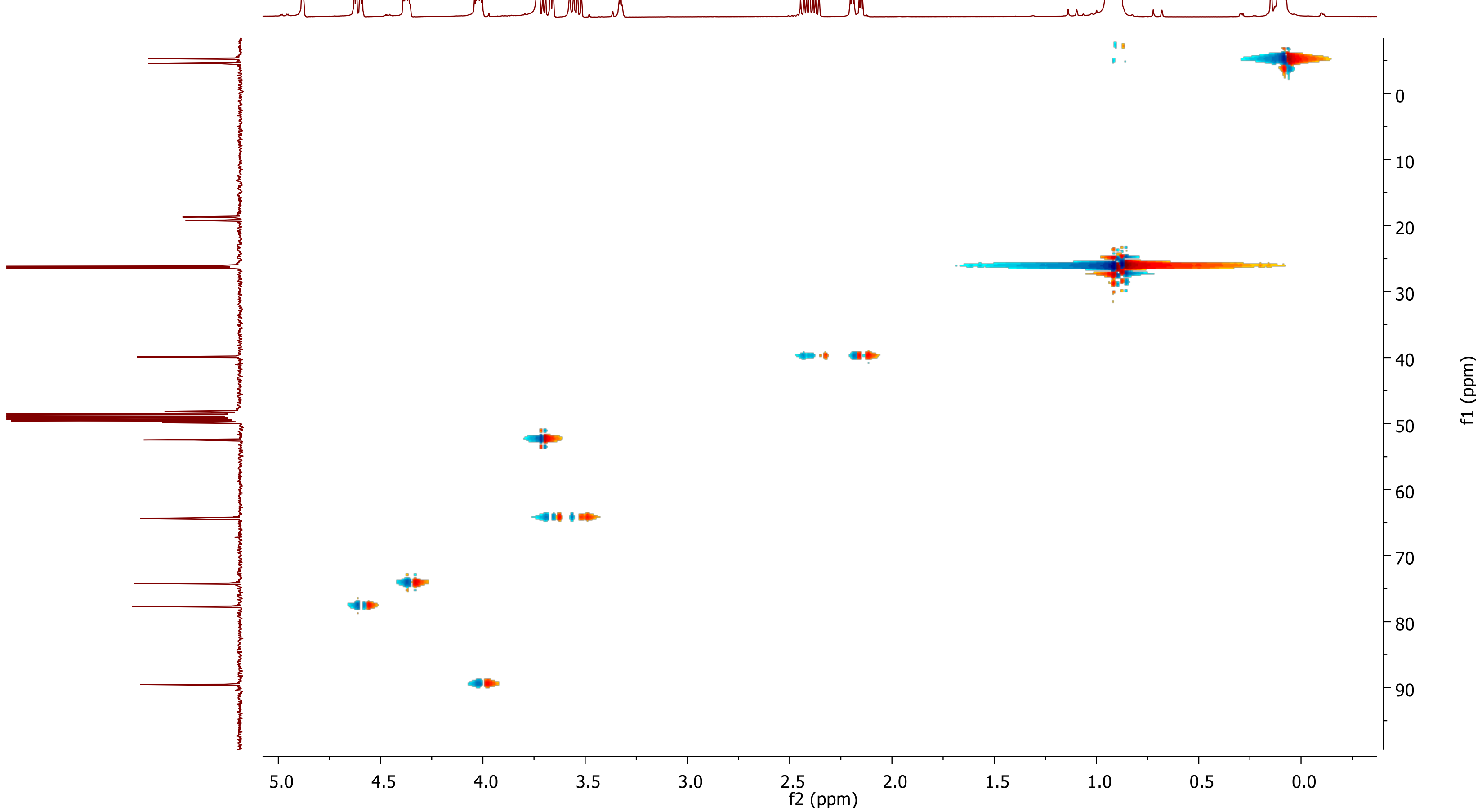

3,5-bis-*O*-(*tert*-Butyldimethylsilyl)-1,2-dideoxy-1α-(methoxycarbonyl)-ᴅ-ribofuranose (20)

HMBC NMR (MeOH-d_4)

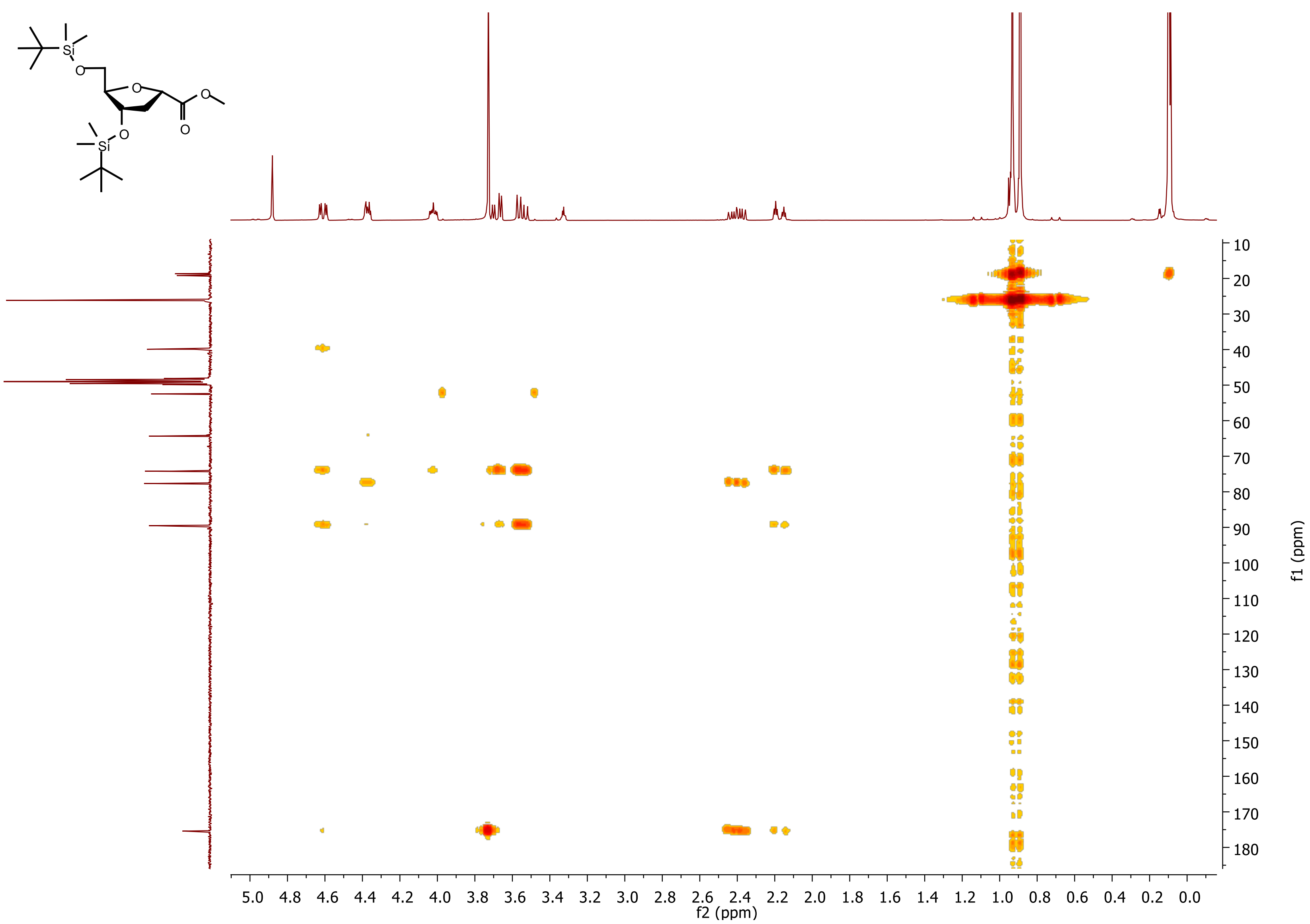

3,5-bis-*O*-(*tert*-Butyldimethylsilyl)-1,2-dideoxy-1α-(hydroxymethyl)-D-ribofuranose (15)

^{1}H NMR (300.13 MHz, MeOH-d_4)

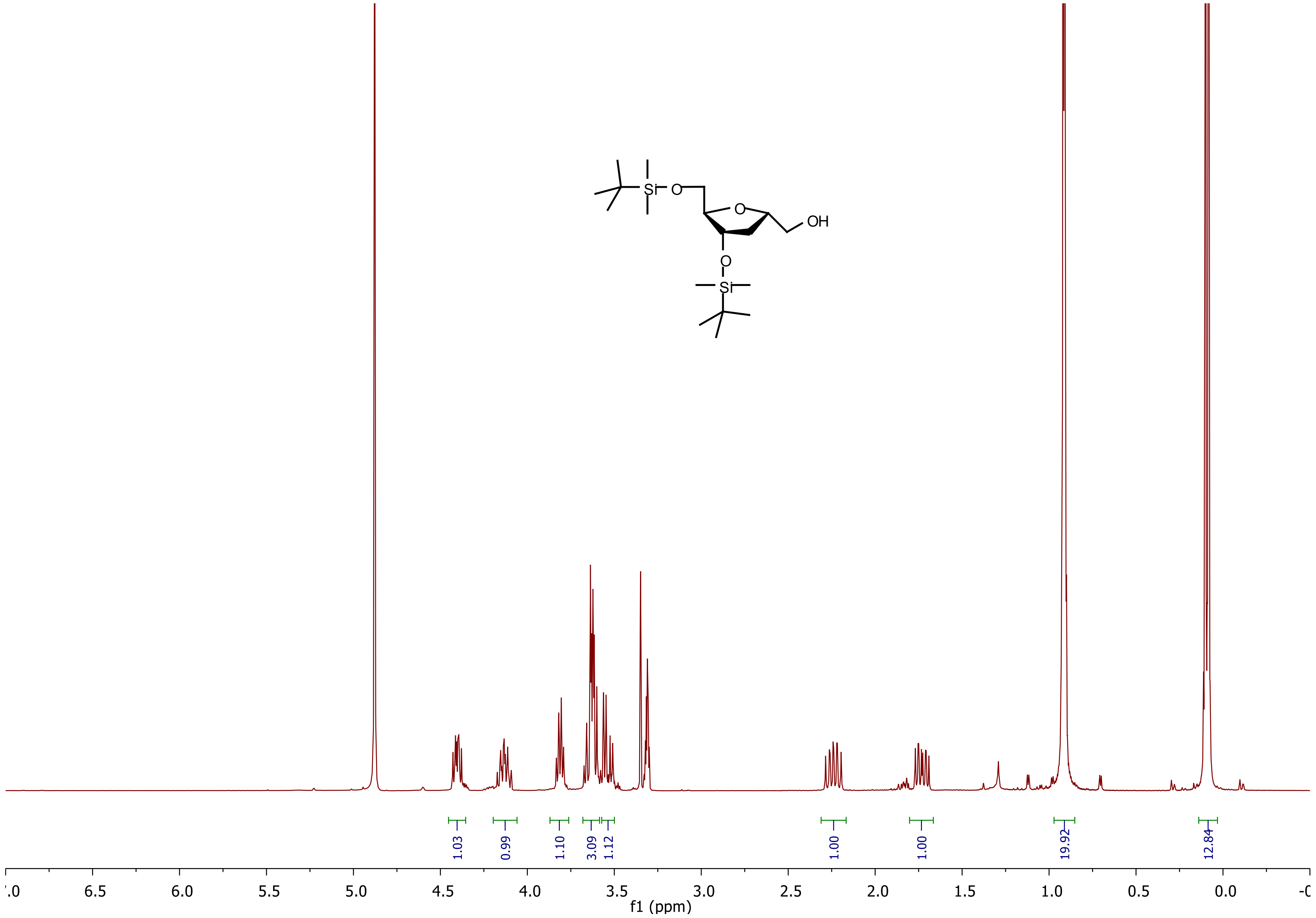

3,5-bis-*O*-(*tert*-Butyldimethylsilyl)-1,2-dideoxy-1α-(hydroxymethyl)-D-ribofuranose (15)

^{13}C NMR (75.5 MHz, MeOH-d_4)

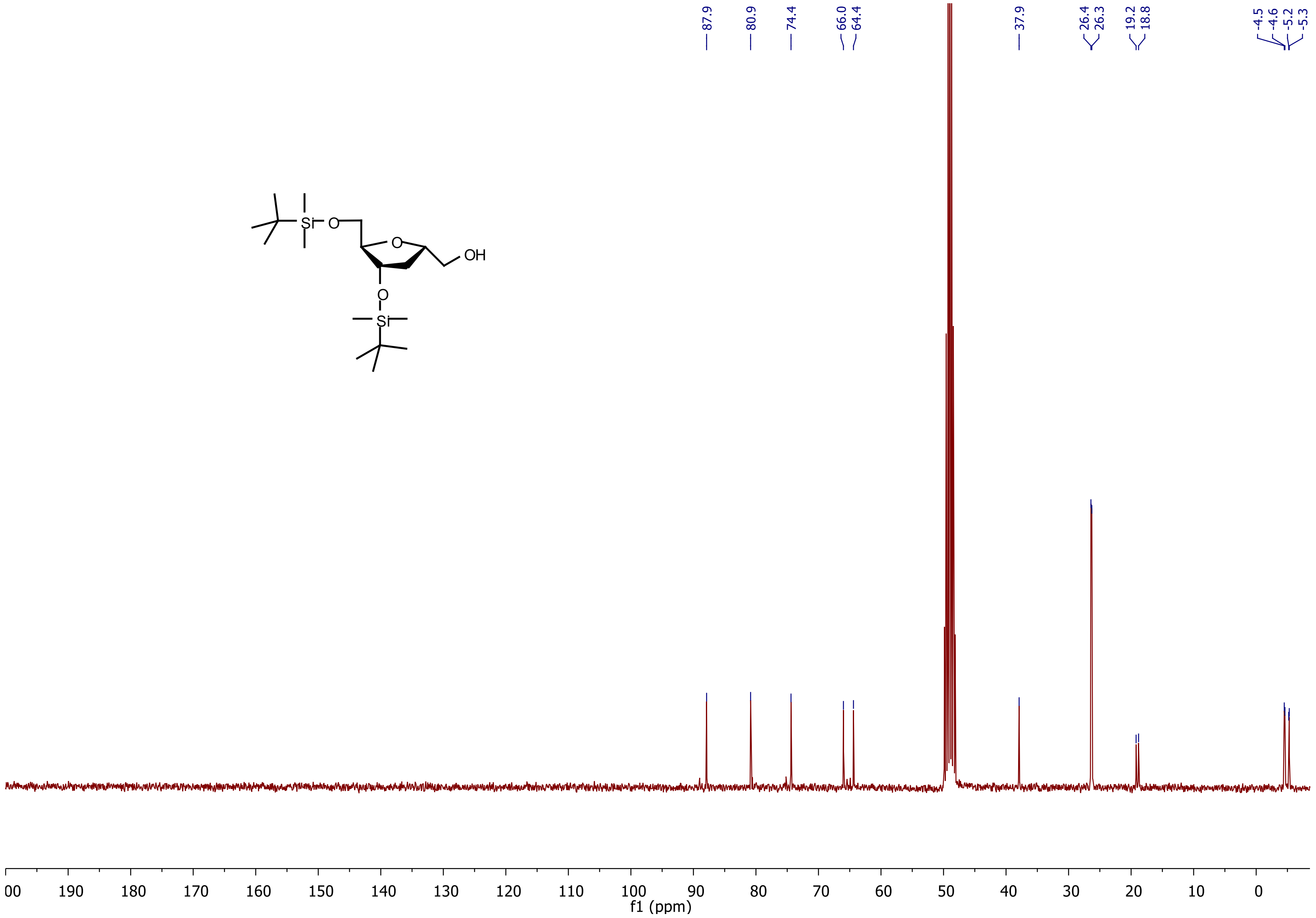

3,5-bis-*O*-(*tert*-Butyldimethylsilyl)-1,2-dideoxy-1α-(hydroxymethyl)-D-ribofuranose (15)

DEPT NMR (75.5 MHz, MeOH-d_4)

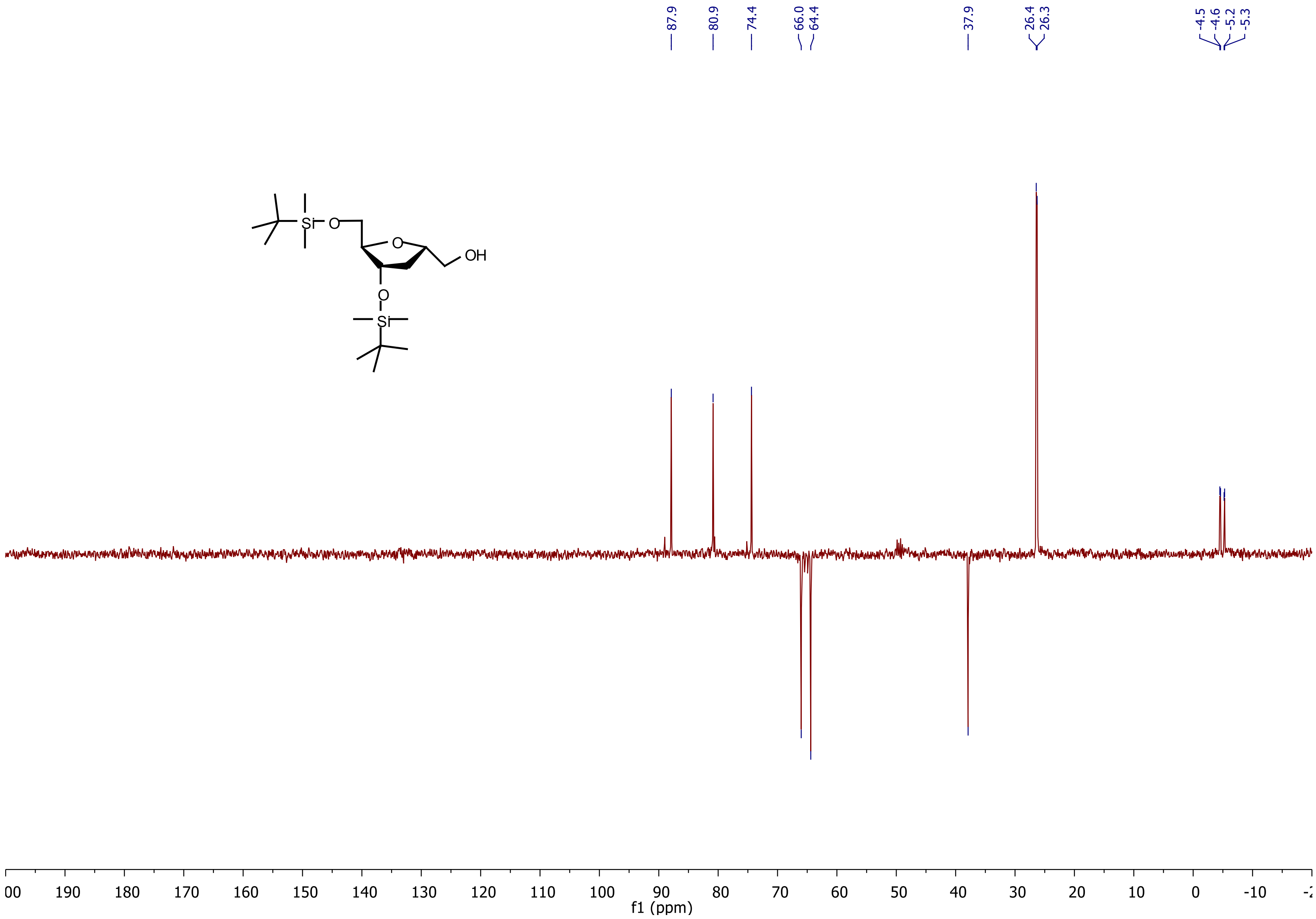

3,5-bis-*O*-(*tert*-Butyldimethylsilyl)-1,2-dideoxy-1α-(hydroxymethyl)-D-ribofuranose (15)

COSY NMR (MeOH-d_4)

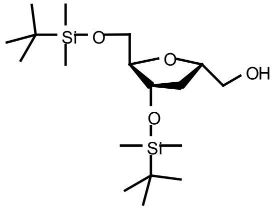

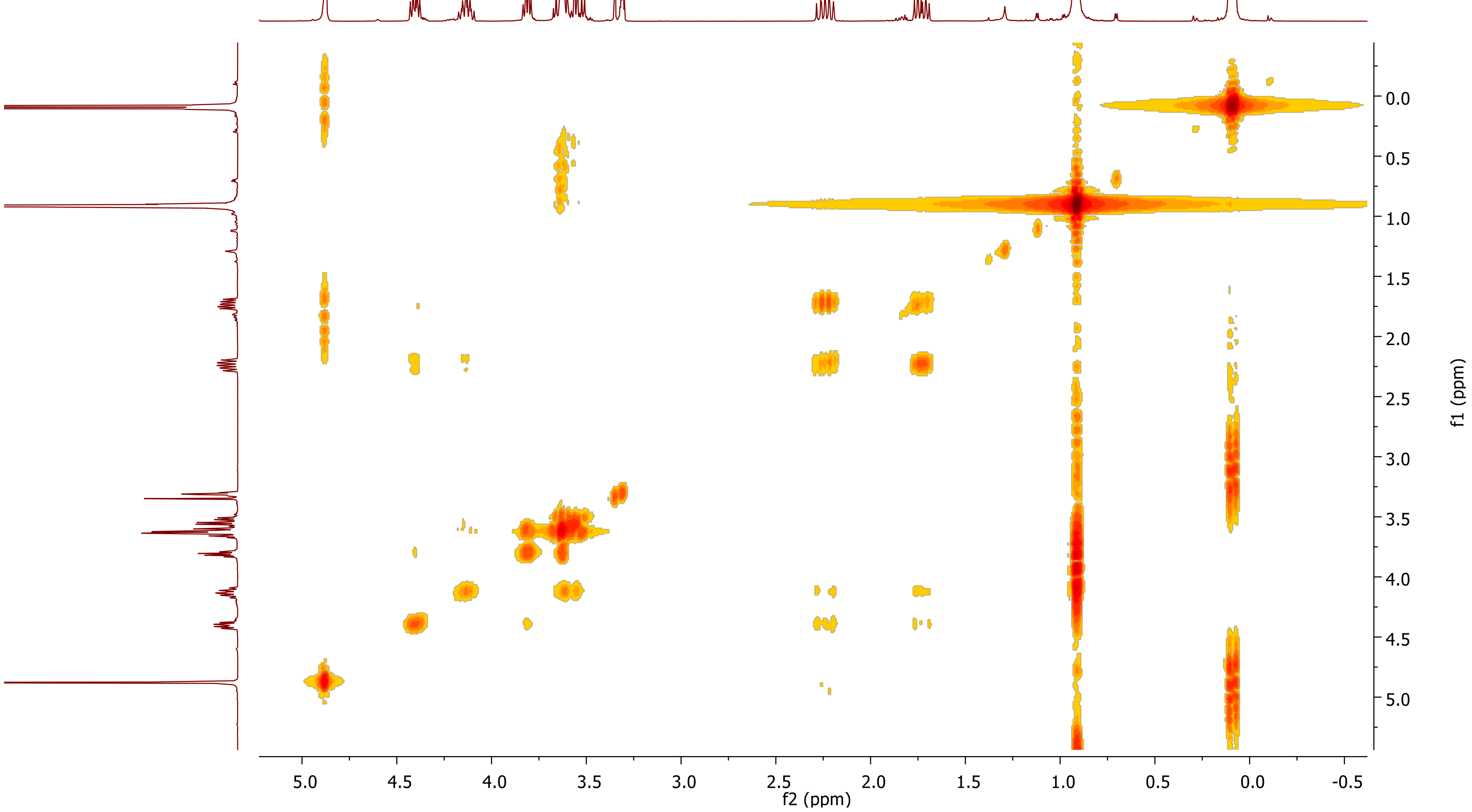

3,5-bis-*O*-(*tert*-Butyldimethylsilyl)-1,2-dideoxy-1α-(hydroxymethyl)-D-ribofuranose (15)

HSQC NMR (MeOH-d_4)

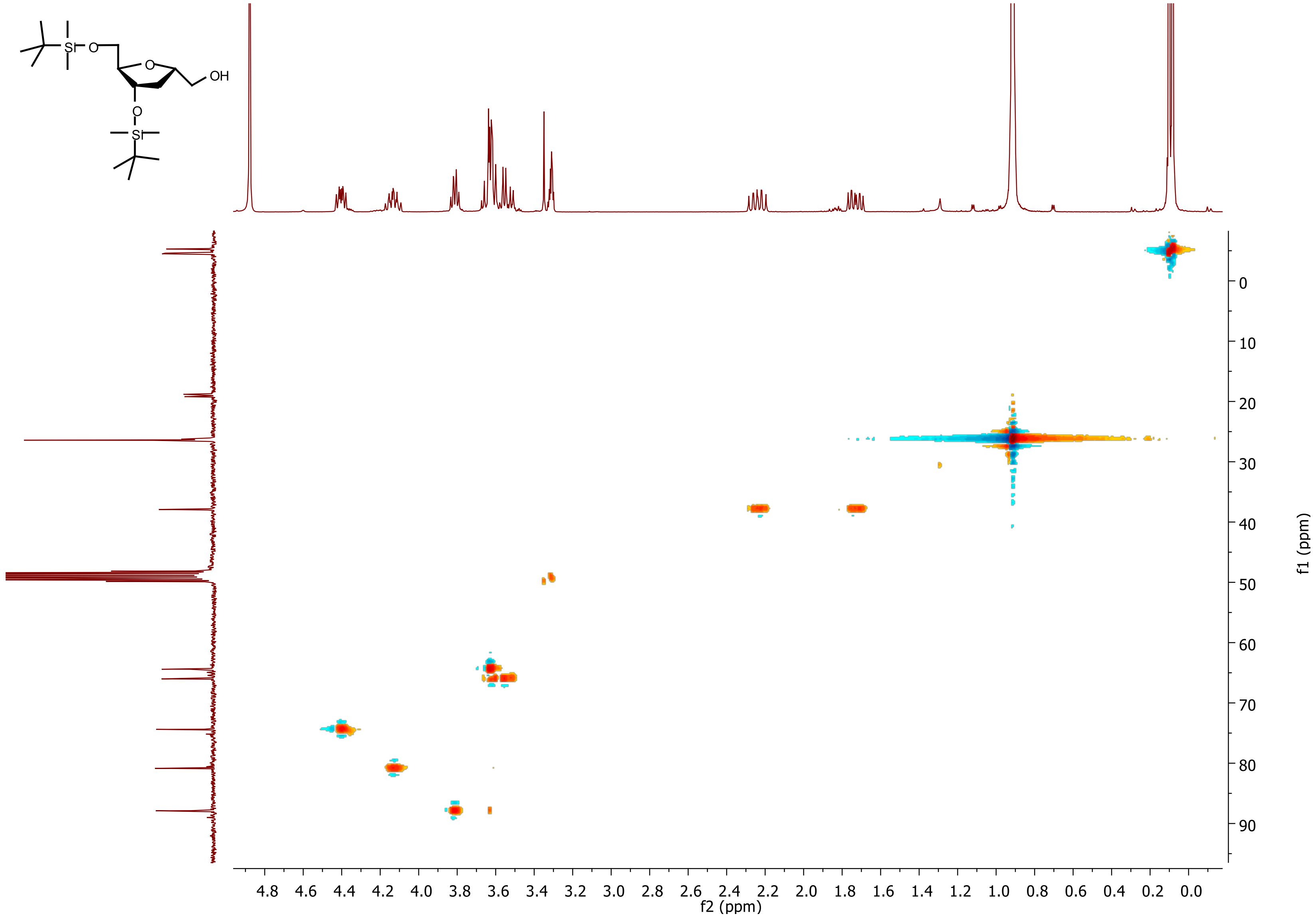

3,5-bis-*O*-(*tert*-Butyldimethylsilyl)-1,2-dideoxy-1α-(hydroxymethyl)-D-ribofuranose (15)

HMBC NMR (MeOH-d_4)

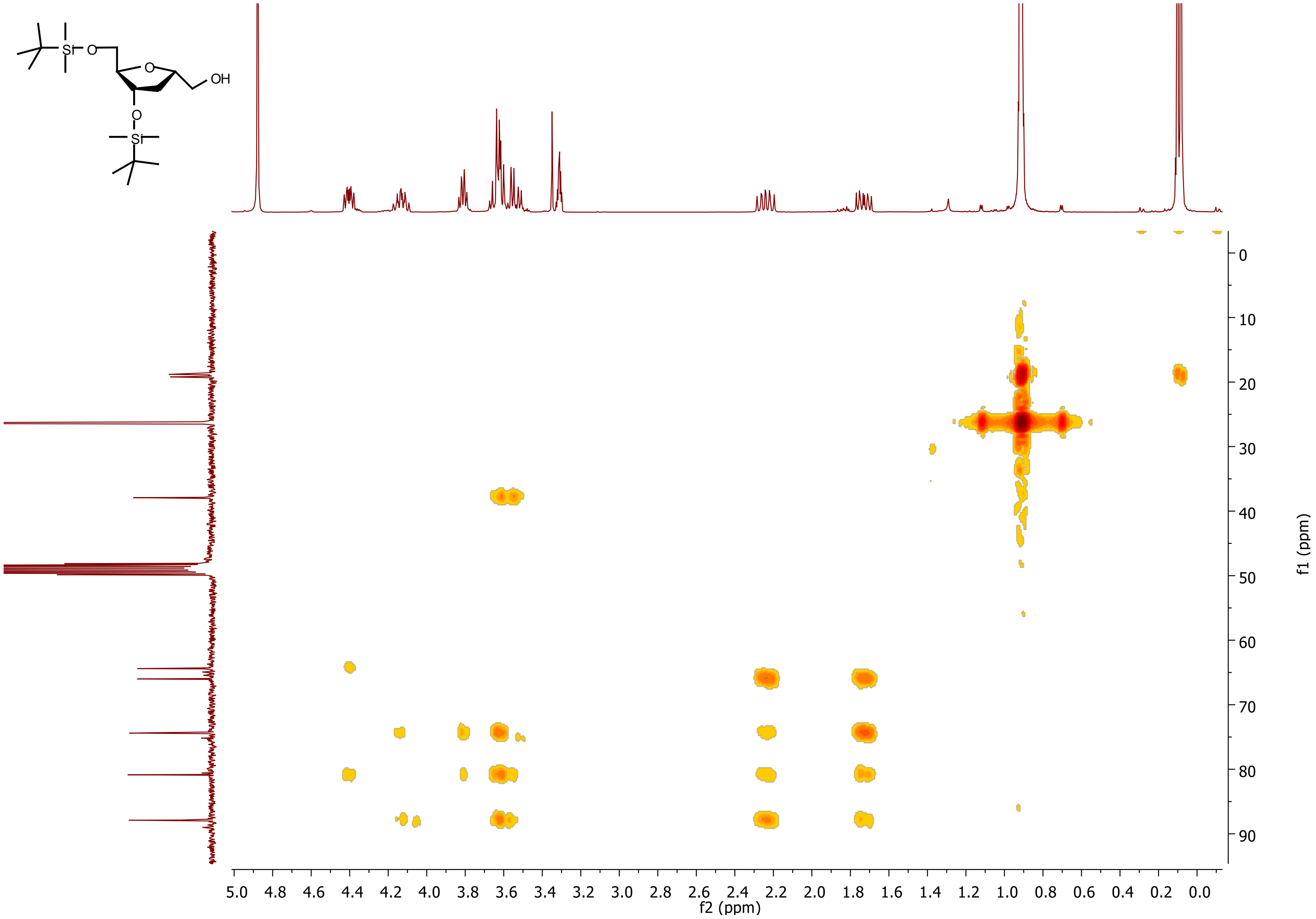

3,5-bis-*O*-(*tert*-Butyldimethylsilyl)-1,2-dideoxy-1α-(((methylsulfonyl)oxy)methyl)-D-ribofuranose (16)

^{1}H NMR (300.13 MHz, MeOH-d_4)

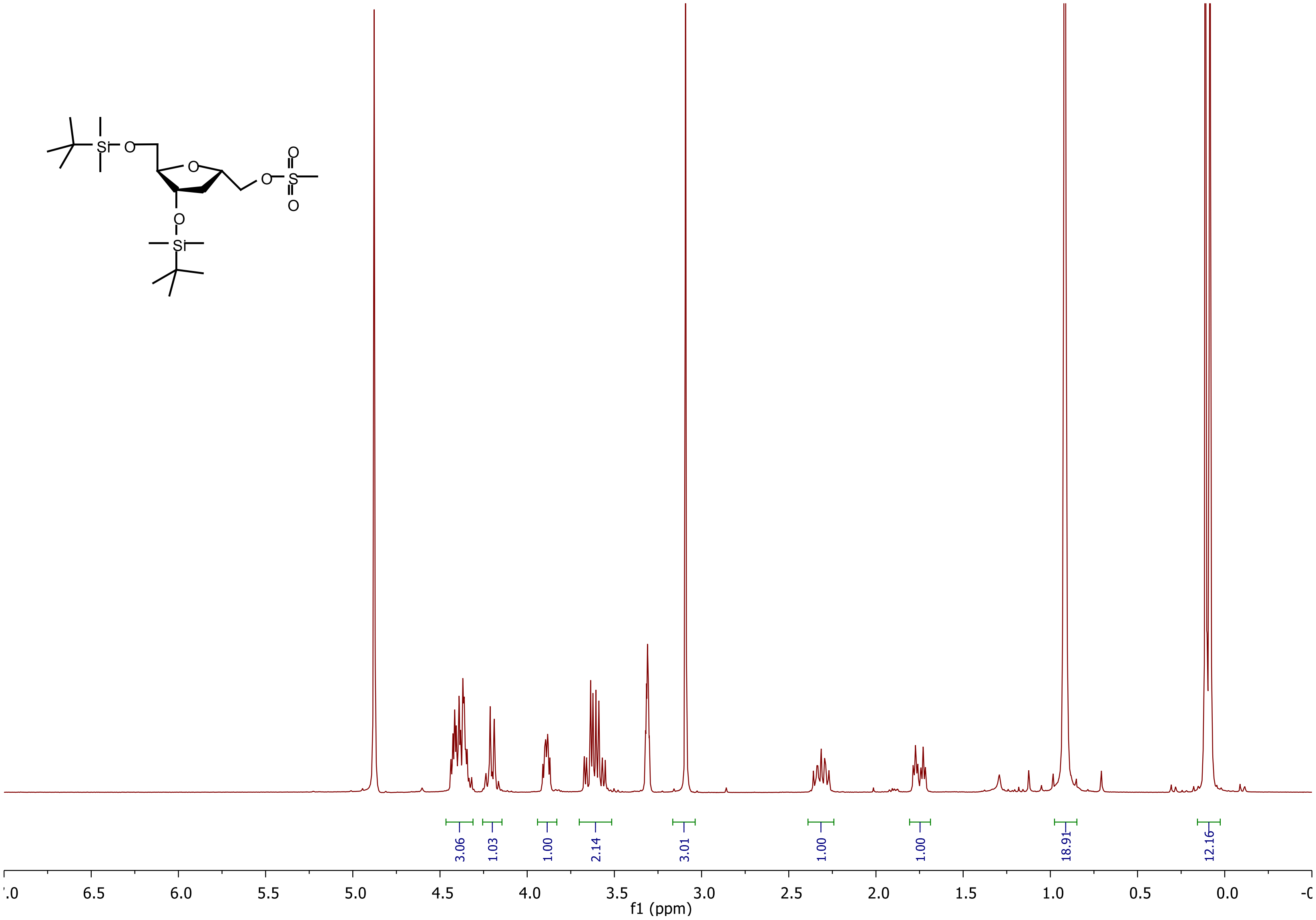

3,5-bis-*O*-(*tert*-Butyldimethylsilyl)-1,2-dideoxy-1α-(((methylsulfonyl)oxy)methyl)-D-ribofuranose (16)

^{13}C NMR (75.5 MHz, MeOH-d_4)

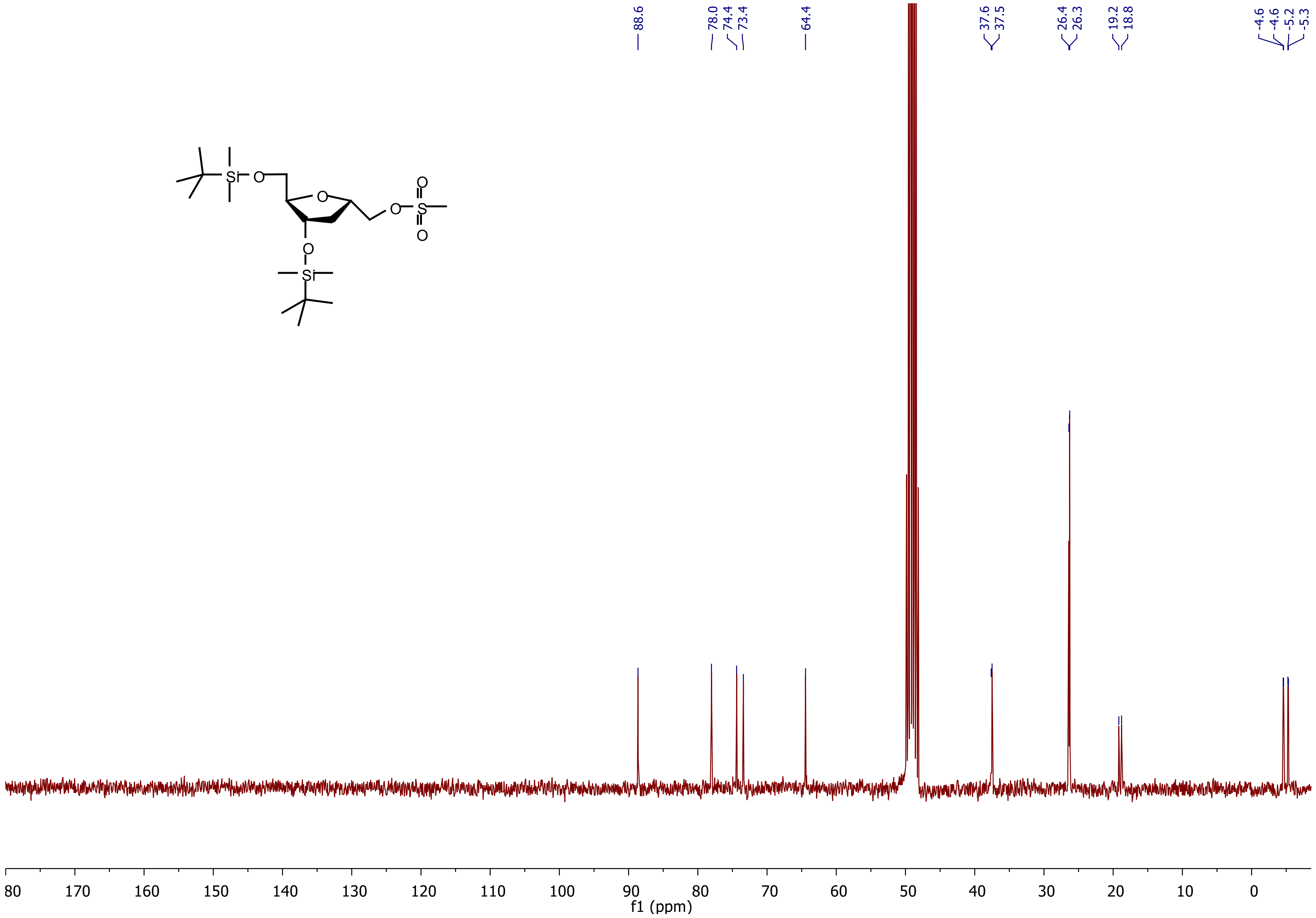

3,5-bis-*O*-(*tert*-Butyldimethylsilyl)-1,2-dideoxy-1α-(((methylsulfonyl)oxy)methyl)-D-ribofuranose (16)

DEPT NMR (75.5 MHz, MeOH-d_4)

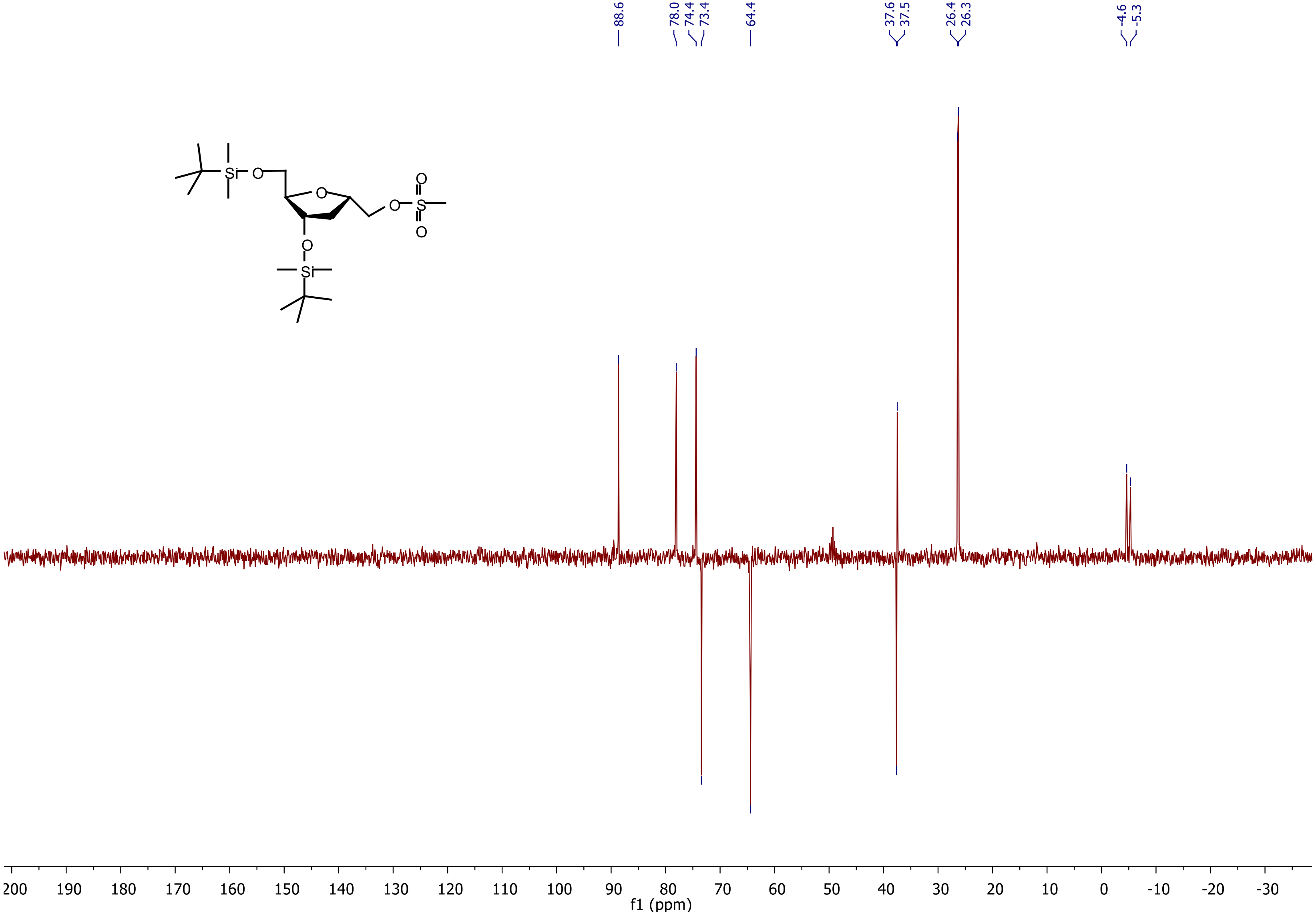

3,5-bis-*O*-(*tert*-Butyldimethylsilyl)-1,2-dideoxy-1α-(((methylsulfonyl)oxy)methyl)-D-ribofuranose (16)

COSY NMR (MeOH-d_4)

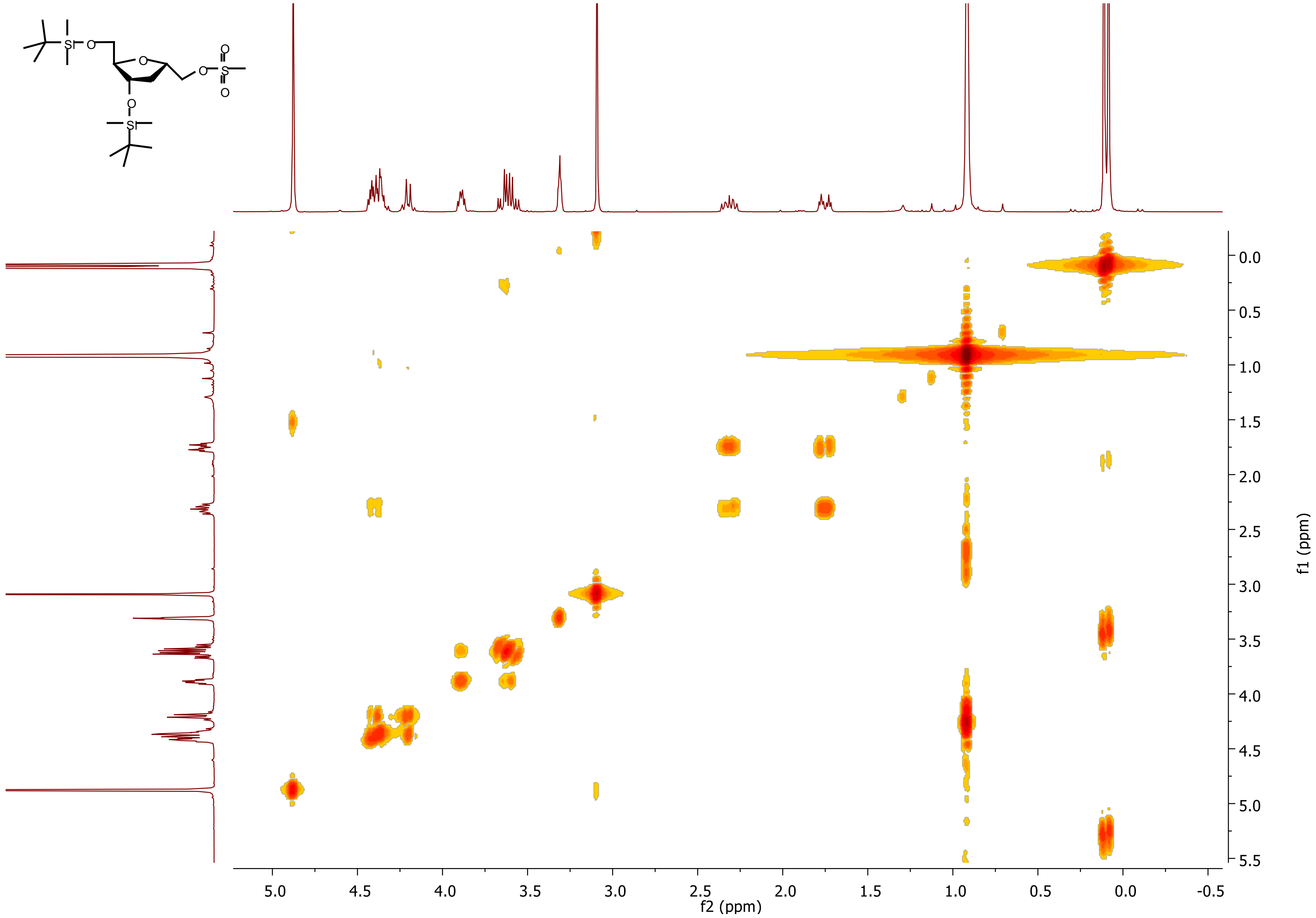

3,5-bis-*O*-(*tert*-Butyldimethylsilyl)-1,2-dideoxy-1α-(((methylsulfonyl)oxy)methyl)-ᴅ-ribofuranose (16)

HSQC NMR (MeOH-d_4)

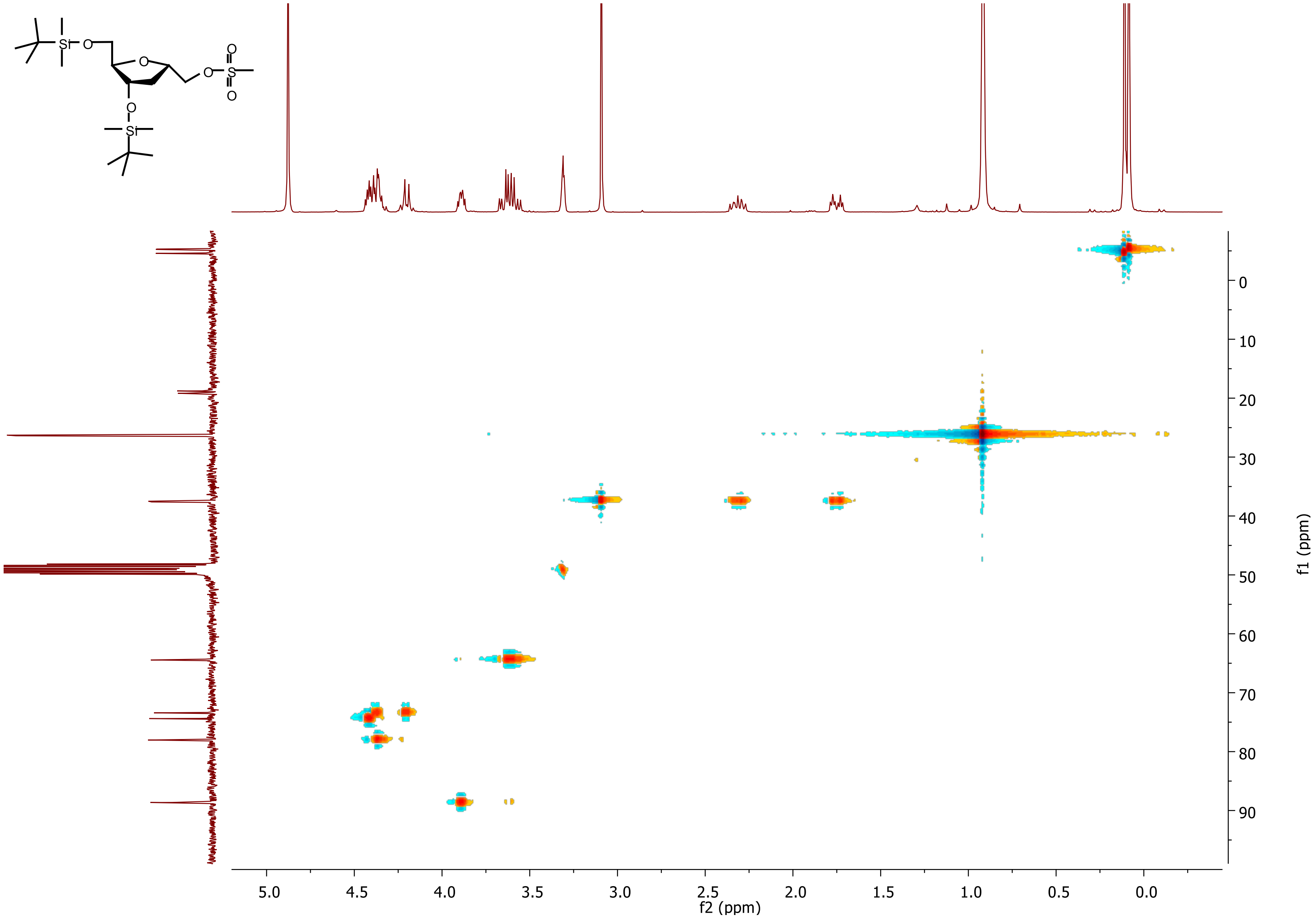

3,5-bis-*O*-(*tert*-Butyldimethylsilyl)-1,2-dideoxy-1α-(((methylsulfonyl)oxy)methyl)-D-ribofuranose (16)

HMBC NMR (MeOH-d_4)

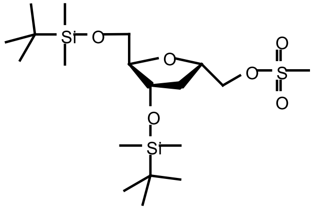

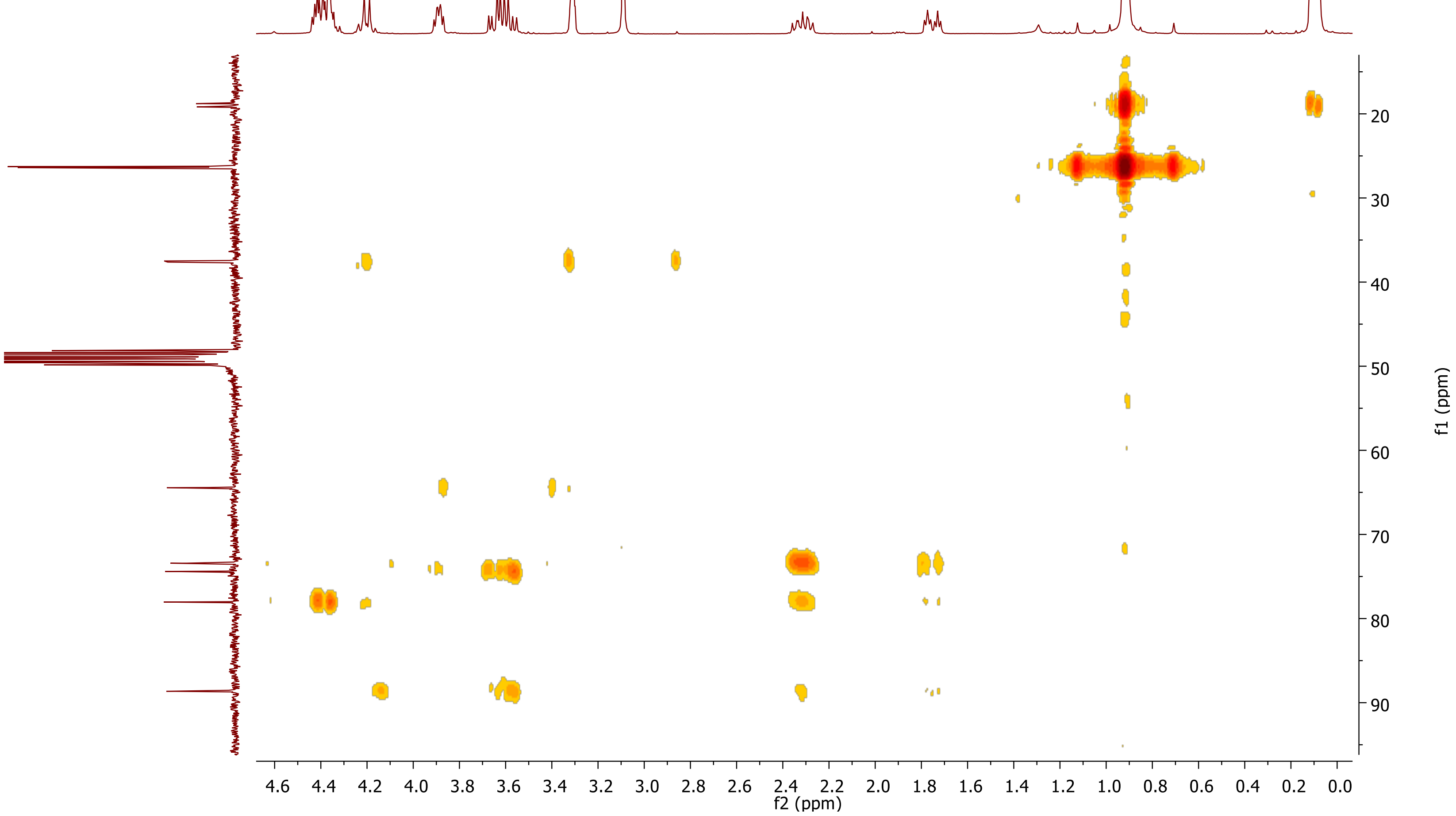

3,5-bis-*O*-(*tert*-Butyldimethylsilyl)-1'-homo-*N*-2'-deoxy-α-adenosine (17a)

^{1}H NMR (300.13 MHz, MeOH-d_4)

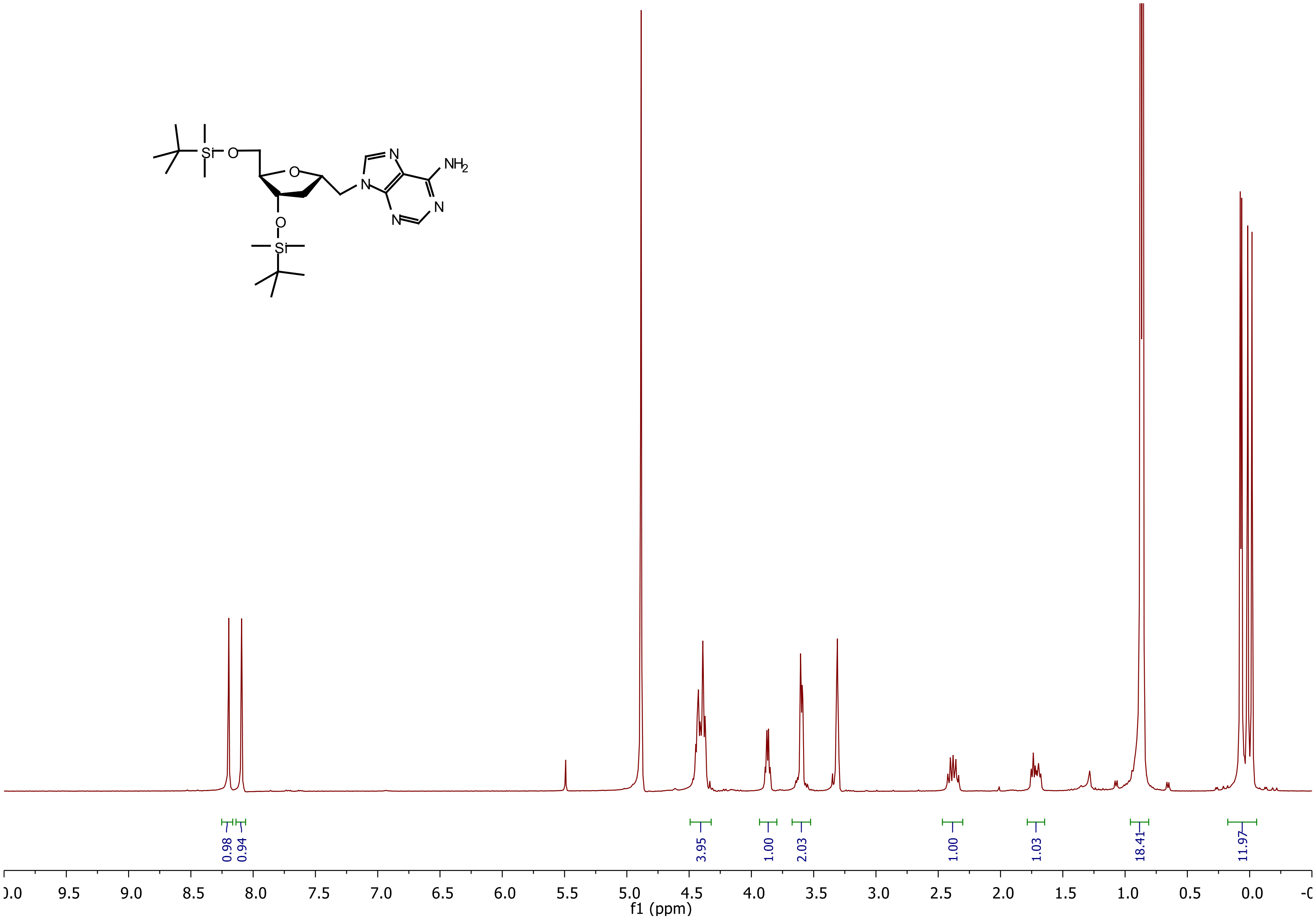

3,5-bis-*O*-(*tert*-Butyldimethylsilyl)-1'-homo-*N*-2'-deoxy-α-adenosine (17a)

^{13}C NMR (75.5 MHz, MeOH-d_4)

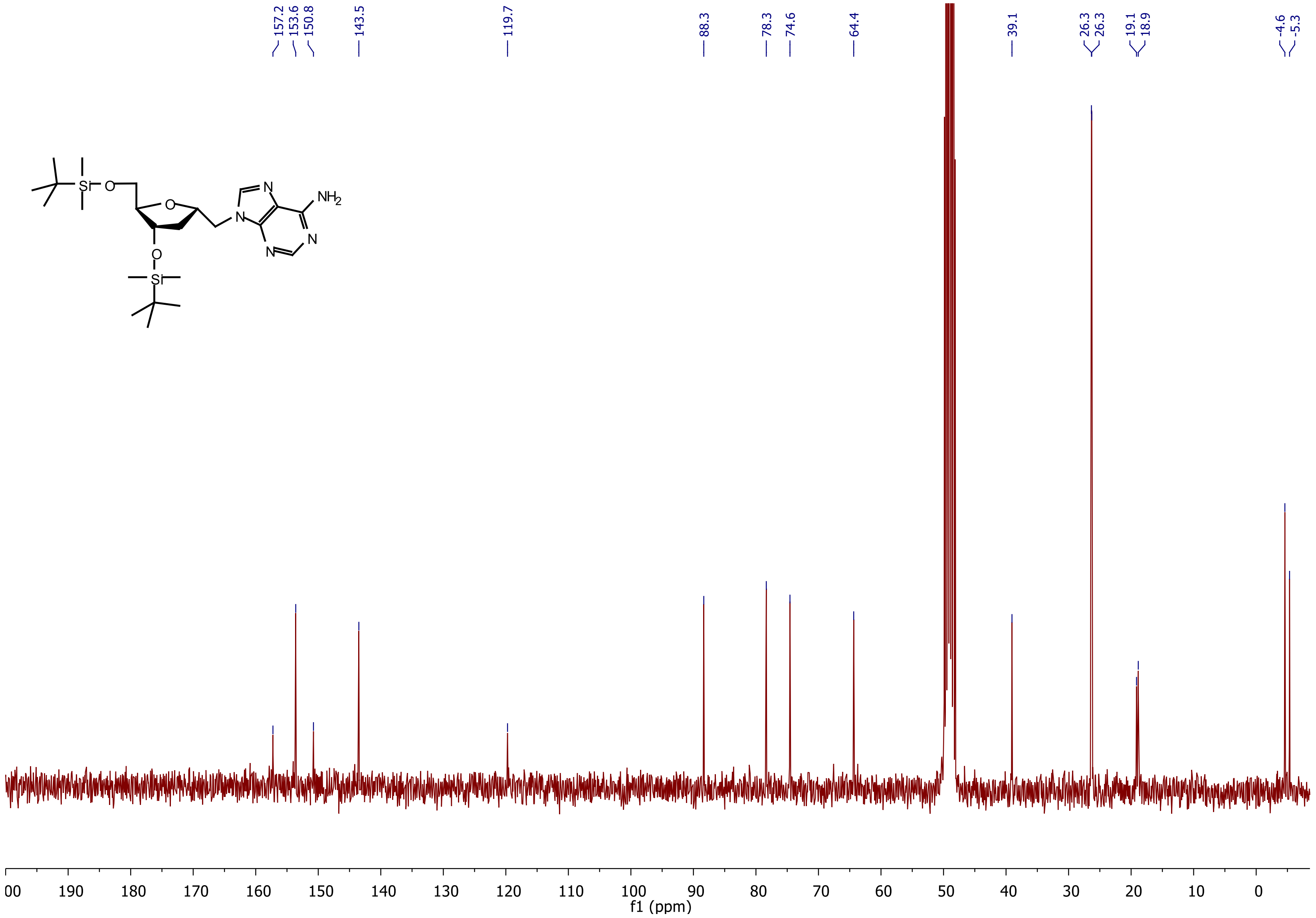

3,5-bis-*O*-(*tert*-Butyldimethylsilyl)-1'-homo-*N*-2'-deoxy-α-adenosine (17a)

DEPT NMR (75.5 MHz, MeOH-d_4)

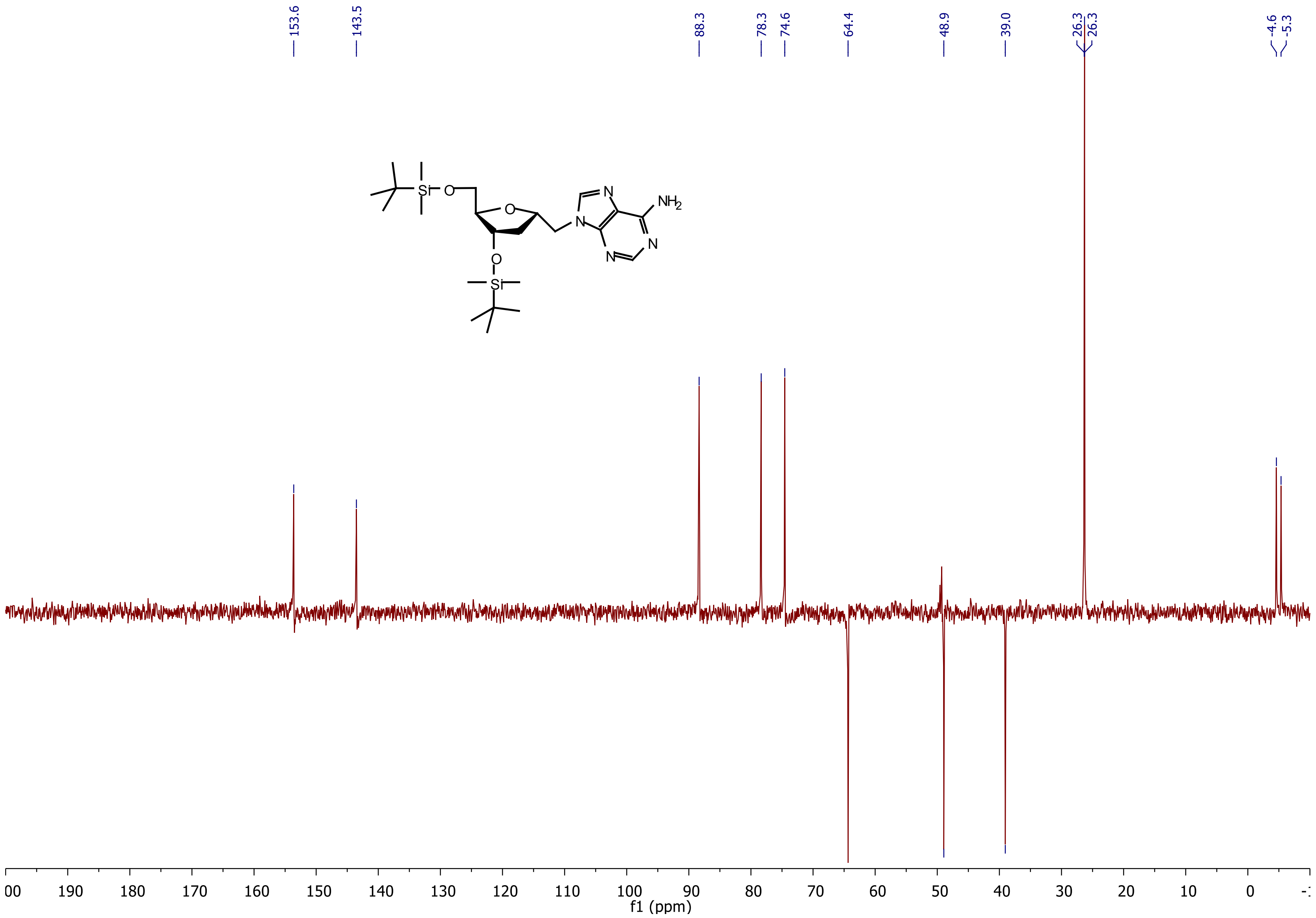

3,5-bis-*O*-(*tert*-Butyldimethylsilyl)-1’-homo-*N*-2’-deoxy-α-adenosine (17a)

COSY NMR (MeOH-d_4)

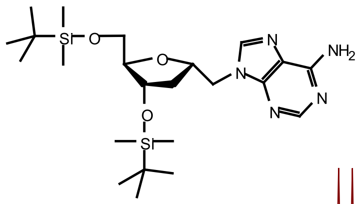

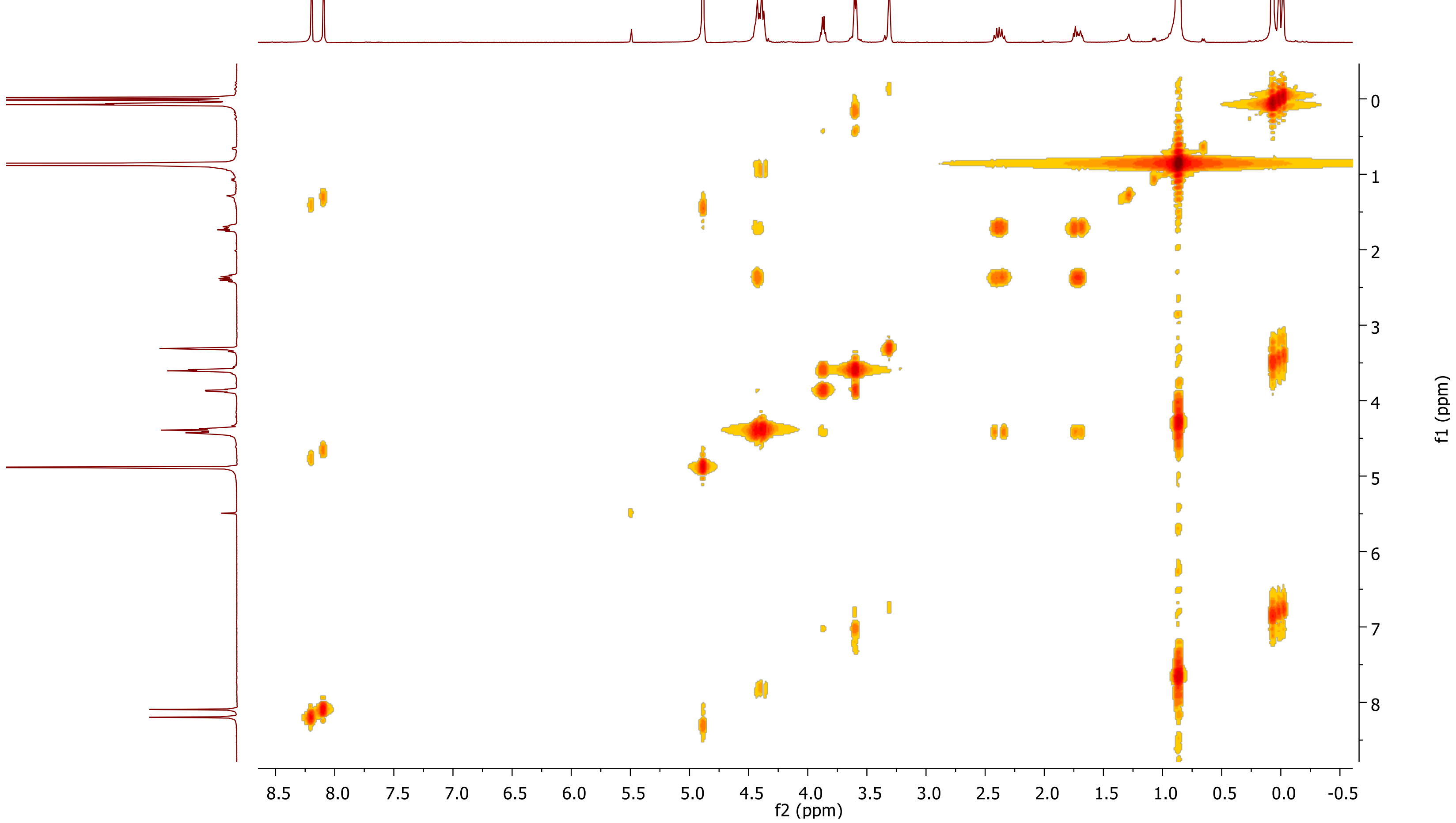

3,5-bis-*O*-(*tert*-Butyldimethylsilyl)-1'-homo-*N*-2'-deoxy-α-adenosine (17a)

HSQC NMR (MeOH-d_4)

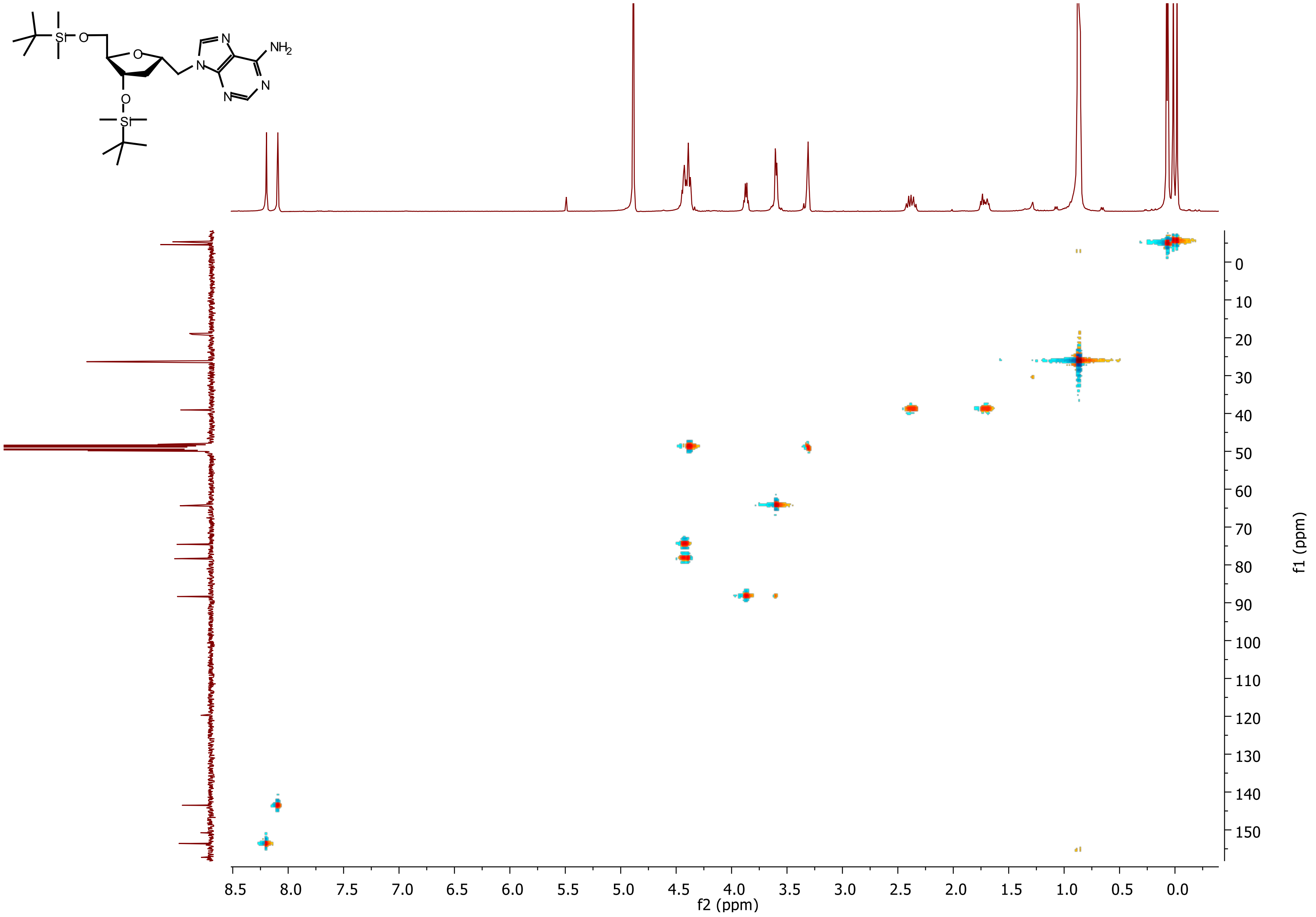

3,5-bis-*O*-(*tert*-Butyldimethylsilyl)-1’-homo-*N*-2’-deoxy-α-adenosine (17a)

HMBC NMR (MeOH-d_4)

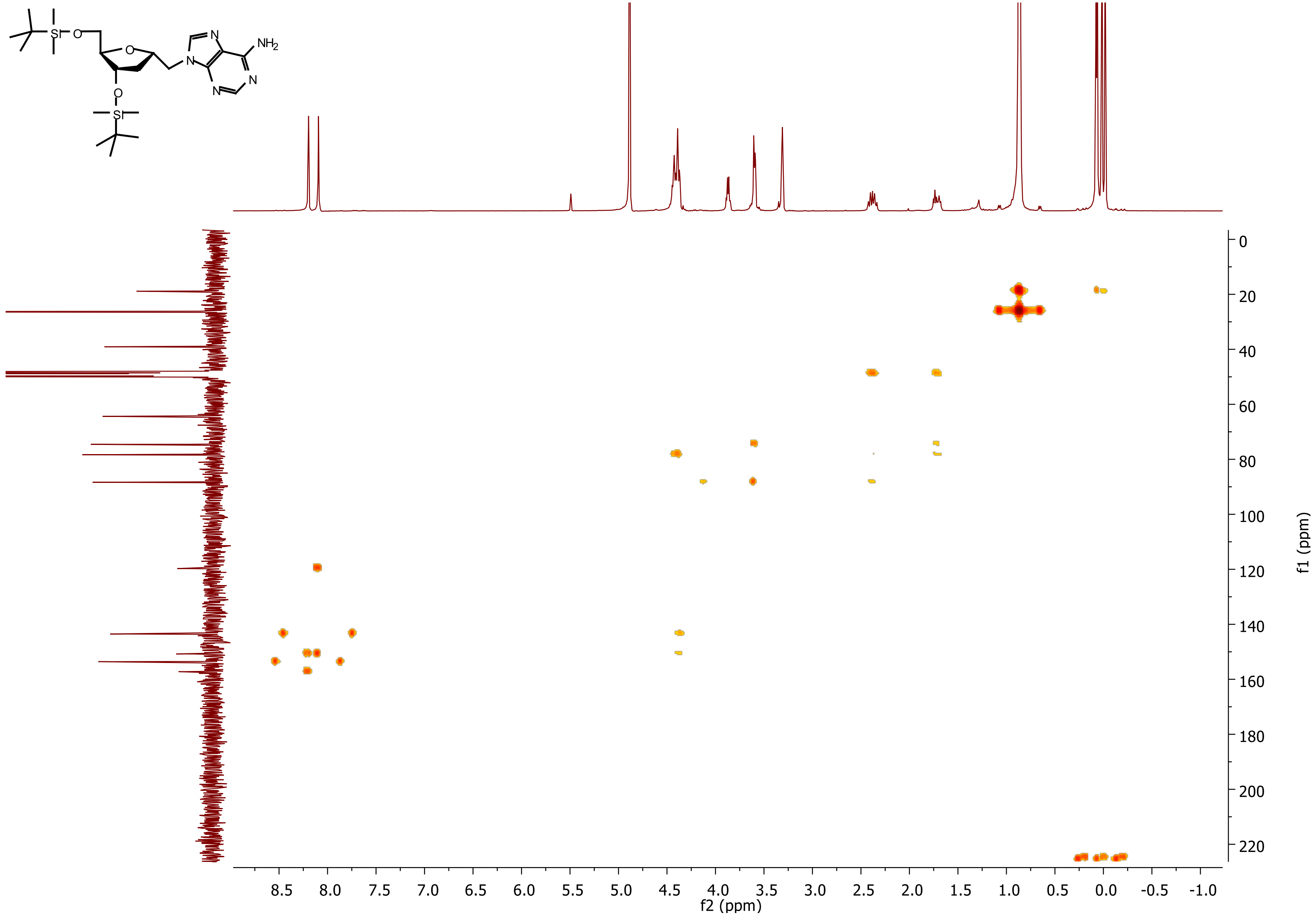

3,5-bis-*O*-(*tert*-Butyldimethylsilyl)-1’-homo-*N*-2’-deoxy-α-uridine (17b)

^{1}H NMR (300.13 MHz, MeOH-d_4)

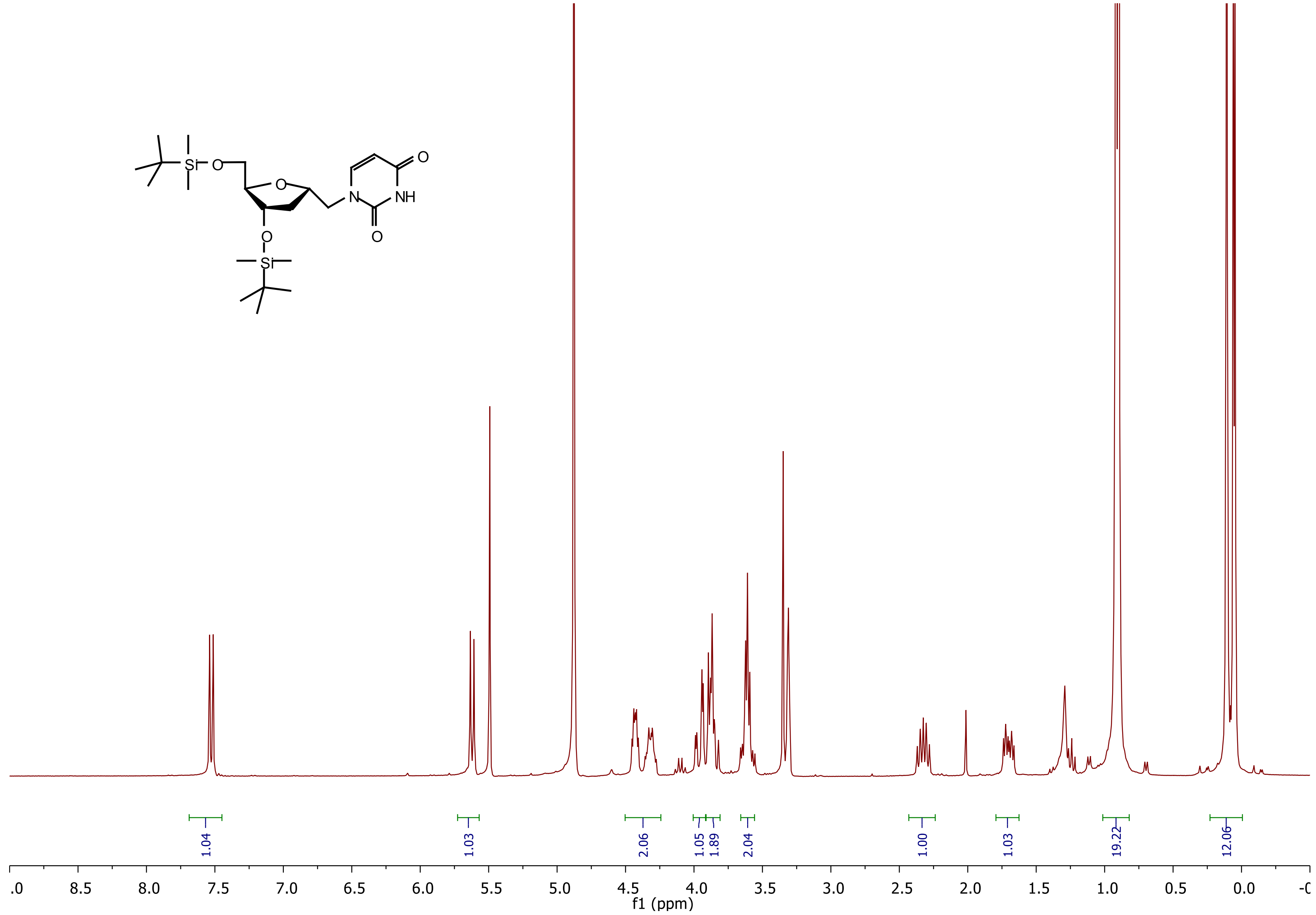

3,5-bis-*O*-(*tert*-Butyldimethylsilyl)-1'-homo-*N*-2'-deoxy-α-uridine (17b)

^{13}C NMR (75.5 MHz, MeOH-d_4)

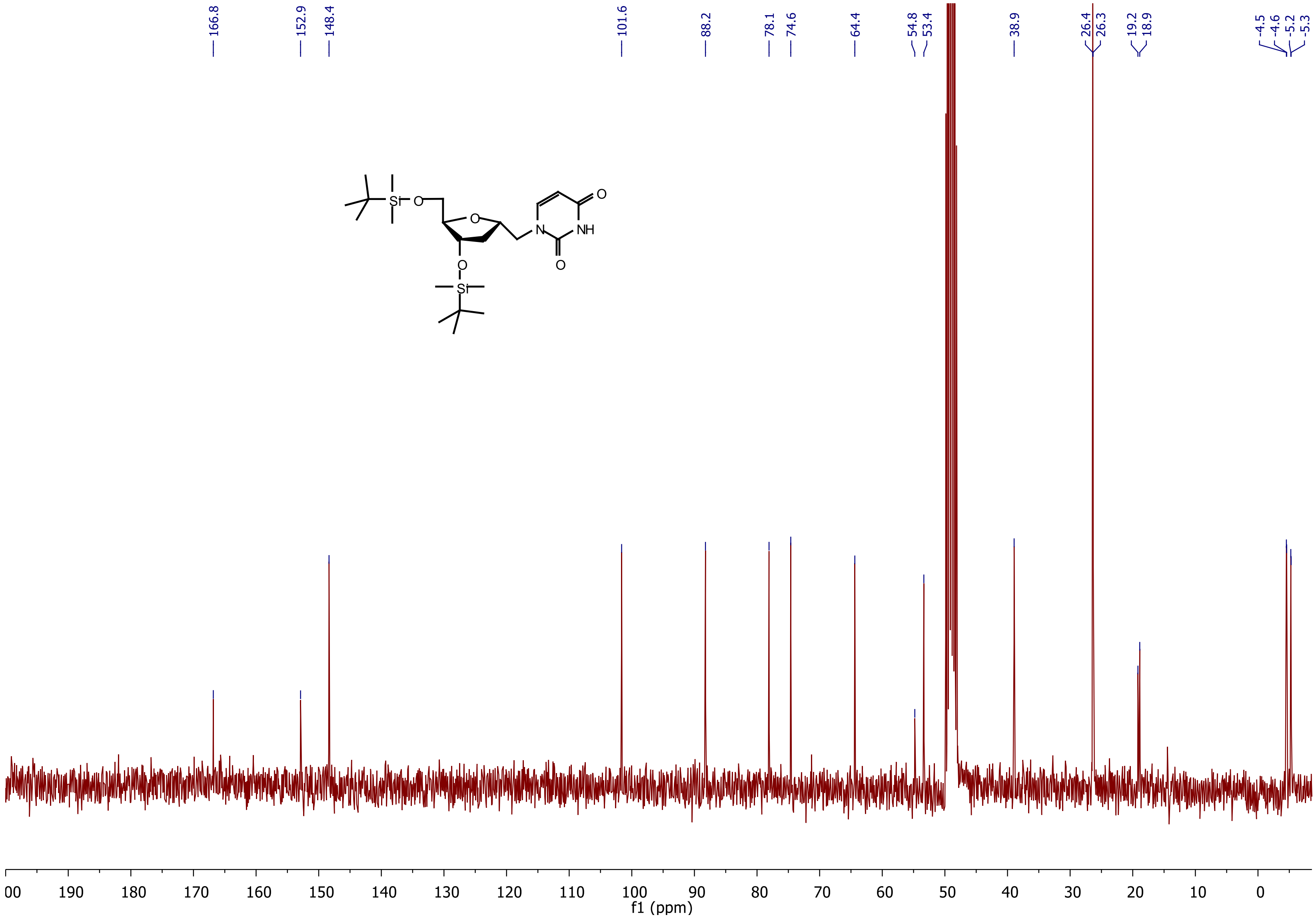

3,5-bis-*O*-(*tert*-Butyldimethylsilyl)-1'-homo-*N*-2'-deoxy-α-uridine (17b)

DEPT NMR (75.5 MHz, MeOH-d_4)

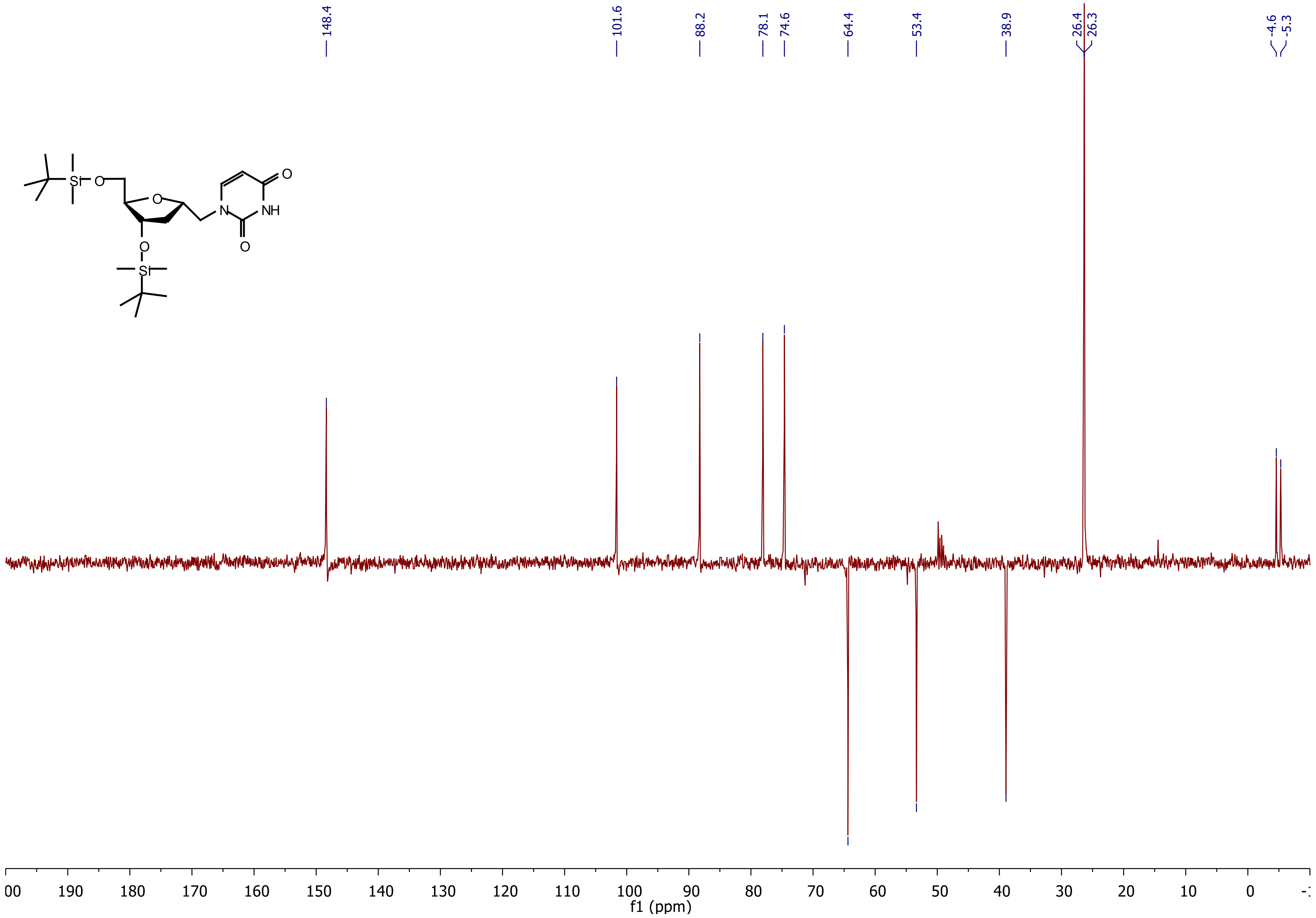

3,5-bis-*O*-(*tert*-Butyldimethylsilyl)-1'-homo-*N*-2'-deoxy-α-uridine (17b)

COSY NMR (MeOH-d_4)

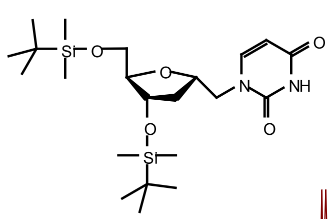

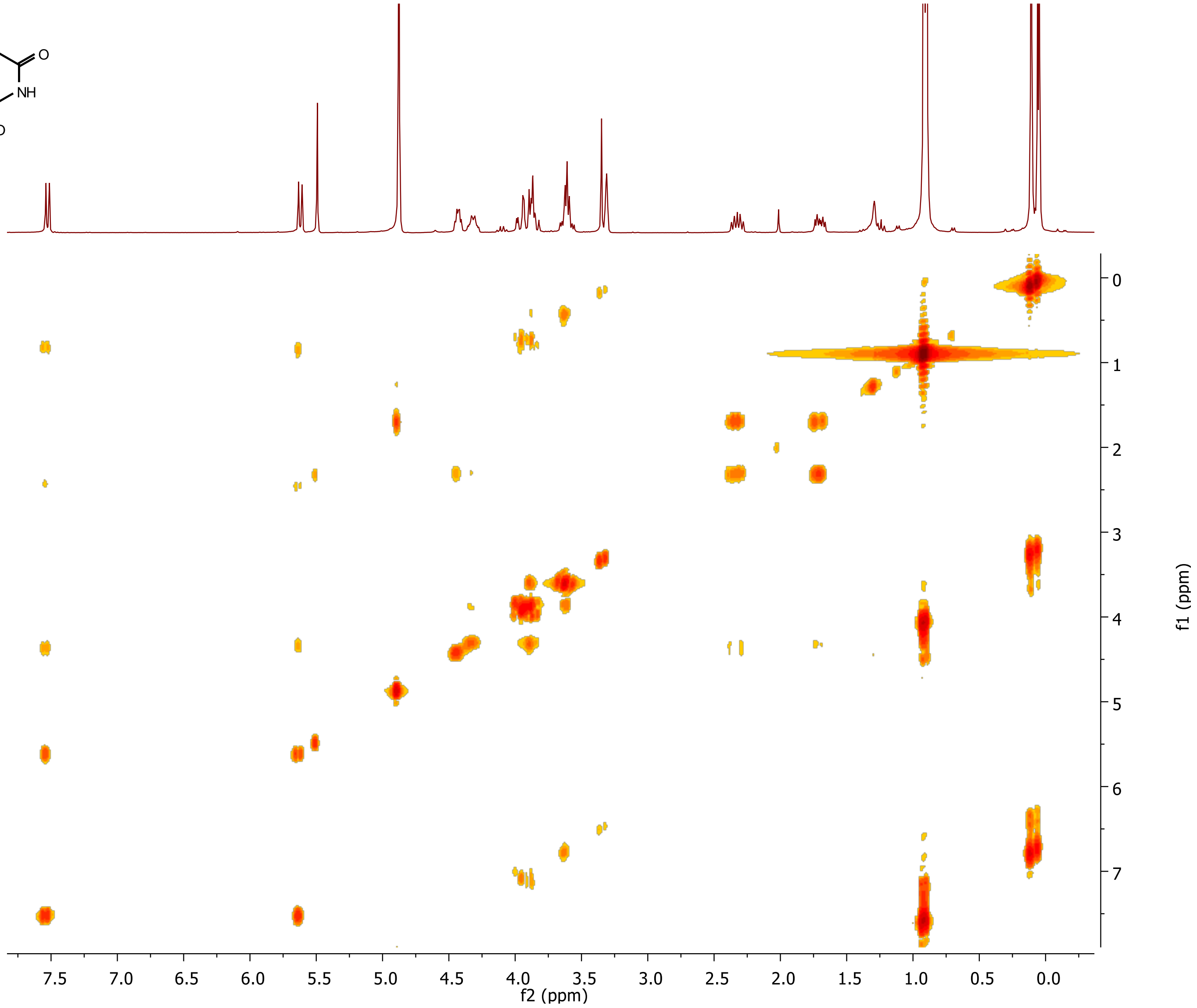

3,5-bis-*O*-(*tert*-Butyldimethylsilyl)-1'-homo-*N*-2'-deoxy-α-uridine (17b)

HSQC NMR (MeOH-d_4)

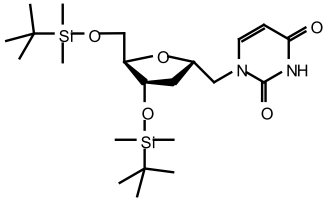

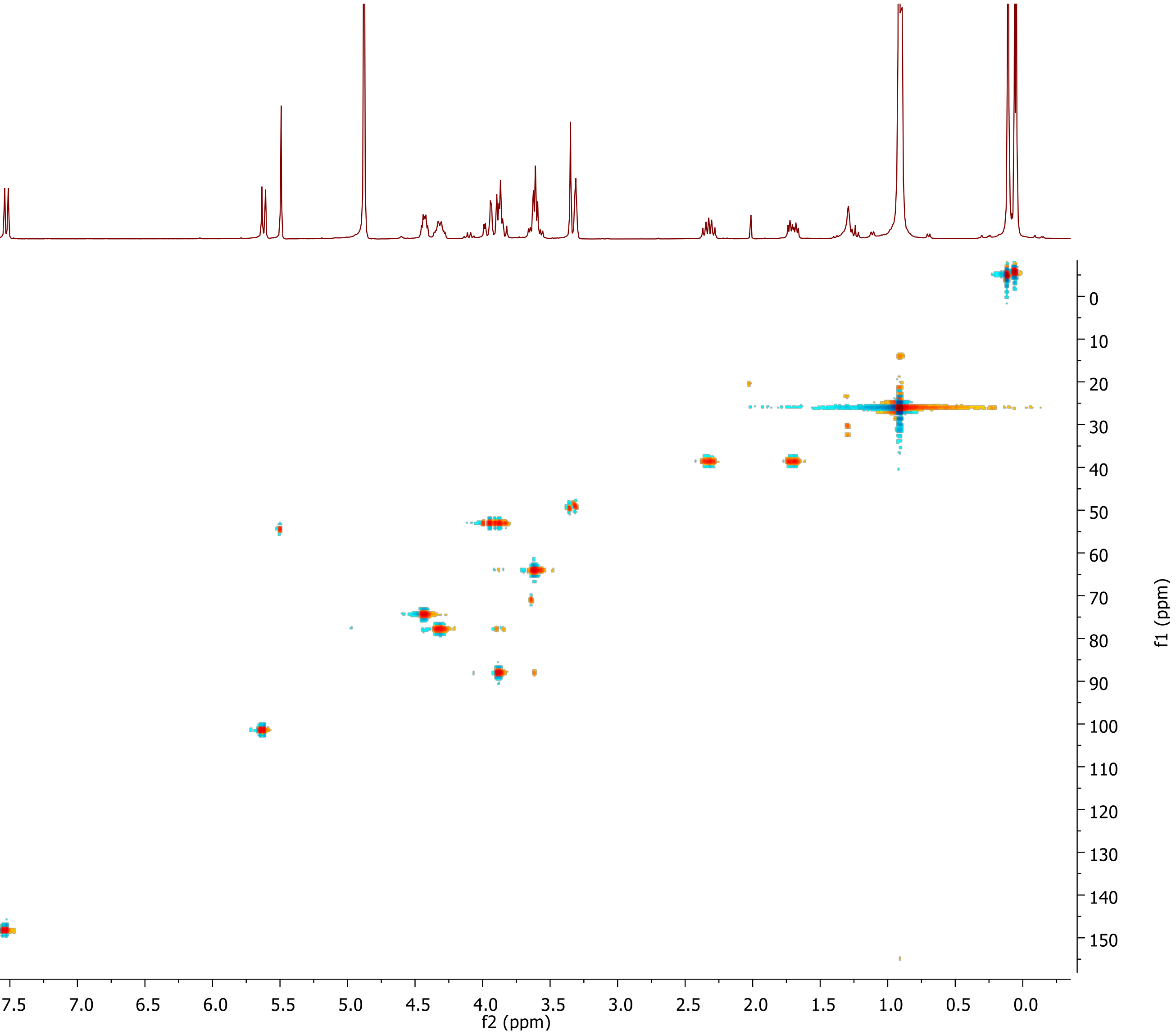

3,5-bis-*O*-(*tert*-Butyldimethylsilyl)-1'-homo-*N*-2'-deoxy-α-uridine (17b)

HMBC NMR (MeOH-d_4)

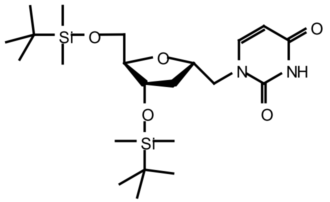

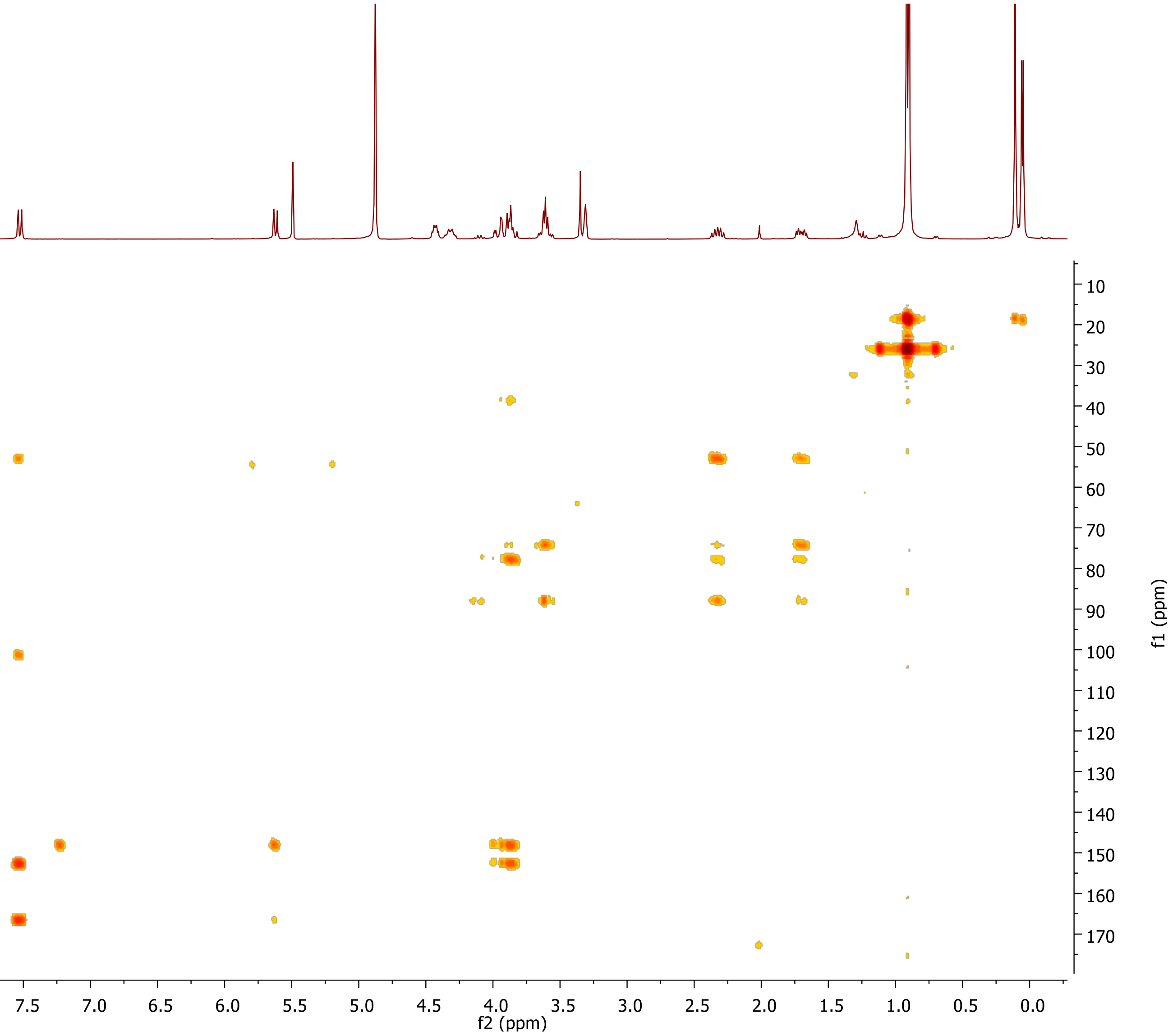

3,5-bis-*O*-(*tert*-Butyldimethylsilyl)-1’-homo-*N*-α-thymidine (17c)

^{1}H NMR (300.13 MHz, MeOH-d_4)

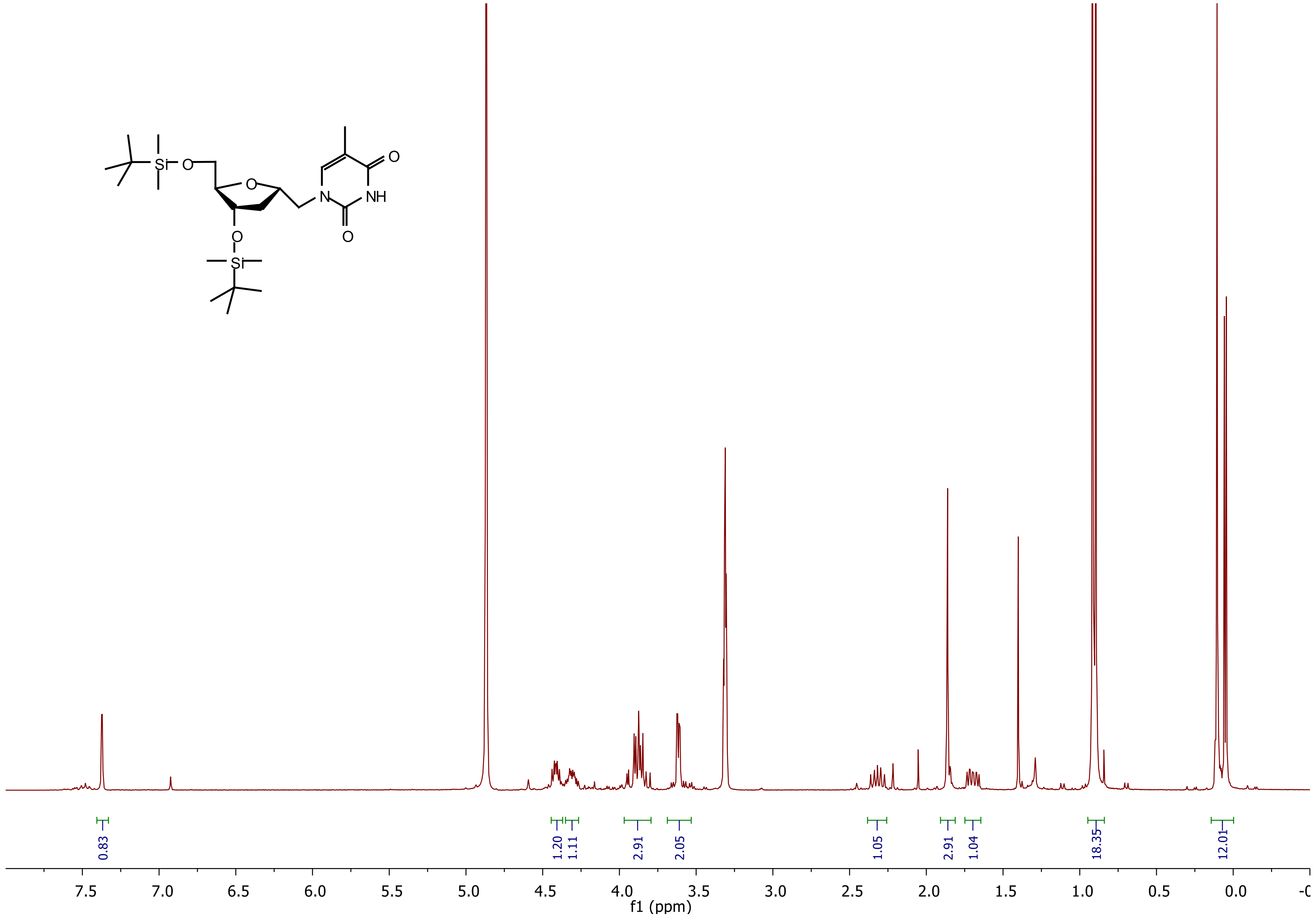

3,5-bis-*O*-(*tert*-Butyldimethylsilyl)-1'-homo-*N*-α-thymidine (17c)

^{13}C NMR (75.5 MHz, MeOH-d_4)

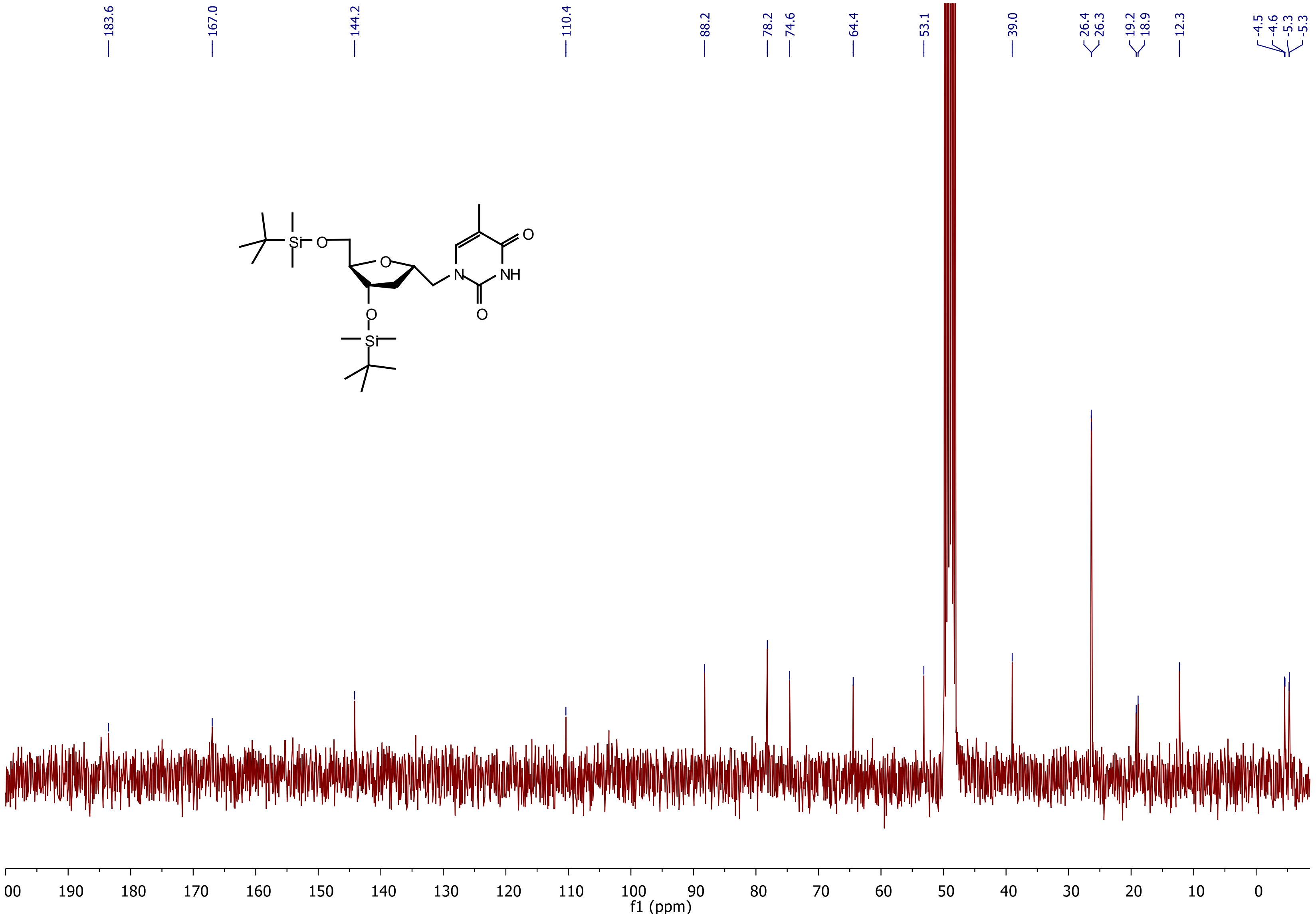

3,5-bis-*O*-(*tert*-Butyldimethylsilyl)-1'-homo-*N*-α-thymidine (17c)

DEPT NMR (75.5 MHz, MeOH-d_4)

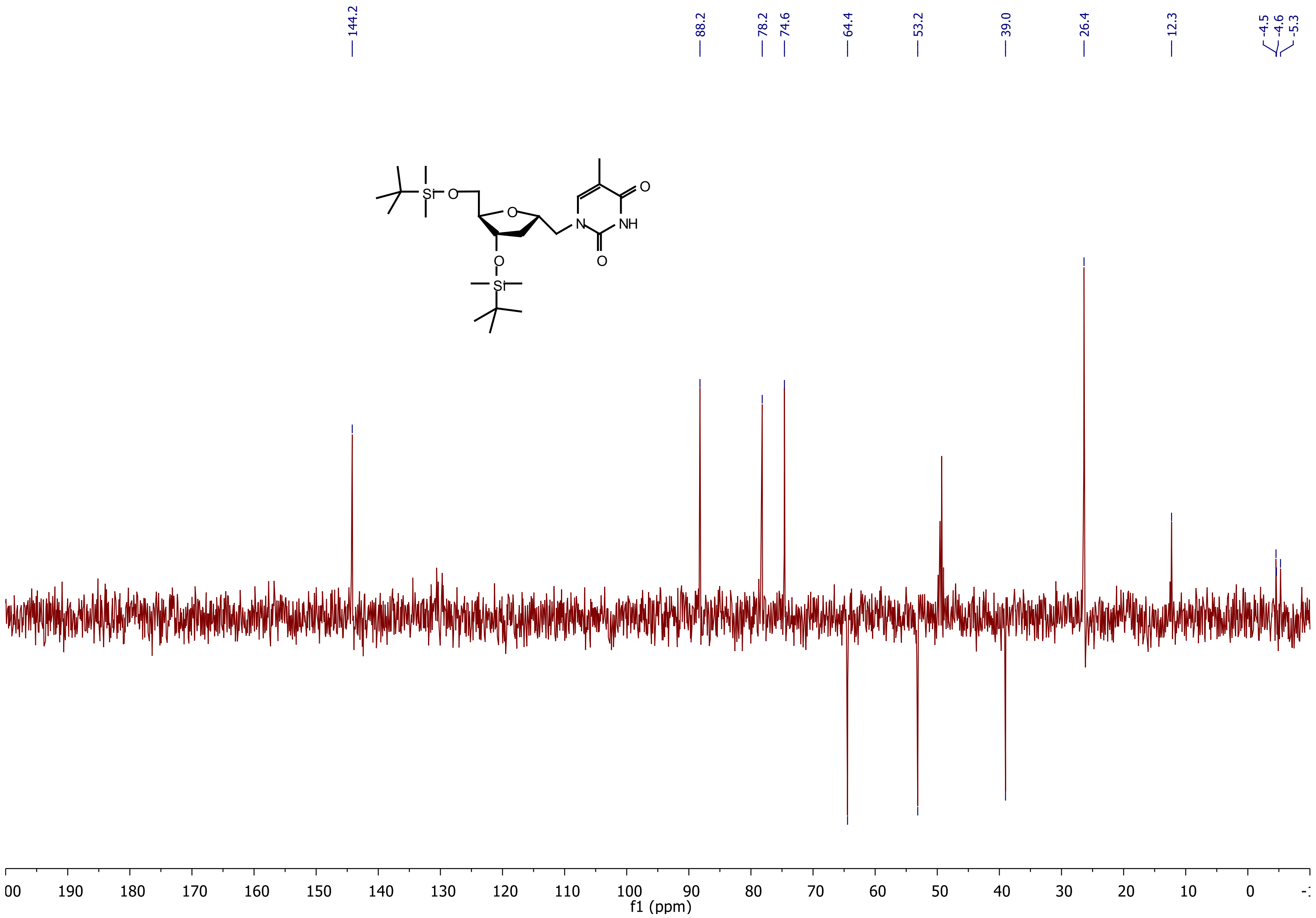

3,5-bis-*O*-(*tert*-Butyldimethylsilyl)-1'-homo-*N*-α-thymidine (17c)

COSY NMR (MeOH-d_4)

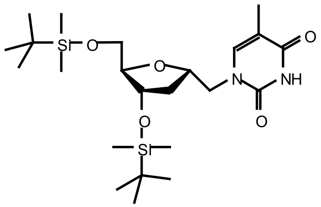

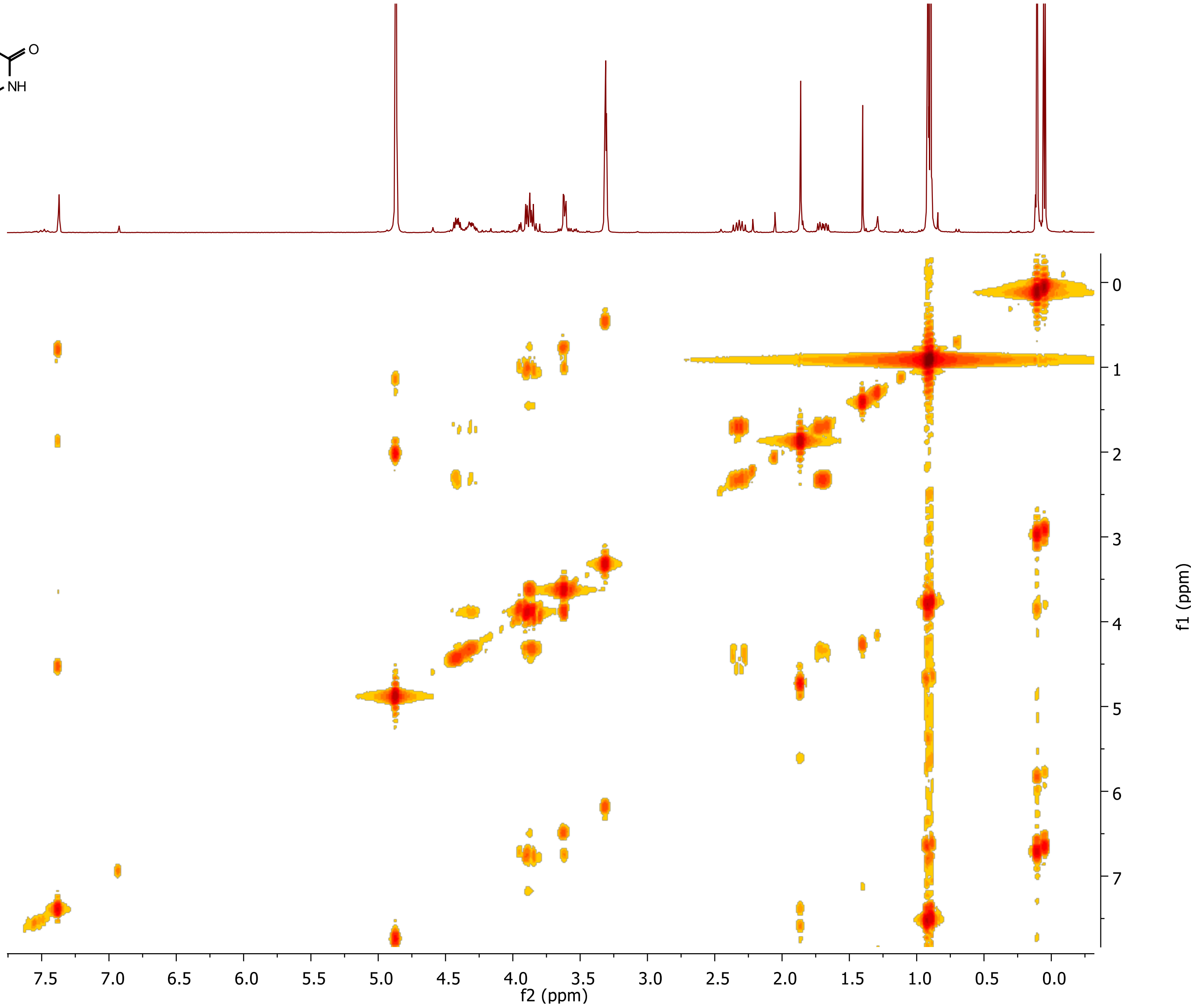

3,5-bis-*O*-(*tert*-Butyldimethylsilyl)-1'-homo-*N*-α-thymidine (17c)

HSQC NMR (MeOH-d_4)

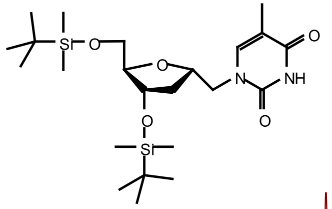

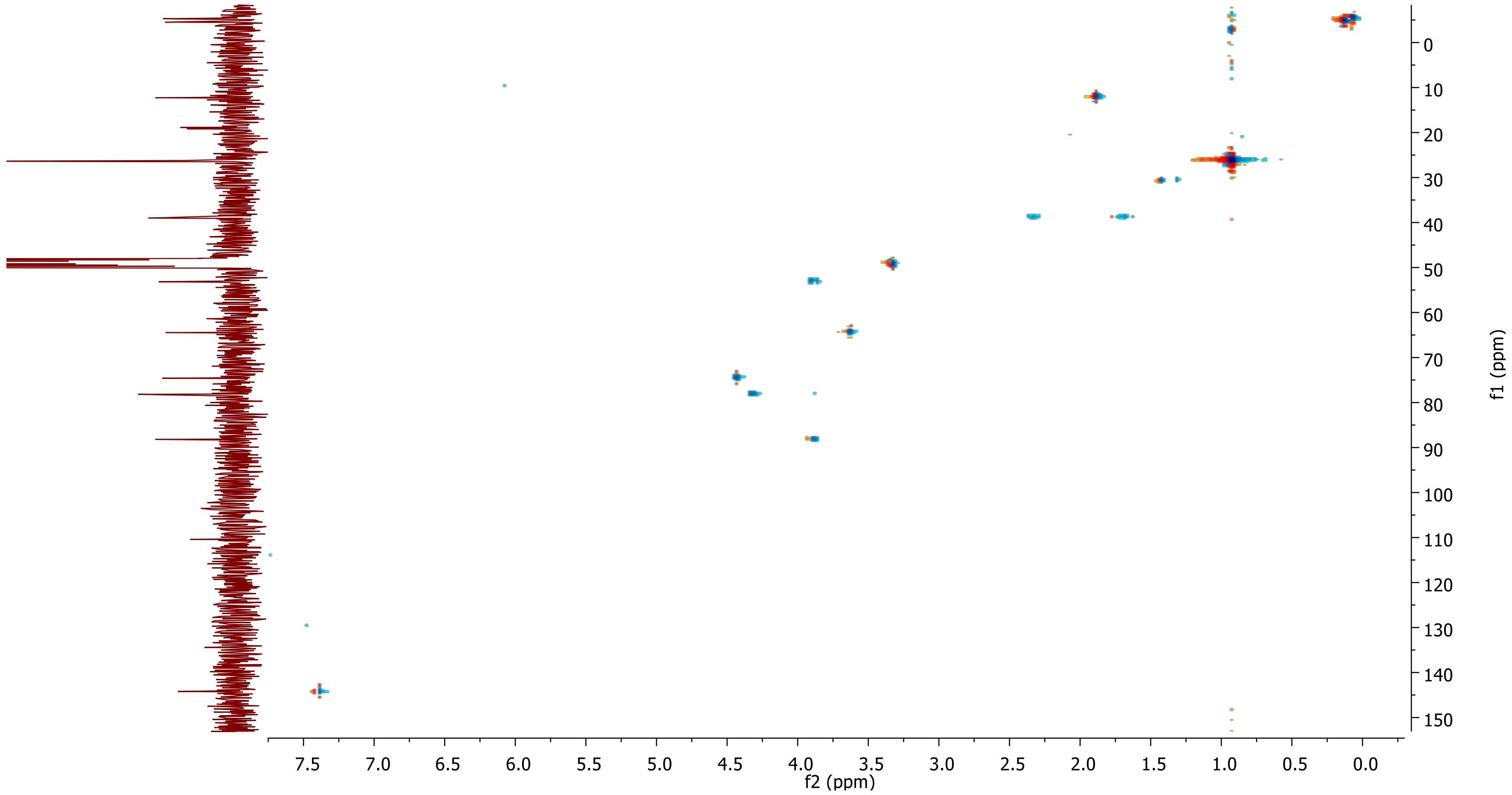

3,5-bis-*O*-(*tert*-Butyldimethylsilyl)-1’-homo-*N*-α-thymidine (17c)

HMBC NMR (MeOH-d_4)

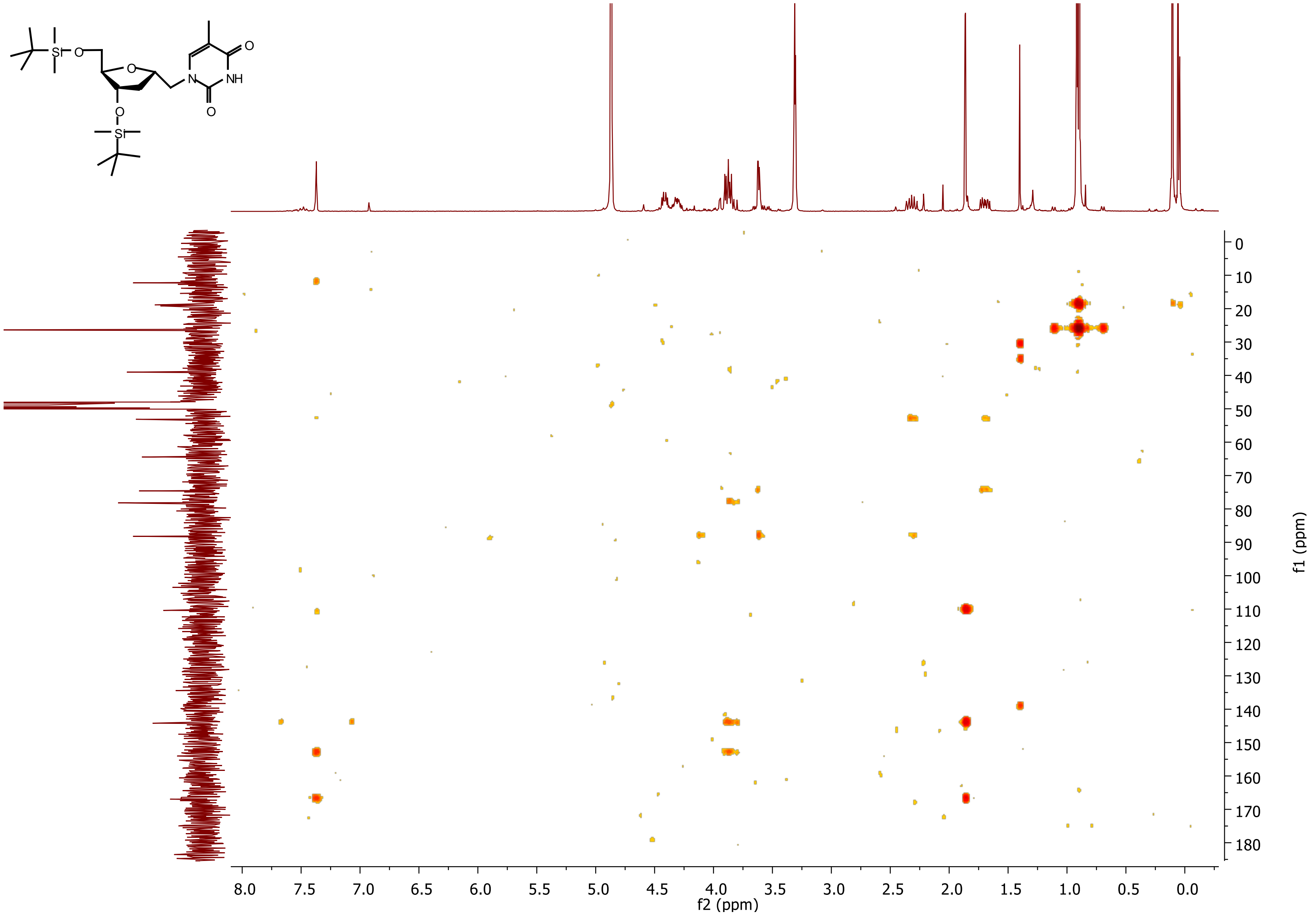

3,5-bis-*O*-(*tert*-Butyldimethylsilyl)-1'-homo-*N*-2'-deoxy-α-5-fluorouridine (17d)

^{1}H NMR (300.13 MHz, MeOH-d_4)

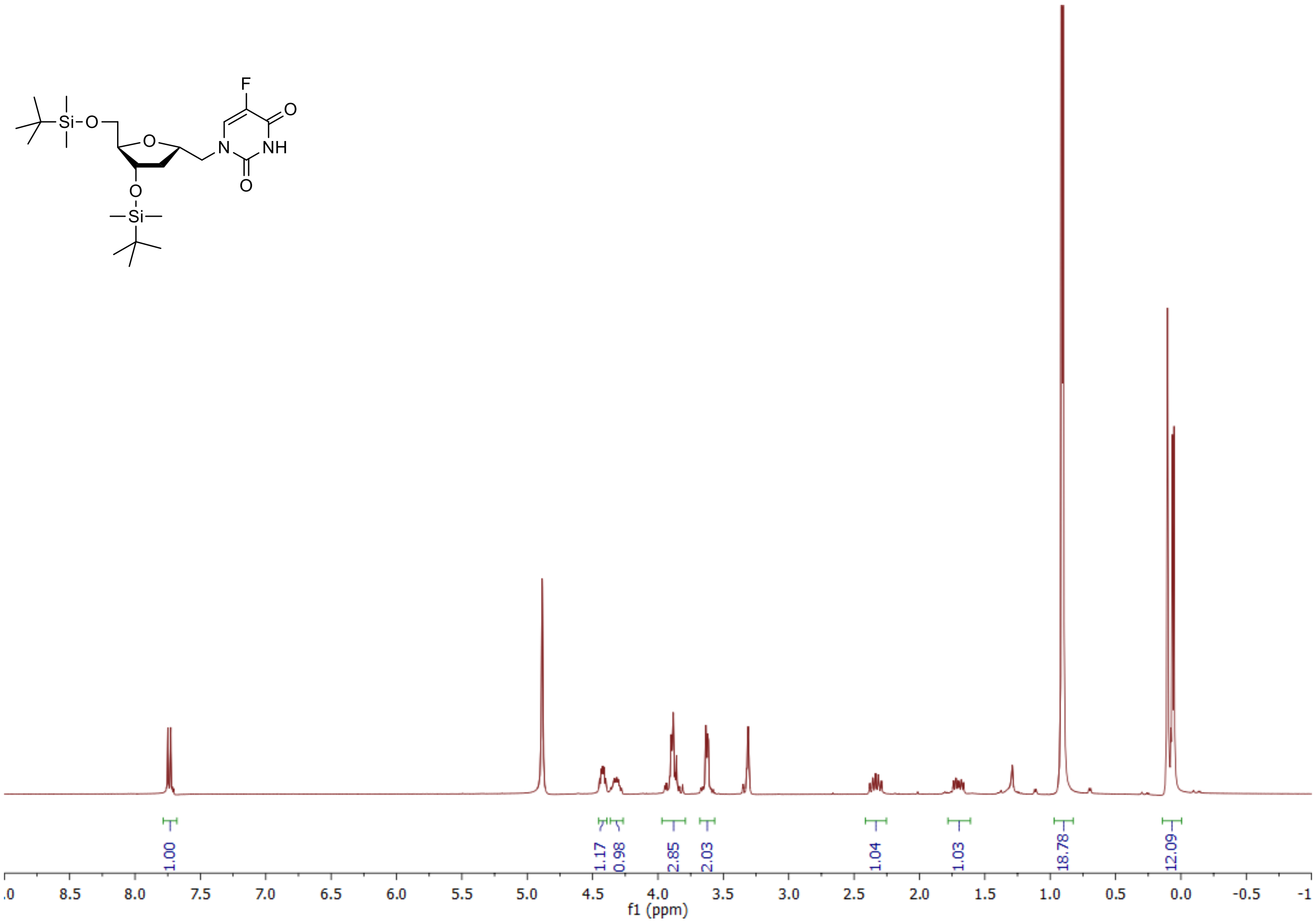

3,5-bis-*O*-(*tert*-Butyldimethylsilyl)-1'-homo-*N*-2'-deoxy-α-5-fluorouridine (17d)

^{13}C NMR (75.5 MHz, MeOH-d_4)

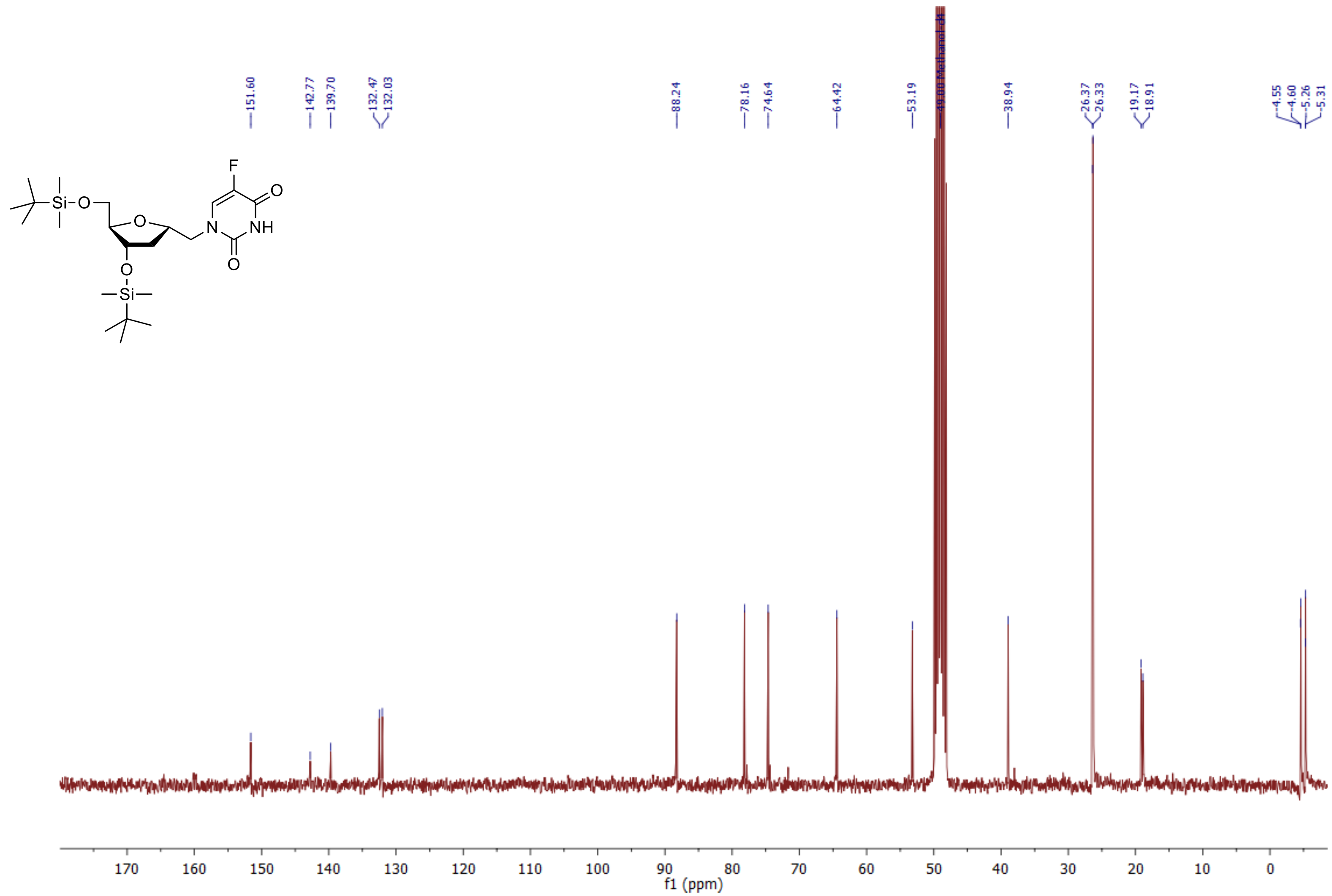

3,5-bis-*O*-(*tert*-Butyldimethylsilyl)-1'-homo-*N*-2'-deoxy-α-5-fluorouridine (17d)

DEPT 135 (75.5 MHz, MeOH-d_4)

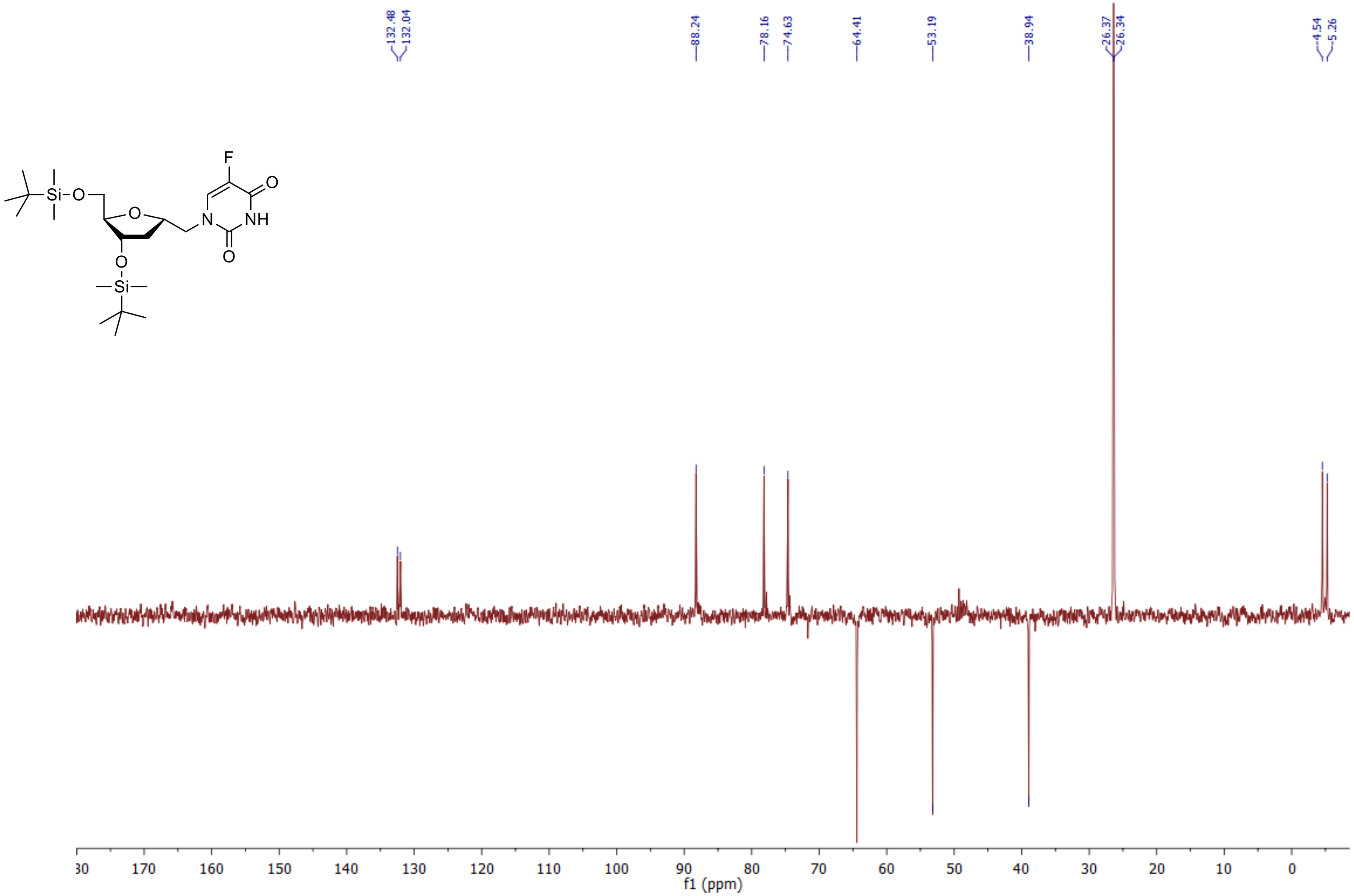

3,5-bis-*O*-(*tert*-Butyldimethylsilyl)-1'-homo-*N*-2'-deoxy-α-5-fluorouridine (17d)

COSY NMR (MeOH-d_4)

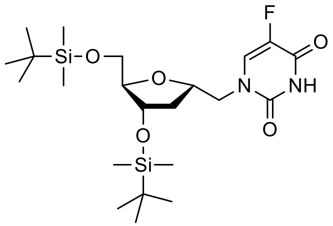

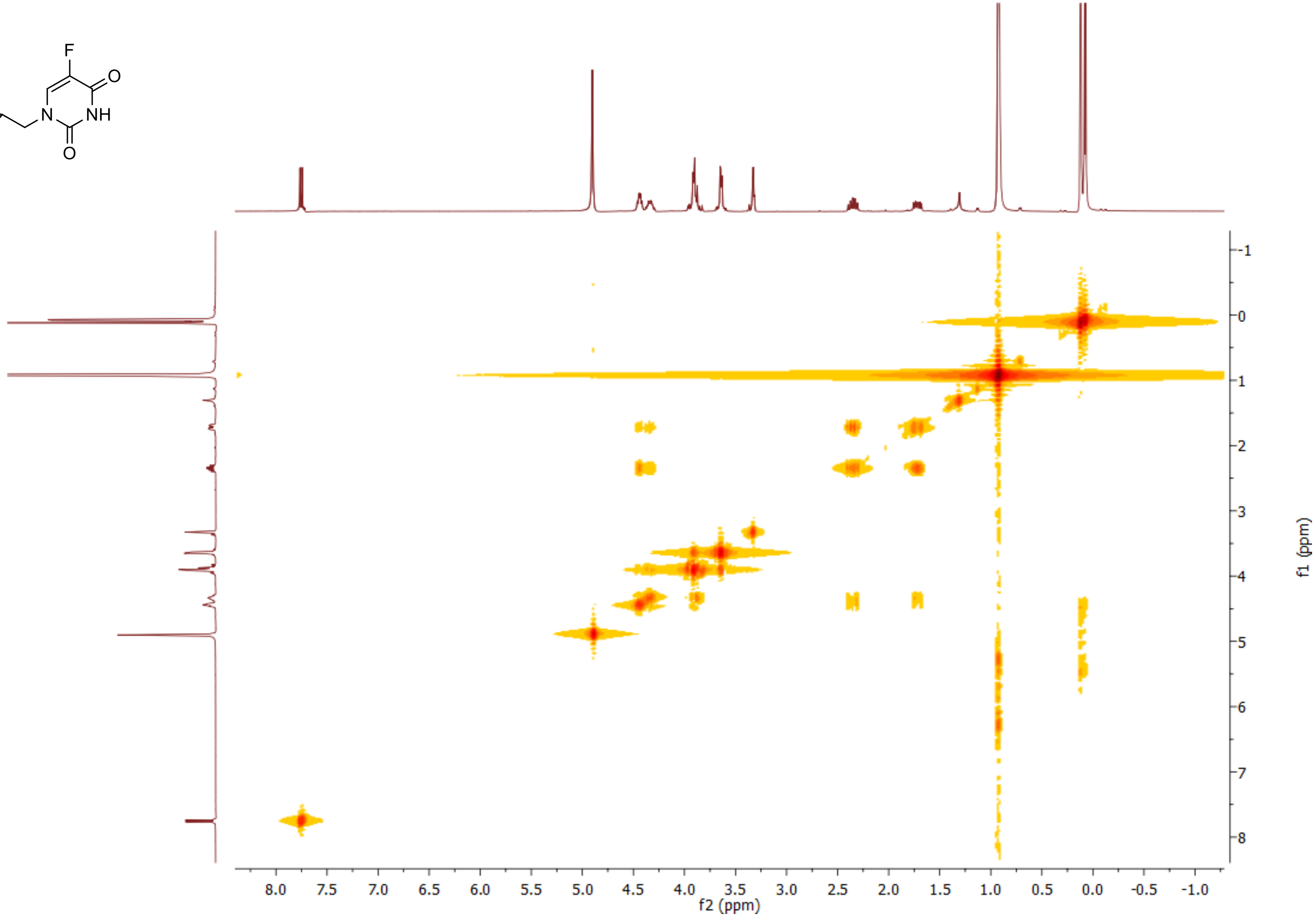

3,5-bis-*O*-(*tert*-Butyldimethylsilyl)-1'-homo-*N*-2'-deoxy-α-5-fluorouridine (17d)

HSQC NMR (MeOH-d_4)

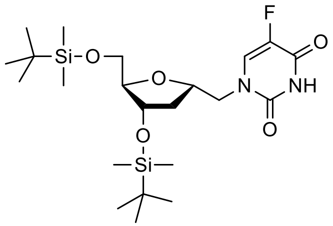

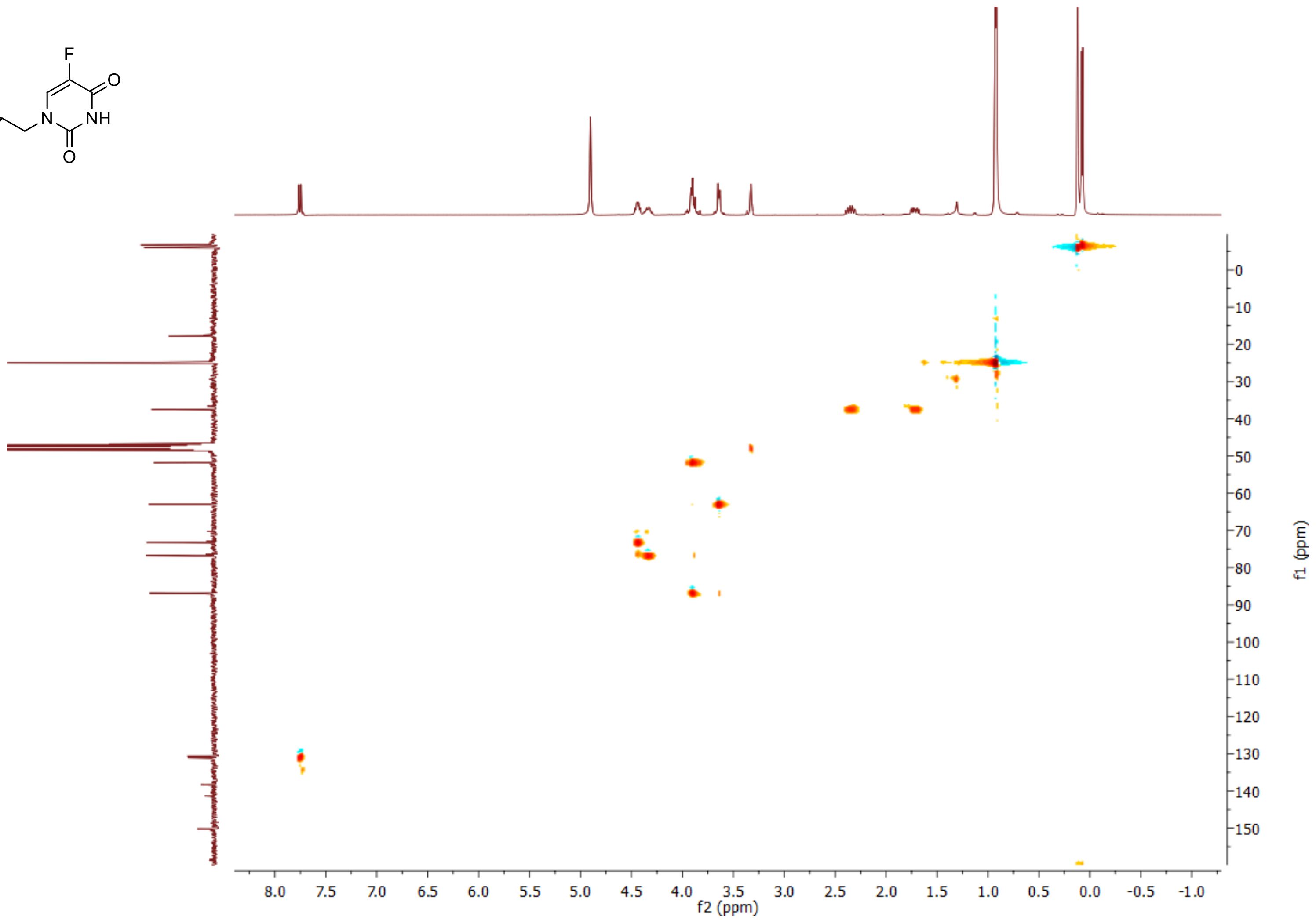

3,5-bis-*O*-(*tert*-Butyldimethylsilyl)-1'-homo-*N*-2'-deoxy-α-5-fluorouridine (17d)

HMBC NMR (MeOH-d_4)

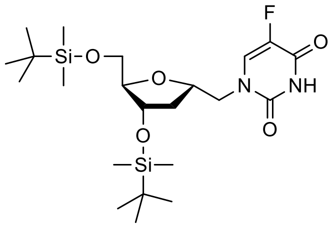

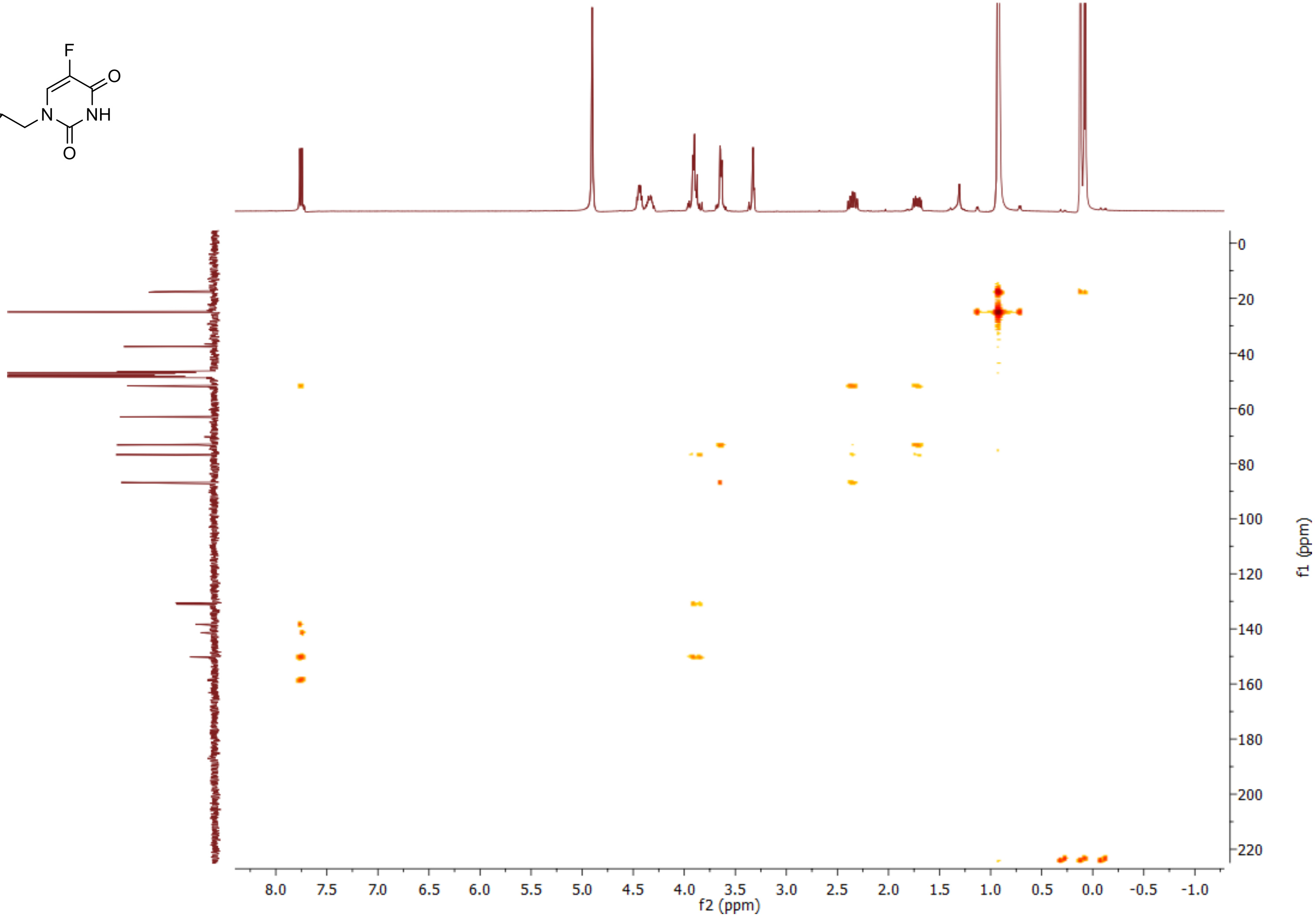

3,5-bis-*O*-(*tert*-Butyldimethylsilyl)-1’-homo-*N*-2’-deoxy-α-cytidine (17e)

^{1}H NMR (300.13 MHz, MeOH-d_4)

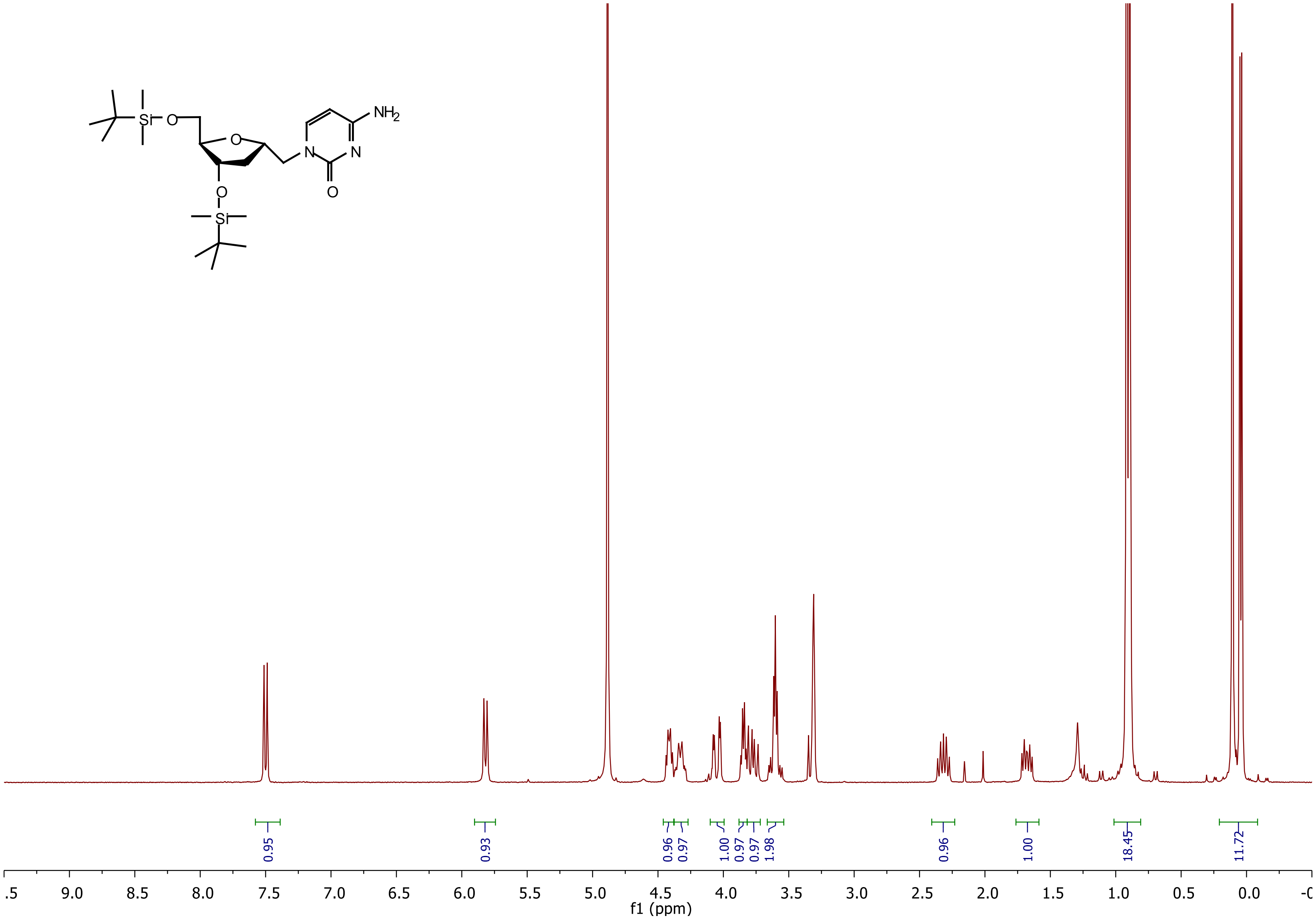

3,5-bis-*O*-(*tert*-Butyldimethylsilyl)-1'-homo-*N*-2'-deoxy-α-cytidine (17e)

^{13}C NMR (75.5 MHz, MeOH-d_4)

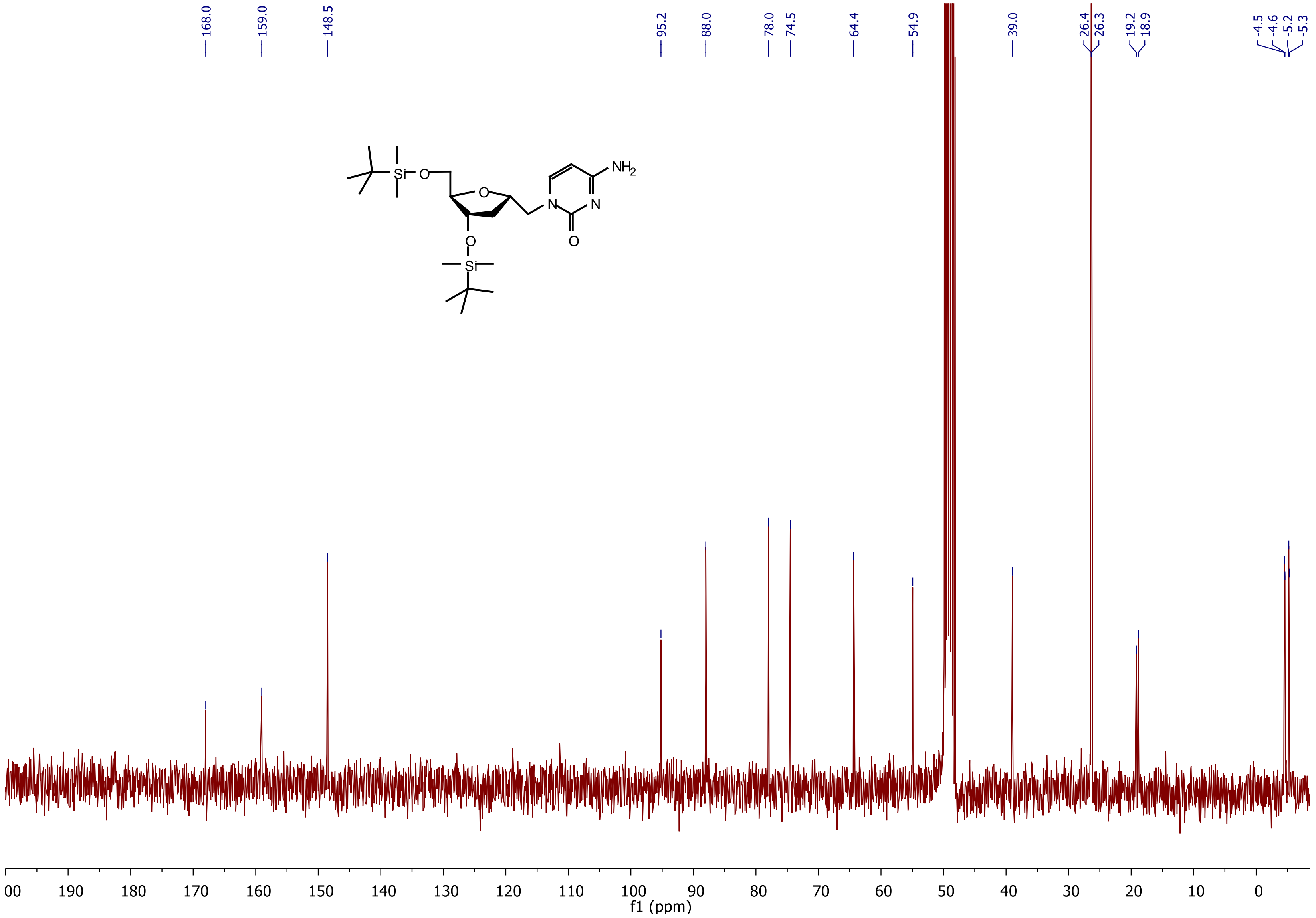

3,5-bis-*O*-(*tert*-Butyldimethylsilyl)-1’-homo-*N*-2’-deoxy-α-cytidine (17e)

DEPT NMR (75.5 MHz, MeOH-d_4)

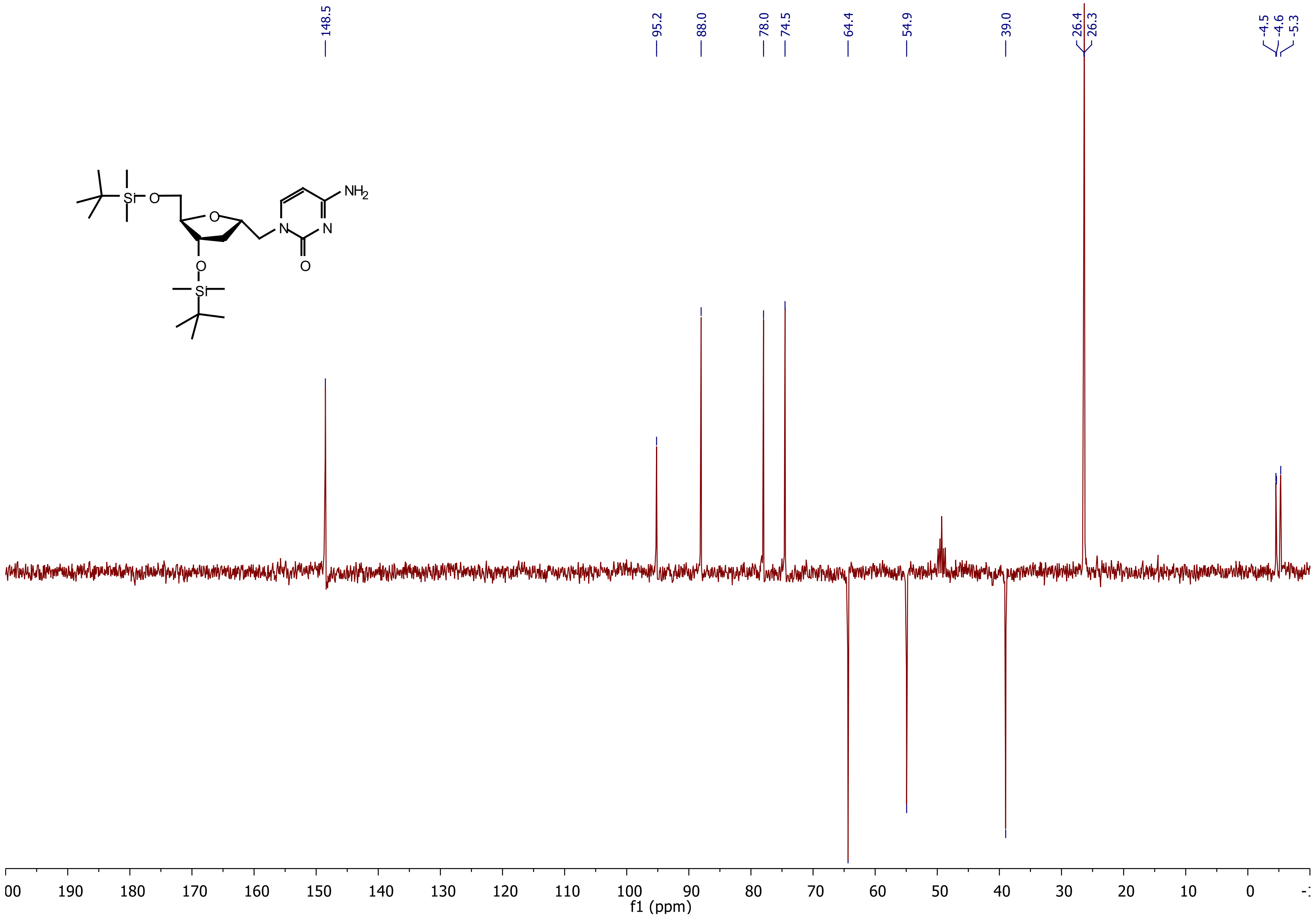

3,5-bis-*O*-(*tert*-Butyldimethylsilyl)-1’-homo-*N*-2’-deoxy-α-cytidine (17e)

COSY NMR (MeOH-d_4)

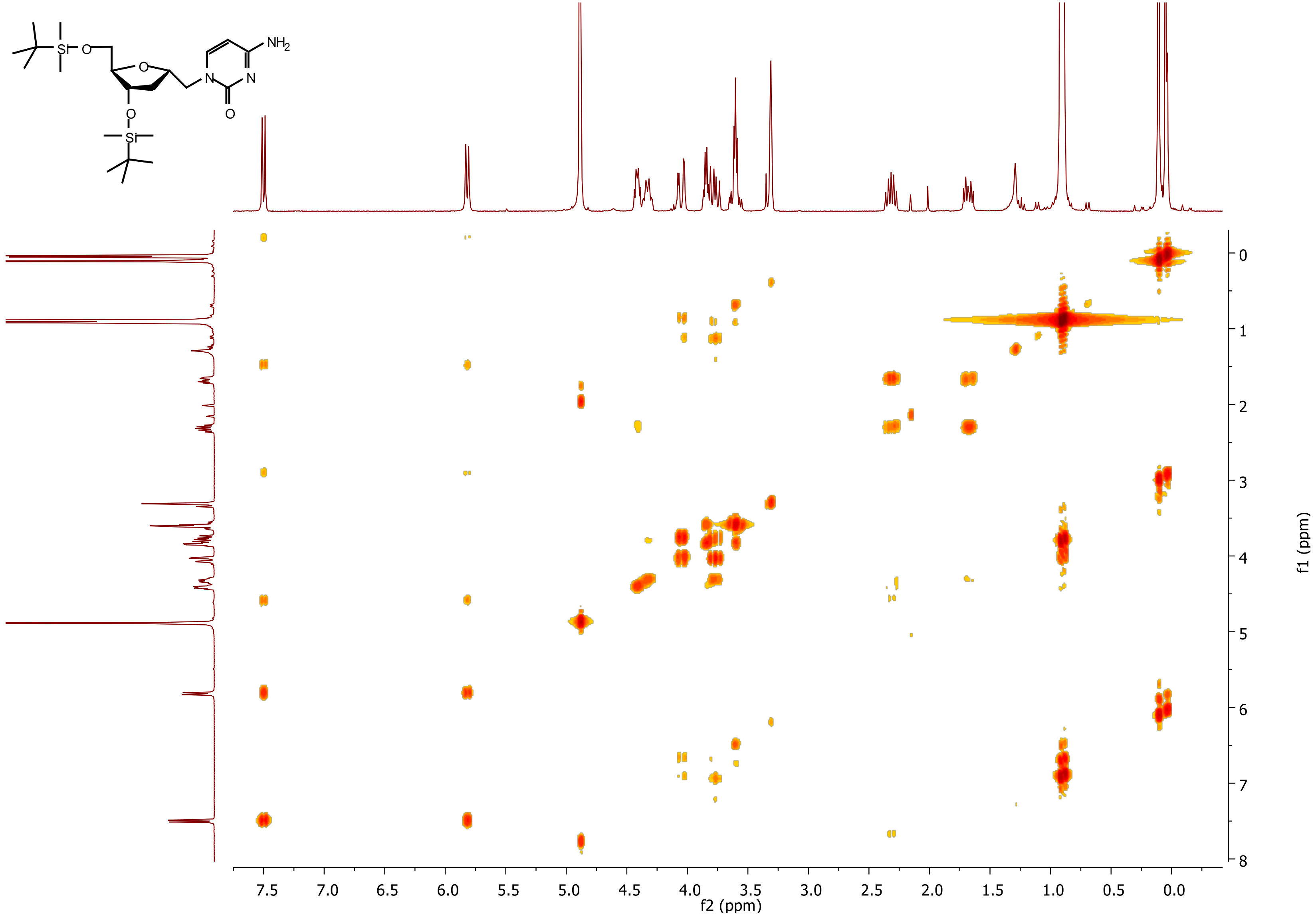

3,5-bis-*O*-(*tert*-Butyldimethylsilyl)-1’-homo-*N*-2’-deoxy-α-cytidine (17e)

HSQC NMR (MeOH-d_4)

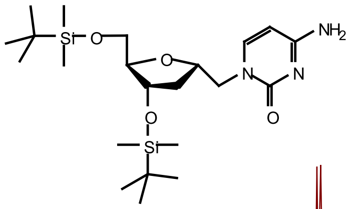

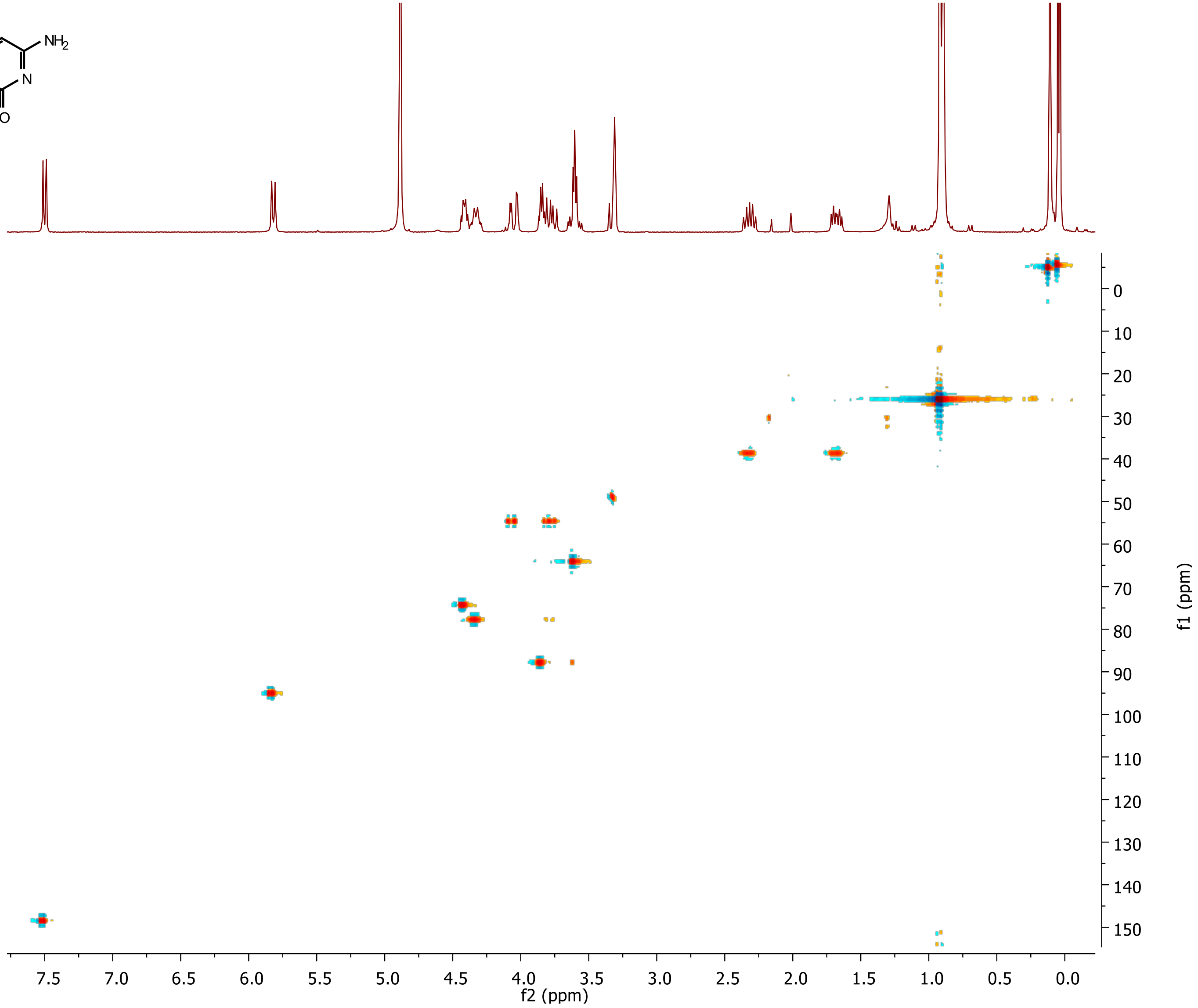

3,5-bis-*O*-(*tert*-Butyldimethylsilyl)-1’-homo-*N*-2’-deoxy-α-cytidine (17e)

HMBC NMR (MeOH-d_4)

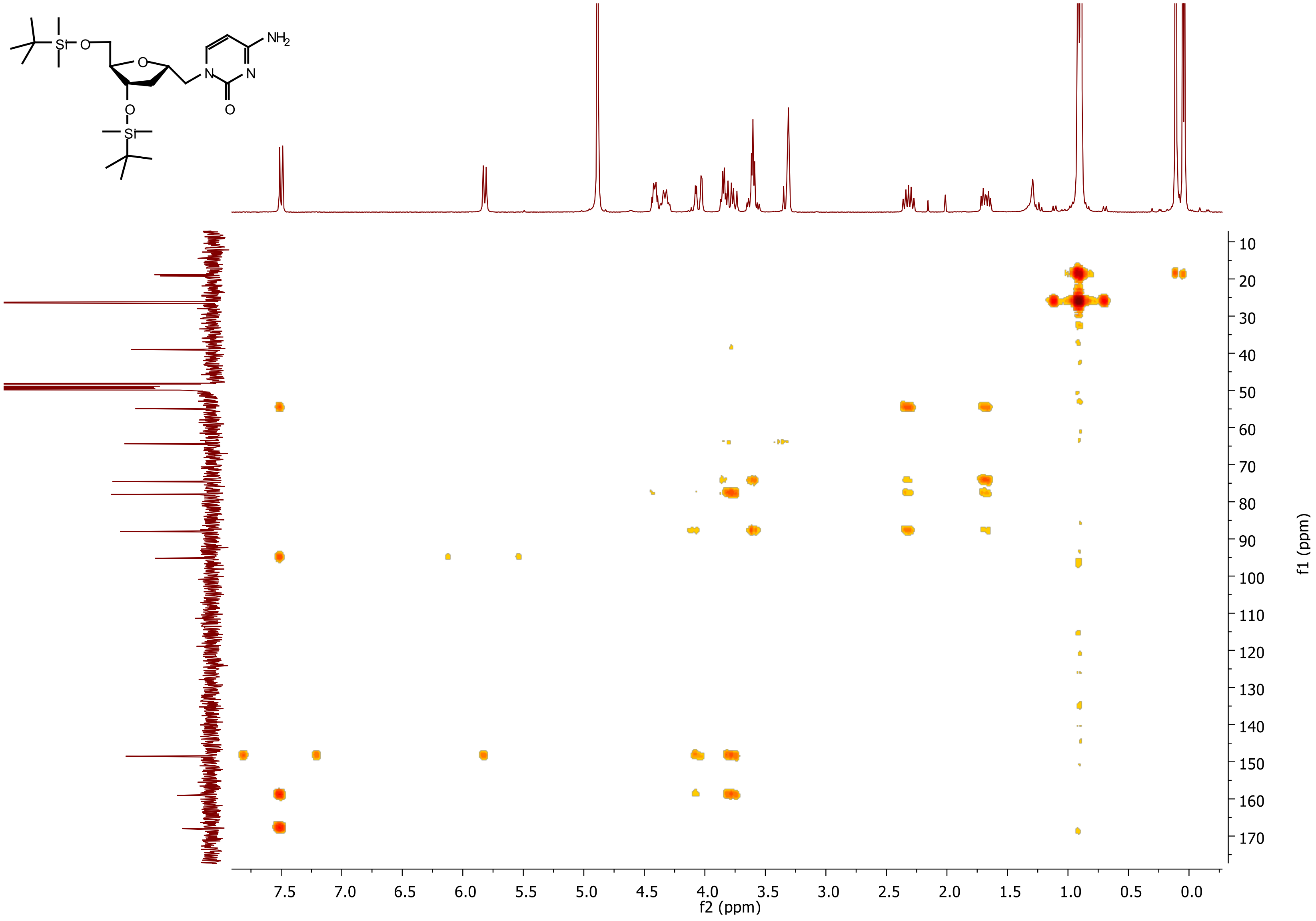

N-3 analogue of 3,5-bis-*O*-(*tert*-Butyldimethylsilyl)-1'-homo-*N*-2'-deoxy-α-cytidine (17e)

^{1}H NMR (300.13 MHz, MeOH-d_4)

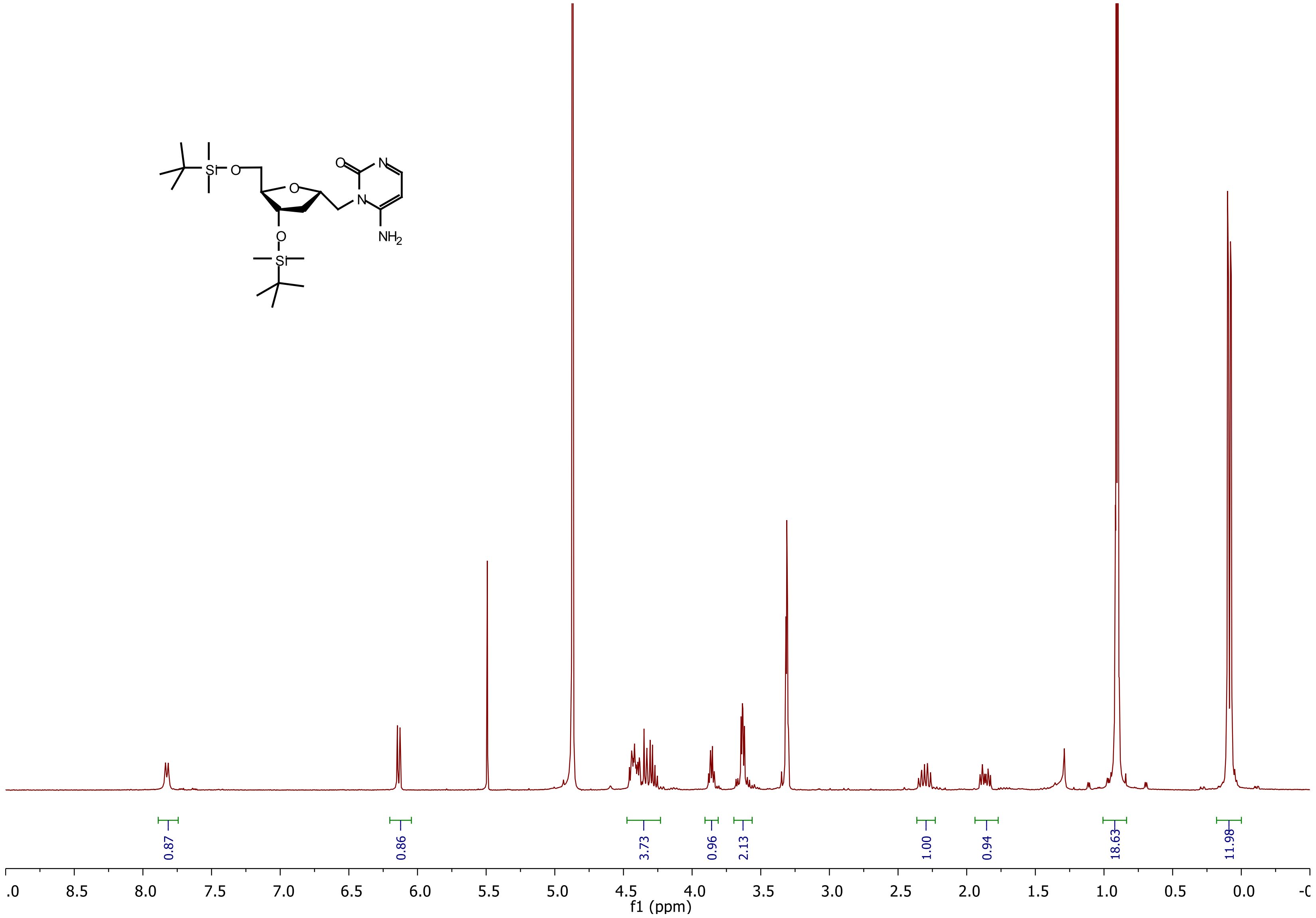

N-3 analogue of 3,5-bis-*O*-(*tert*-Butyldimethylsilyl)-1'-homo-*N*-2'-deoxy-α-cytidine (17e)

^{13}C NMR (75.5 MHz, MeOH-d_4)

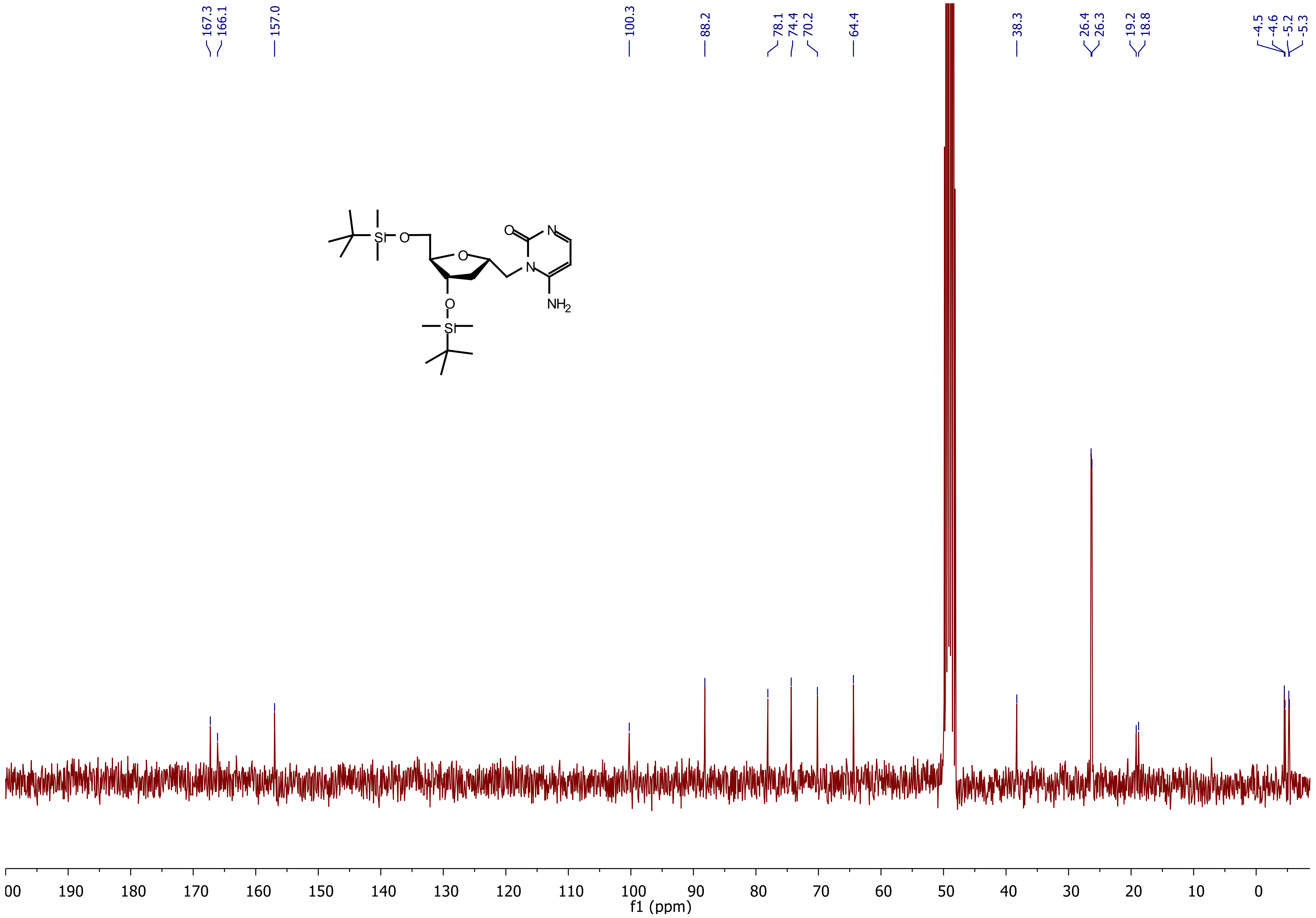

N-3 analogue of 3,5-bis-*O*-(*tert*-Butyldimethylsilyl)-1’-homo-*N*-2’-deoxy-α-cytidine (17e)

DEPT NMR (75.5 MHz, MeOH-d_4)

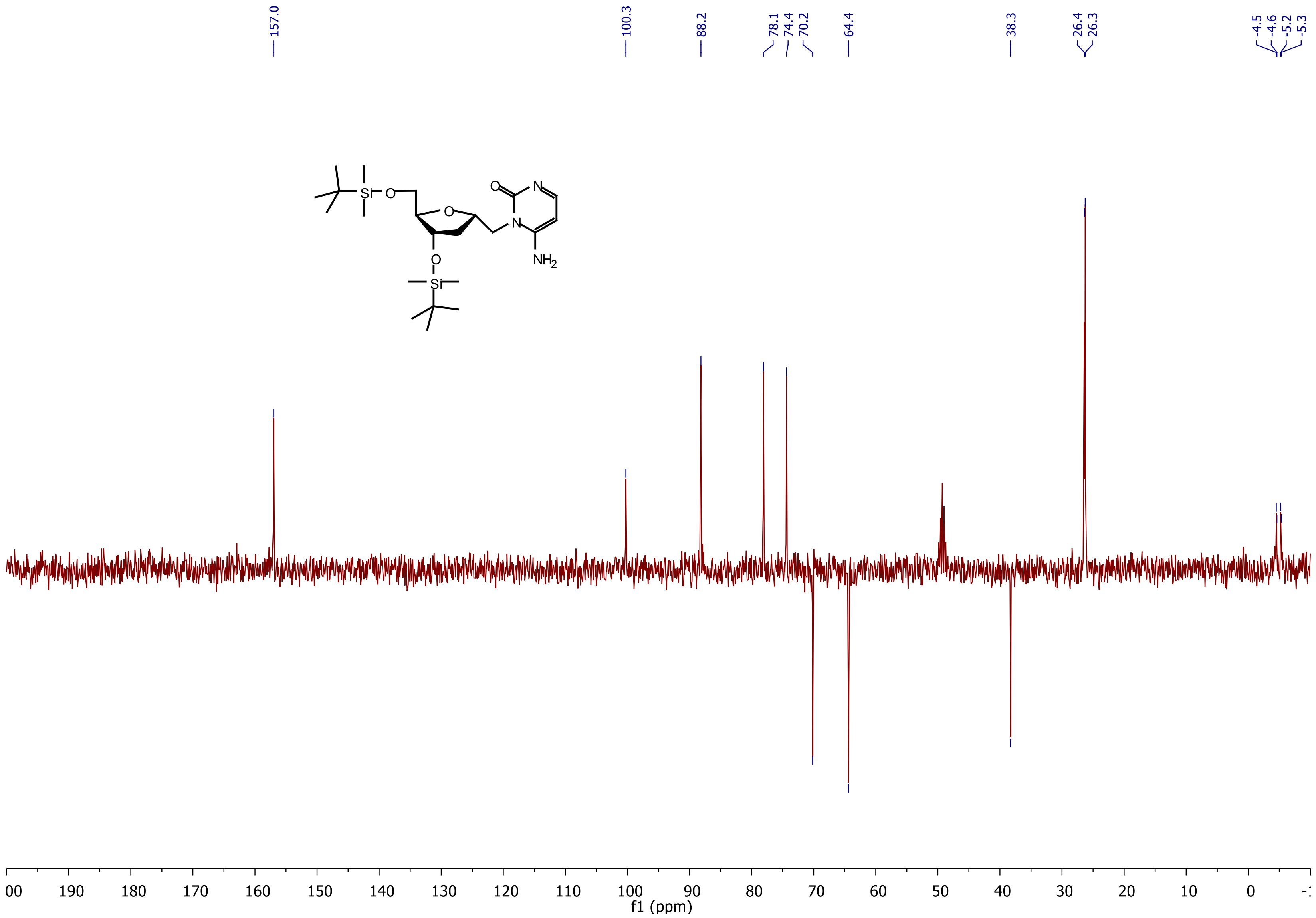

N-3 analogue of 3,5-bis-*O*-(*tert*-Butyldimethylsilyl)-1'-homo-*N*-2'-deoxy-α-cytidine (17e)

COSY NMR (MeOH-d_4)

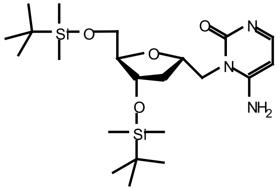

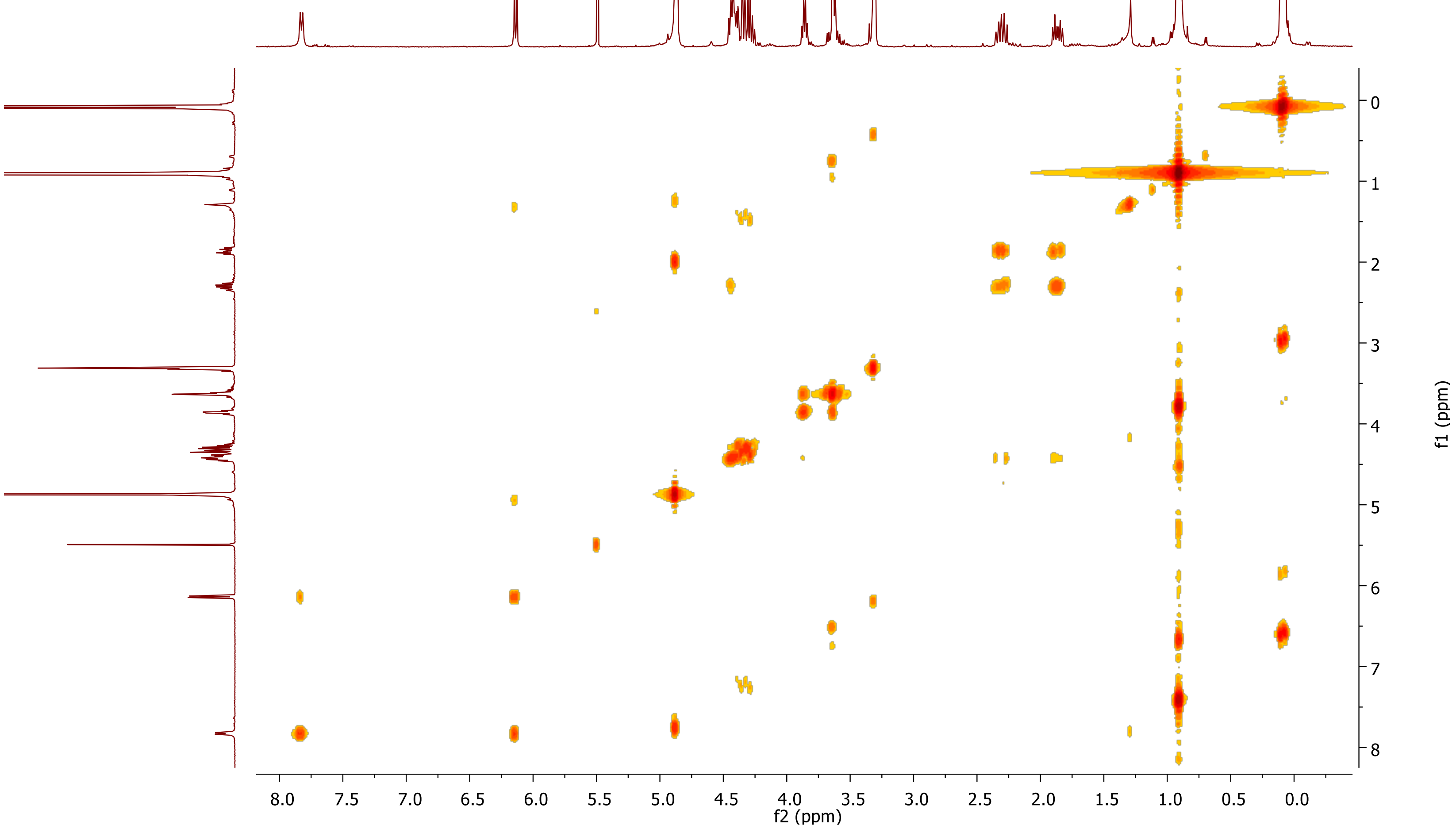

N-3 analogue of 3,5-bis-*O*-(*tert*-Butyldimethylsilyl)-1'-homo-*N*-2'-deoxy-α-cytidine (17e)

HSQC NMR (MeOH-d_4)

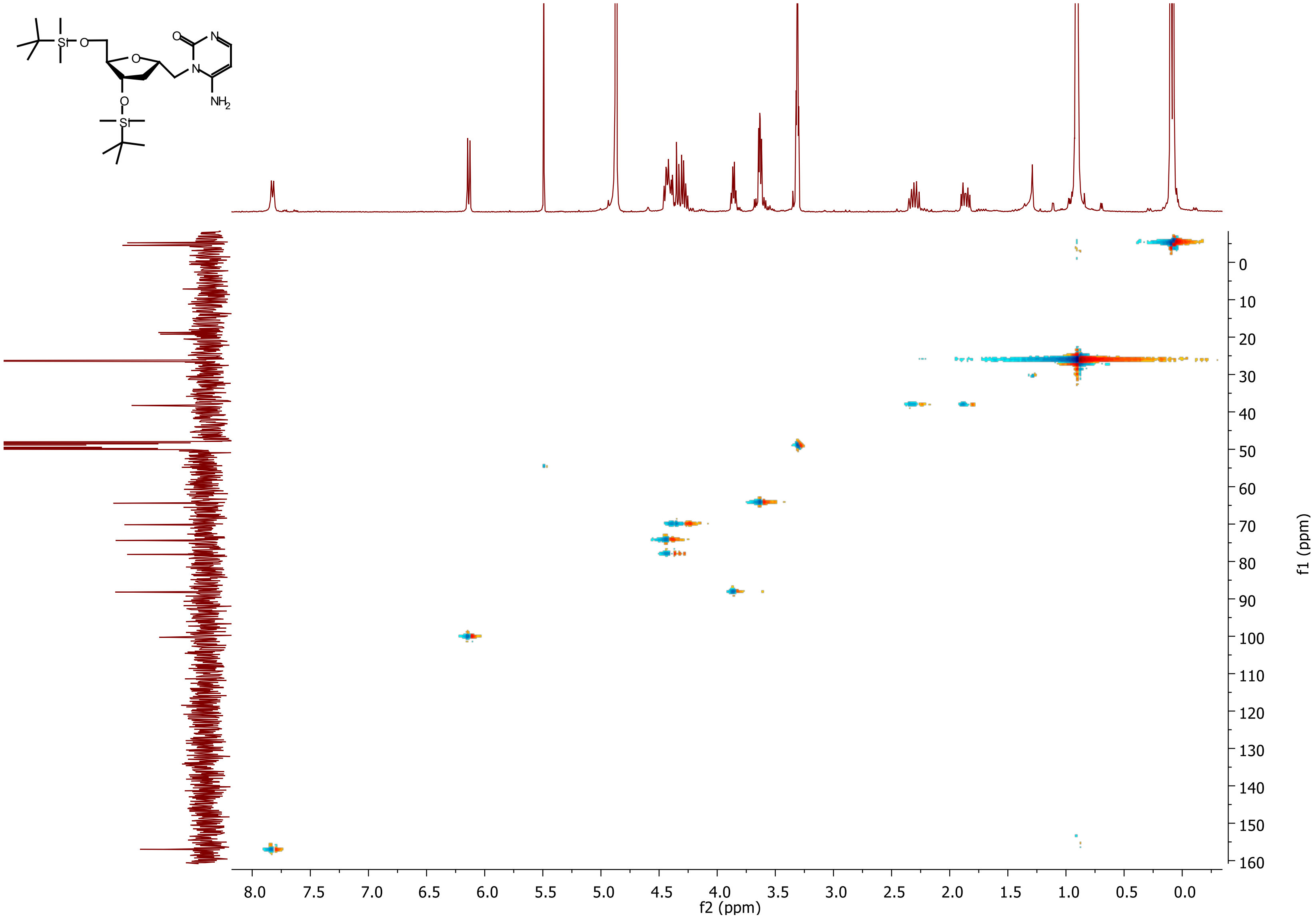

N-3 analogue of 3,5-bis-*O*-(*tert*-Butyldimethylsilyl)-1'-homo-*N*-2'-deoxy-α-cytidine (17e)

HMBC NMR (MeOH-d_4)

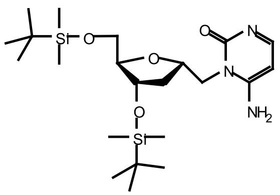

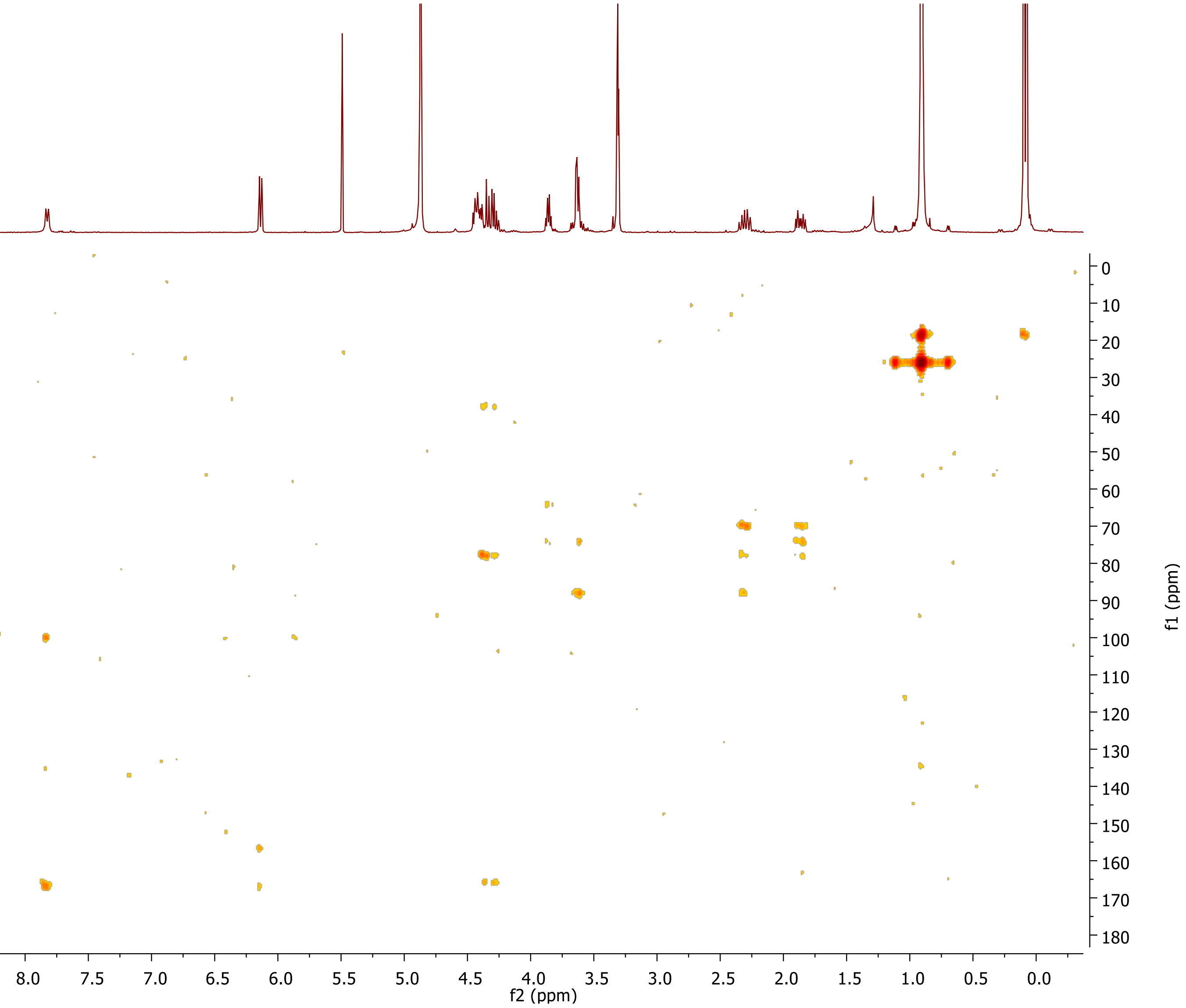

3,5-bis-*O*-(*tert*-Butyldimethylsilyl)-1’-homo-*N*-2’-deoxy-α-5-iodouridine (17f)

^{1}H NMR (300.13 MHz, MeOH-d_4)

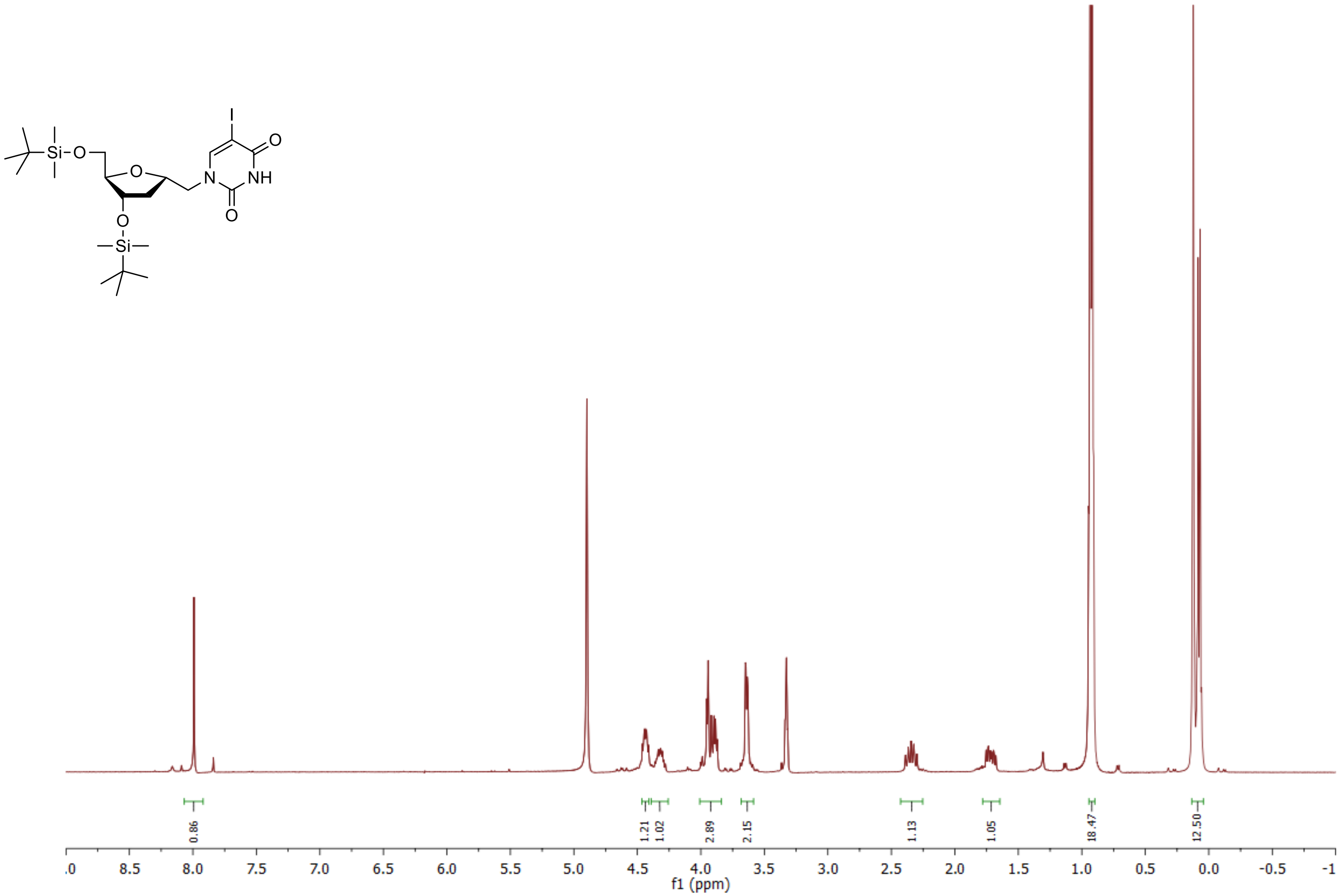

3,5-bis-*O*-(*tert*-Butyldimethylsilyl)-1'-homo-*N*-2'-deoxy-α-5-iodouridine (17f)

^{13}C NMR (75.5 MHz, MeOH-d_4)

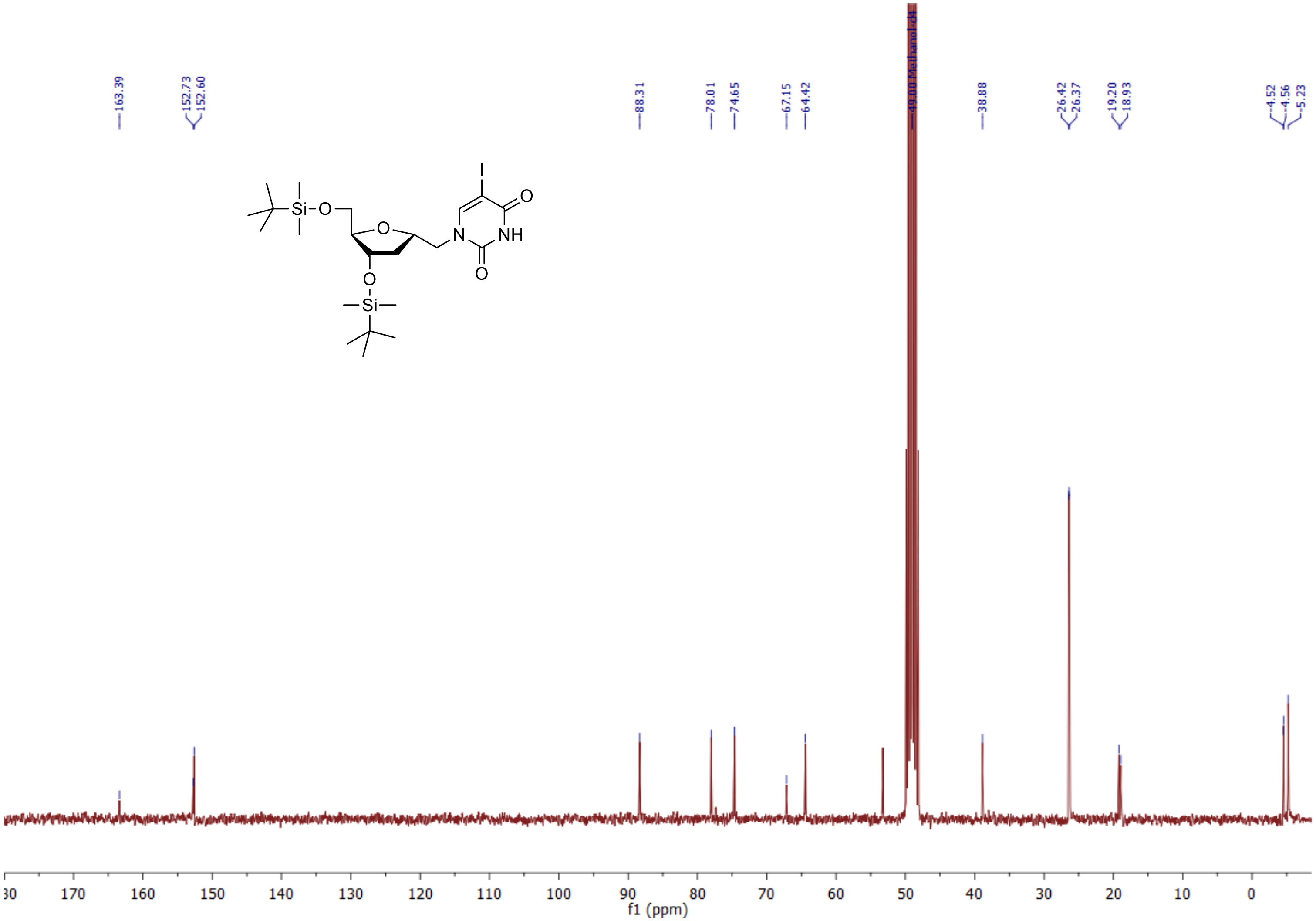

3,5-bis-*O*-(*tert*-Butyldimethylsilyl)-1'-homo-*N*-2'-deoxy-α-5-iodouridine (17f)

DEPT 135 NMR (75.5 MHz, MeOH-d_4)

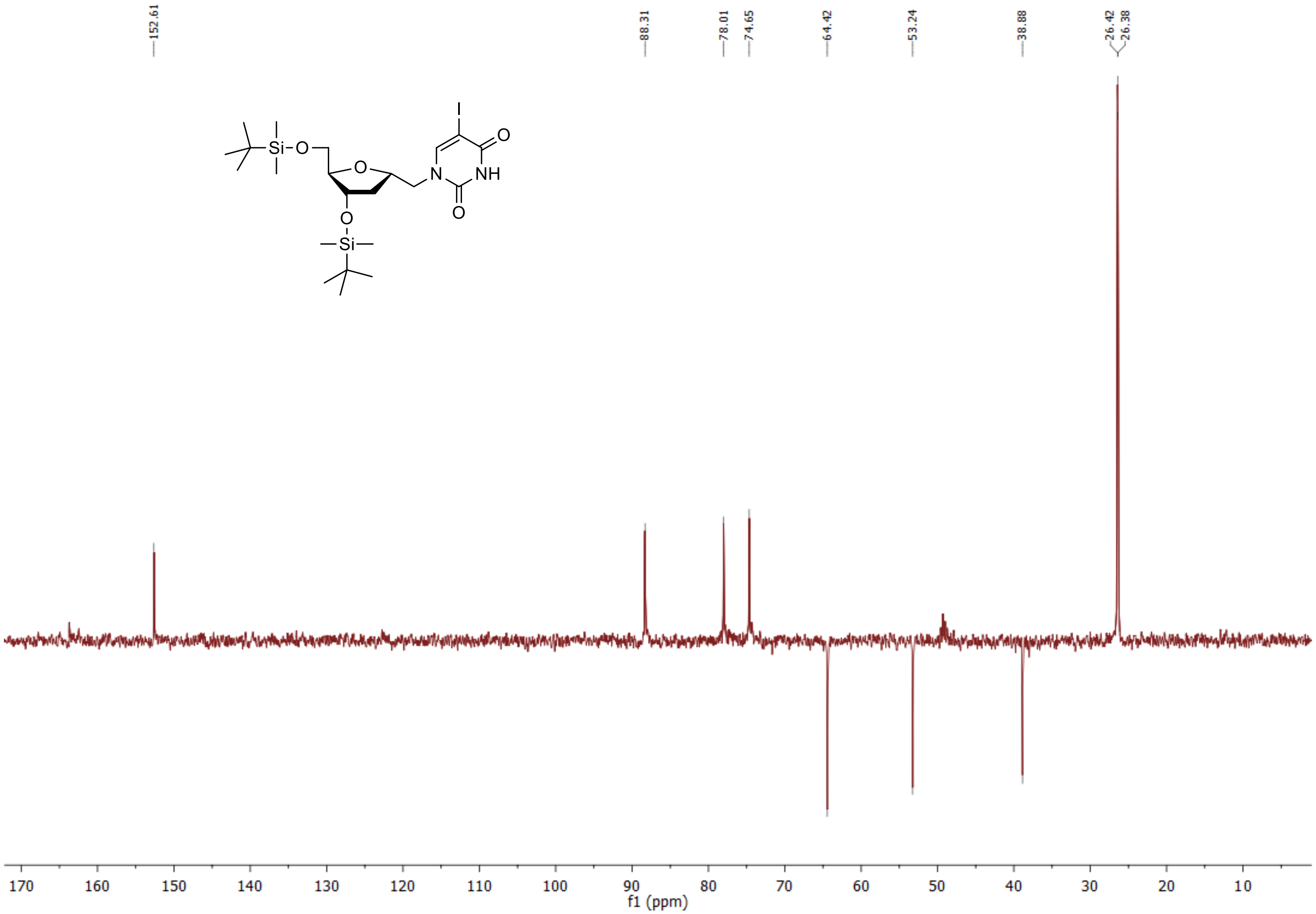

3,5-bis-*O*-(*tert*-Butyldimethylsilyl)-1'-homo-*N*-2'-deoxy-α-5-iodouridine (17f)

COSY NMR (MeOH-d_4)

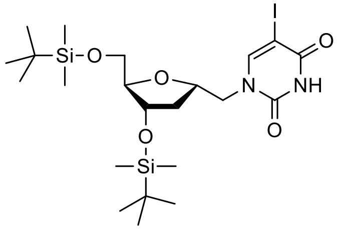

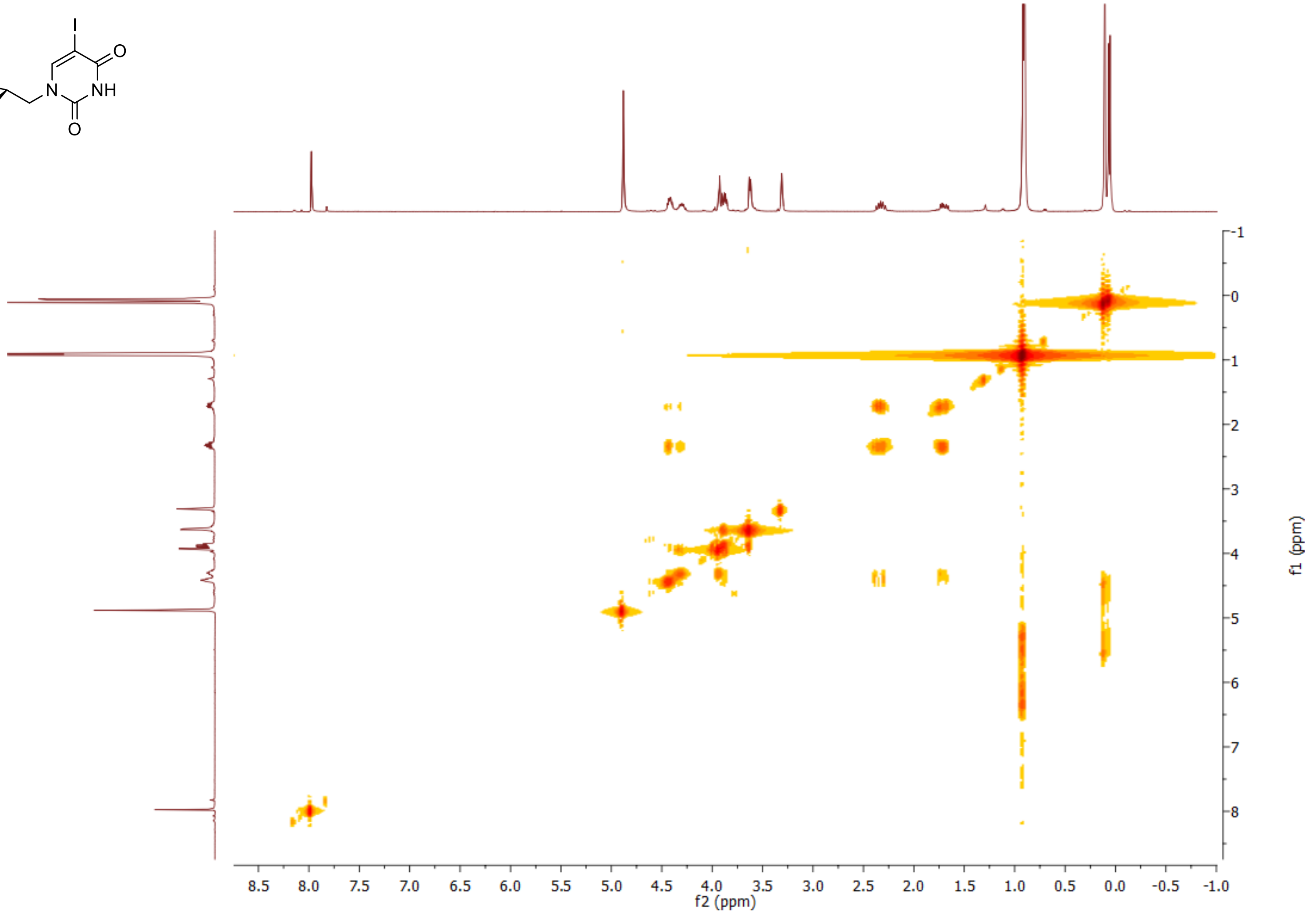

3,5-bis-*O*-(*tert*-Butyldimethylsilyl)-1’-homo-*N*-2’-deoxy-α-5-iodouridine (17f)

HSQC NMR (MeOH-d_4)

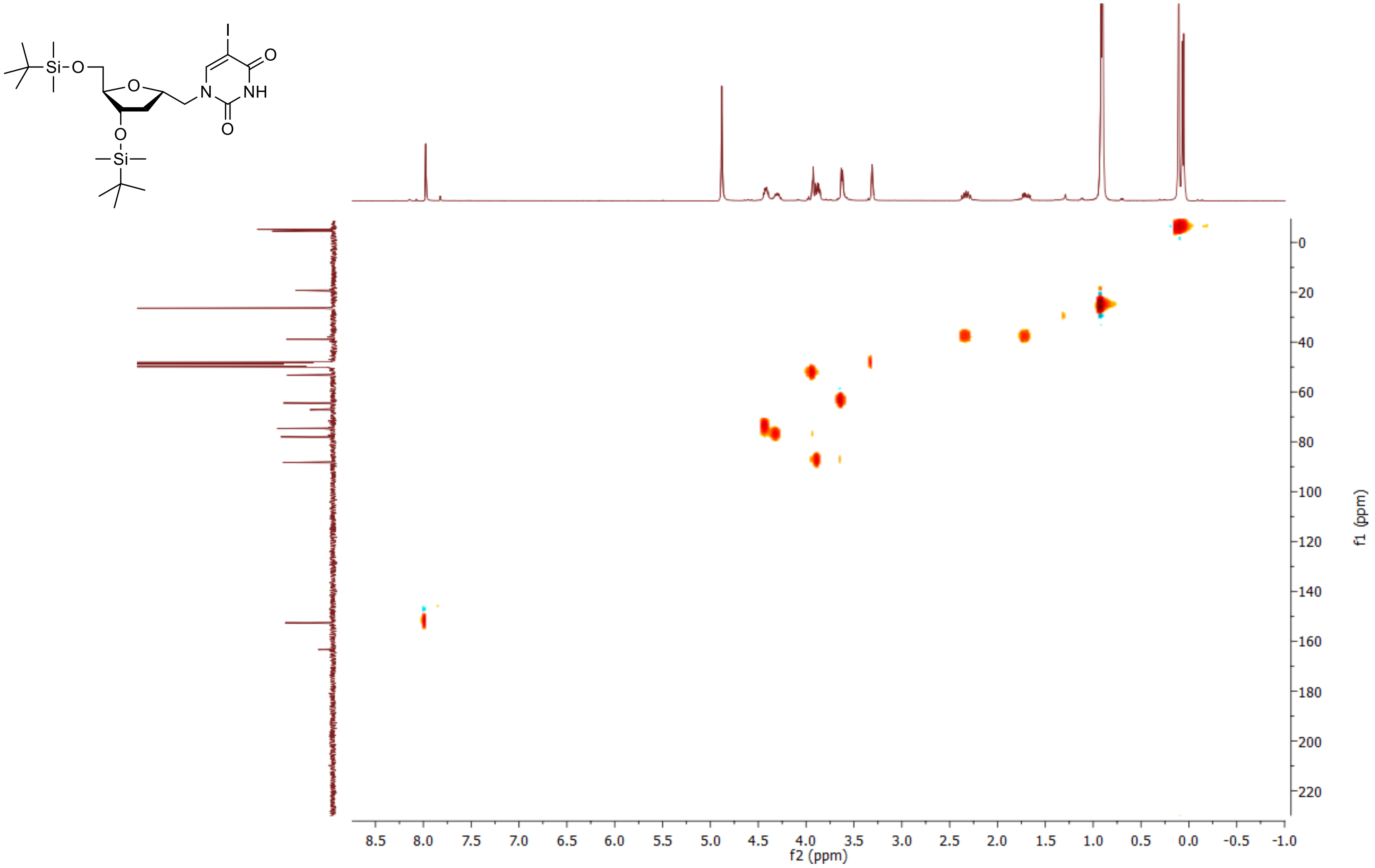

3,5-bis-*O*-(*tert*-Butyldimethylsilyl)-1'-homo-*N*-2'-deoxy-α-5-iodouridine (17f)

HMBC NMR (MeOH-d_4)

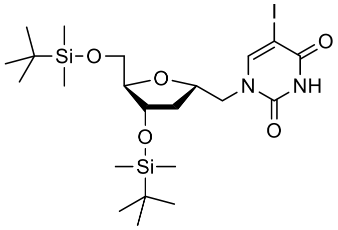

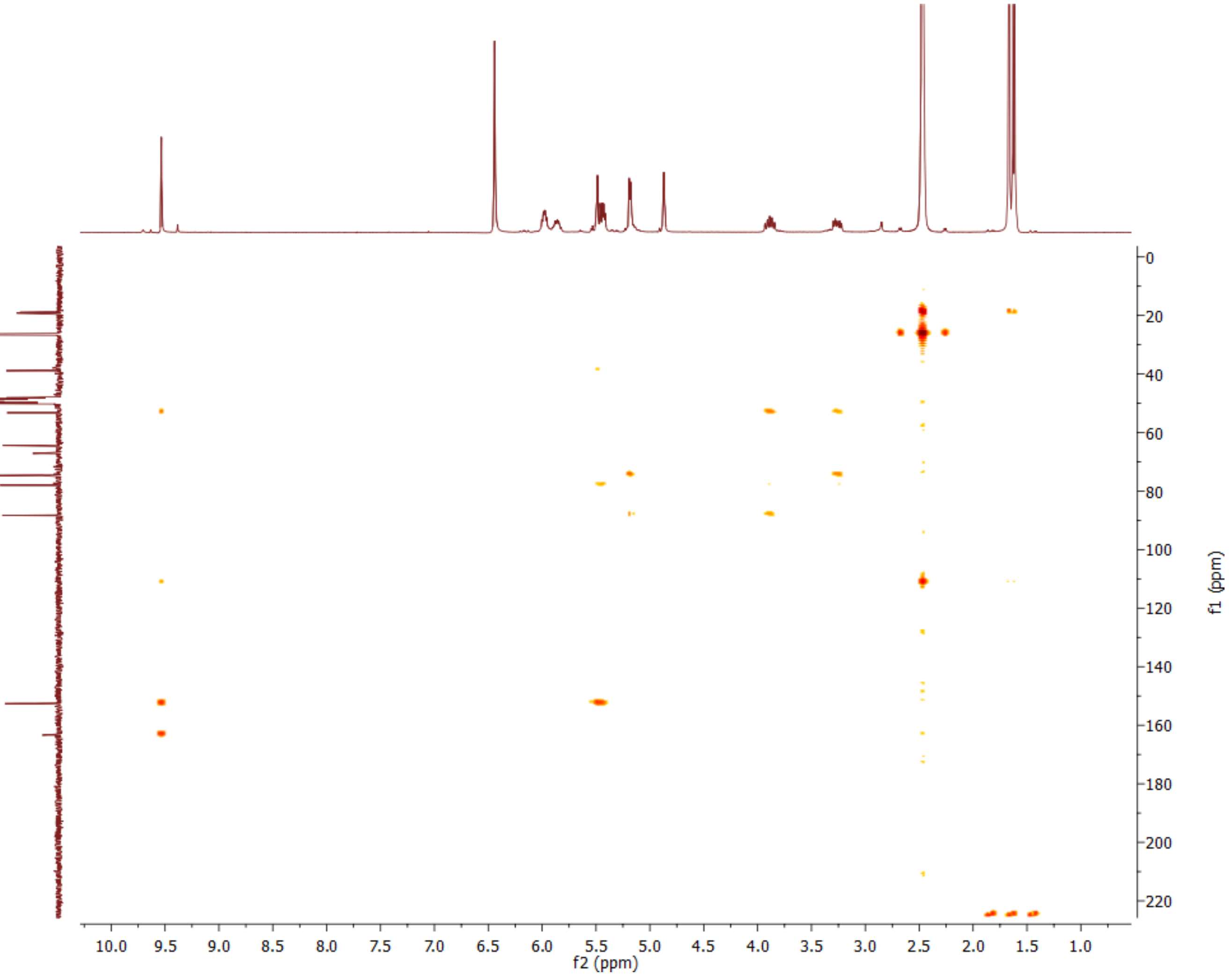

N^1,N^3-bis-[3,5-bis-*O*-(*tert*-Butyldimethylsilyl)-1,2-dideoxy-α-ribofuranosylmethyl]uracil (18b)

^{1}H NMR (300.13 MHz, MeOH-d_4)

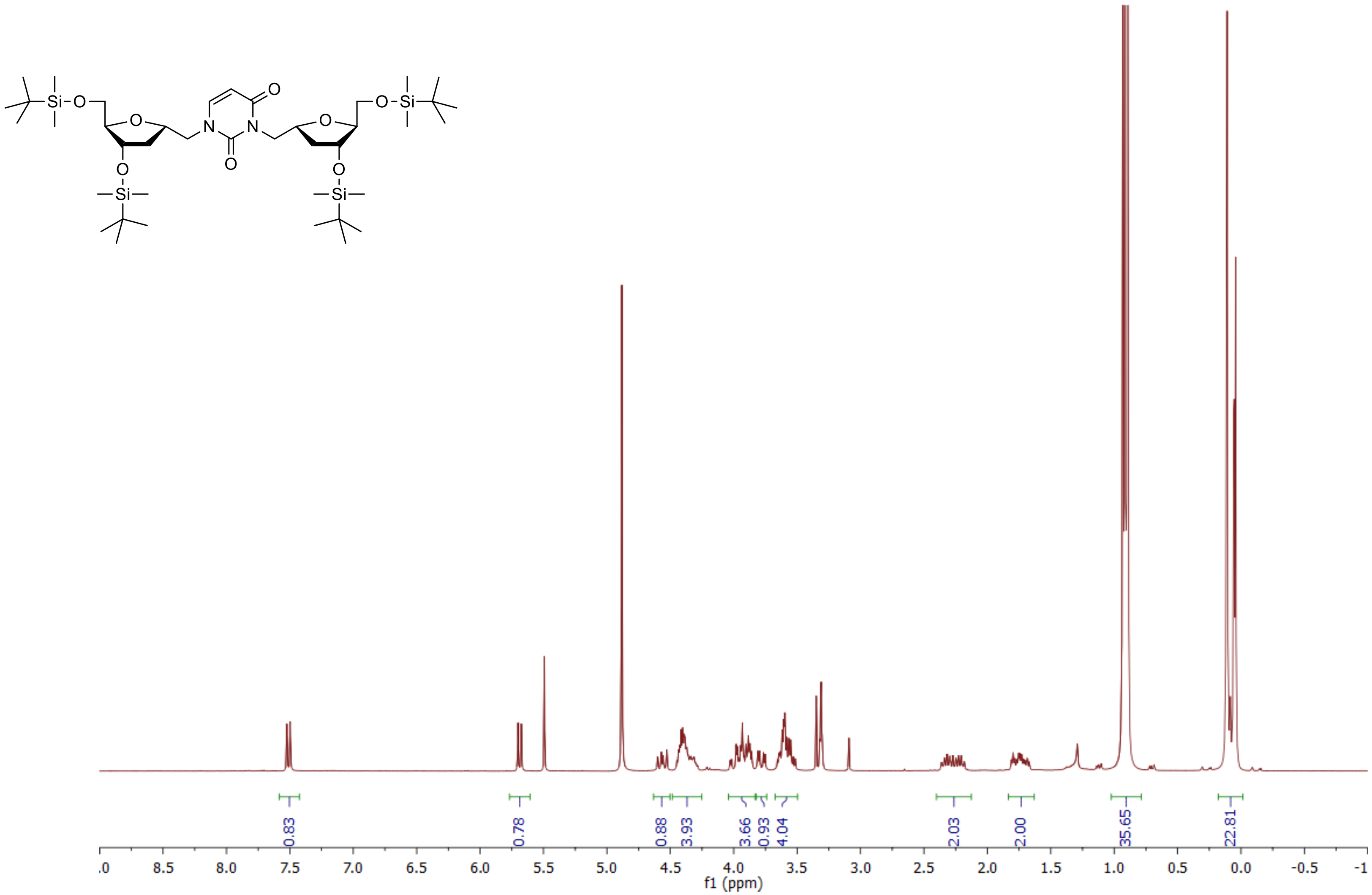

N^1,N^3-bis-[3,5-bis-*O*-(*tert*-Butyldimethylsilyl)-1,2-dideoxy-α-ribofuranosylmethyl]uracil (18b)

^{13}C NMR (75.5 MHz, MeOH-d_4)

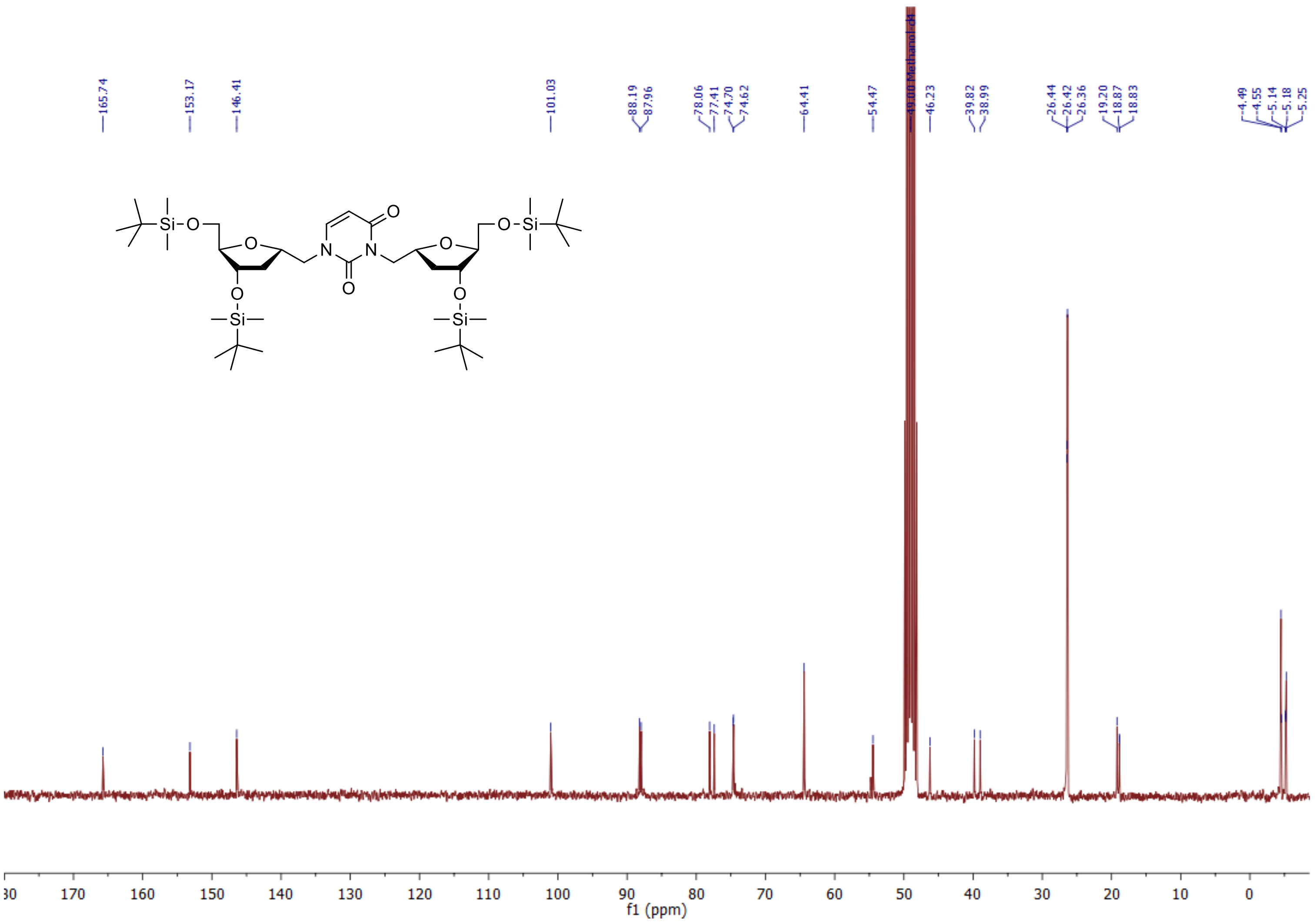

N^1,N^3-bis-[3,5-bis-*O*-(*tert*-Butyldimethylsilyl)-1,2-dideoxy-α-ribofuranosylmethyl]uracil (18b)

DEPT 135 NMR (75.5 MHz, MeOH-d_4)

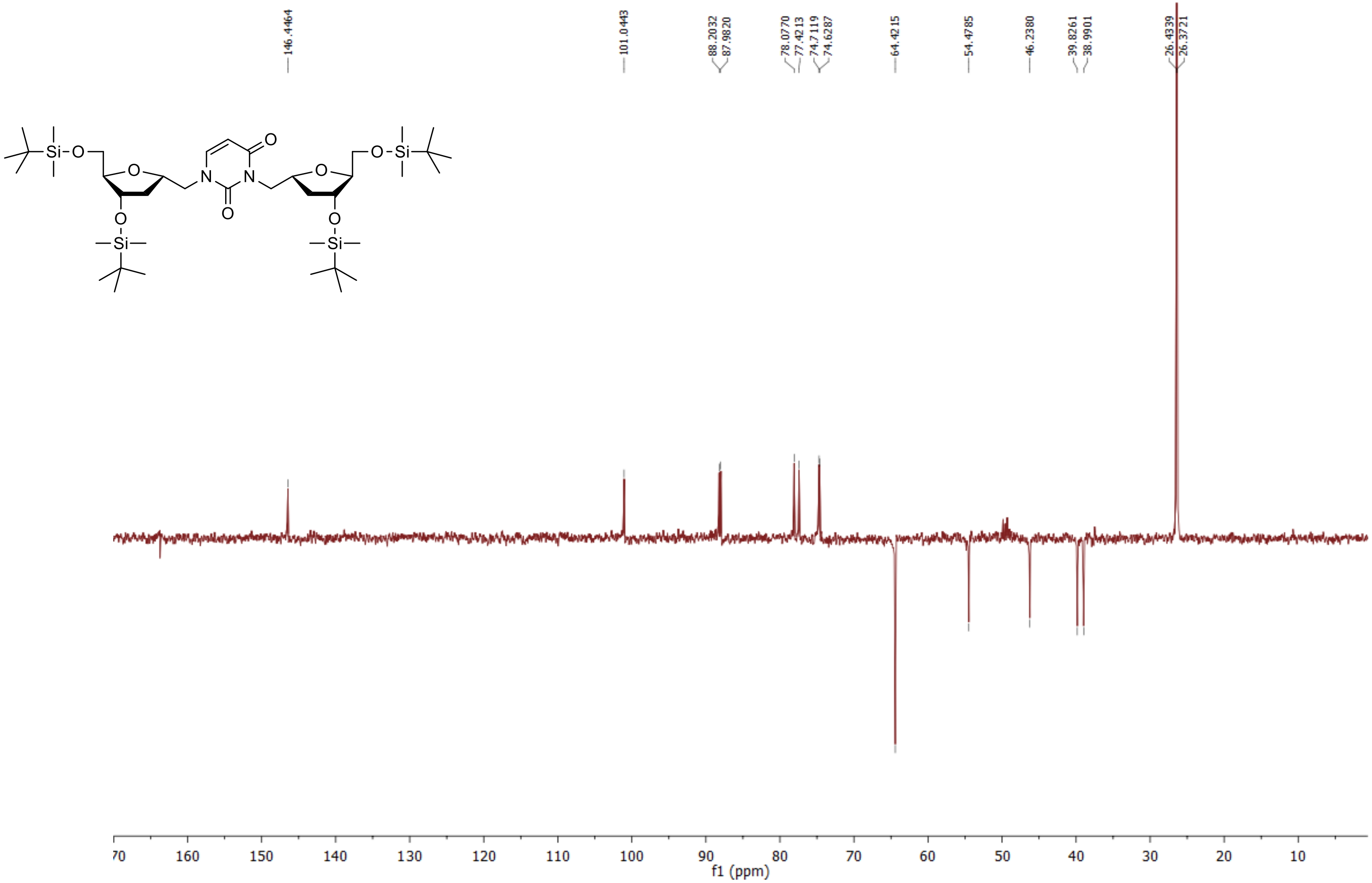

N^1,N^3-bis-[3,5-bis-*O*-(*tert*-Butyldimethylsilyl)-1,2-dideoxy-α-ribofuranosylmethyl]uracil (18b)

COSY NMR (MeOH-d_4)

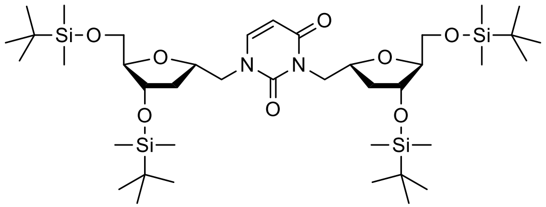

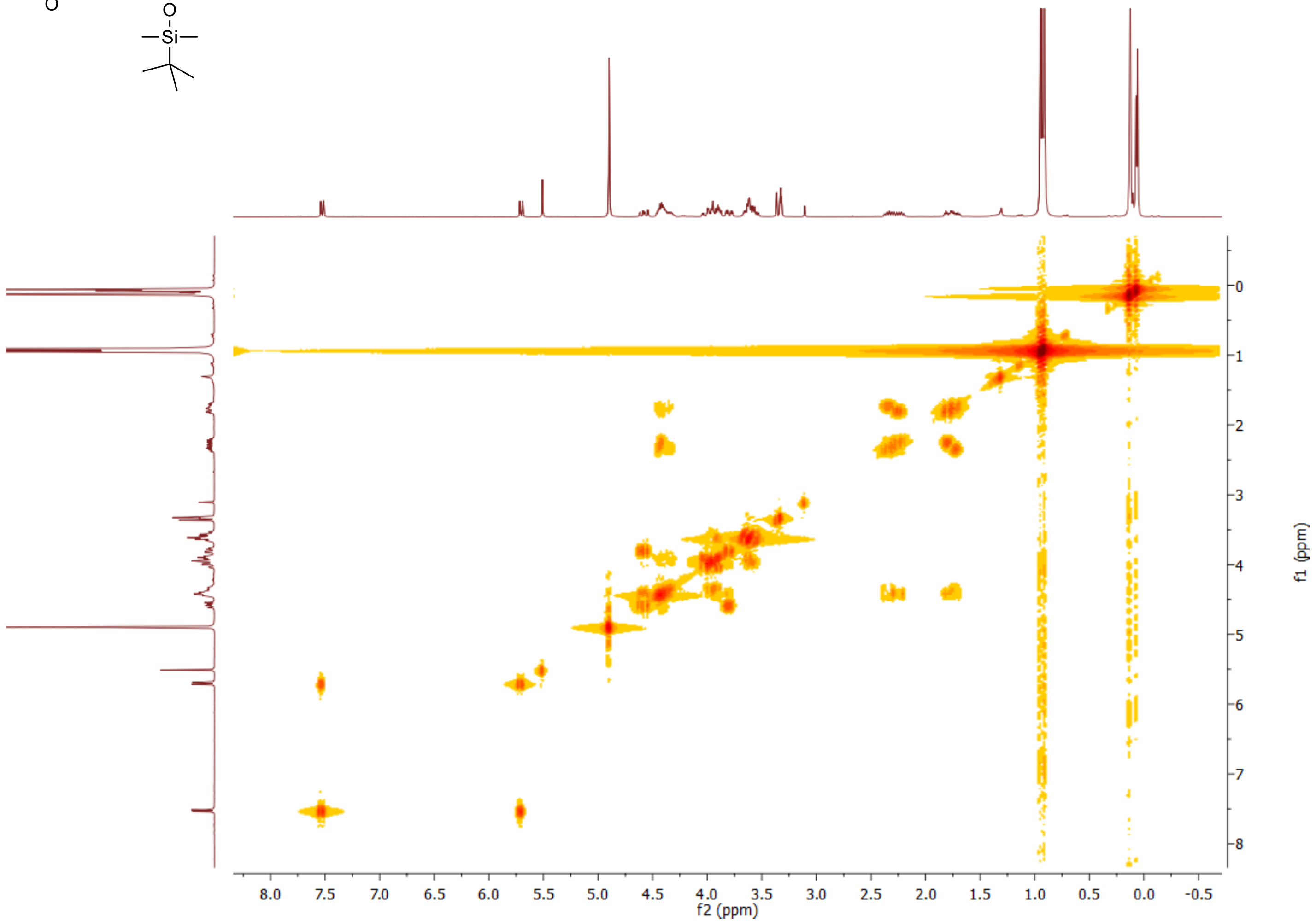

N^1,N^3-bis-[3,5-bis-*O*-(*tert*-Butyldimethylsilyl)-1,2-dideoxy-α-ribofuranosylmethyl]uracil (18b)

HSQC NMR (MeOH-d_4)

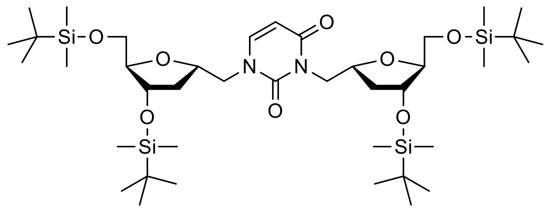

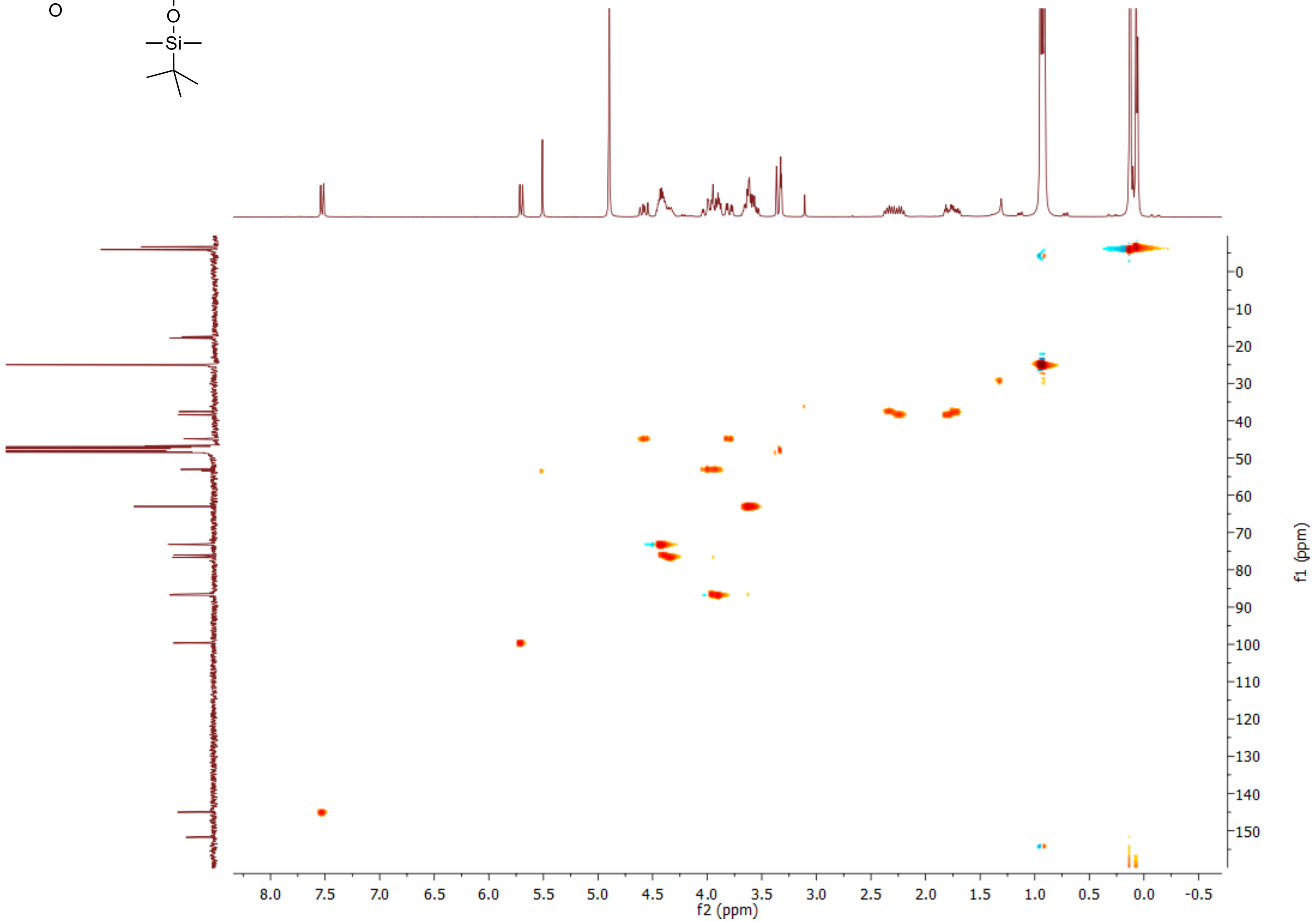

N^1,N^3-bis-[3,5-bis-*O*-(*tert*-Butyldimethylsilyl)-1,2-dideoxy-α-ribofuranosylmethyl]uracil (18b)

HMBC NMR (MeOH-d_4)

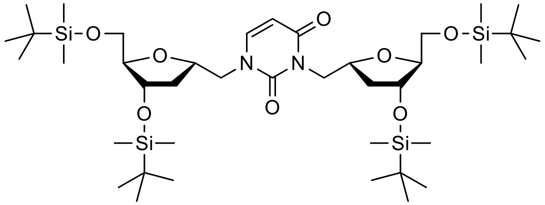

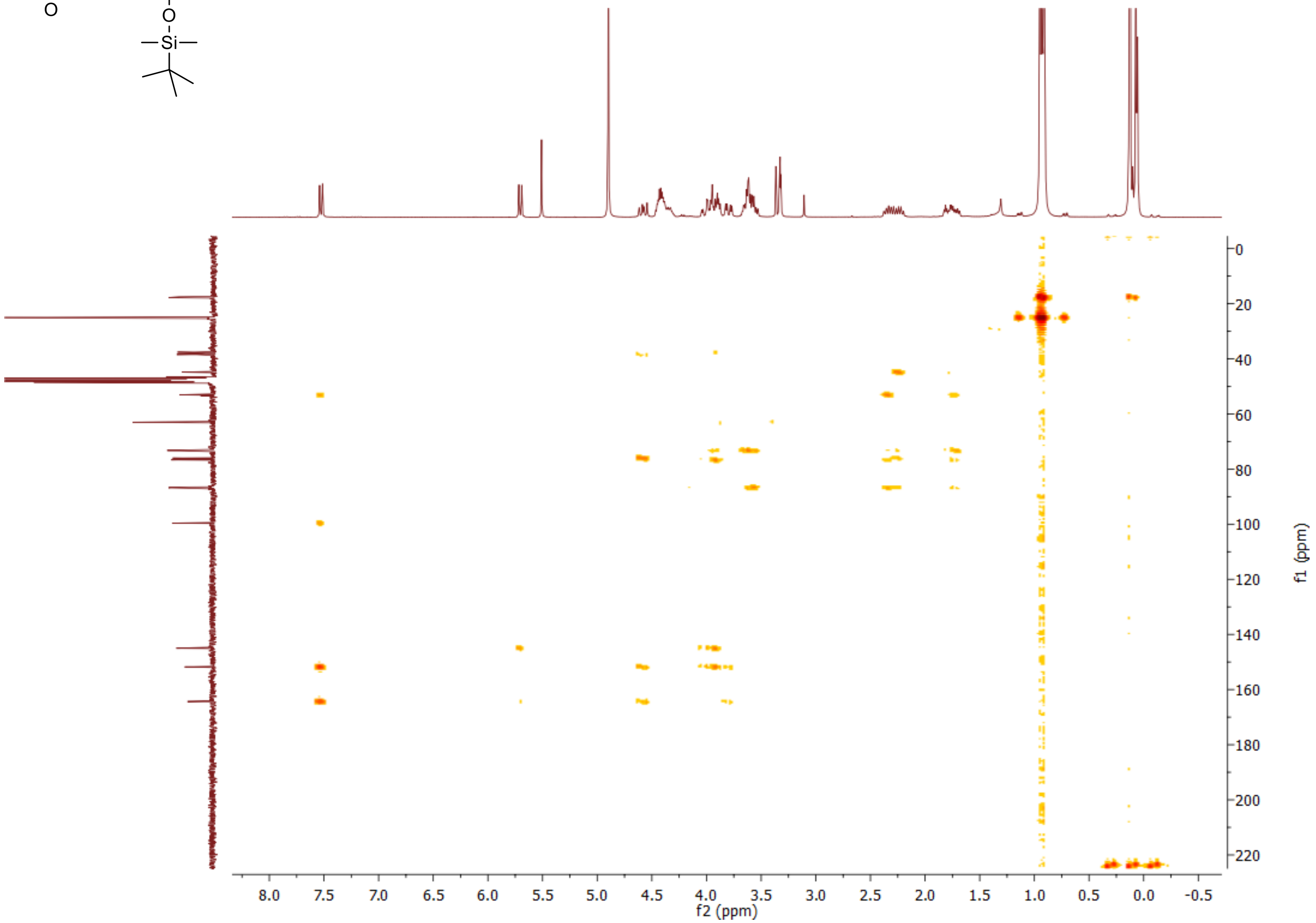

N^1,N^3-bis-[3,5-bis-O-(*tert*-Butyldimethylsilyl)-1,2-dideoxy-α-ribofuranosylmethyl]thymine (18c)

^{1}H NMR (300.13 MHz, MeOH-d_4)

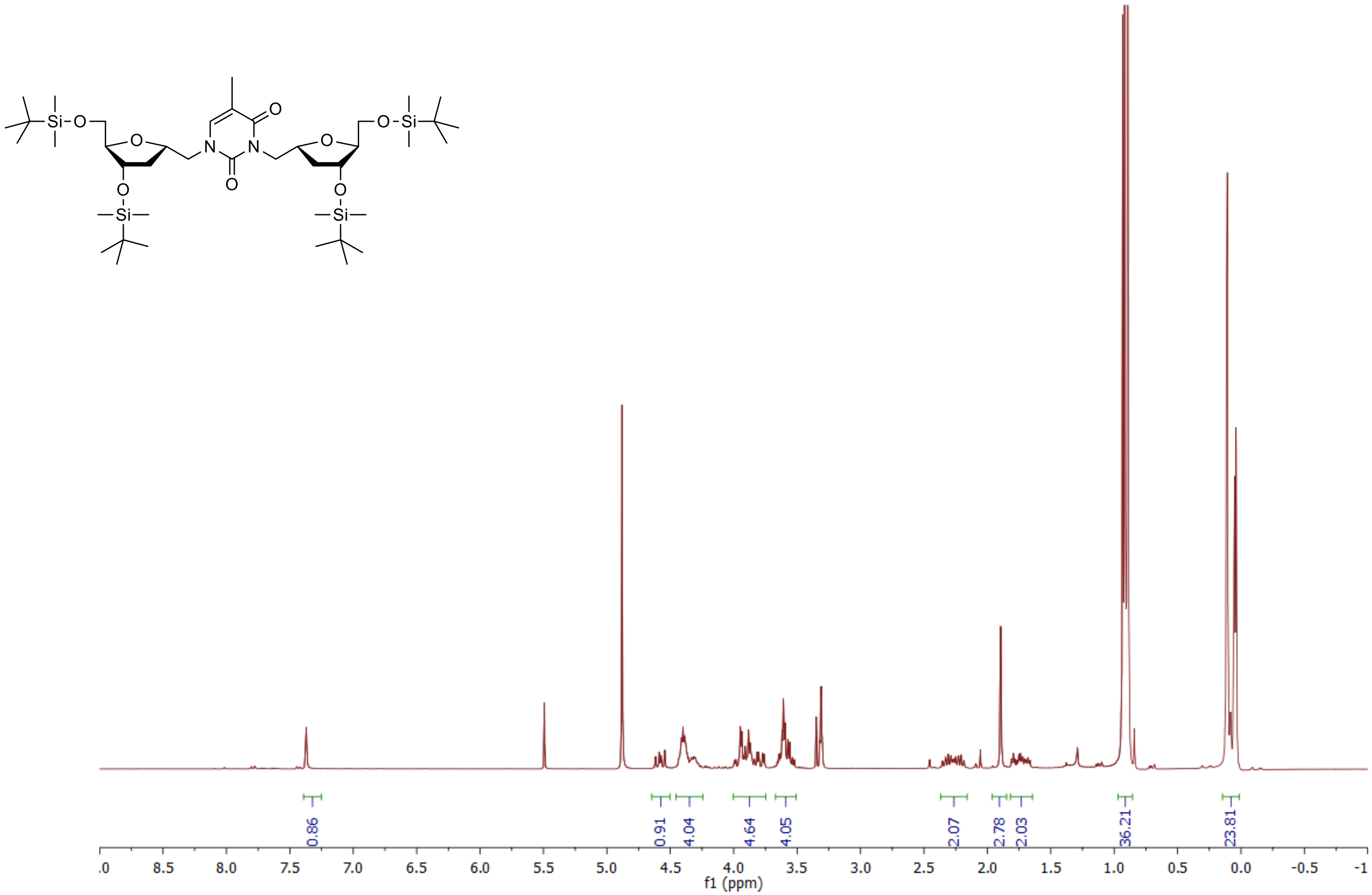

N^1,N^3-bis-[3,5-bis-*O*-(*tert*-Butyldimethylsilyl)-1,2-dideoxy-α-ribofuranosylmethyl]thymine (18c)

^{13}C NMR (75.5 MHz, MeOH-d_4)

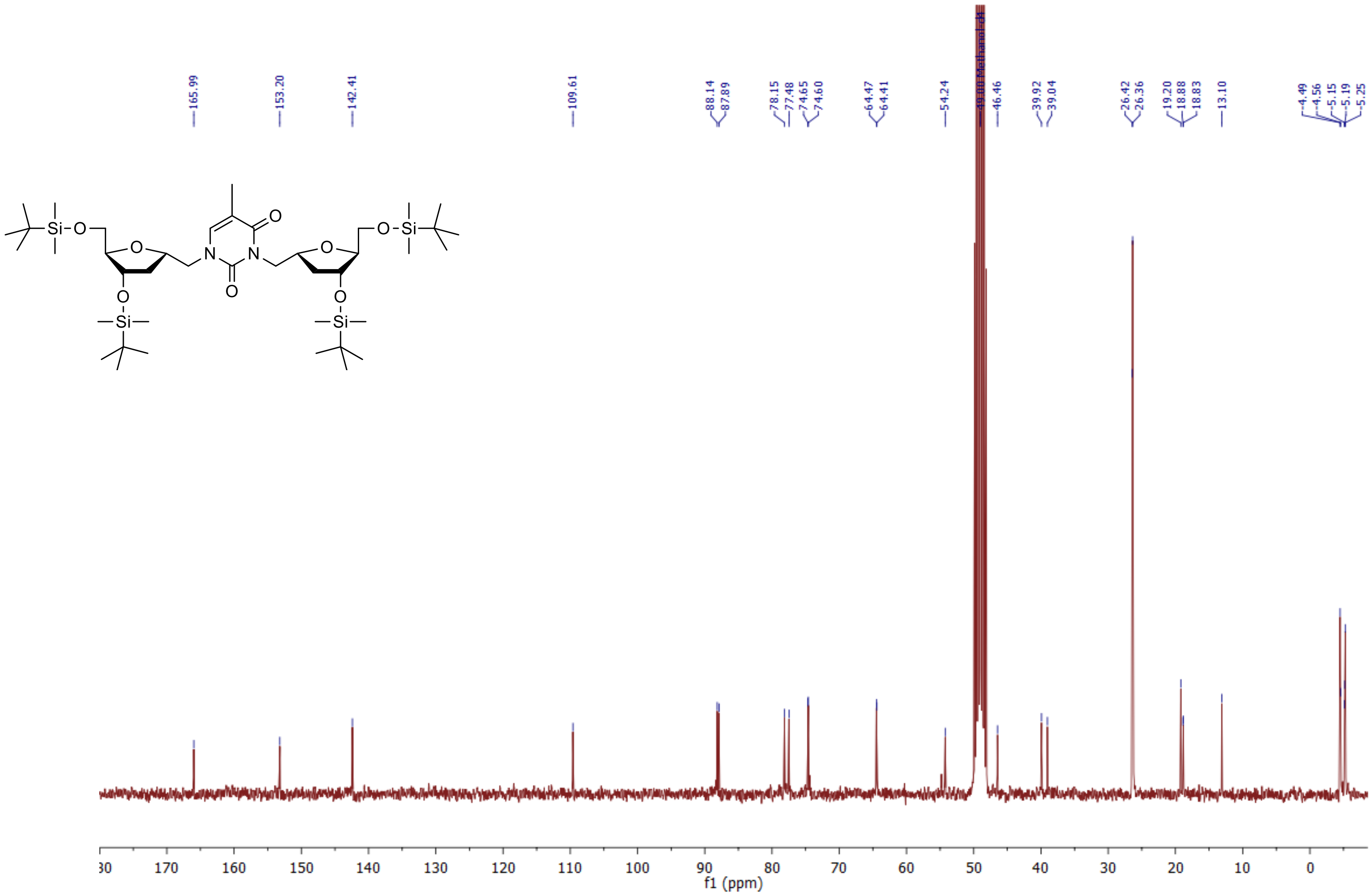

N^1,N^3-bis-[3,5-bis-*O*-(*tert*-Butyldimethylsilyl)-1,2-dideoxy-α-ribofuranosylmethyl]thymine (18c)

DEPT 135 NMR (75.5 MHz, MeOH-d_4)

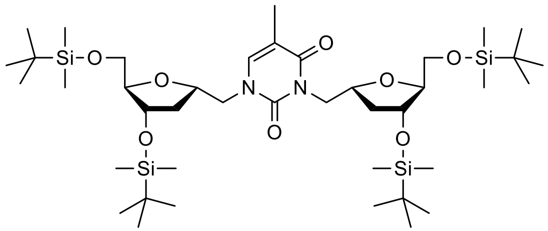

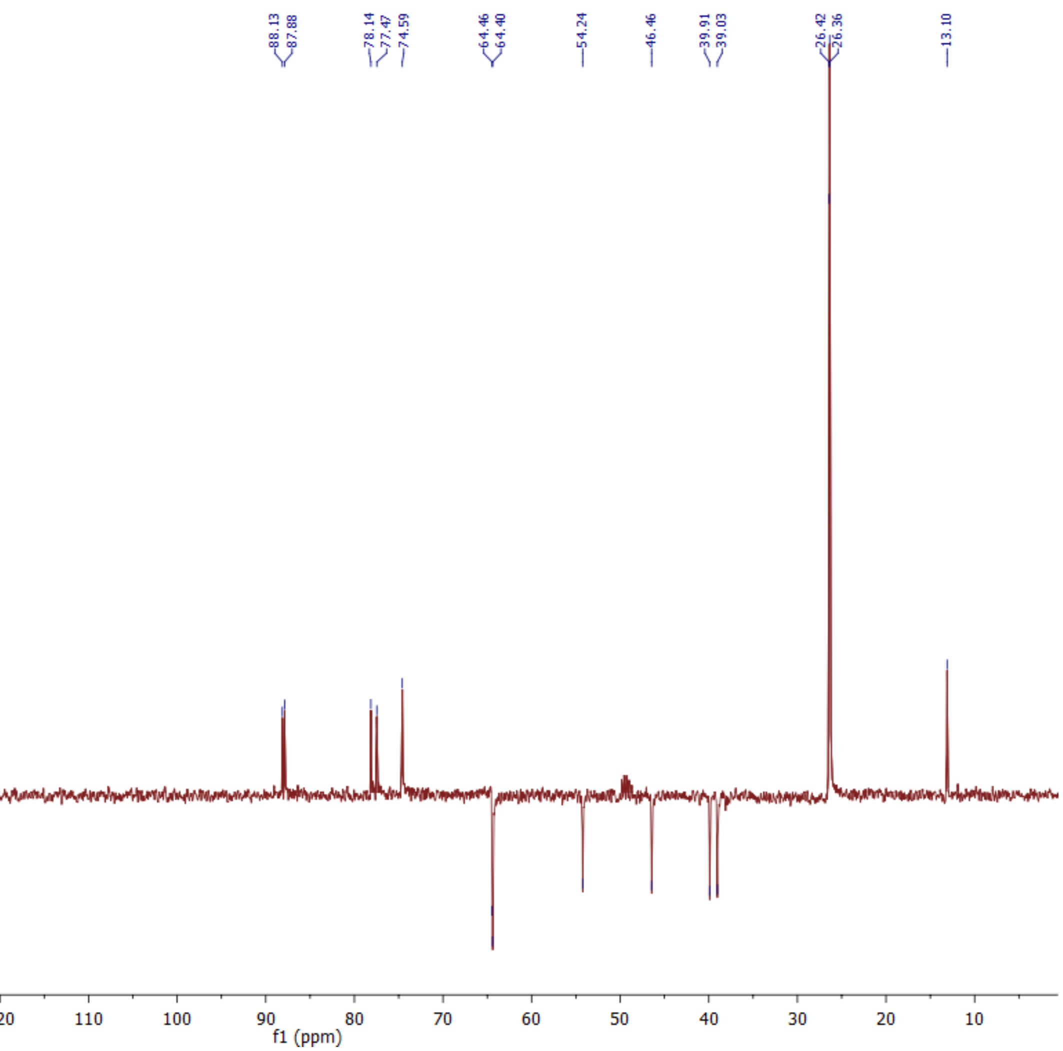

N^1,N^3-bis-[3,5-bis-*O*-(*tert*-Butyldimethylsilyl)-1,2-dideoxy-α-ribofuranosylmethyl]thymine (18c)

COSY NMR (MeOH-d_4)

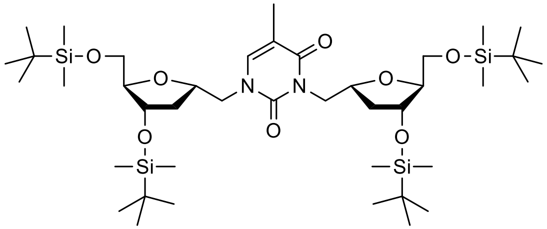

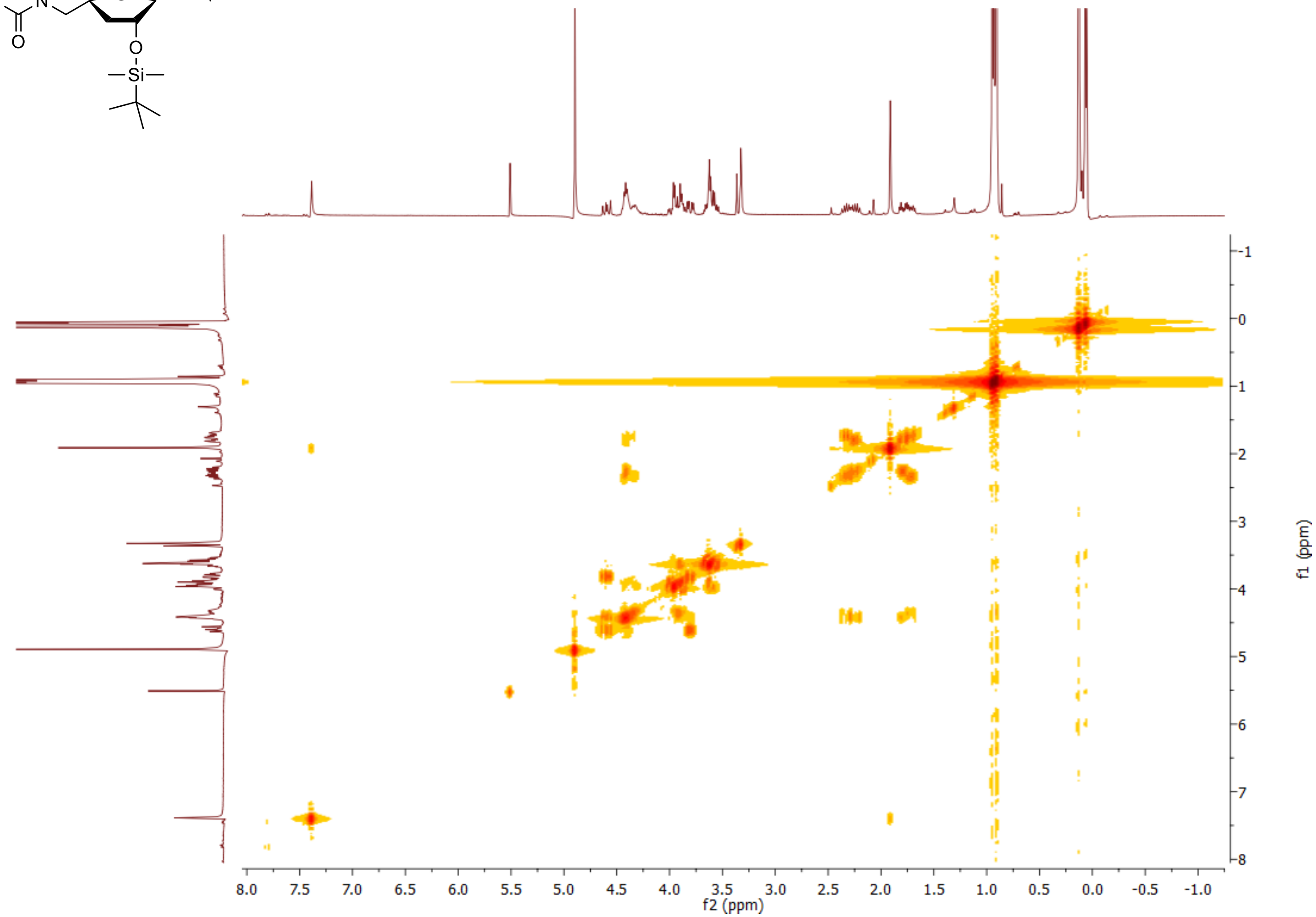

N^1,N^3-bis-[3,5-bis-*O*-(*tert*-Butyldimethylsilyl)-1,2-dideoxy-α-ribofuranosylmethyl]thymine (18c)

HSQC (75.5 MHz, MeOH-d_4)

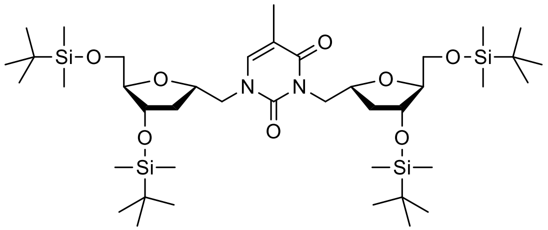

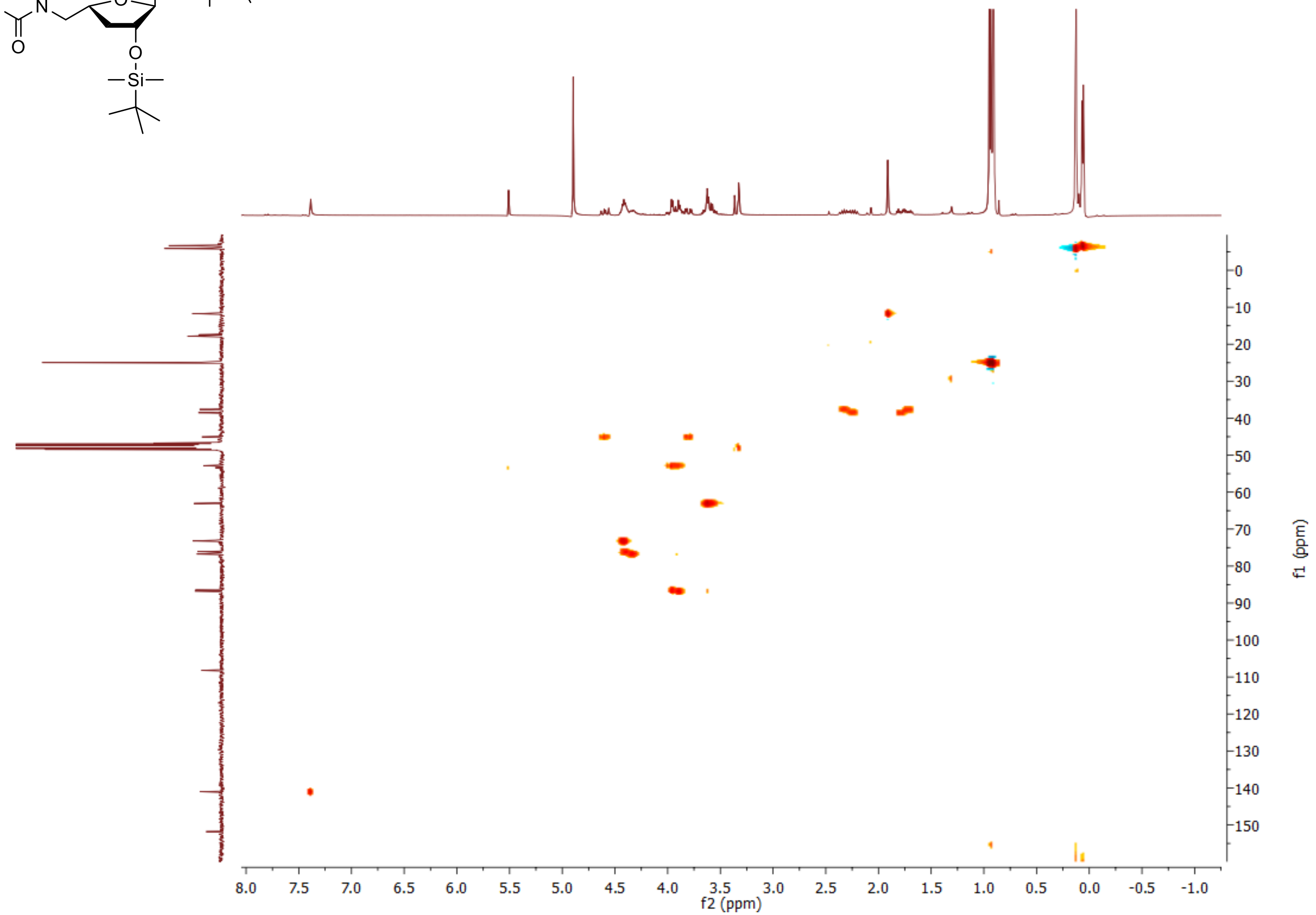

N^1,N^3-bis-[3,5-bis-*O*-(*tert*-Butyldimethylsilyl)-1,2-dideoxy-α-ribofuranosylmethyl]thymine (18c)

HMBC (MeOH-d_4)

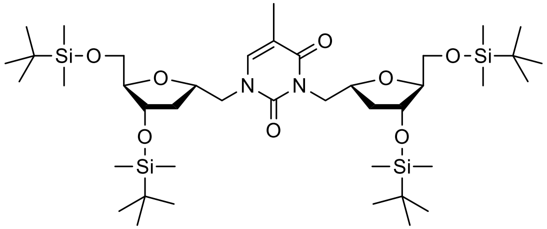

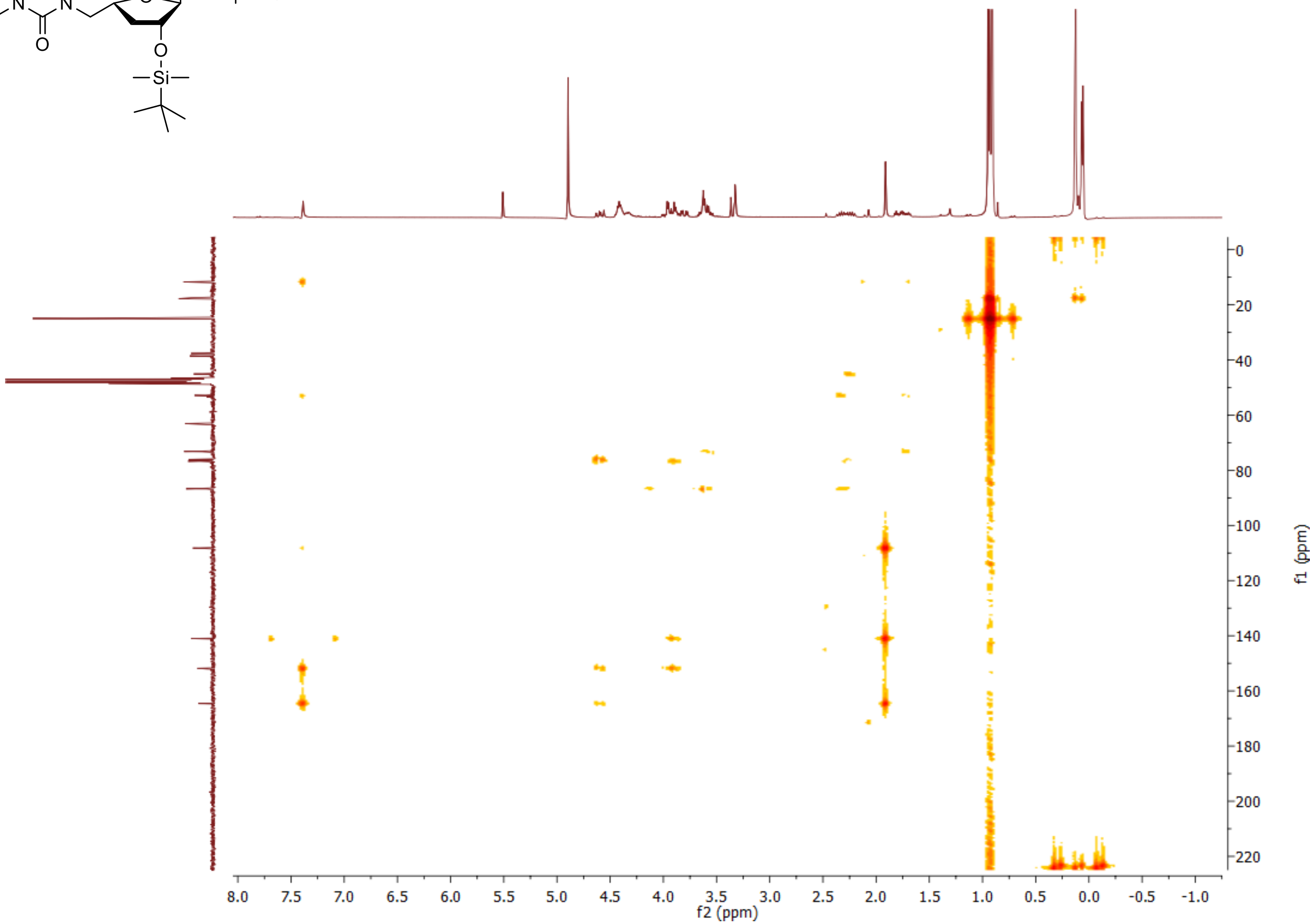

N^1,N^3-bis-[3,5-bis-*O*-(*tert*-Butyldimethylsilyl)-1,2-dideoxy-α-ribofuranosylmethyl]-5-fluorouracil (18d)

^{1}H NMR (300.13 MHz, MeOH-d_4)

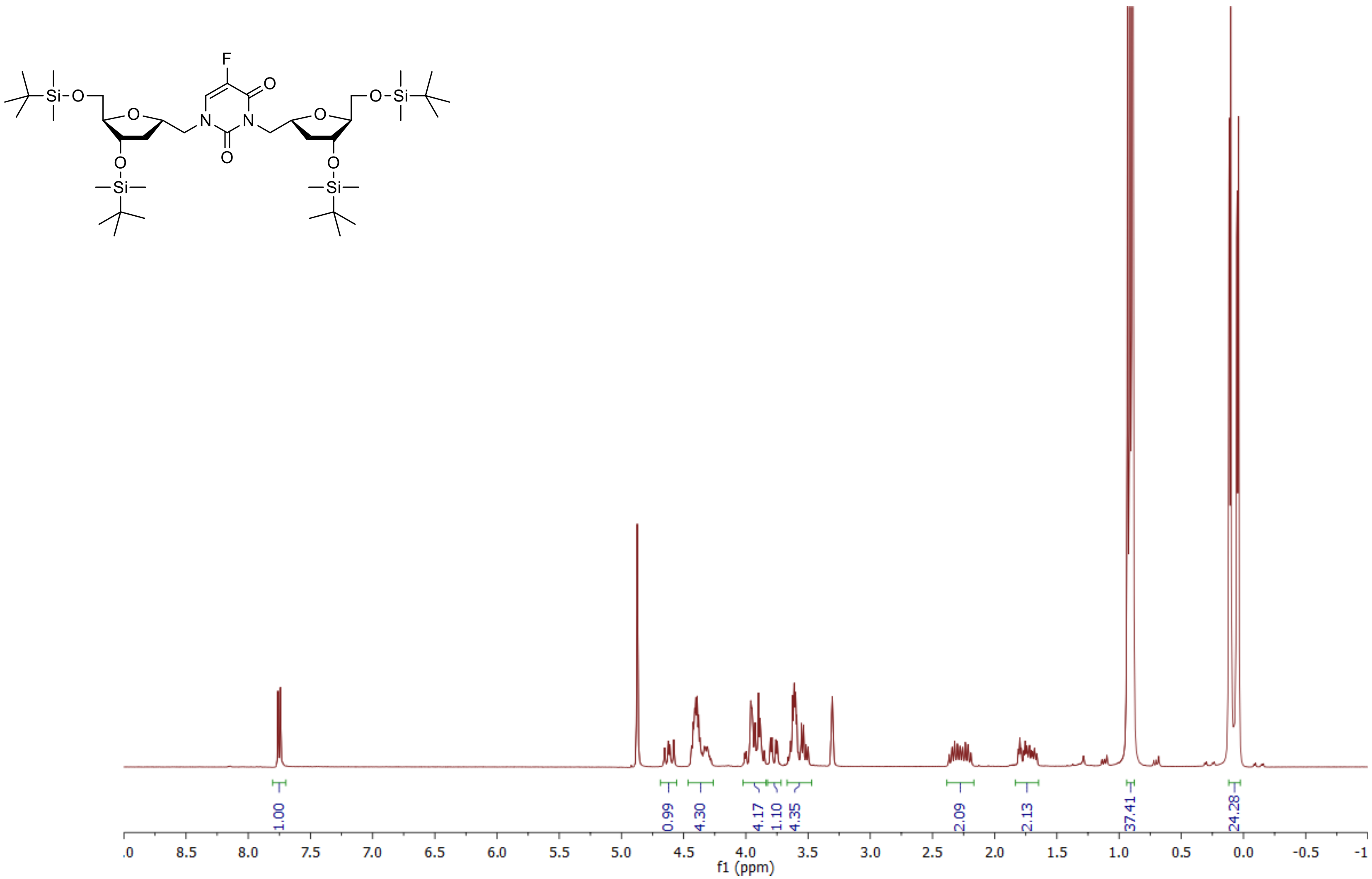

N^1,N^3-bis-[3,5-bis-*O*-(*tert*-Butyldimethylsilyl)-1,2-dideoxy-α-ribofuranosylmethyl]-5-fluorouracil (18d)

^{13}C NMR (75.5 MHz, MeOH-d_4)

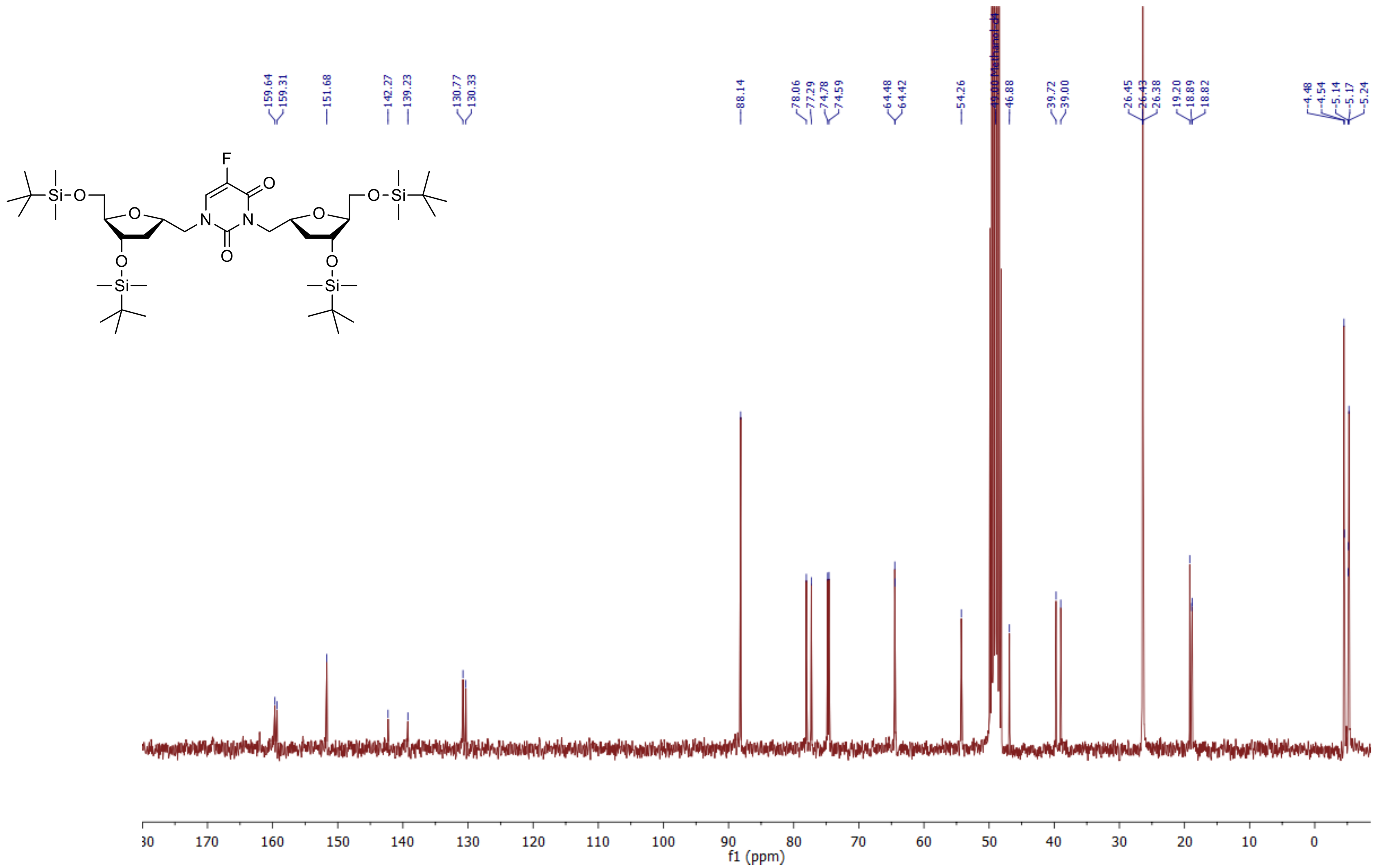

N[1],*N*[3]-bis-[3,5-bis-*O*-(*tert*-Butyldimethylsilyl)-1,2-dideoxy-α-ribofuranosylmethyl]-5-fluorouracil (18d)

DEPT 135 NMR (75.5 MHz, MeOH-d_4)

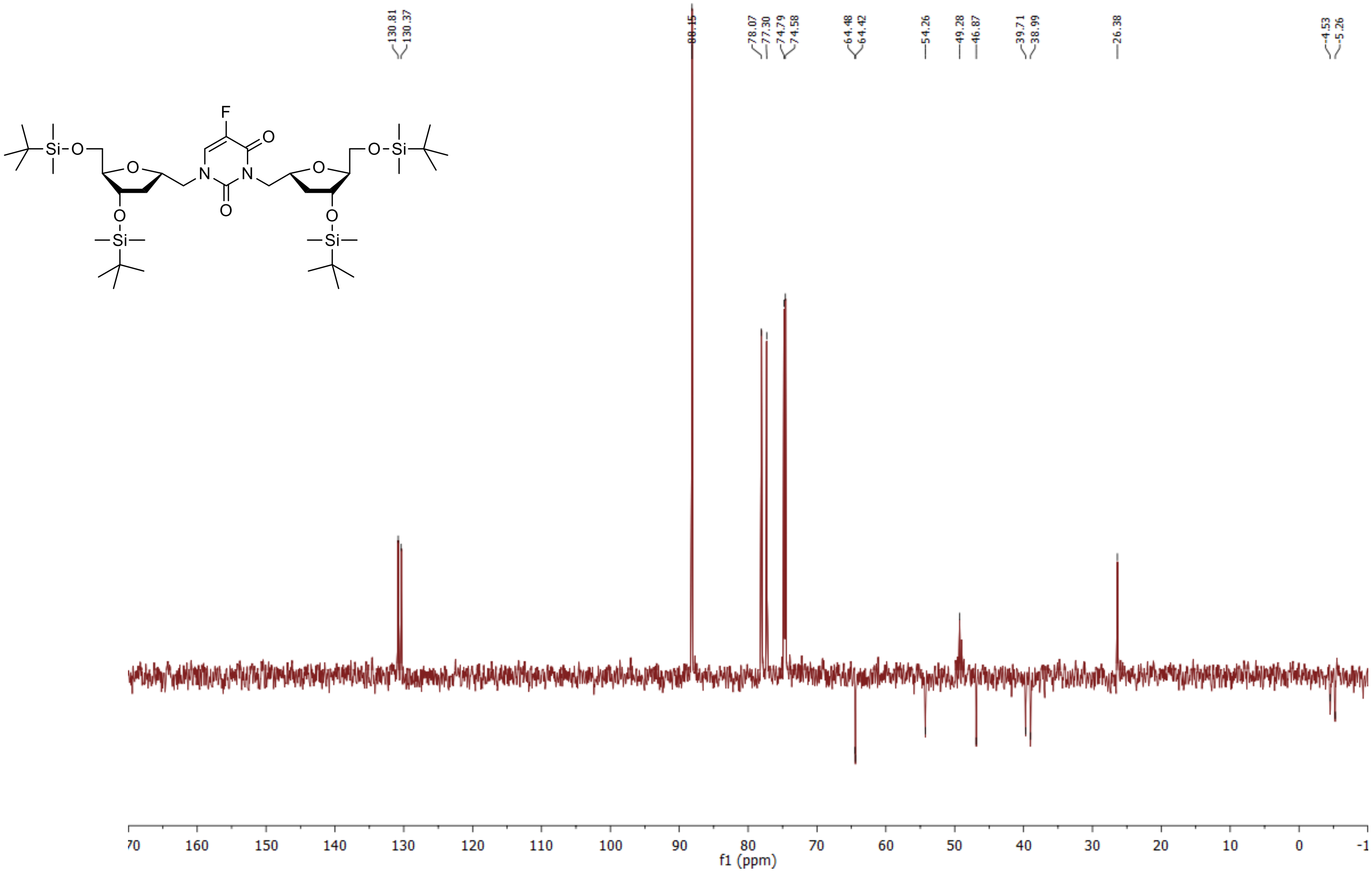

N^1,N^3-bis-[3,5-bis-*O*-(*tert*-Butyldimethylsilyl)-1,2-dideoxy-α-ribofuranosylmethyl]-5-fluorouracil (18d)

COSY NMR (MeOH-d_4)

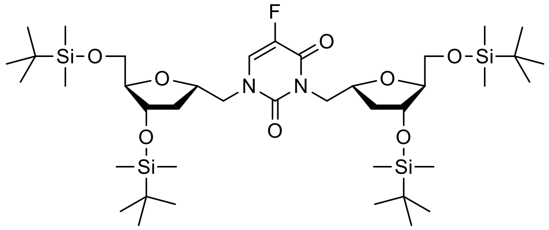

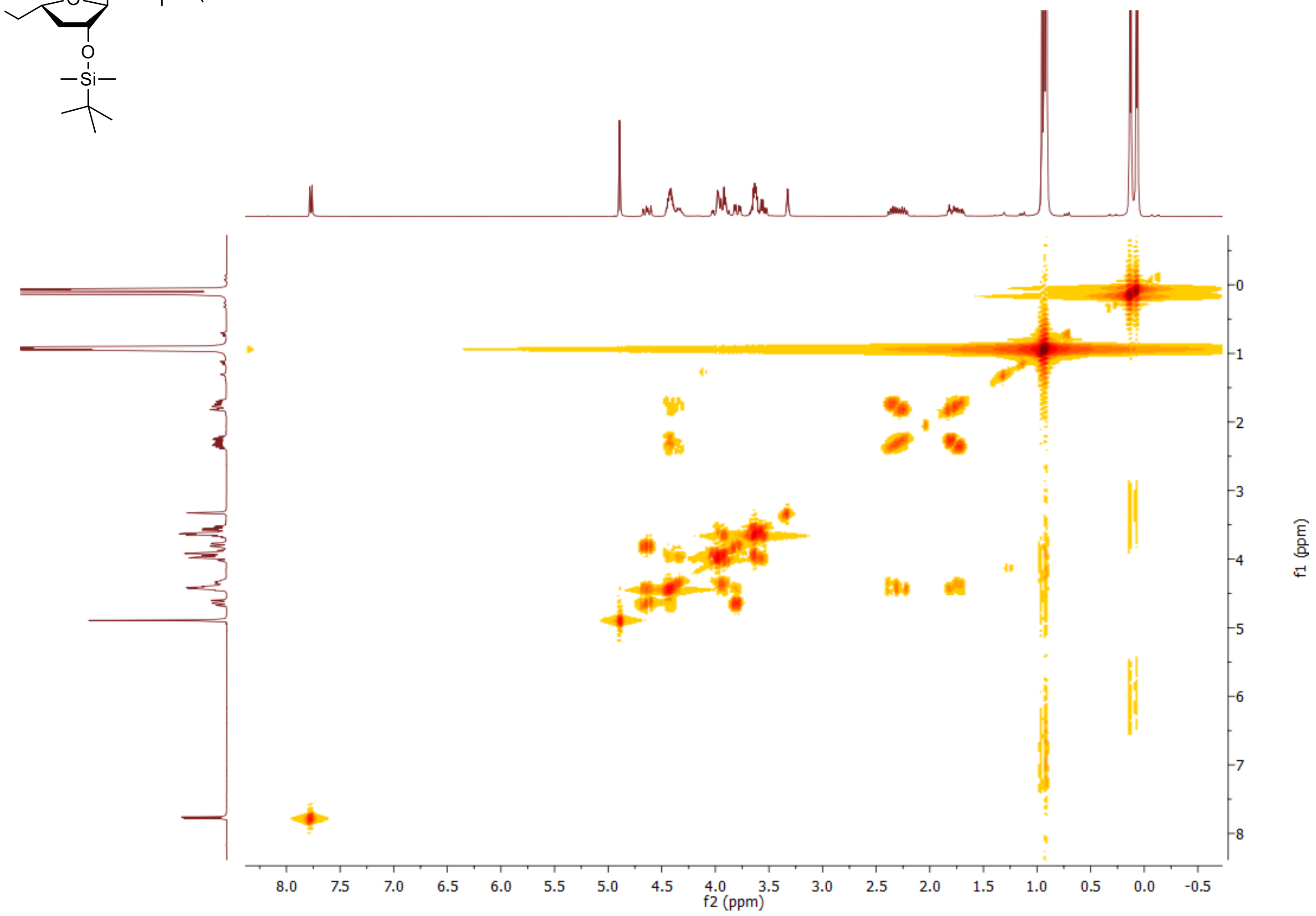

N^1,N^3-bis-[3,5-bis-*O*-(*tert*-Butyldimethylsilyl)-1,2-dideoxy-α-ribofuranosylmethyl]-5-fluorouracil (18d)

HSQC NMR (MeOH-d_4)

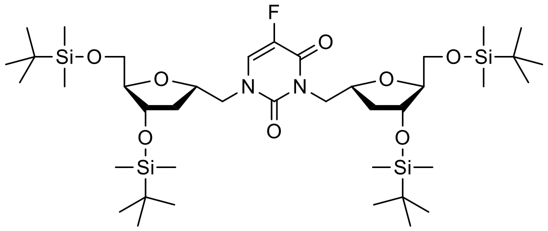

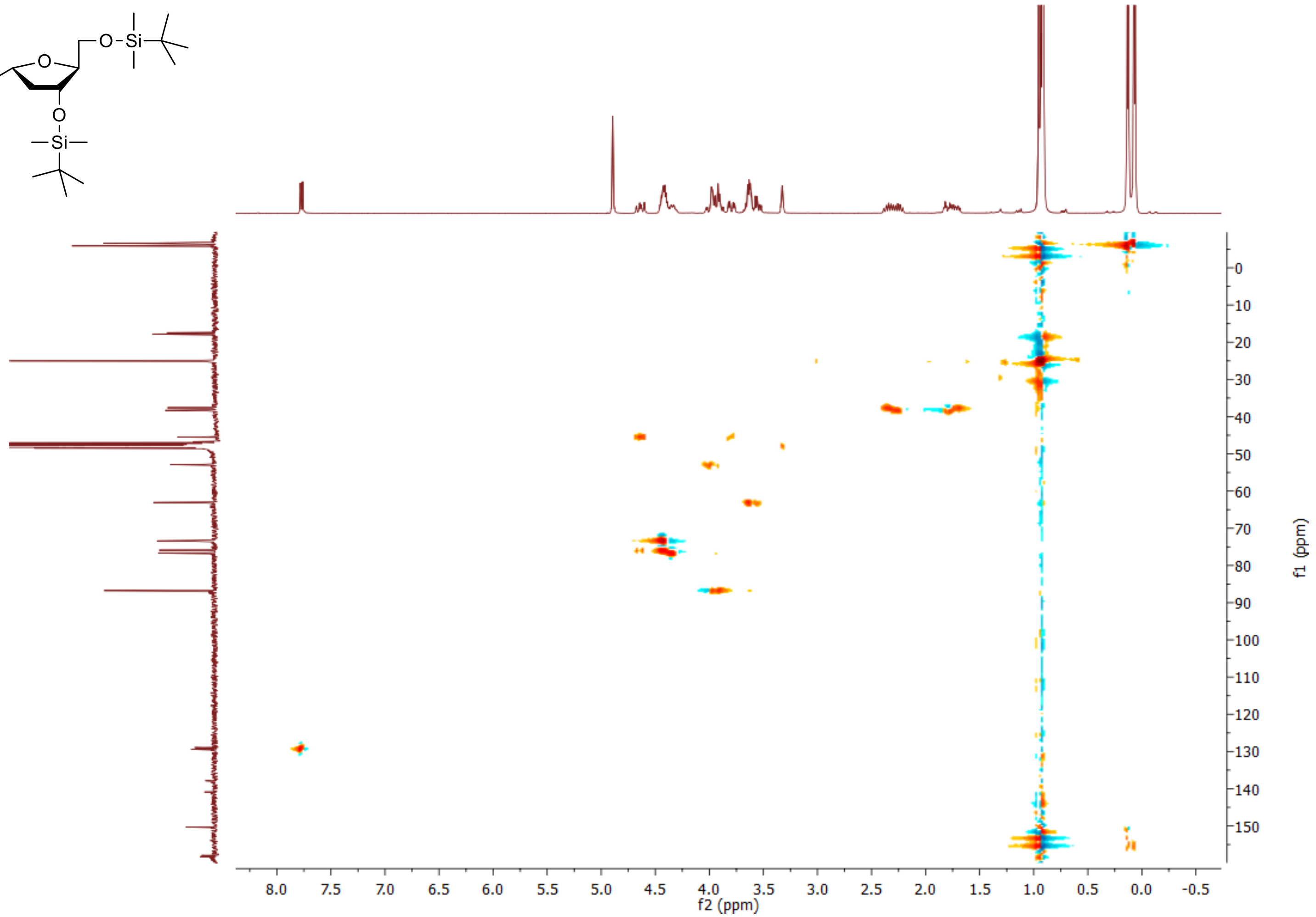

N^1,N^3-bis-[3,5-bis-*O*-(*tert*-Butyldimethylsilyl)-1,2-dideoxy-α-ribofuranosylmethyl]-5-fluorouracil (18d)

HMBC NMR (MeOH-d_4)

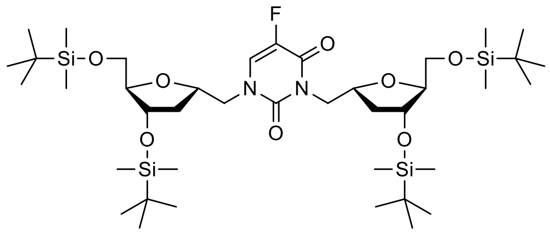

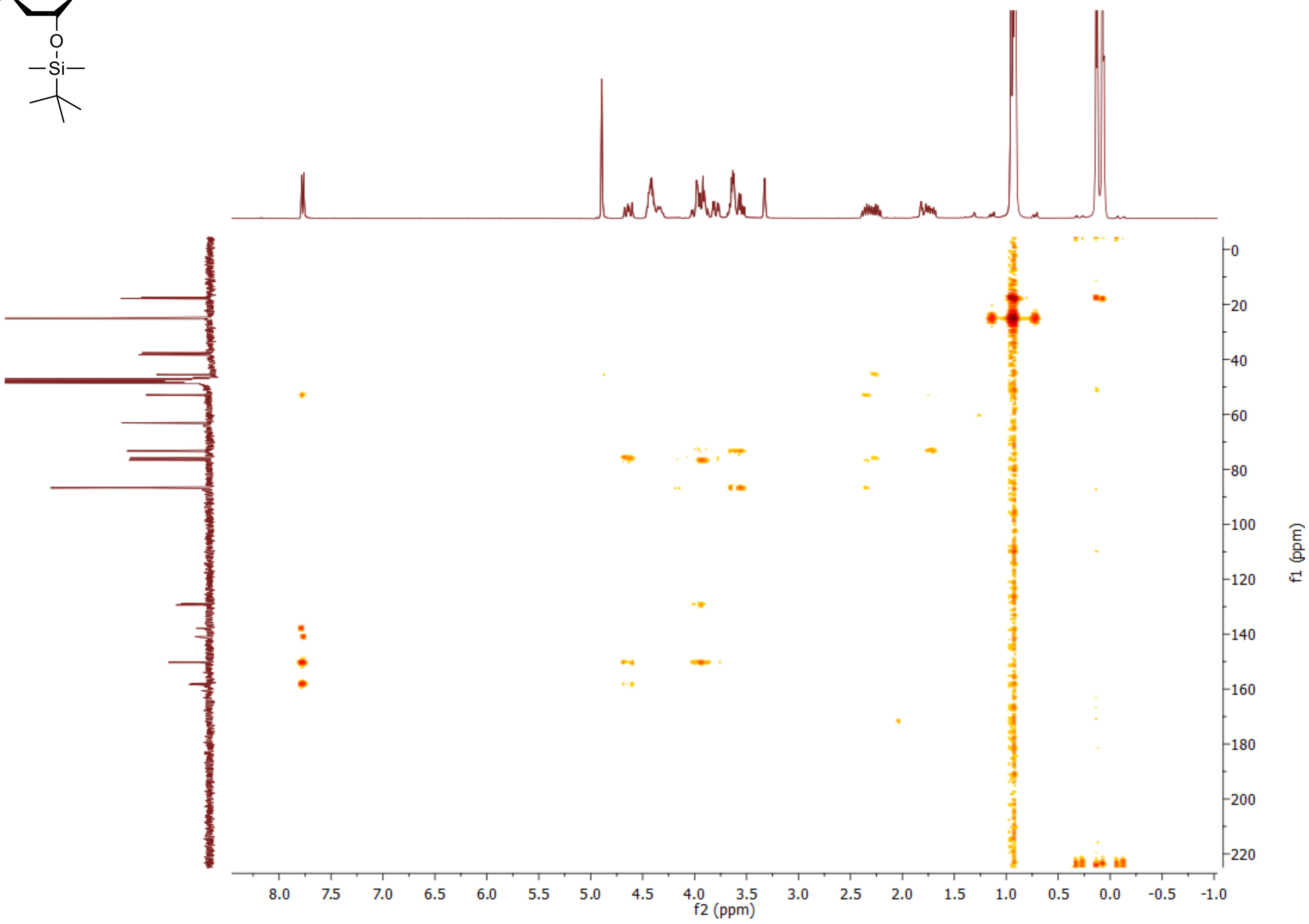

N^1,N^3-bis-[3,5-bis-*O*-(*tert*-Butyldimethylsilyl)-1,2-dideoxy-α-ribofuranosylmethyl]-5-iodouracil (18f)

^{1}H NMR (300.13 MHz, MeOH-d_4)

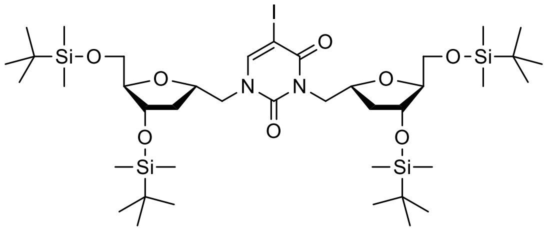

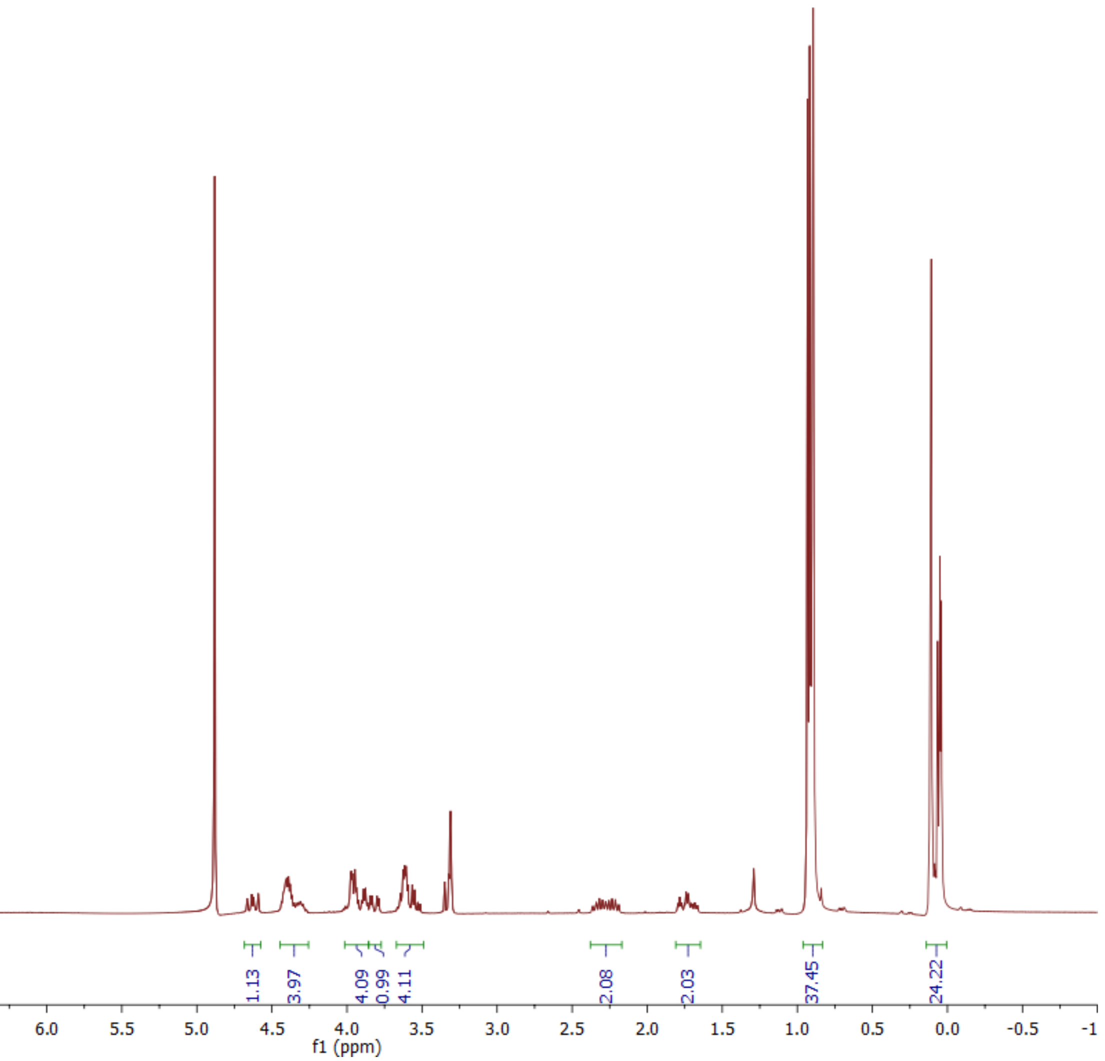

N^1,N^3-bis-[3,5-bis-*O*-(*tert*-Butyldimethylsilyl)-1,2-dideoxy-α-ribofuranosylmethyl]-5-iodouracil (18f)

^{13}C NMR (75.5 MHz, MeOH-d_4)

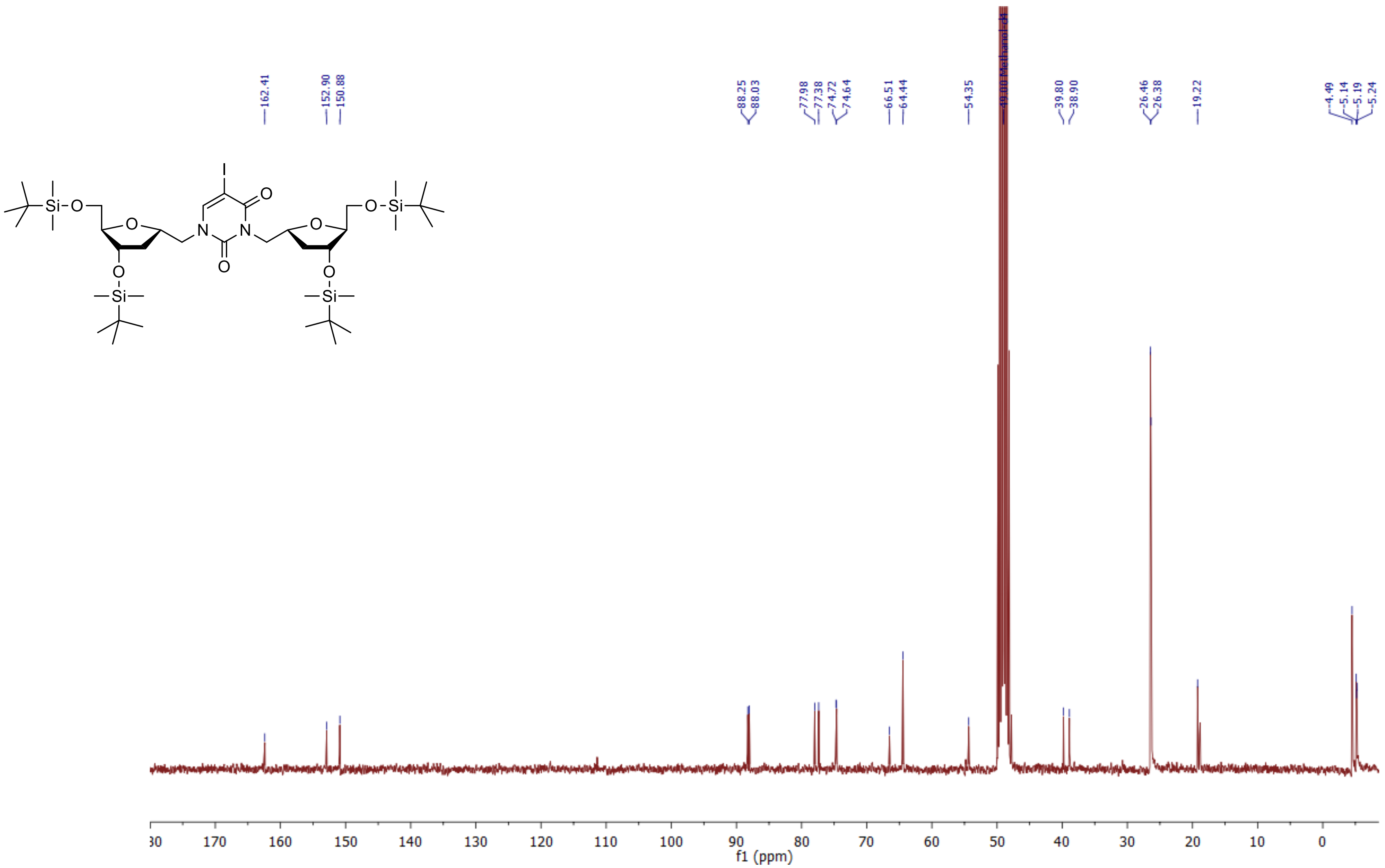

N^1,N^3-bis-[3,5-bis-*O*-(*tert*-Butyldimethylsilyl)-1,2-dideoxy-α-ribofuranosylmethyl]-5-iodouracil (18f)

DEPT 135 NMR (75.5 MHz, MeOH-d_4)

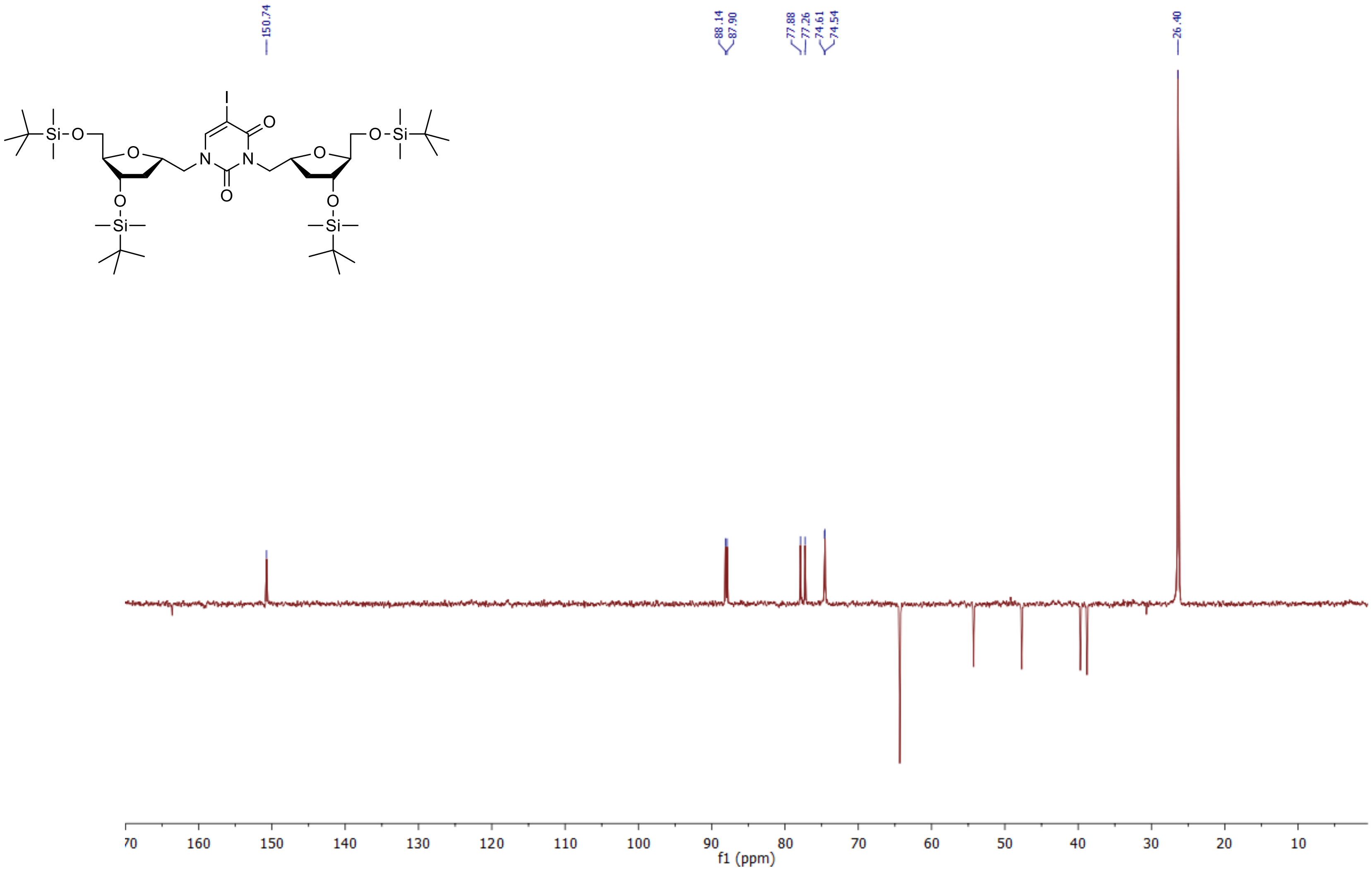

N^1,N^3-bis-[3,5-bis-*O*-(*tert*-Butyldimethylsilyl)-1,2-dideoxy-α-ribofuranosylmethyl]-5-iodouracil (18f)

COSY NMR (MeOH-d_4)

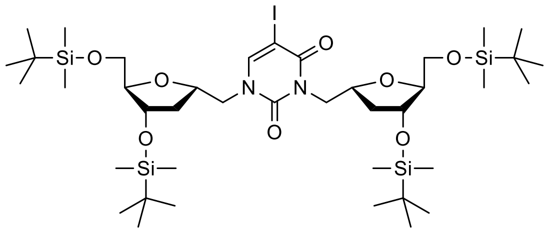

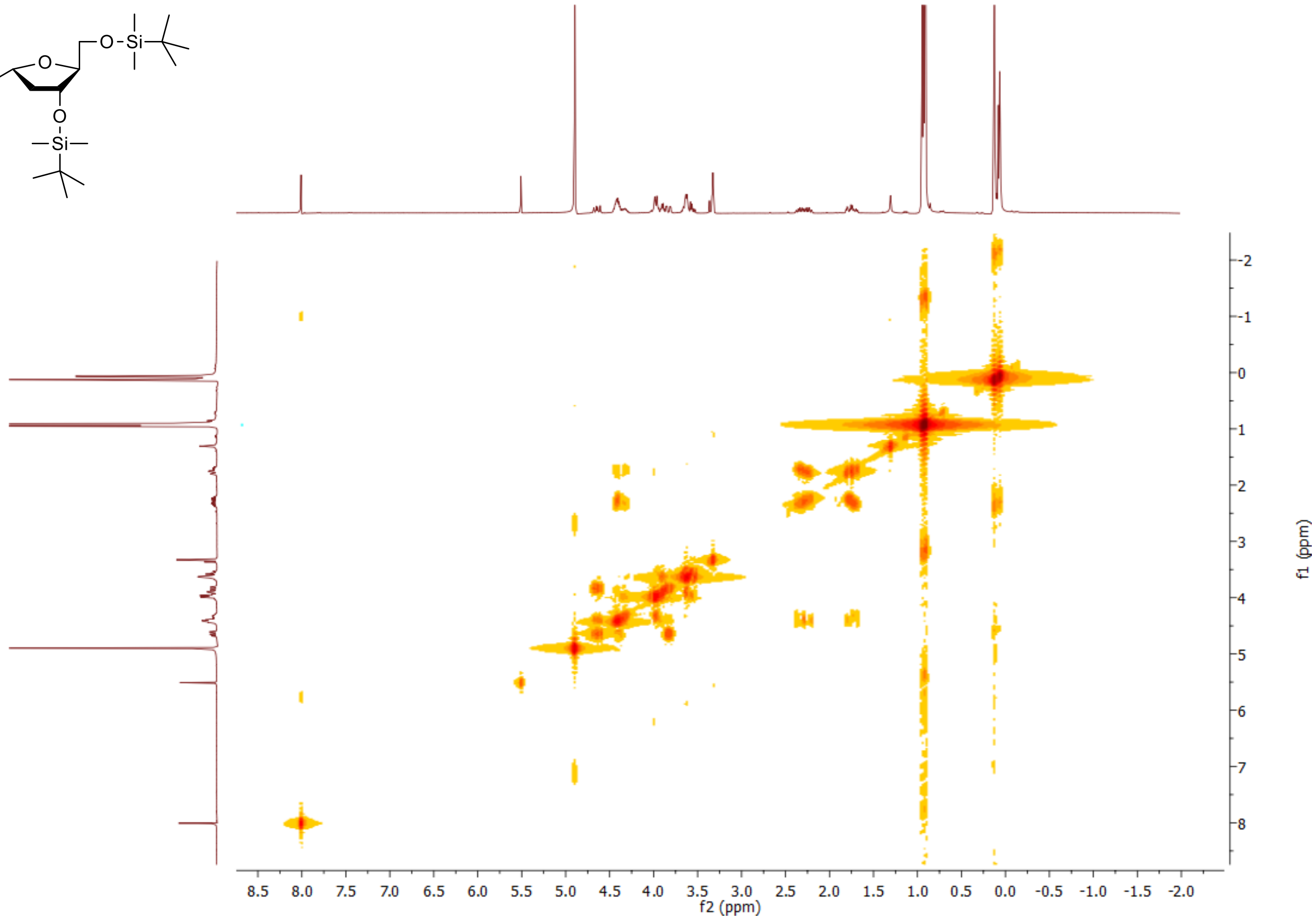

N^1,N^3-bis-[3,5-bis-*O*-(*tert*-Butyldimethylsilyl)-1,2-dideoxy-α-ribofuranosylmethyl]-5-iodouracil (18f)

HSQC NMR (MeOH-d_4)

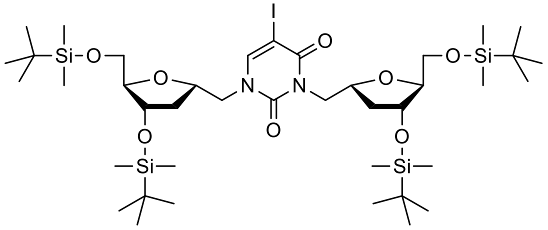

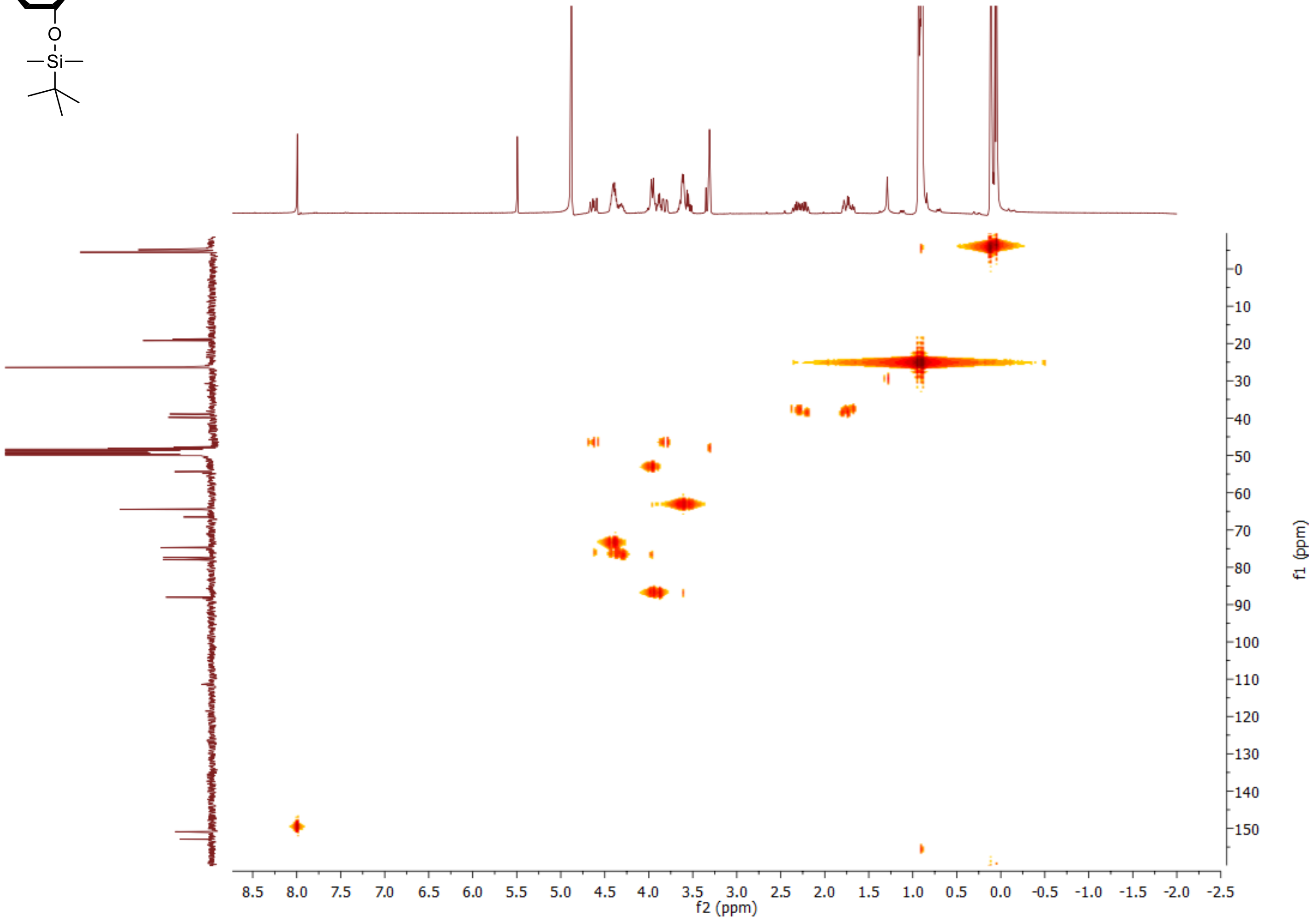

N^1,N^3-bis-[3,5-bis-*O*-(*tert*-Butyldimethylsilyl)-1,2-dideoxy-α-ribofuranosylmethyl]-5-iodouracil (18f)

HMBC NMR (MeOH-d_4)

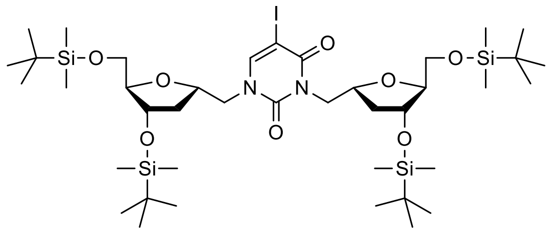

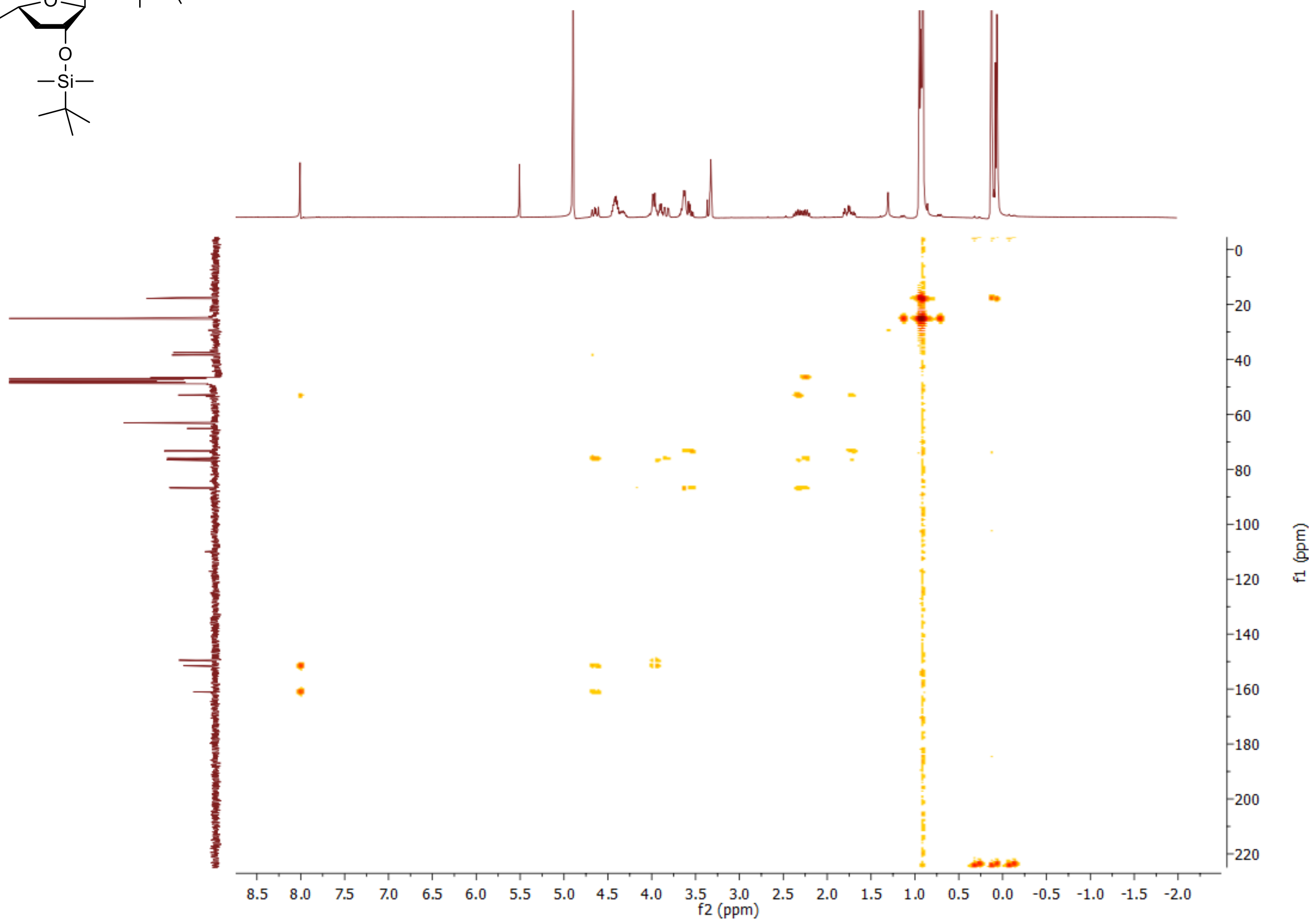

3,5-bis-*O*-(*tert*-Butyldimethylsilyl)-1,2-dideoxy-1α-((acetoxy)methyl)-ᴅ-ribofuranose (19)

^{1}H NMR (300.13 MHz, MeOH-d_4)

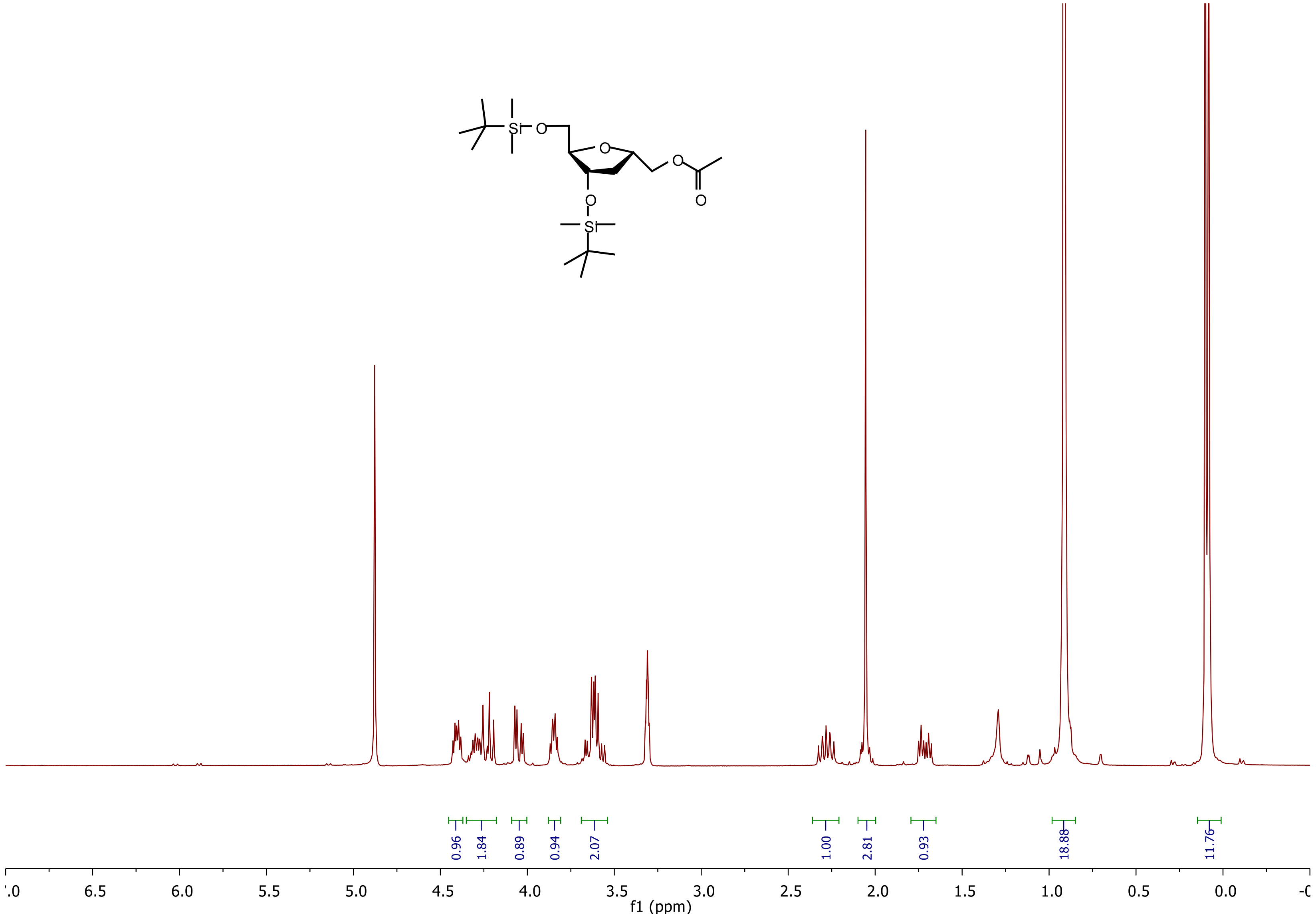

3,5-bis-*O*-(*tert*-Butyldimethylsilyl)-1,2-dideoxy-1α-((acetoxy)methyl)-D-ribofuranose (19)

^{13}C NMR (75.5 MHz, MeOH-d_4)

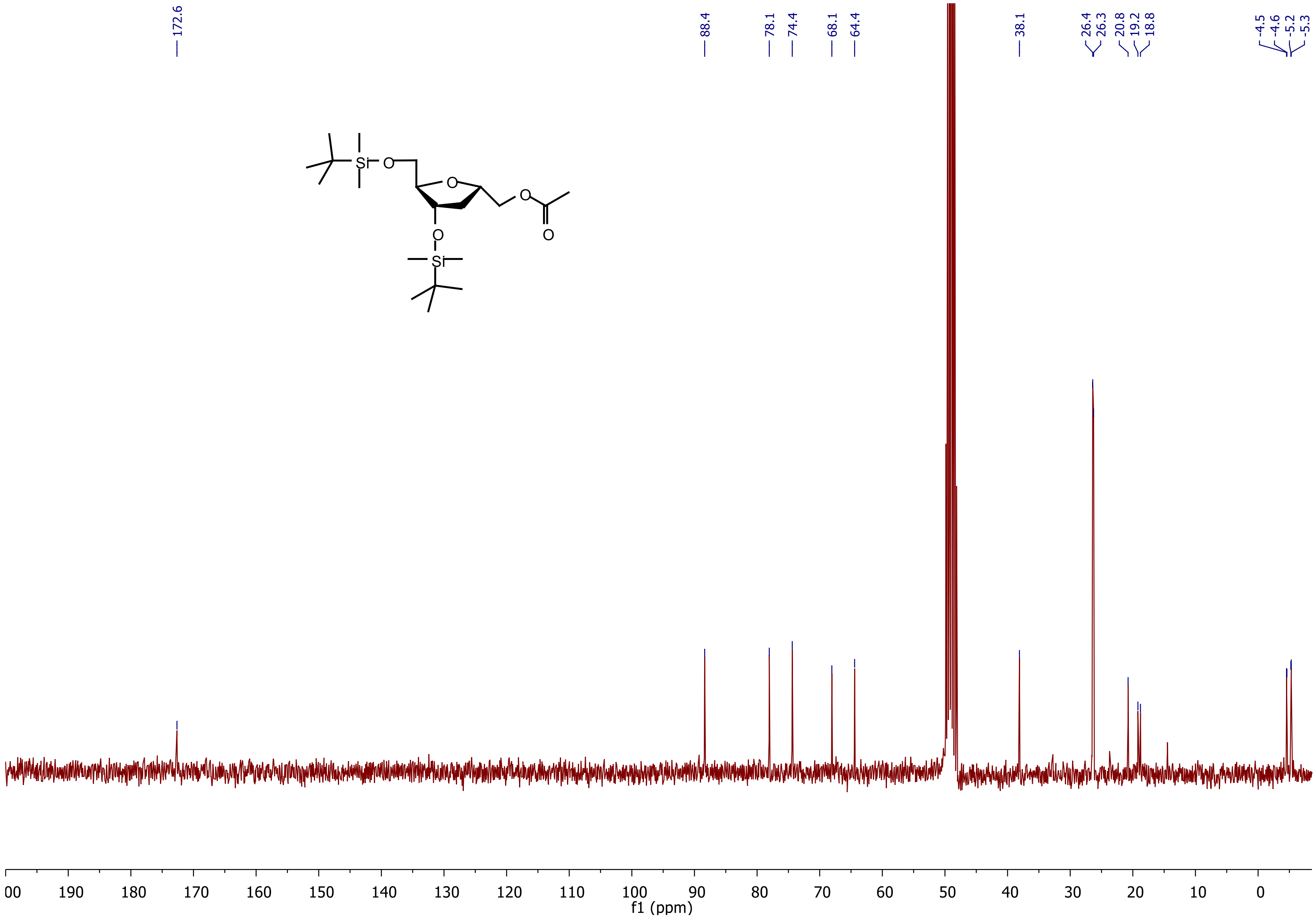

3,5-bis-*O*-(*tert*-Butyldimethylsilyl)-1,2-dideoxy-1α-((acetoxy)methyl)-D-ribofuranose (19)

DEPT NMR (75.5 MHz, MeOH-d_4)

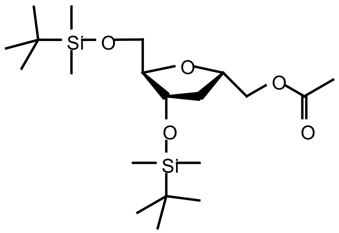

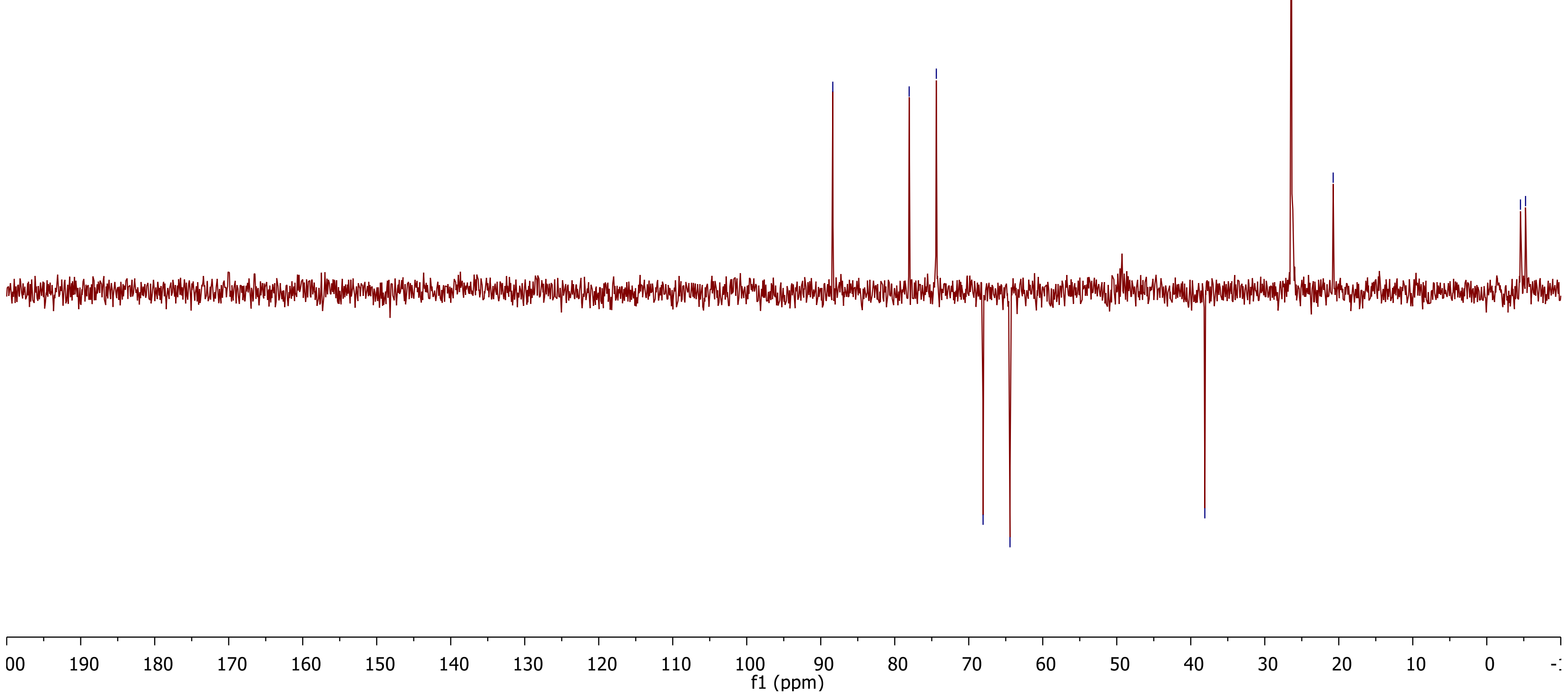

3,5-bis-*O*-(*tert*-Butyldimethylsilyl)-1,2-dideoxy-1α-((tosyloxy)methyl)-D-ribofuranose (20)

^{1}H NMR (300.13 MHz, MeOH-d_4)

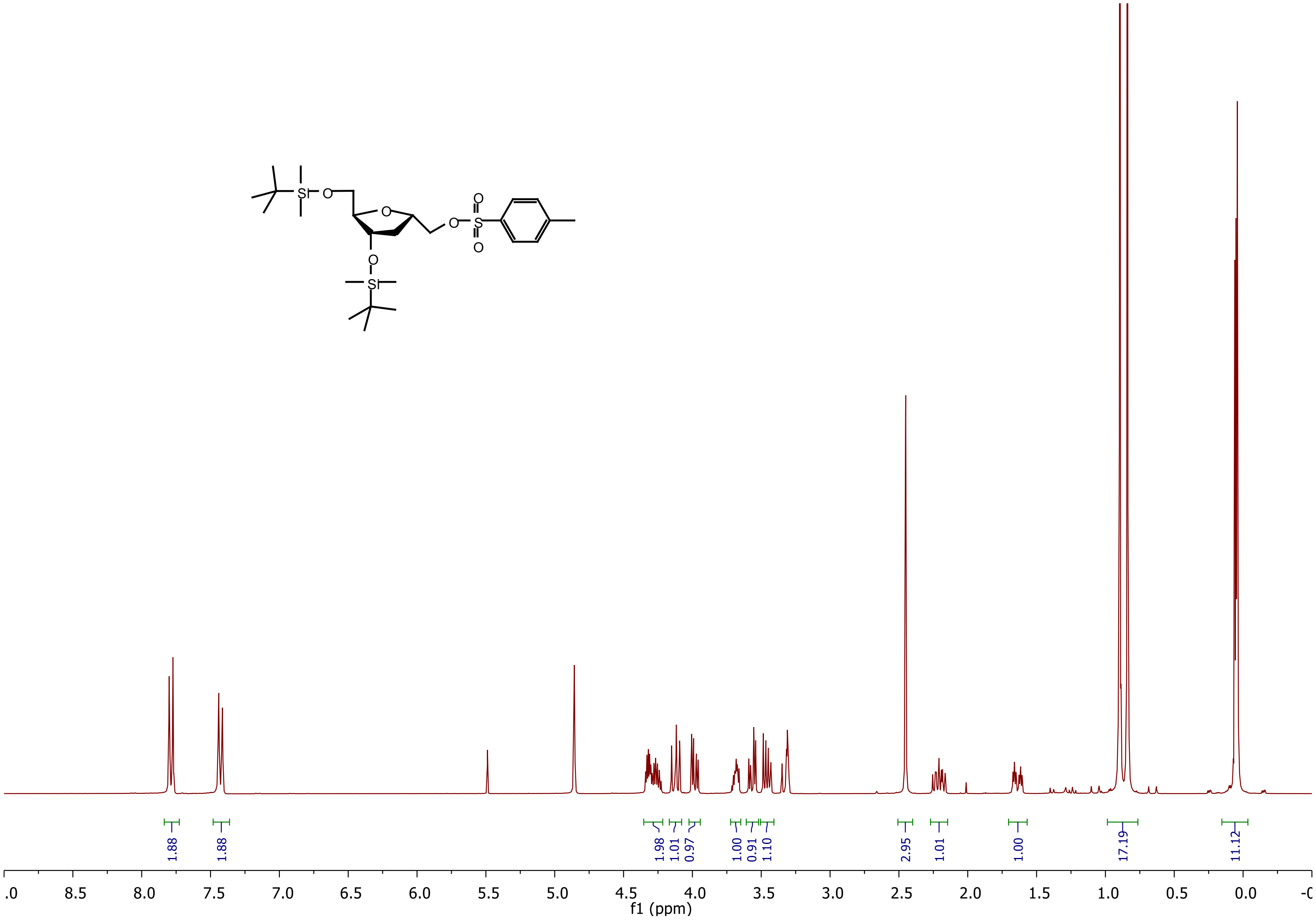

3,5-bis-*O*-(*tert*-Butyldimethylsilyl)-1,2-dideoxy-1α-((tosyloxy)methyl)-D-ribofuranose (20)

^{13}C NMR (75.5 MHz, MeOH-d_4)

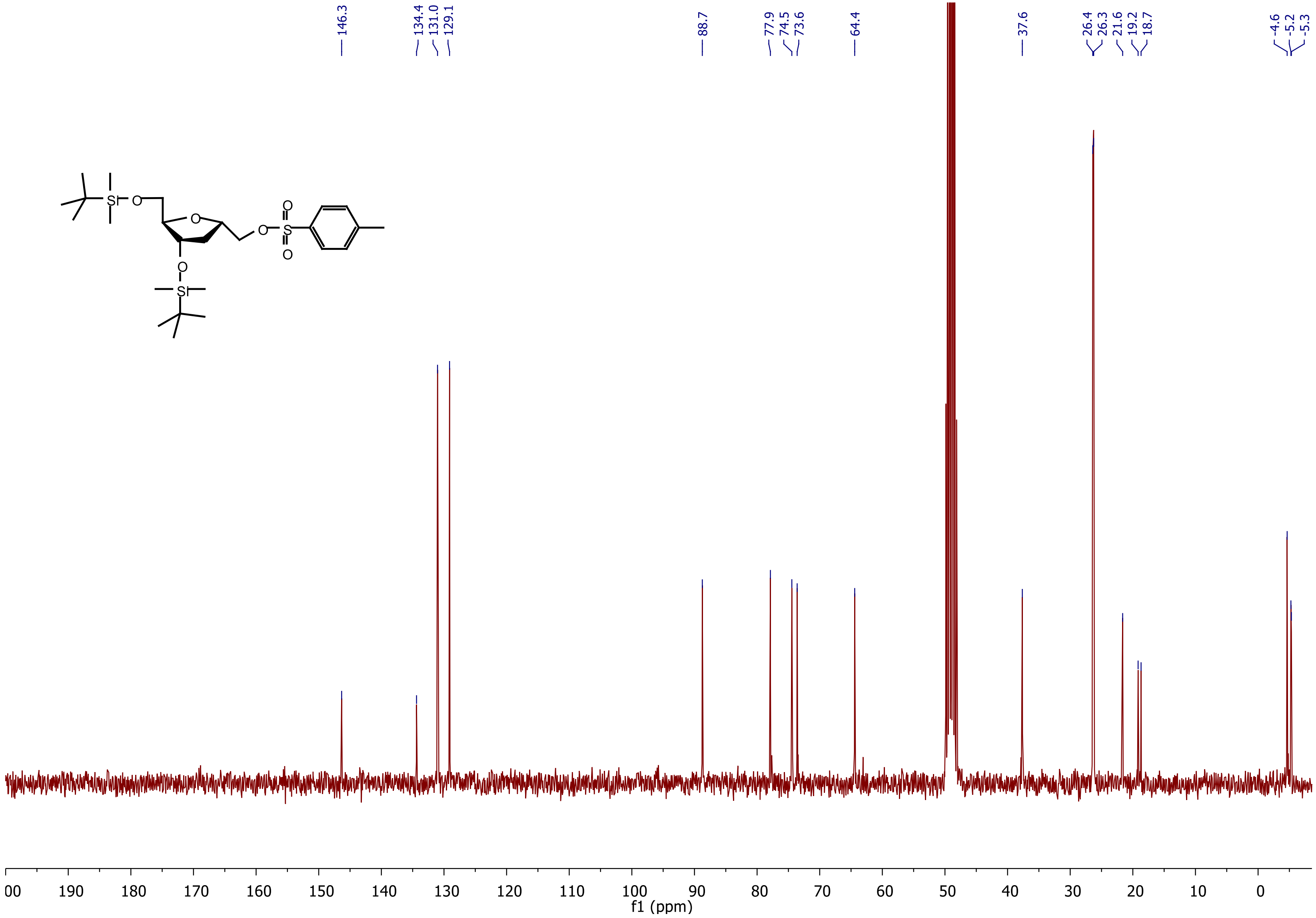

3,5-bis-*O*-(*tert*-Butyldimethylsilyl)-1,2-dideoxy-1α-((tosyloxy)methyl)-D-ribofuranose (20)

DEPT NMR (75.5 MHz, MeOH-d_4)

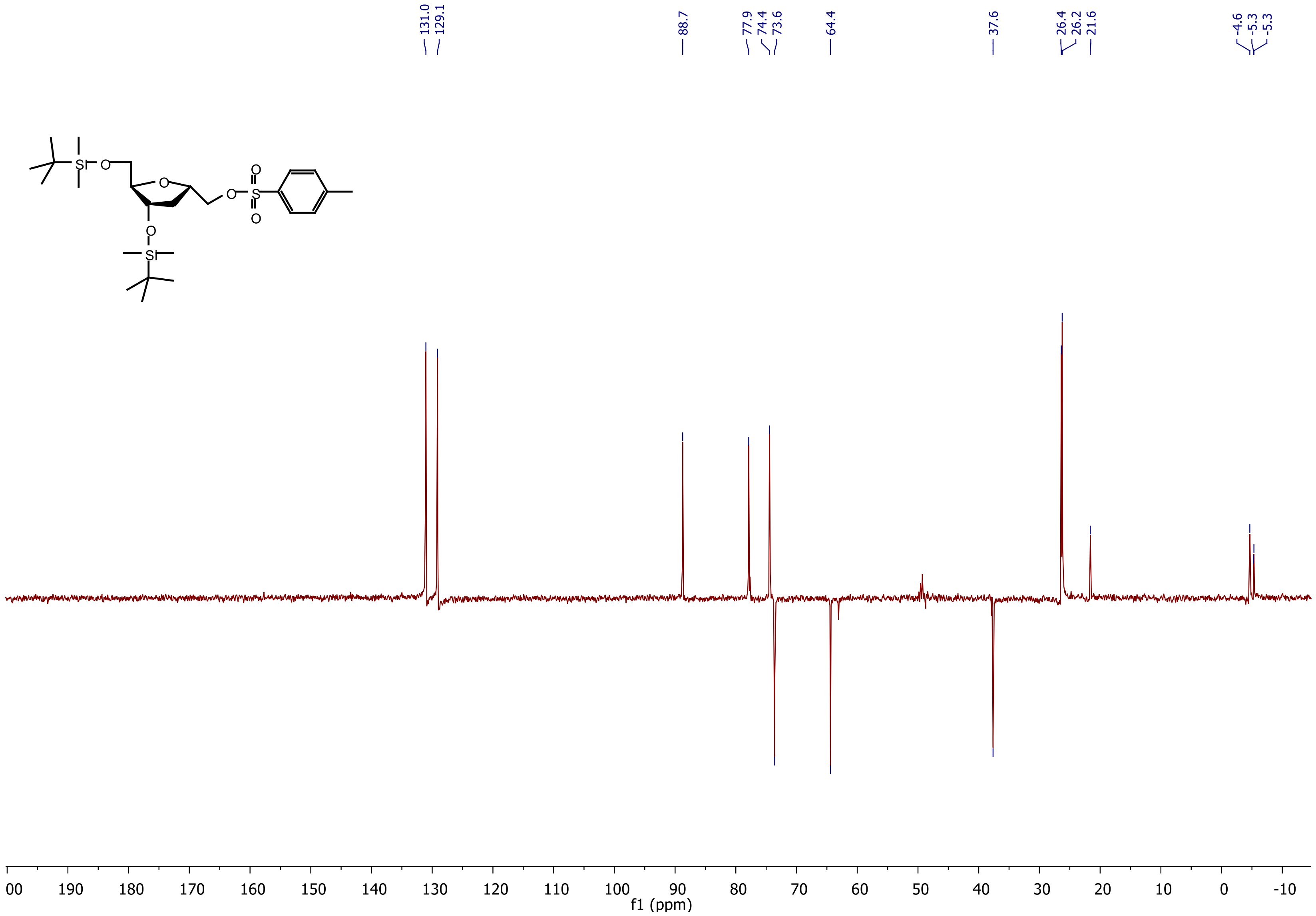

3,5-bis-*O*-(*tert*-Butyldimethylsilyl)-1,2-dideoxy-1α-((tosyloxy)methyl)-D-ribofuranose (20)

COSY NMR (MeOH-d_4)

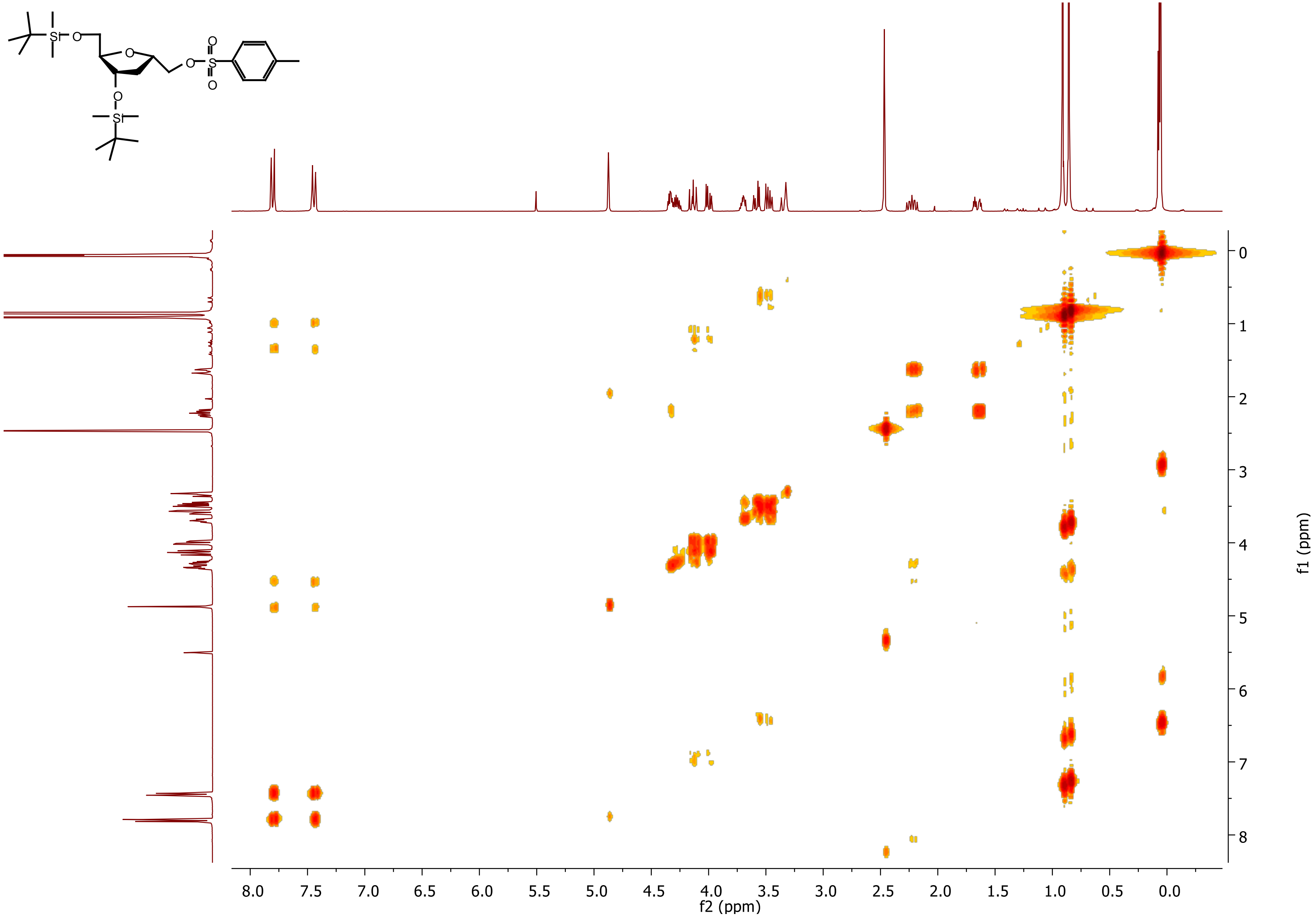

3,5-bis-*O*-(*tert*-Butyldimethylsilyl)-1,2-dideoxy-1α-((tosyloxy)methyl)-D-ribofuranose (20)

HSQC NMR (MeOH-d_4)

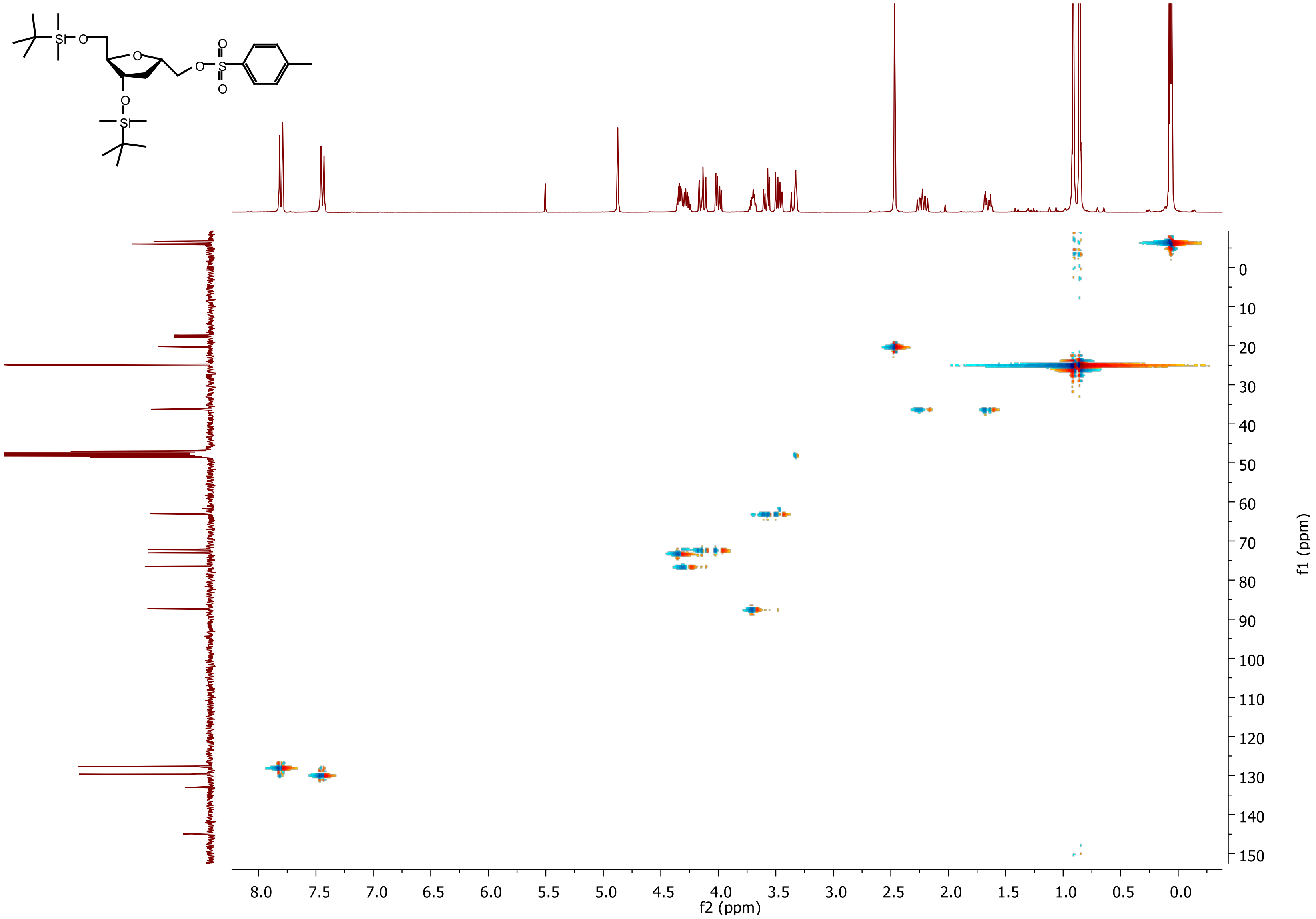

3,5-bis-*O*-(*tert*-Butyldimethylsilyl)-1,2-dideoxy-1α-((tosyloxy)methyl)-D-ribofuranose (20)

HMBC NMR (MeOH-d_4)

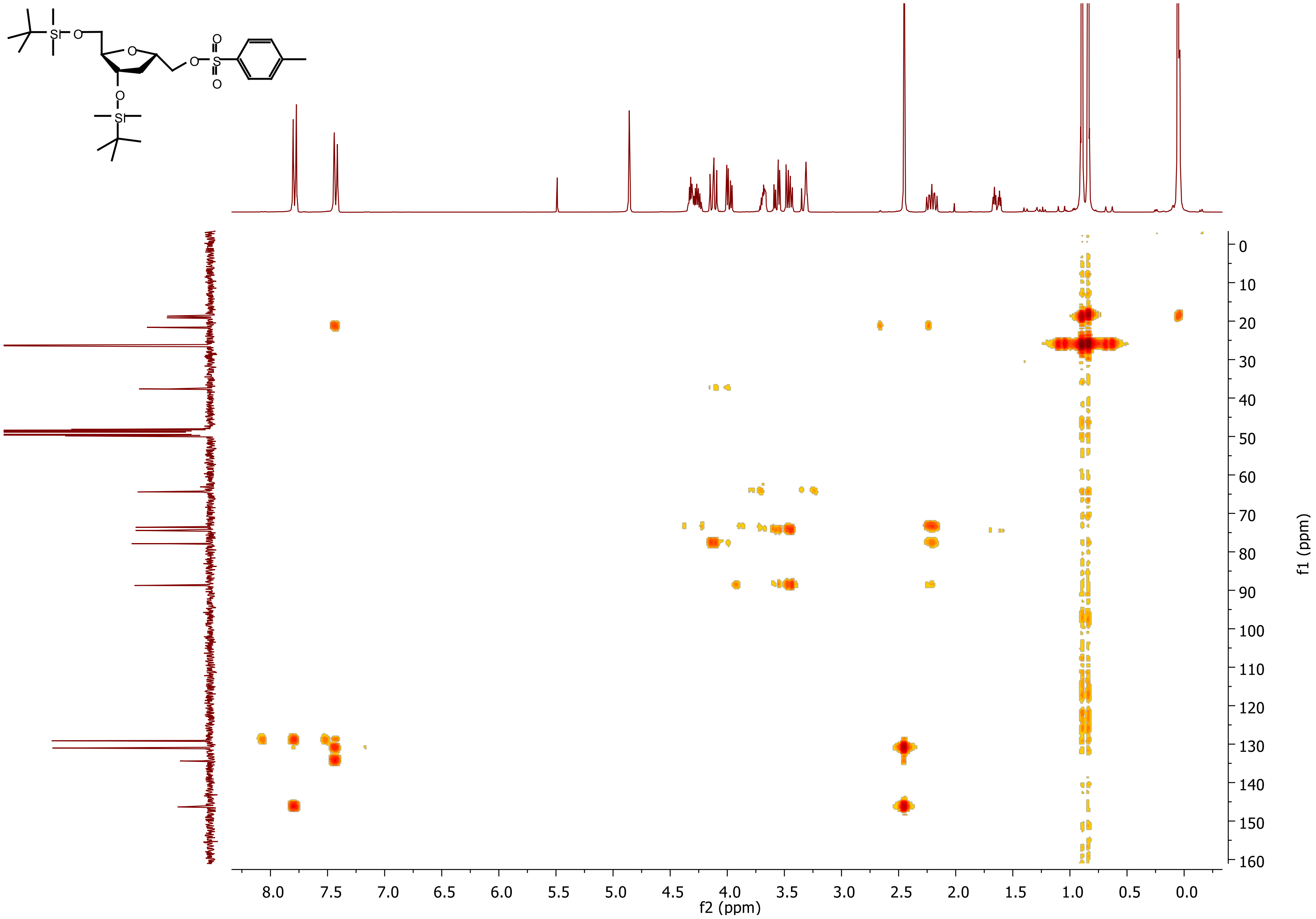

N^1,N^3-bis-(1,2-Dideoxy-α-ribofuranosylmethyl)-5-fluorouracil (21d)

^{1}H NMR (300.13 MHz, MeOH-d_4)

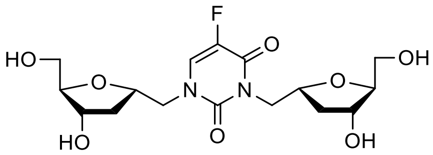

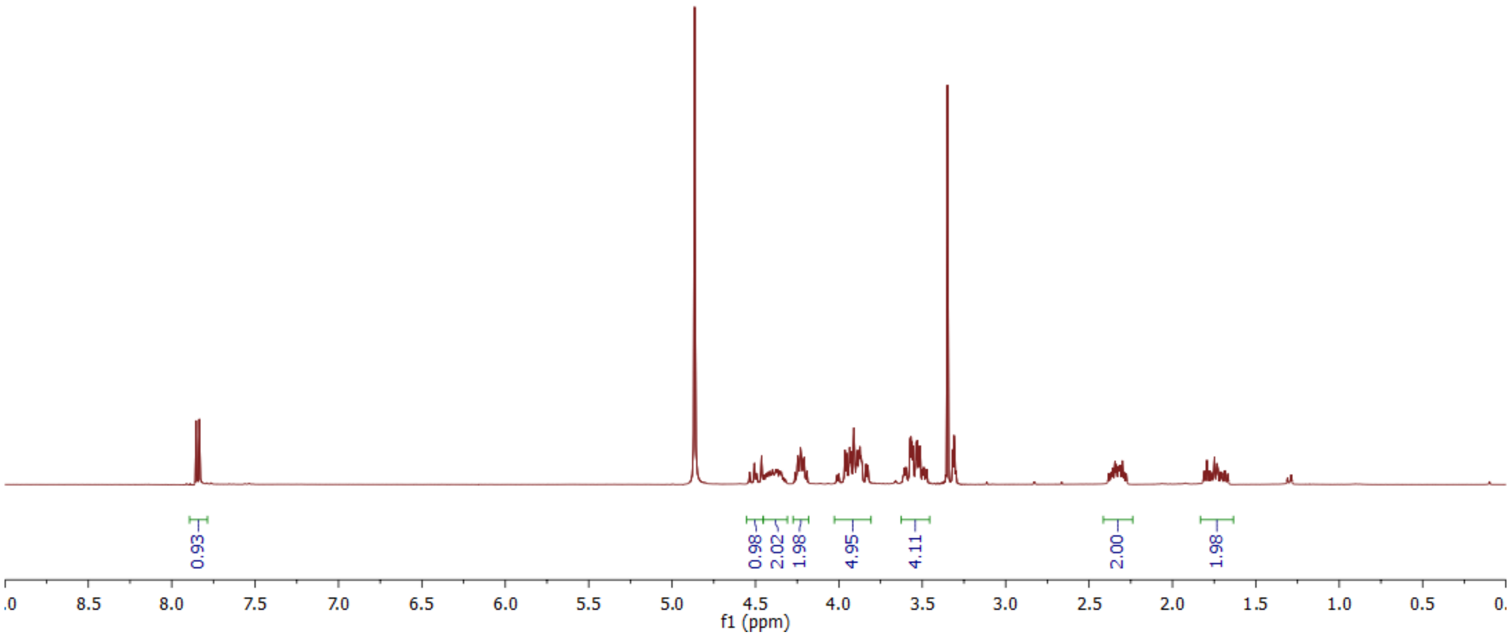

N^1,N^3-bis-(1,2-Dideoxy-α-ribofuranosylmethyl)-5-fluorouracil (21d)

^{13}C NMR (75.5 MHz, MeOH-d_4)

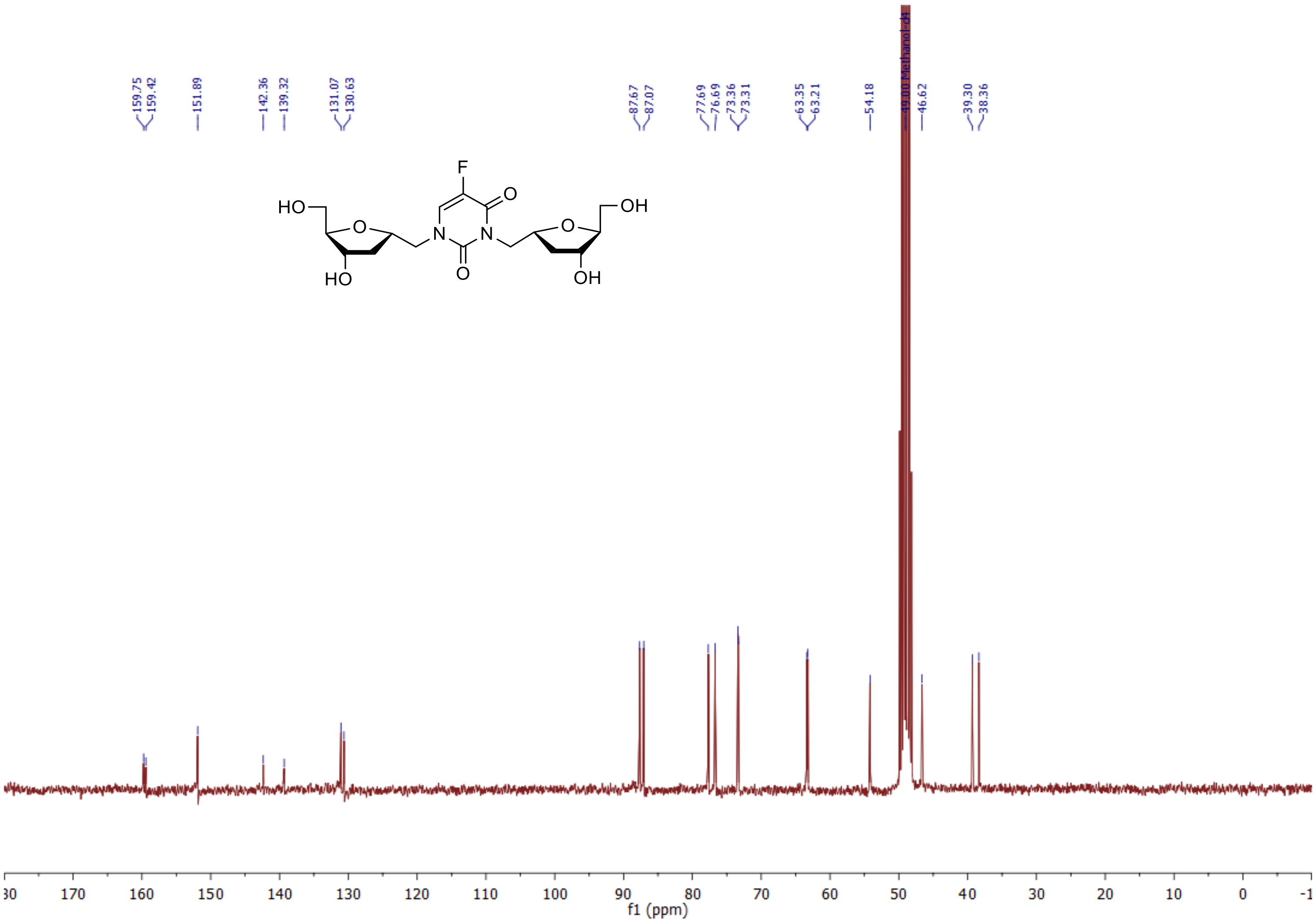

N^1,N^3-bis-(1,2-Dideoxy-α-ribofuranosylmethyl)-5-fluorouracil (21d)

DEPT 135 NMR (75.5 MHz, MeOH-d_4)

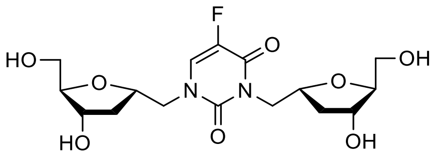

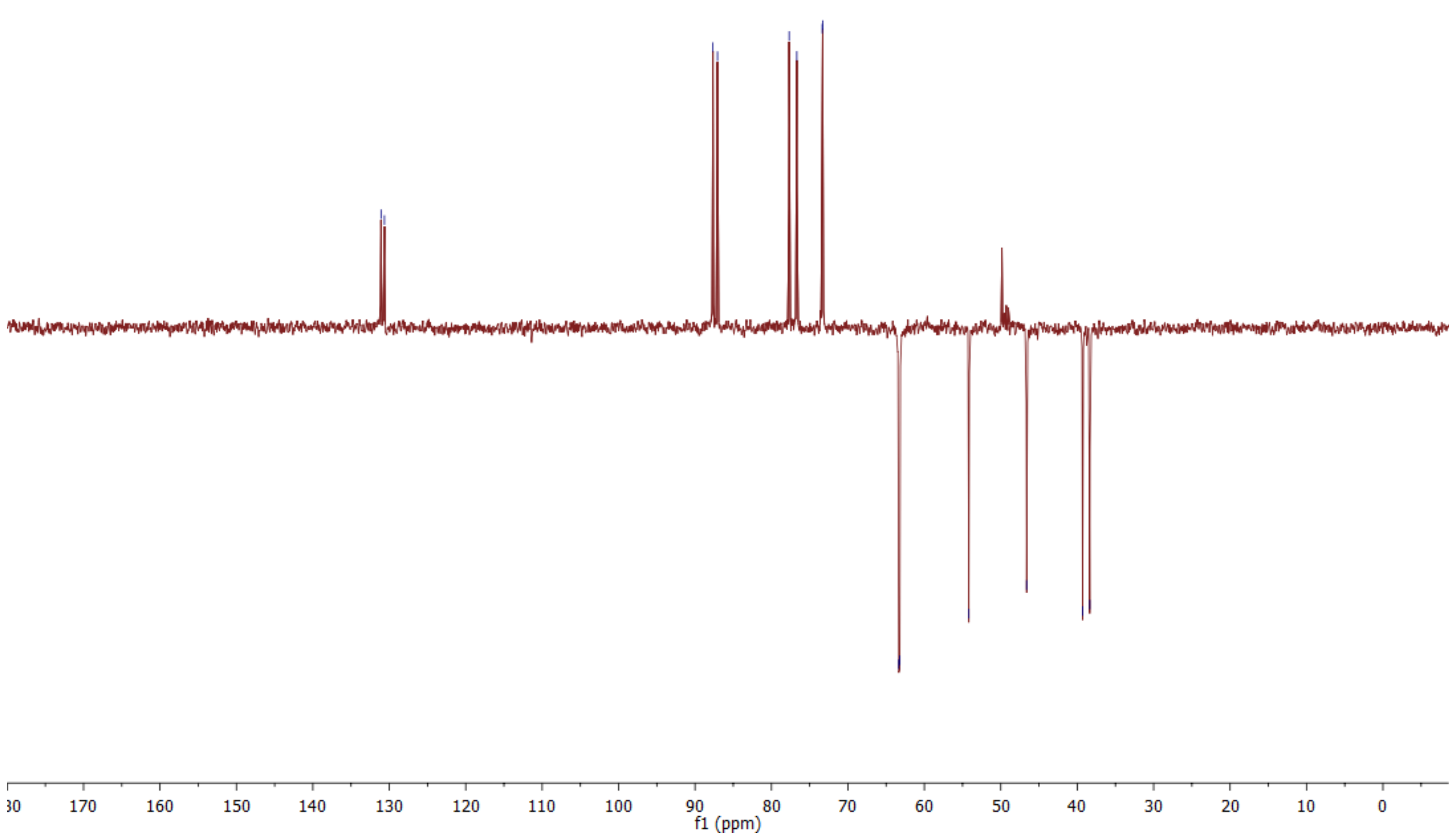

N^1,N^3-bis-(1,2-Dideoxy-α-ribofuranosylmethyl)-5-fluorouracil (21d)

COSY NMR (MeOH-d_4)

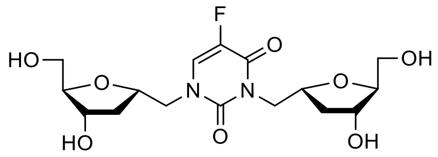

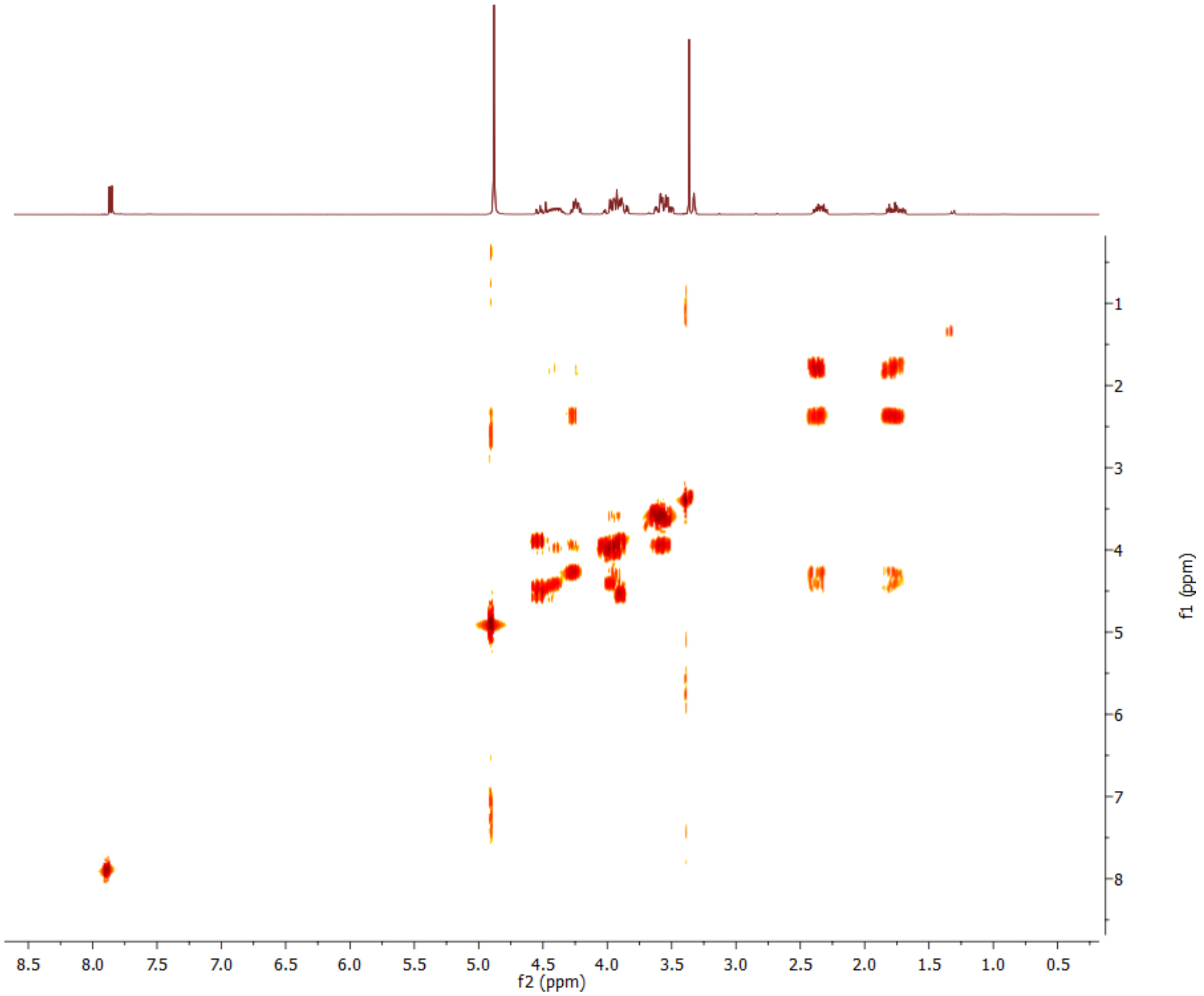

N^1,N^3-bis-(1,2-Dideoxy-α-ribofuranosylmethyl)-5-fluorouracil (21d)

HSQC NMR (MeOH-d_4)

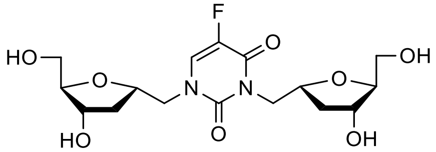

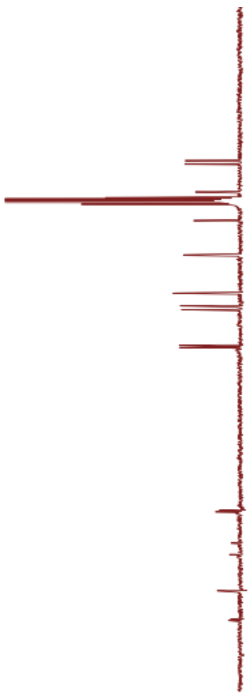

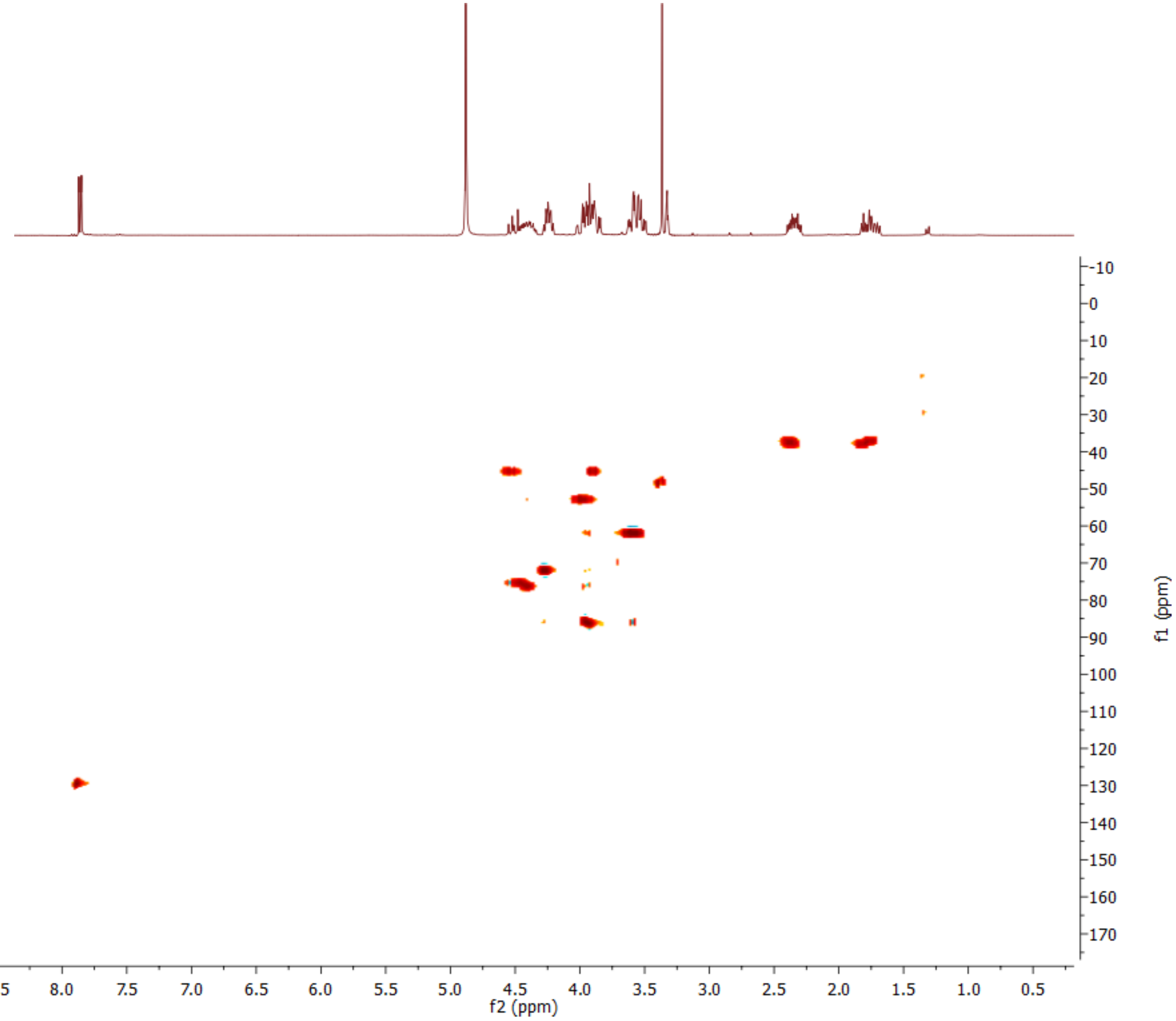

N^1,N^3-bis-(1,2-Dideoxy-α-ribofuranosylmethyl)-5-fluorouracil (21d)

HMBC NMR (MeOH-d_4)

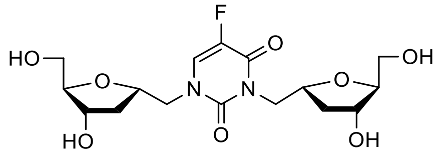

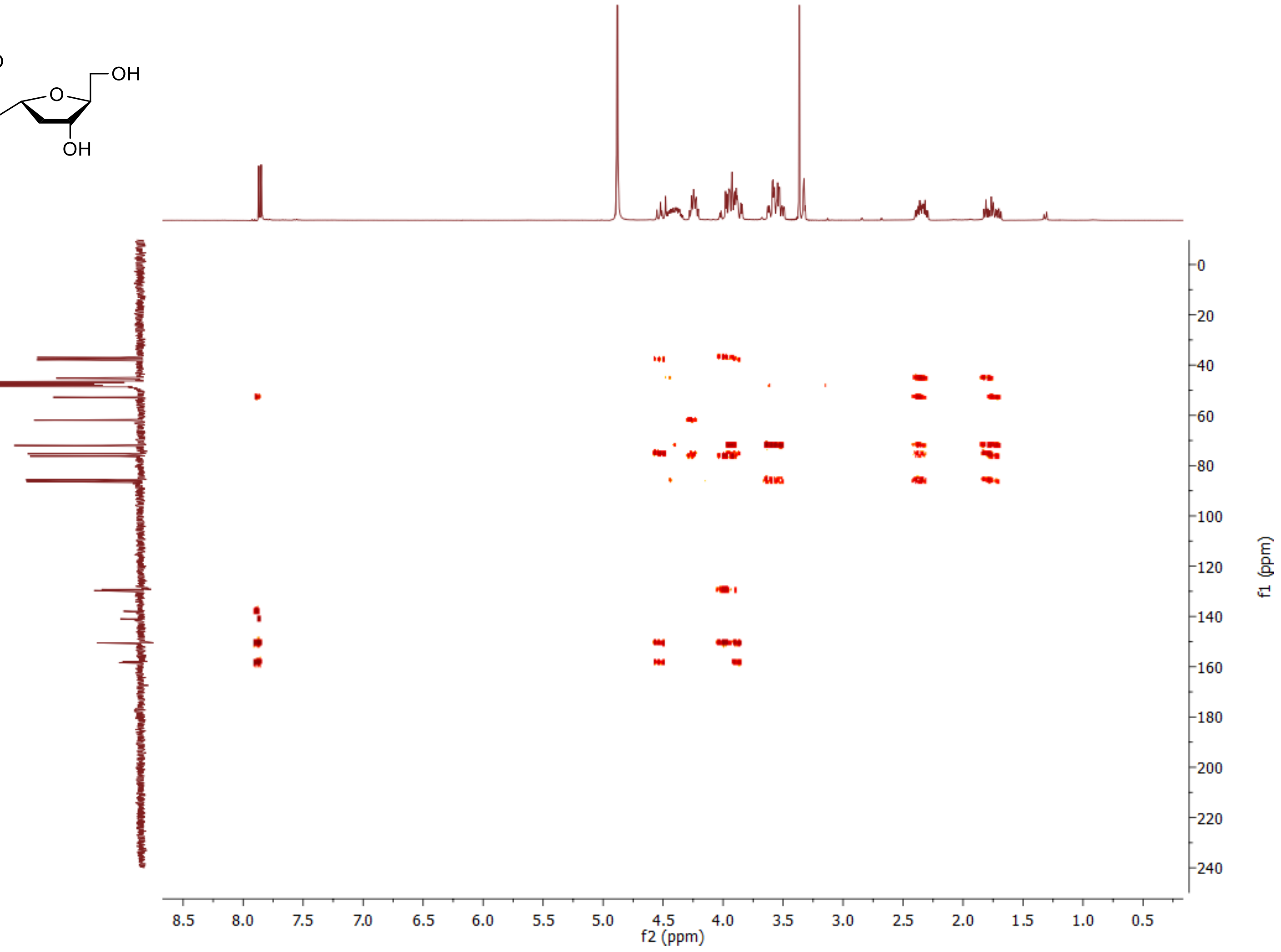

N^1,N^3-bis-(1,2-Dideoxy-α-ribofuranosylmethyl)-5-iodouracil (21f)

^{1}H NMR (300.13 MHz, MeOH-d_4)

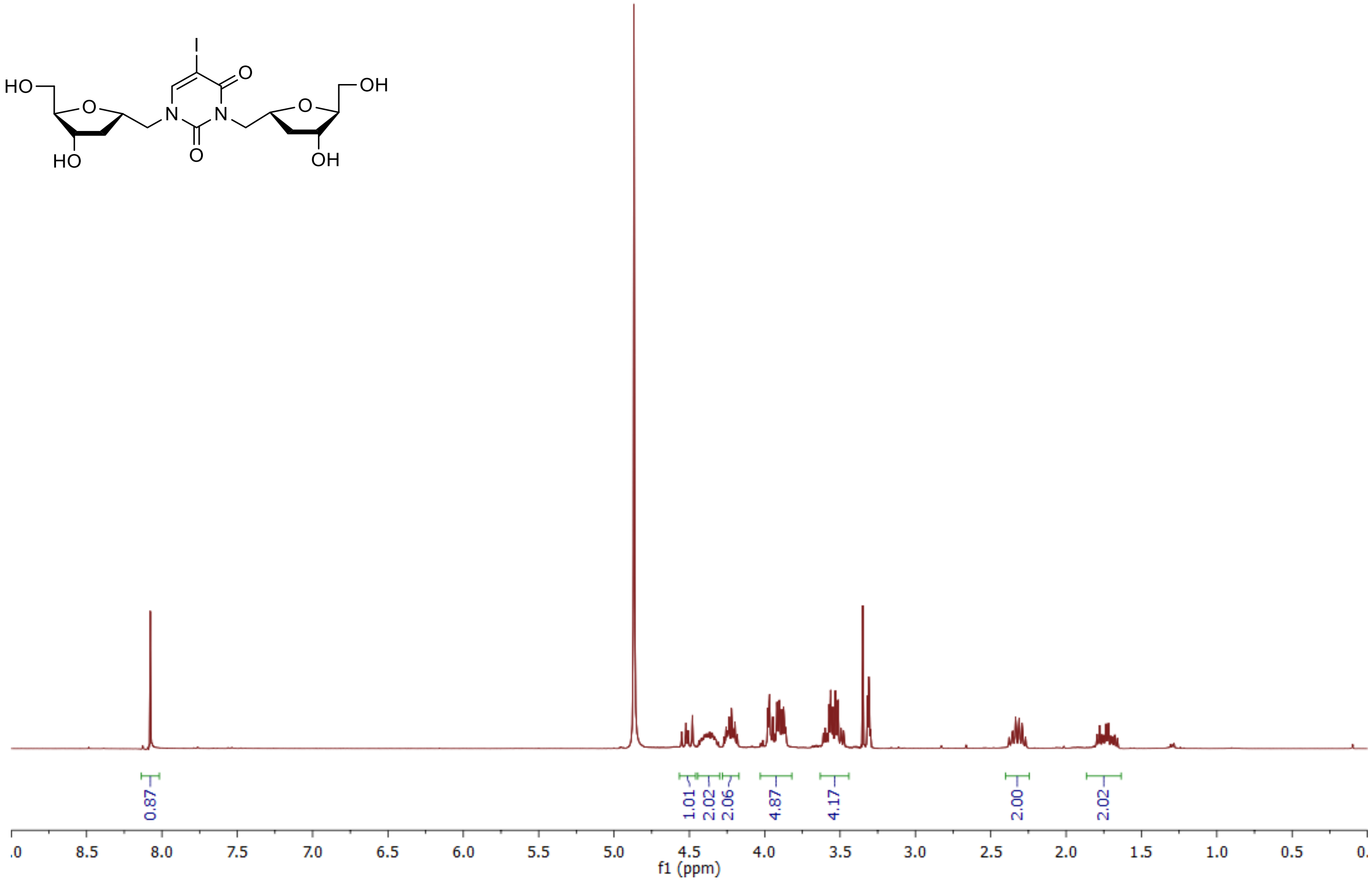

N^1,N^3-bis-(1,2-Dideoxy-α-ribofuranosylmethyl)-5-iodouracil (21f)

^{13}C NMR (75.5 MHz, MeOH-d_4)

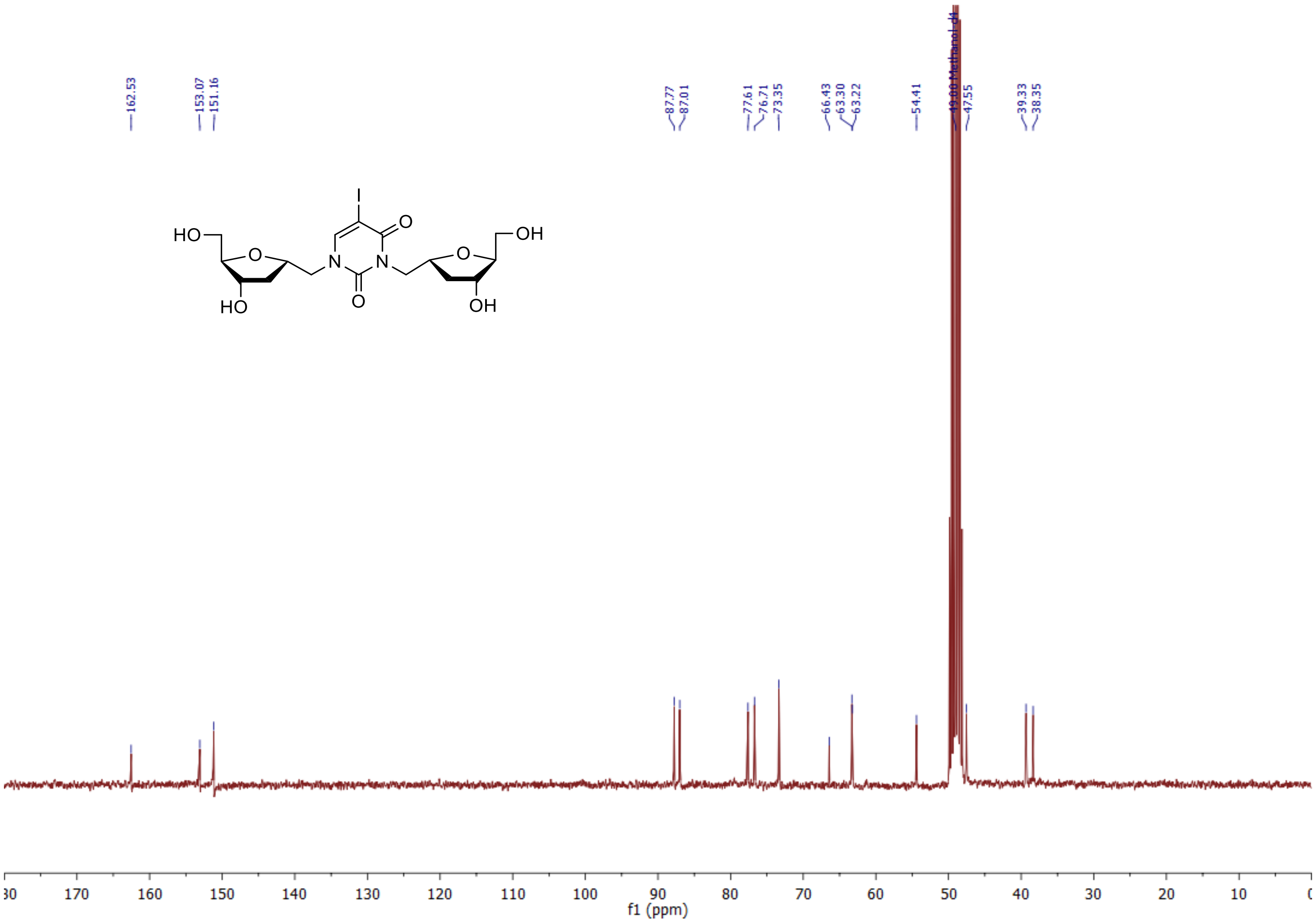

N^1,N^3-bis-(1,2-Dideoxy-α-ribofuranosylmethyl)-5-iodouracil (21f)

DEPT 135 NMR (75.5 MHz, MeOH-d_4)

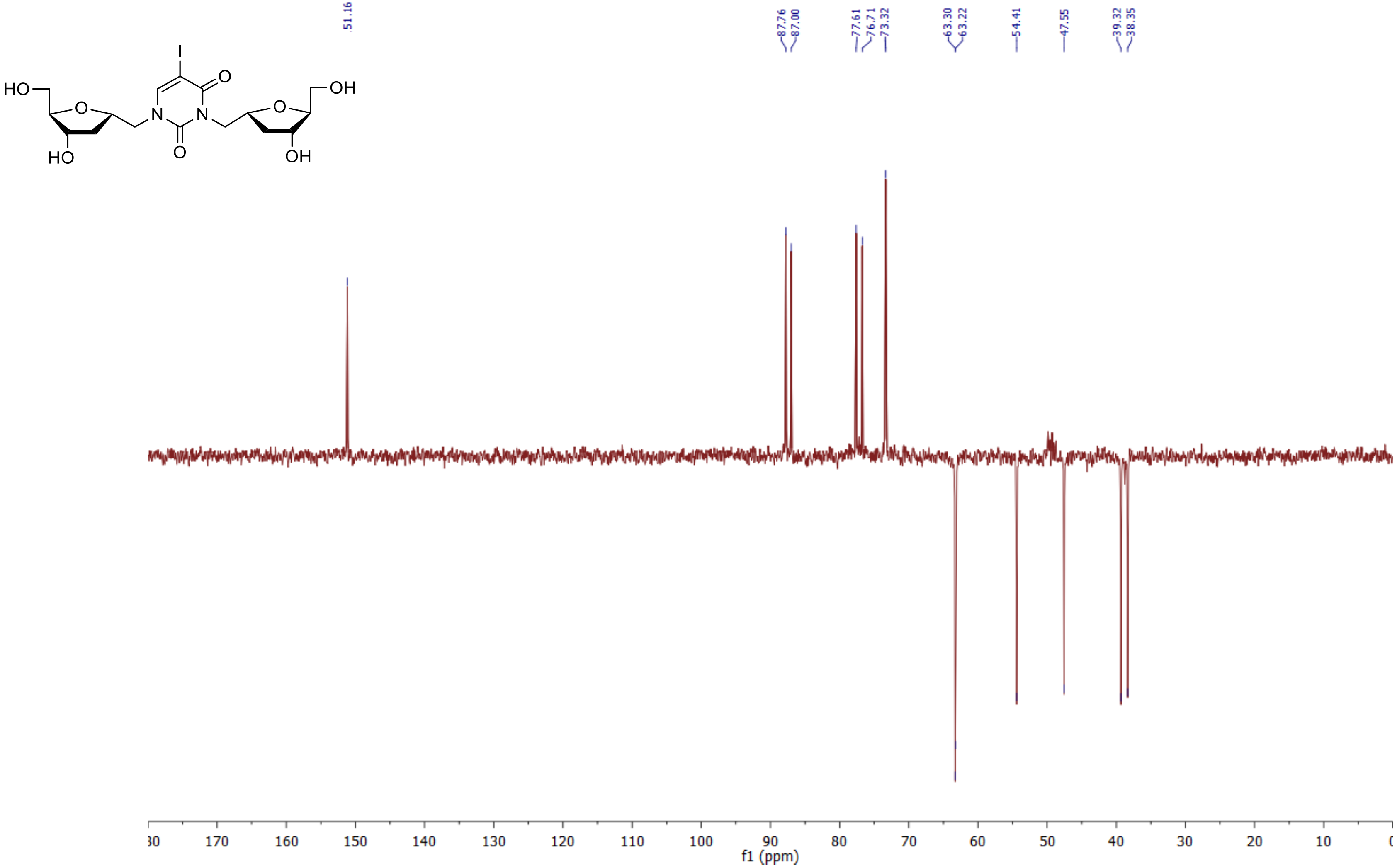

N^1,N^3-bis-(1,2-Dideoxy-α-ribofuranosylmethyl)-5-iodouracil (21f)

COSY NMR (MeOH-d_4)

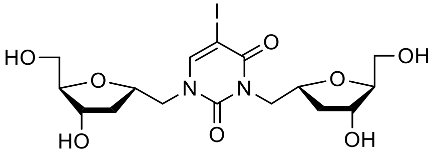

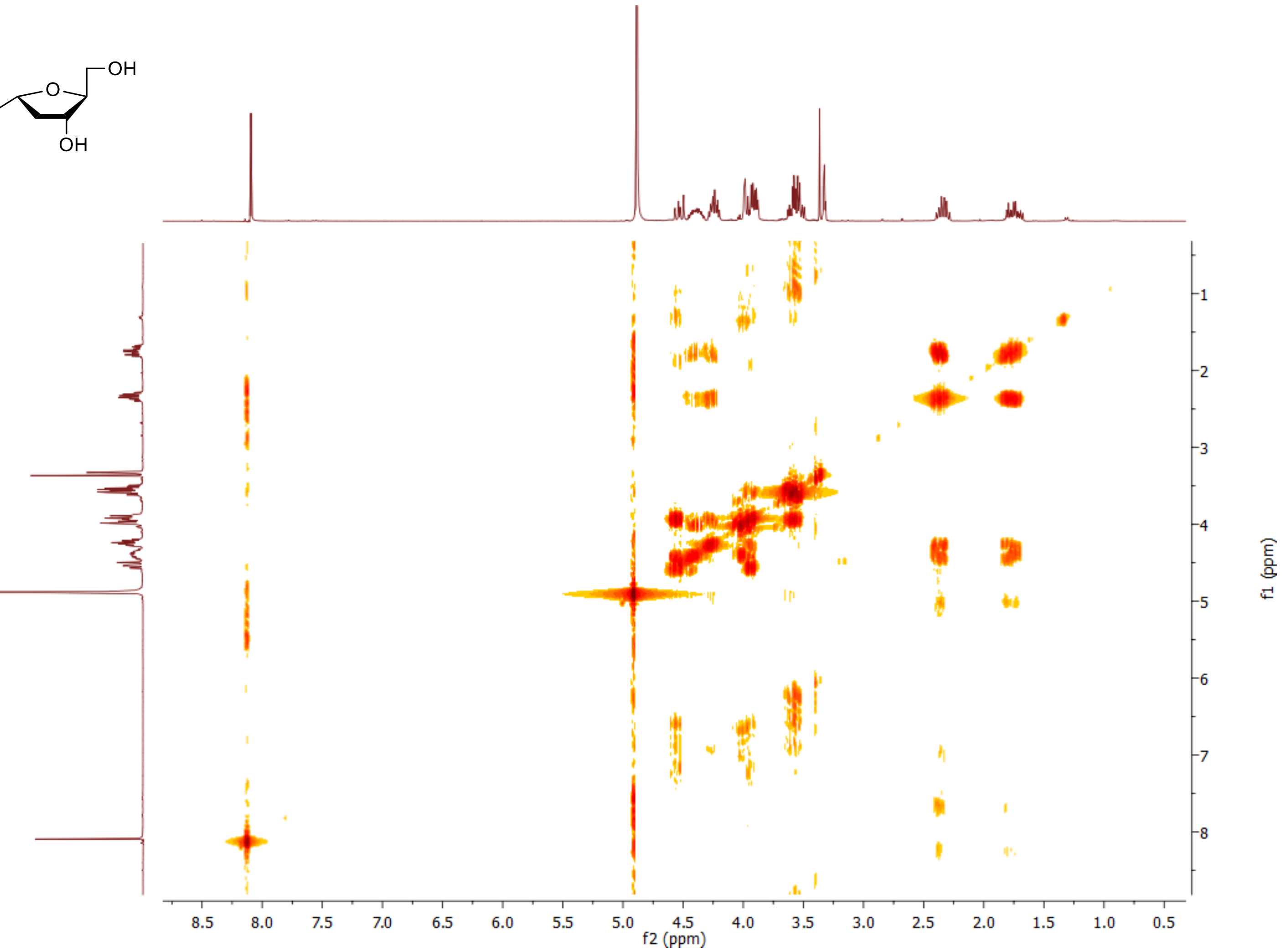

N^1,N^3-bis-(1,2-Dideoxy-α-ribofuranosylmethyl)-5-iodouracil (21f)

HSQC NMR (MeOH-d_4)

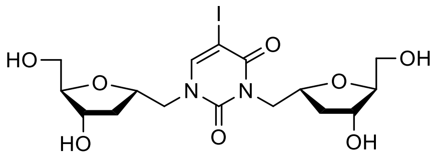

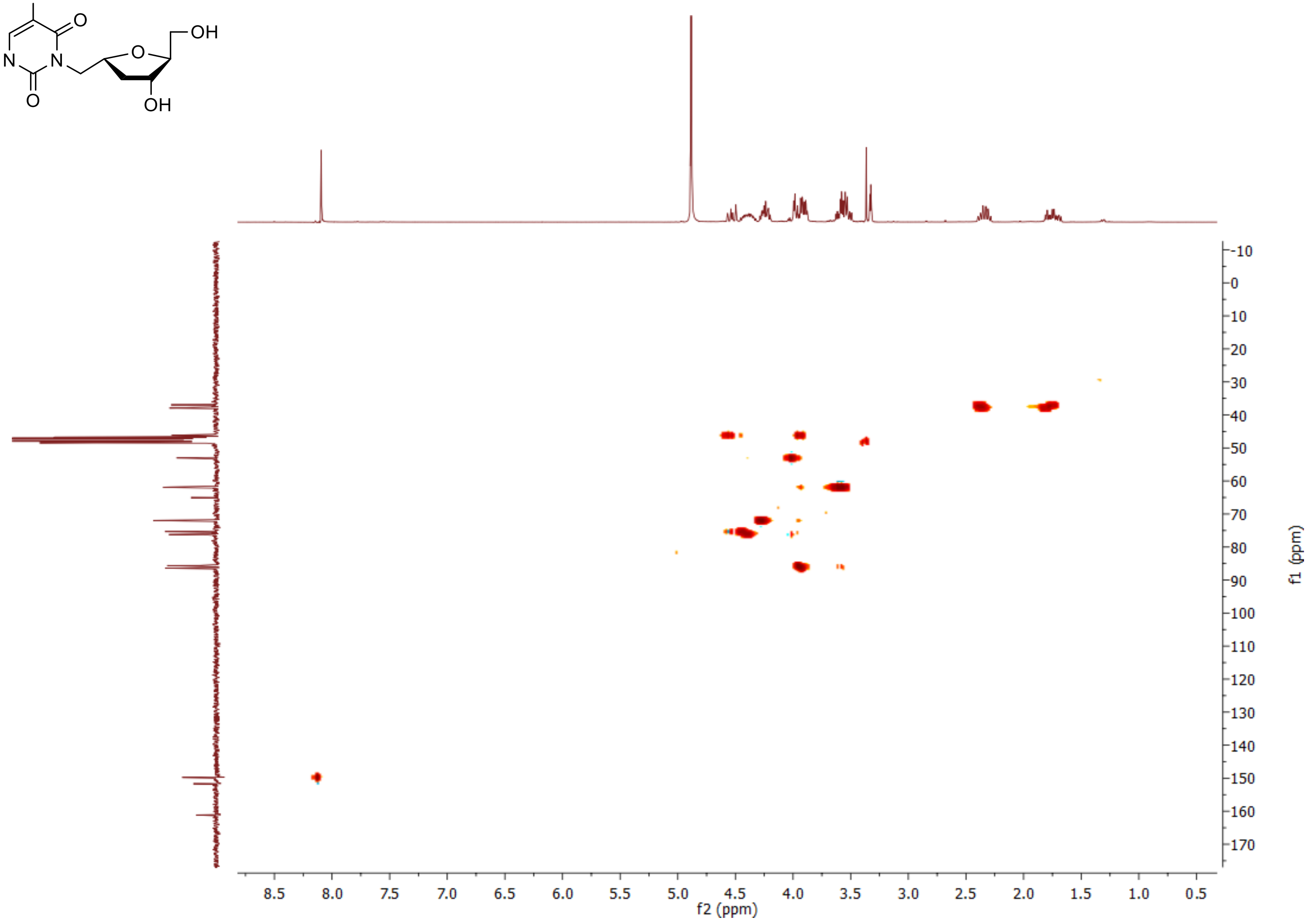

HMBC NMR (MeOH-d_4)

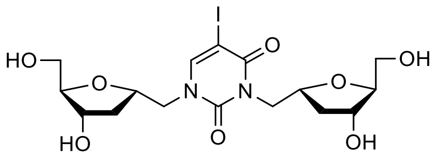

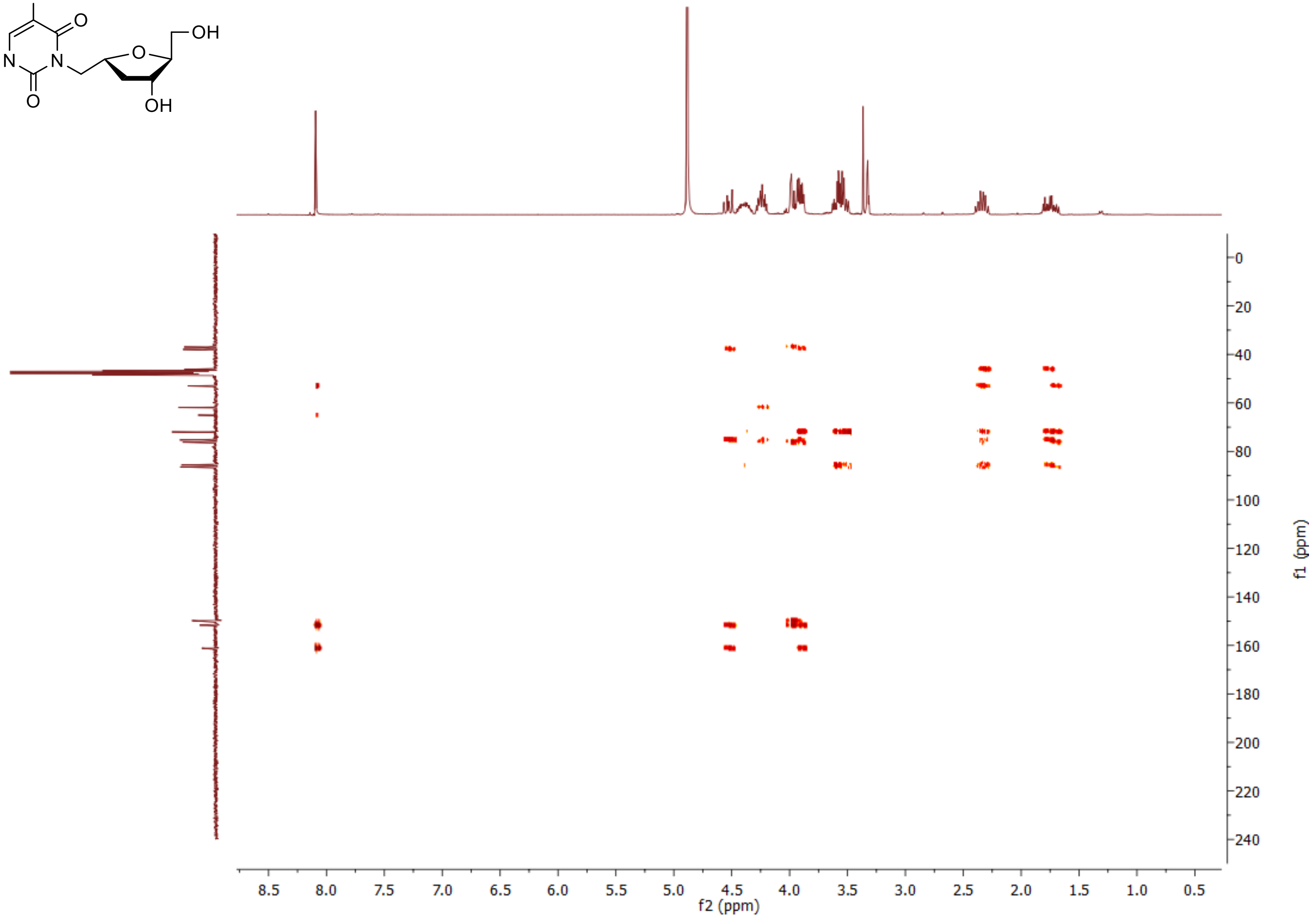

Supplement: RA-010-D0RA03254A-s001 [file RA-010-D0RA03254A-s001.pdf]
